# Diversity and Distribution of *Calonectria* Species in Soils from *Eucalyptus urophylla* × *E*. *grandis*, *Pinus massoniana*, and *Cunninghamia lanceolata* Plantations in Four Provinces in Southern China

**DOI:** 10.3390/jof9020198

**Published:** 2023-02-03

**Authors:** Ying Liu, Shuaifei Chen

**Affiliations:** 1 Research Institute of Fast-Growing Trees (RIFT)/China Eucalypt Research Centre (CERC), Chinese Academy of Forestry (CAF), Zhanjiang 524022, China; 2 Nanjing Forestry University (NJFU), Nanjing 210037, China

**Keywords:** forest pathogen, fungal biodiversity, fungal ecology, phylogeny, plantation tree species, soilborne fungi

## Abstract

The species of *Calonectria* include many notorious plant pathogens and are widely distributed around the world. Leaf blight caused by *Calonectria* species is considered one of the most prominent diseases in *Eucalyptus* plantations in China. Some *Calonectria* species isolated from soils in *Eucalyptus* plantations are highly pathogenic to inoculated *Eucalyptus* genotypes. In southern China, the plantation trees *Cunninghamia lanceolata*, *Eucalyptus* spp., and *Pinus massoniana* are always adjacently planted, especially in FuJian, GuangDong, GuangXi, and YunNan Provinces. The aim of this study was to understand the diversity and distribution of *Calonectria* in soils from plantations of different tree species in different geographic regions. Soil samples were collected from 12 sampling sites in *Eucalyptus urophylla* × *E*. *grandis*, *P*. *massoniana*, and *C*. *lanceolata* plantations in FuJian, GuangDong, GuangXi, and YunNan Provinces. Approximately 250 soil samples were collected from each sampling site, and a total of 2991 soil samples were obtained. A total of 1270 *Calonectria* isolates were obtained from 1270 soil samples. The 1270 isolates were identified based on DNA sequence comparisons of the partial gene regions of *act*, *cmdA*, *his3*, *rpb2*, *tef1*, and *tub2*. These isolates were identified as 11 *Calonectria* species: *Calonectria aconidialis* (69.50%), *C*. *kyotensis* (13.10%), *C*. *hongkongensis* (10.80%), *C*. *ilicicola* (2.50%), *C*. *asiatica* (2.36%), *C*. *curvispora* (0.31%), *C*. *chinensis* (0.24%), *C*. *pacifica* (0.24%), *C*. *yunnanensis* (0.16%), and *C*. *canadiana* (0.08%) in the *C*. *kyotensis* species complex and *C*. *eucalypti* (0.71%) in the *C*. *colhounii* species complex. The three dominant species, *C*. *aconidialis*, *C*. *kyotensis*, and *C*. *hongkongensis*, were widely distributed. The richness of *Calonectria* (percentage of soil samples that yielded *Calonectria*) in soils in the eastern regions (relatively humid regions) was higher than that in the western regions. The *Calonectria* richness of *E*. *urophylla* × *E*. *grandis*, *P*. *massoniana*, and *C*. *lanceolata* plantations decreased gradually. For each of the three dominant species, its richness in the eastern regions was generally higher than that in the western regions; the species richness was highest in *E*. *urophylla* × *E*. *grandis* plantations for *C*. *aconidialis*, while for each of *C*. *kyotensis* and *C*. *hongkongensis,* its species richness was highest in *P*. *massoniana* plantations. The genetic variation in *C*. *aconidialis*, *C*. *kyotensis*, and *C*. *hongkongensis* was more greatly affected by geographic region than by plantation tree species. This study expanded our understanding of the richness, species diversity, and distribution characteristics of *Calonectria* in soils from the plantations of different tree species in different geographic regions in southern China. Results in this study enhanced our understanding of the influencing characteristics of geographic region and tree species on the species and genetic diversity of soilborne fungi.

## 1. Introduction

The genus *Calonectria* includes a range of important plant pathogens that are widely distributed in tropical, subtropical, and temperate regions throughout the world [1,2,3]. These aggressive pathogens can infect approximately 335 plant species residing in 100 plant families, including important agricultural, horticultural, and forestry crops [1,2,3,4]. Leaf blight caused by *Calonectria* species is considered one of the most prominent diseases of *Eucalyptus* plantations in China, Brazil, Colombia, Vietnam, and other countries [5,6,7,8,9,10,11,12]. *Calonectria* species also produce other disease symptoms, including stem and root rot, branch canker, and shoot blight on *Eucalyptus* [1,3,10,12]. Besides *Eucalyptus*, *Calonectria* also causes disease in other plantation tree species, such as *Acacia* spp. and *Pinus* spp. [1,5,13].

Calonectria leaf blight (CLB) has become a major threat to *Eucalyptus* plantations in China and has caused significant economic losses [10,12,14,15]. In the past decade, eleven *Calonectria* species, namely *Calonectria aciculata*, *C*. *aconidialis*, *C*. *cerciana*, *C*. *crousiana*, *C*. *eucalypti*, *C*. *fujianensis*, *C*. *hawksworthii*, *C*. *pauciramosa*, *C*. *pseudoretedudii C*. *queenslandica*, and *C*. *reteaudii*, have been associated with leaf blight of *Eucalyptus* in plantations in China [10,12,15,16,17,18,19]. CLB has been found on a number of *Eucalyptus* species and hybrids, including several *E*. *urophylla* × *E*. *grandis* and *E*. *urophylla* × *E*. *tereticornis* genotypes, which are widely planted in southern China [10,12,15,16,17,18,19].

In China, plantation forestry has grown rapidly due to the increasing demand for wood and pulp [20,21]. China’s domestic wood is mainly produced by plantations in southern regions. The main plantation tree species include *Cunninghamia lanceolata*, *Eucalyptus* spp., and *Pinus massoniana* [20,22,23]. The plantation areas of *C*. *lanceolata*, *Eucalyptus* spp., and *Pinus massoniana* cover 9.9, 5.4, and 2.5 million hectares, respectively [24,25,26,27]. *Cunninghamia lanceolata* is the most planted species. *Cunninghamia lanceolata* plantations account for almost 12.4% of the country’s total plantation area [25]. *Eucalyptus* plantations account for about 6.5% of the country’s total plantation area and provide more than one-third of the total annual domestic timber output [24,27]. Plantations of *C*. *lanceolata*, *Eucalyptus* spp., and *P*. *massoniana* are always adjacently planted in southern China, especially in FuJian, GuangDong, GuangXi, and YunNan Provinces. These plantations provide many timber resources and bring great ecological benefits to China [20,23].

To date, 34 *Calonectria* species have been identified and described in China based on DNA sequence comparisons and morphological characteristics [12,15,16,18,19,28,29,30,31,32,33]. Previous research results have indicated that *Calonectria* species are frequently isolated from *Eucalyptus* plantations. Eleven and sixteen *Calonectria* species were isolated from diseased tissues and soils, respectively, in *Eucalyptus* plantations. *Calonectria pseudoreteaudii*, *C*. *reteaudii*, *C*. *aconidialis*, and *C*. *cerciana* have been isolated from both diseased *Eucalyptus* tissues and soils in *Eucalyptus* plantations [10,12,18,19,30,31,32,34].

Previous research results have shown that *Calonectria* species isolated from diseased *Eucalyptus* tissues in China are pathogenic to tested *Eucalyptus* genotypes [10,12,16,19]. Research results in our previous research work indicated that *C*. *aconidialis*, *C*. *auriculiformis*, *C*. *chinensis*, *C*. *hongkongensis*, *C*. *ilicicola*, *C*. *kyotensis*, *C*. *orientalis*, *C*. *pseudoreteaudii*, and *C*. *reteaudii* isolated from soils under *Eucalyptus* plantations are pathogenic to tested *Eucalyptus* genotypes [19,35]. All of these species cause leaf spot, leaf blight, and seedling rot to the tested *Eucalyptus* plants within three days after inoculation [19,35]. Research results in our recent studies further showed that *Calonectria* species are found in soils associated with *C*. *lanceolata* and *P*. *massoniana* in southern China [32].

Currently, several studies have been conducted to understand *Calonectria* species diversity in the soils of *Eucalyptus* plantations, while little information is known about the species diversity of *Calonectria* in the soil of plantations other than *Eucalyptus* [19,32]. The aims of this study were to (i) understand the richness and species diversity of *Calonectria* in the soil of adjacent plantations of *Eucalyptus urophylla × E*. *grandis*, *C*. *lanceolata* and *P*. *massoniana* in southern China; and (ii) understand the diversity and distribution characteristics of *Calonectria* species affected by plantation tree species and geographical regions.

## 2. Materials and Methods

### 2.1. Study Site, Soil Sample Collection, and Calonectria Isolation

**Figure 1:**
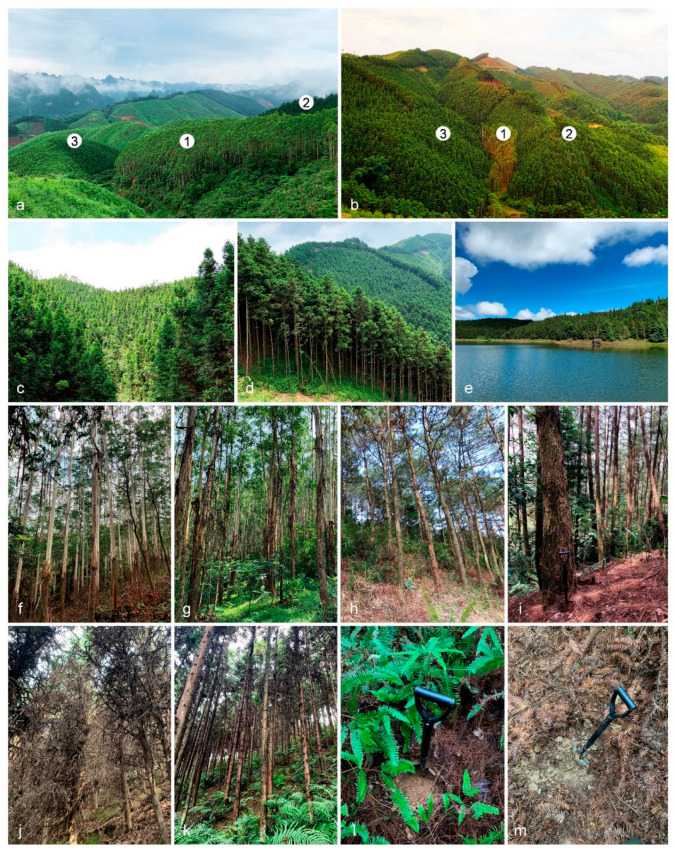
Plantations of *Eucalyptus urophylla* × *E*. *grandis*, *Pinus massoniana*, and *Cunninghamia lanceolata* in Fujian, Guangdong, Guangxi, and Yunnan Provinces in southern China. (**a**,**b**). The adjacently planted *E*. *urophylla* × *E*. *grandis* (indicated by number “1”), *P*. *massoniana* (number “2”), and *C*. *lanceolata* (number “3”) in GuangXi; **c**–**e**. *Cunninghamia lanceolata* plantations in Guangdong (**c**), Guangxi (**d**), and Yunnan (**e**); (**f**,**g**). *Eucalyptus urophylla* × *E. grandis* plantations in Fujian (**f**) and Guangdong (**g**); (**h**,**i**). *Pinus massoniana* plantations in Fujian (**h**) and Guangxi (**i**); (**j**,**k**). *Cunninghamia lanceolata* plantations in Fujian (**j**) and Guangxi (**k**); (**l**). Soil in *P*. *massoniana* plantation in Guangxi; (**m**). Soil in *C*. *lanceolata* plantation in FuJian.

**Figure 2:**
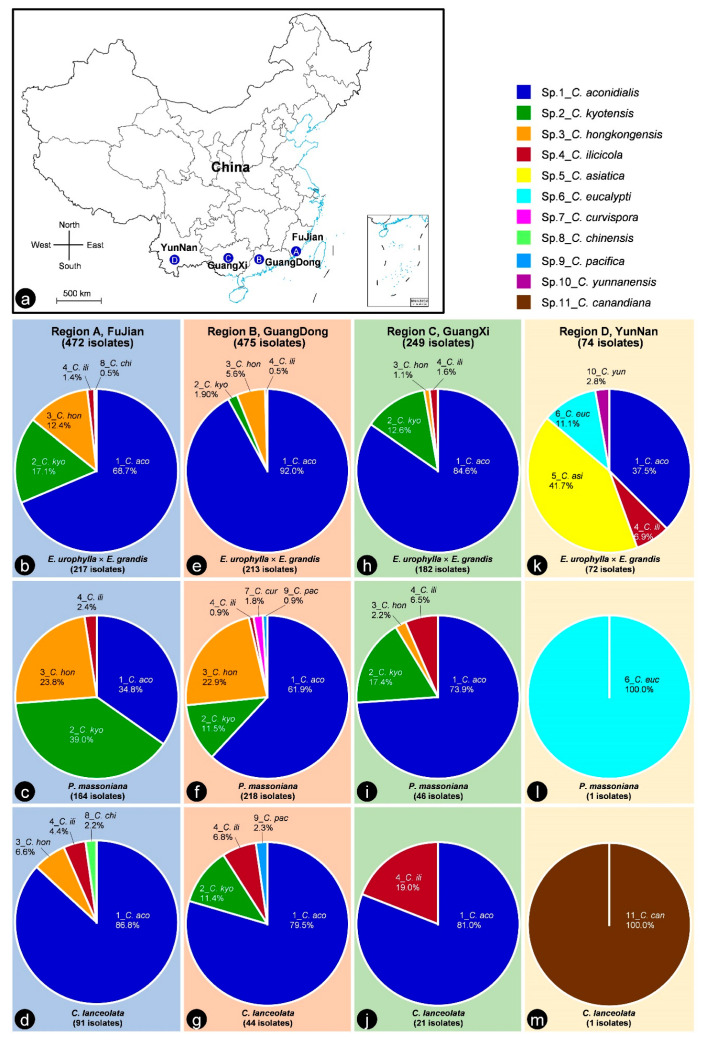
*Calonectria* species collected from soils in plantations of three tree species in four regions (provinces). (**a**). Map of China indicating the four regions in four provinces where soils were sampled; (**b**–**m**). Percentage of each *Calonectria* species in each plantation of *Eucalyptus urophylla* × *E*. *grandis*, *Pinus massoniana*, and *Cunninghamia lanceolata* in each of the four regions (FuJian, GuangDong, GuangXi, and YunNan Provinces). Different *Calonectria* species are indicated by numbers with different colors.

Soil samples were collected from plantations regions where *E*. *urophylla* × *E*. *grandis*, *P*. *massoniana*, and *C*. *lanceolata*, were adjacently planted (Figure 1). We tried to select the regions in which the three plantations were connected, to ensure the soil types of the three plantations (sites) in each region were similar. These samples were collected from four plantation regions in each of FuJian, GuangDong, GuangXi, and YunNan Provinces (Figure 2a, Table 1). The latitudes of the four sampled regions were similar. The distances between adjacent regions were 300–500 km (Figure 2a). The areas of each plantation of *E*. *urophylla* × *E*. *grandis*, *P*. *massoniana*, and *C*. *lanceolata* in each region were around 50 hectares. For each of the four selected regions, *E*. *urophylla* × *E*. *grandis*, *P*. *massoniana*, and *C*. *lanceolata* trees were planted for more than 10 years, although the *E*. *urophylla* × *E*. *grandis* trees were 5–6 years old. According to the size of the *E*. *urophylla* × *E*. *grandis* stumps, it is clear that *E*. *urophylla* × *E*. *grandis* trees had been planted in the relative regions for at least one more rotation period (5–6 years) before our soil sampling. We estimated that the period of *E*. *urophylla* × *E*. *grandis* trees planted was more than ten years (Table 1). *Pinus massoniana* and *C*. *lanceolata* trees were 15–20 and 10–20 years old, respectively (Table 1).

Soil samples were collected from each of 12 sampling sites (4 regions × 3 sites /region) (Table 1). In each of the 12 sites, approximately 250 soil samples were collected. We adopted a “Z”-shaped random sampling pattern, collecting soil every 10 m at each of the 12 sites (plantations). The plantations typically had thick layers of leaf litter, which were removed before soil sample collection. Soil samples were collected from the upper 0–20 cm of the humid soil profile. Each soil sample was placed in a resealable plastic bag and transferred to the laboratory for isolation and further molecular analyses. Soil samples were collected from May to July 2021.

To obtain *Calonectria* isolates, each soil sample was thoroughly mixed and transferred to a plastic cylinder sampling cup (diameter = 4.5 cm, height = 5 cm, and volume = 80 mL) (Chengdu Rich Science Industry Co., Ltd., Chengdu, China). The soil sample took up half to two-thirds of the whole sampling cup volume. The soil sample was moistened by spraying it with sterile water, and it was mixed well with a sterilized bamboo stick. After a superficial sterilization (soaked 30 s in 75% ethanol and washed several times with sterile water), thirty to fifty *Medicago sativa* (alfalfa) seeds were scattered onto the soil surface in each sampling cup. Treated sampling cups with soil and alfalfa seeds were placed in an alternating environment of 12 h of daylight and 12 h of darkness and incubated at 25 °C for six to seven days until white masses of conidiophores with typical morphological characteristics of *Calonectria* species [1] were observed on infected alfalfa tissue. Using a dissecting microscope (AxioCam Stemi 2000C, Carl Zeiss, Ltd., Jena, Germany), every single one conidial mass was selected and scattered onto 2% malt extract agar (MEA) (20 g malt extract powder and 20 g agar powder per liter of water: malt extract powder was obtained from Beijing Shuangxuan microbial culture medium products factory, Beijing, China; the agar powder was obtained from Beijing Solarbio Science and Technology Co., Ltd., Beijing, China) using a sterile needle. After incubation at 25 °C for three to four hours, germinated conidia were individually transferred onto fresh MEA under a dissecting microscope and incubated at 25 °C for one week to obtain single-conidium cultures. One single-conidium culture was obtained from each soil sample with white masses of conidiophores. All obtained single conidium cultures were deposited in the culture collection (CSF) at the Research Institute of Fast-growing Trees (RIFT) of the Chinese Academy of Forestry (CAF) in ZhanJiang, GuangDong Province, China.

### 2.2. DNA Extraction, PCR Amplifications, and Sequencing

All *Calonectria* morphological-like isolates obtained in this study were used for total genomic DNA extraction and sequence comparisons. Mycelia were scraped from 7-day-old cultures using a sterilized scalpel and transferred into 2 mL Eppendorf tubes. Total genomic DNA was extracted using the cetyltrimethylammonium bromide (CTAB) protocol described by Van Burik and co-authors [36]. The extracted DNA was dissolved by adding 30 µL TE buffer (1 M Tris-HCl and 0.5 M EDTA, pH 8.0), and 2.5 µL RNase (10 mg/mL) was added to degrade the RNA. The mixture was incubated at 37 °C for 1 h. The DNA concentration was quantified using a NanoDrop 2000 spectrometer (Thermo Fisher Scientific, Waltham, MA, USA). All DNA samples were diluted to approximately 100 ng/uL with DNase/RNasefree ddH_2_O (Sangon Biotech Co., Ltd., Shanghai, China) and stored at –20 °C for further use.

Based on previous research results, partial gene regions of actin (*act*), calmodulin (*cmdA*), histone H3 (*his3*), the DNA-directed RNA polymerase II second largest subunit (*rpb2*), translation elongation factor 1-alpha (*tef1*), and β-tubulin (*tub2*) served as reliable DNA barcodes to clearly distinguish species in *Calonectria* [19,30,31]. The primer pairs ACT-512F/ACT-783R, CAL-228F/CAL-2Rd, CYLH3F/CYLH3R, fRpb2-5F/fRpb2-7cR, EF1-728F/EF2, and T1/CYLTUB1R were used to amplify the fragments of *act*, *cmdA*, *his3*, *rpb2*, *tef1*, and *tub2* genes, respectively [30]. The PCR reactions were conducted as described by Liu and co-authors [30].

To ensure the accuracy and integrity of all sequences, all PCR products were sequenced in both the forward and reverse directions using the same primers used for PCR amplification. Sequence reactions were performed by the Beijing Genomics Institute, Guangzhou, China. All obtained sequences were edited and assembled using MEGA v. 7.0 software [37] and deposited in GenBank (https://www.ncbi.nlm.nih.gov; accessed date: 24 January 2023).

For all the *Calonectria* morphological-like isolates, the *tef1* gene regions were sequenced, and a standard nucleotide BLAST search was conducted using the *tef1* sequences to preliminarily identify these fungi. For all isolates preliminarily identified as *Calonectria*, the *tub2* gene regions were then sequenced. All obtained *Calonectria* isolates were genotyped by the *tef1* and *tub2* sequences. Based on the genotypes generated by *tef1* and *tub2* sequences, isolates for each *tef1*-*tub2* genotype obtained from different regions and plantation tree species were selected for sequencing the *act*, *cmdA*, *his3*, and *rpb2* gene regions.

### 2.3. Multi-Gene Phylogenetic Analyses and Species Identification

All sequences of the six gene regions (*act*, *cmdA*, *his3*, *rpb2*, *tef1*, and *tub2*) generated in this study were compared with the sequences of type specimen strains of published *Calonectria* species. Sequences of all published species in the relevant species complexes were used for sequence comparisons and phylogenetic analyses. The datasets of Liu and co-authors [30] were used as templates, and the sequences of other recently described *Calonectria* species in the relevant species complexes were all used for sequence comparisons.

Sequences of each of the *act*, *cmdA*, *his3*, *rpb2*, *tef1*, and *tub2* gene regions, as well as the combination of these six gene regions, were aligned using the online version of MAFFT v. 7 (http://mafft.cbrc.jp/alignment/server; accessed date: 10 August 2022) with the alignment strategy FFT-NS-i (Slow; interactive refinement method). The alignments were manually edited using MEGA v. 7.0 software [37] when necessary. All alignments used for phylogenetic analyses were submitted to TreeBASE (http://treebase.org; accessed date: 15 August 2022).

The Maximum likelihood (ML) and Bayesian inference (BI) approaches were used for phylogenetic analyses of the sequence datasets of each of the six genes and the combined dataset of all six gene regions. ML analyses were conducted using RaxML v. 8.2.4 [38] on the CIPRES Science Gateway v. 3.3. BI analyses were conducted using MrBayes v. 3.2.6 [39] on the CIPRES Science Gateway v. 3.3. ML analyses were performed with a default GTR substitution matrix and 1000 bootstrap replicates. For BI analyses, four Markov chain Monte Carlo (MCMC) chains were run from a random starting tree for five million generations, and trees were sampled every 100th generation. The first 25% of the trees sampled were discarded as burn-in, and the remaining trees were used to determine the posterior probabilities. Two isolates of *Curvicladiella cignea* (CBS 109167 and CBS 109168) were used as outgroup taxa [30]. Phylogenetic trees generated by ML and BI analyses were viewed using MEGA v. 7.0. [37] and Fig Tree v. 1.4.3 (http://tree.bio.ed.ac.uk/software/figtree/; accessed date: 2 September 2022), respectively.

### 2.4. Calonectria Richness in Soils from Four Provinces and Plantations of Three Tree Species

The *Calonectria* isolates obtained in this study were identified. The numbers of *Calonectria* isolates obtained at each of the 12 sampling sites were counted. Furthermore, the percentage of soil samples that yielded *Calonectria* (*Calonectria* richness) at each sampling site was computed. The distribution characteristics of *Calonectria* in four regions (provinces) and plantations of three tree species were recorded, including the influencing characteristics of *Calonectria* richness by geographic region (provinces) and plantation tree species.

### 2.5. Calonectria Species Diversity in Four Provinces and Plantations of Three Tree Species

According to the species identification results of all isolates, the number of isolates of each *Calonectria* species obtained at each of the 12 sampling sites was counted. The percentage of soil samples that yielded each *Calonectria* species at each sampling site was also computed. The distribution characteristics of each *Calonectria* species in four provinces and plantations of three tree species were recorded, including the influencing characteristics of *Calonectria* species number and each species richness by geographic region (provinces) and plantation tree species.

### 2.6. Genotyping of Isolates within each Calonectria Species

The genotypes of the isolates within each identified *Calonectria* species were determined based on the *tef1* and *tub2* sequences. The number of genotypes of each species and the number of isolates belonging to each genotype were recorded. Furthermore, the number of genotypes of each *Calonectria* species in each of the 12 plantations (12 sampling sites) of three tree species in four provinces was counted.

### 2.7. Genotype Diversity of Calonectria Species in Four Provinces and Plantations of Three Tree Species

For each dominant *Calonectria* species, to preliminarily understand whether its genetic variation (based on shared genotype) was affected by geographical regions and plantation tree species, the numbers of shared genotypes among isolates at 12 sampling sites were counted. We further compared the number of shared genotypes for each dominant species to evaluate the influencing characteristics of geographical regions (provinces) and plantation tree species on the genetic variations of each dominant species.

## 3. Results

### 3.1. Soil Sample Collection and Calonectria Isolation

A total of 2991 soil samples were collected, with 244–250 soil samples from each of the 12 sampling sites (Table 1). After the soil samples were incubated with alfalfa seeds, a single-conidium culture was obtained from each soil sample with white masses of conidiophores with typical morphological characteristics of *Calonectria* species. In total, 1308 *Calonectria* morphological-like isolates were obtained.

### 3.2. Sequencing

For all 1308 *Calonectria* morphological-like isolates obtained from soil samples, the *tef1* gene sequences were amplified and used to conduct a standard nucleotide BLAST search to preliminarily identify the species. Ultimately, 1270 isolates were identified as *Calonectria* species (Appendix A Table A1). The majority of the remaining 38 isolates were grouped into the genus *Cylindrocladiella*. The *tub2* gene region was also amplified and sequenced for the 1270 *Calonectria* isolates (Appendix A Table A1). Ninety-seven *tef1*-*tub2* genotypes were generated based on the *tef1* and *tub2* gene sequences (Table 2). Subsequently, 207 isolates were selected to amplify the *act*, *cmdA*, *his3*, and *rpb2* gene regions. These 207 isolates presented all three tree species in all four sampling regions (provinces), and presented all 97 genotypes based on *tef1* and *tub2* gene sequences (Table 3). One to ten isolates of each genotype revealed by the *tef1* and *tub2* sequences were selected (Table 2 and Table 3). Amplicons generated for the *act*, *cmdA*, *his3*, *rpb2*, *tef1*, and *tub2* gene regions were approximately 235, 680, 430, 1030, 500, and 620 bp, respectively.

### 3.3. Multi-Gene Phylogenetic Analyses and Species Identification

The standard nucleotide BLAST search results conducted using the *act*, *cmdA*, *his3*, *rpb2*, *tef1*, and *tub2* gene sequences showed that the isolates obtained in the current study belonged to two species complexes of *Calonectria*, the *C*. *kyotensis* species complex and the *C*. *colhounii* species complex. The 207 *Calonectria* isolates with six sequenced gene regions were used for phylogenetic analyses (Table 3). Based on the published results in Liu and co-authors [30] and several recent publications [29,32,33,40,41,42], sequences of *act*, *cmdA*, *his3*, *rpb2*, *tef1*, and *tub2* of 44 published species in the *C*. *kyotensis* species complex and *C*. *colhounii* species complex were downloaded from GenBank and used for sequence comparisons and phylogenetic analyses (Table 4).

**Figure 3:**
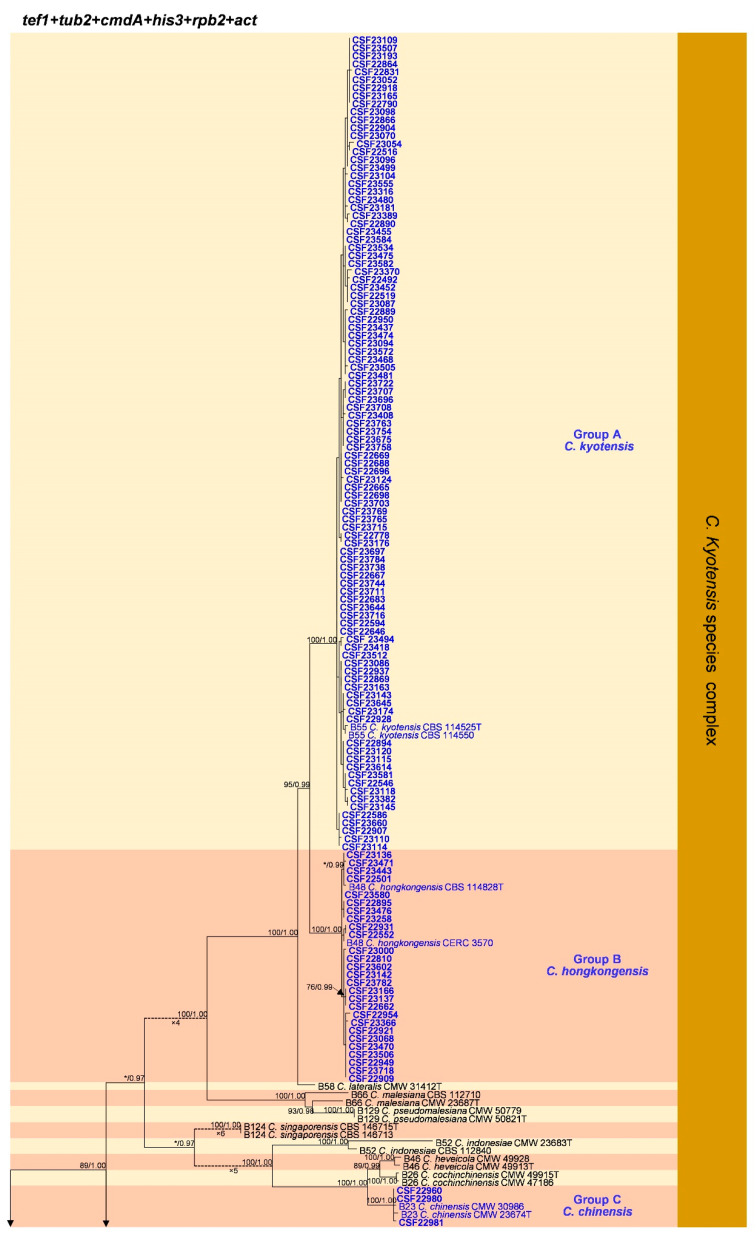

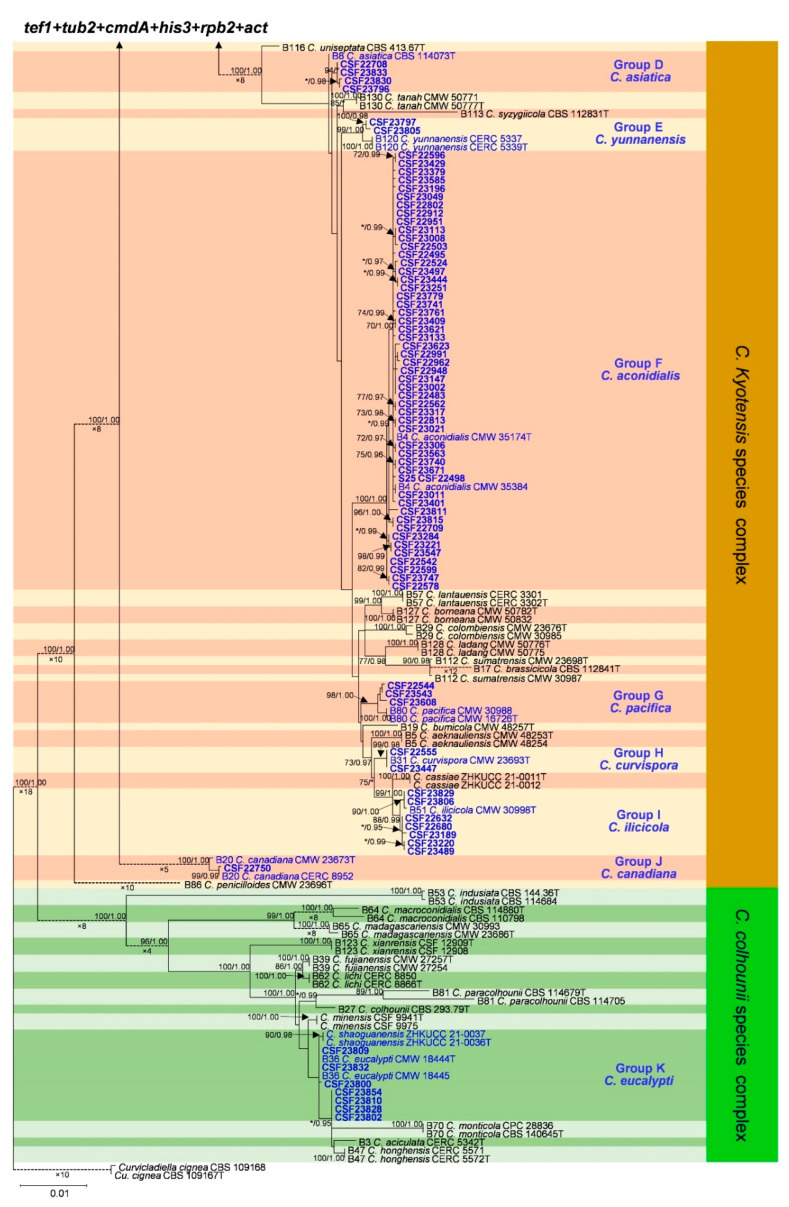
Phylogenetic tree of *Calonectria* species based on maximum likelihood (ML) analysis of the combined DNA dataset of *act*, *cmdA*, *his3*, *rpb2*, *tef1*, and *tub2* gene sequences. Bootstrap support values ≥ 70% from ML analysis and posterior probabilities values ≥ 0.95 obtained from Bayesian inference (BI) are indicated at the nodes as ML/BI. Bootstrap values < 70% or posterior probabilities values < 0.95 are marked with “*”, and absent analysis values are marked with “-”. “*/*”, “*/-”, “-/*”, and “-/-” are not displayed. Isolates obtained in this study are highlighted in blue and bold. Ex-type isolates are indicated with “T”. The “B” species codes are consistent with the recently published results of Liu and co-authors [30]. *Curvicladiella cignea* (CBS 109167 and CBS 109168) was used as the outgroup taxon.

**Figure A1:**
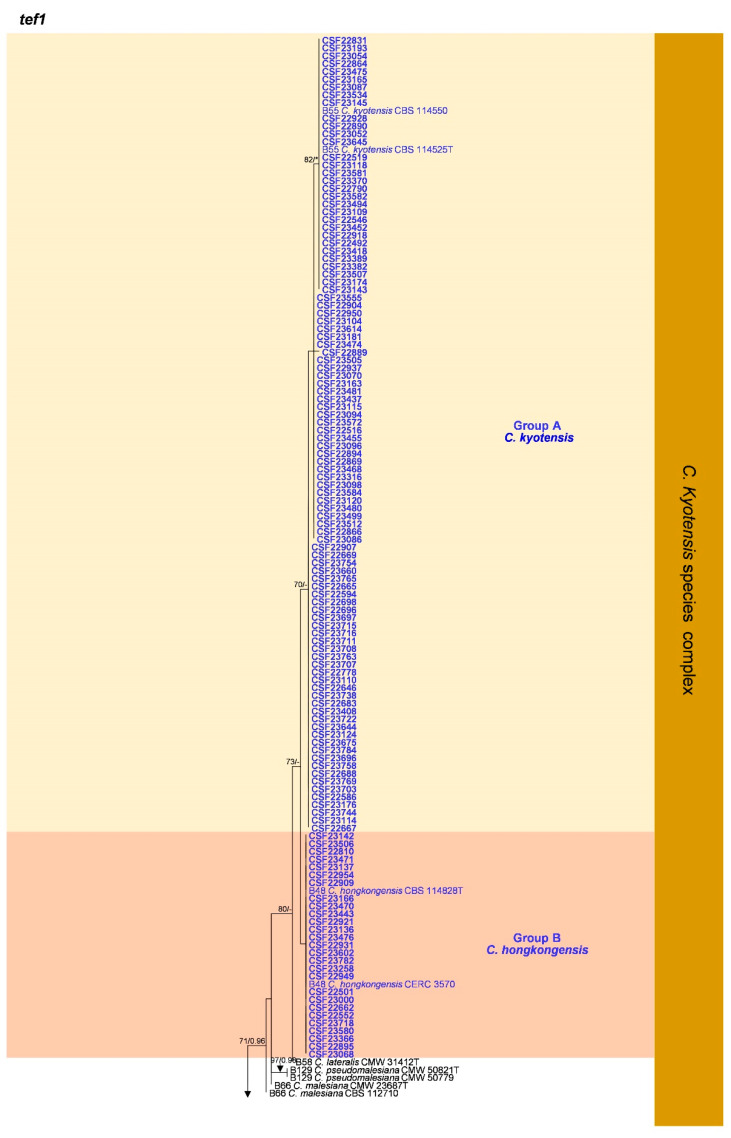

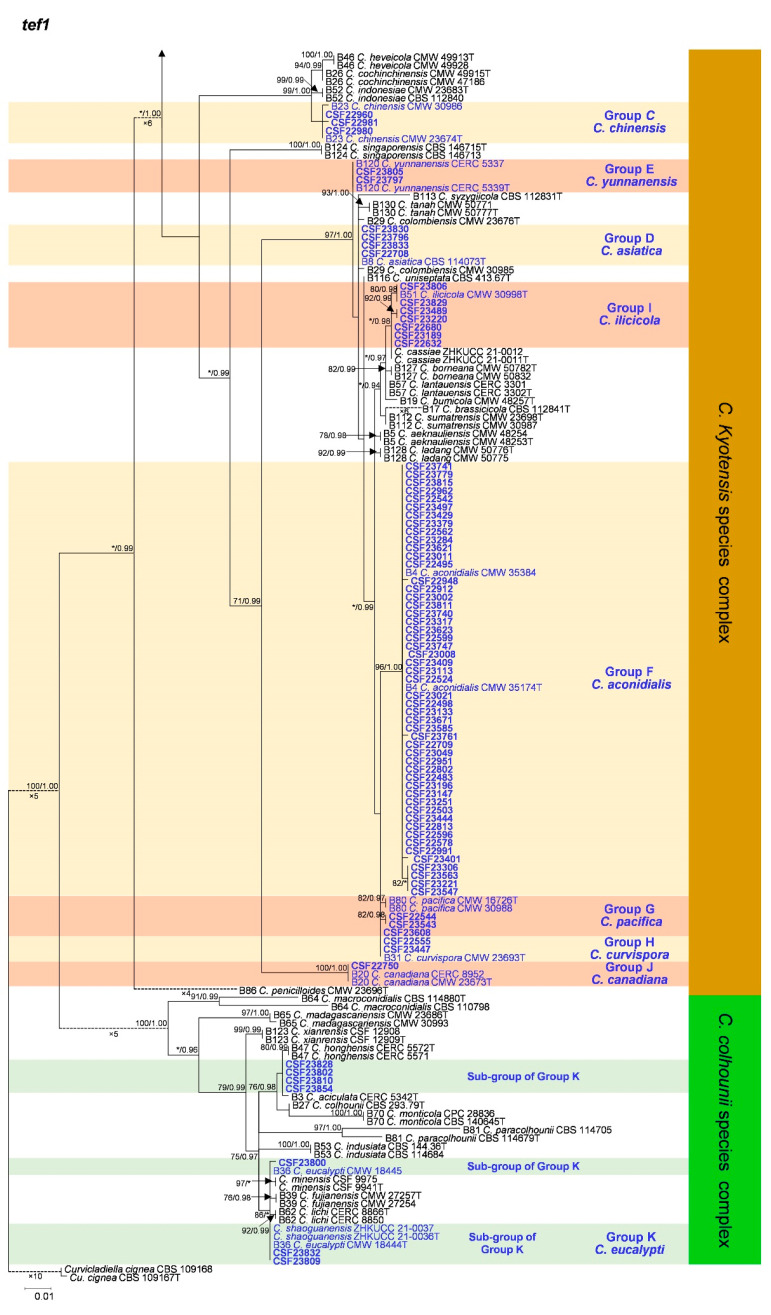
Phylogenetic tree of *Calonectria* species based on maximum likelihood (ML) analysis of the DNA dataset of the *tef1* gene sequences. Bootstrap support values ≥ 70% from ML analysis and posterior probabilities values ≥ 0.95 obtained from Bayesian inference (BI) are indicated at the nodes as ML/BI. Bootstrap values < 70% or posterior probabilities values < 0.95 are marked with “*”, and absent analysis values are marked with “-”. “*/*”, “*/-”, “-/*”, and “-/-” are not displayed. Isolates obtained in this study are highlighted in blue and bold. Ex-type isolates are indicated with “T”. The “B” species codes are consistent with the recently published results of Liu and co-authors [30]. *Curvicladiella cignea* (CBS 109167 and CBS 109168) was used as the outgroup taxon.

**Figure A2:**
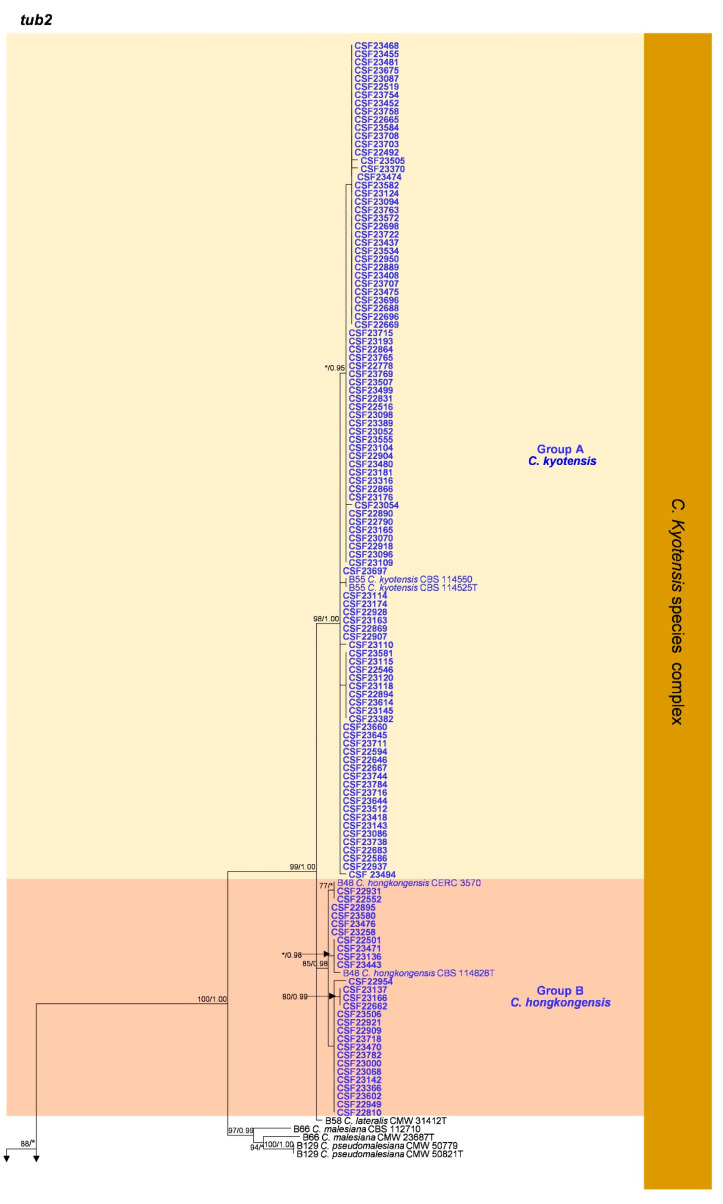

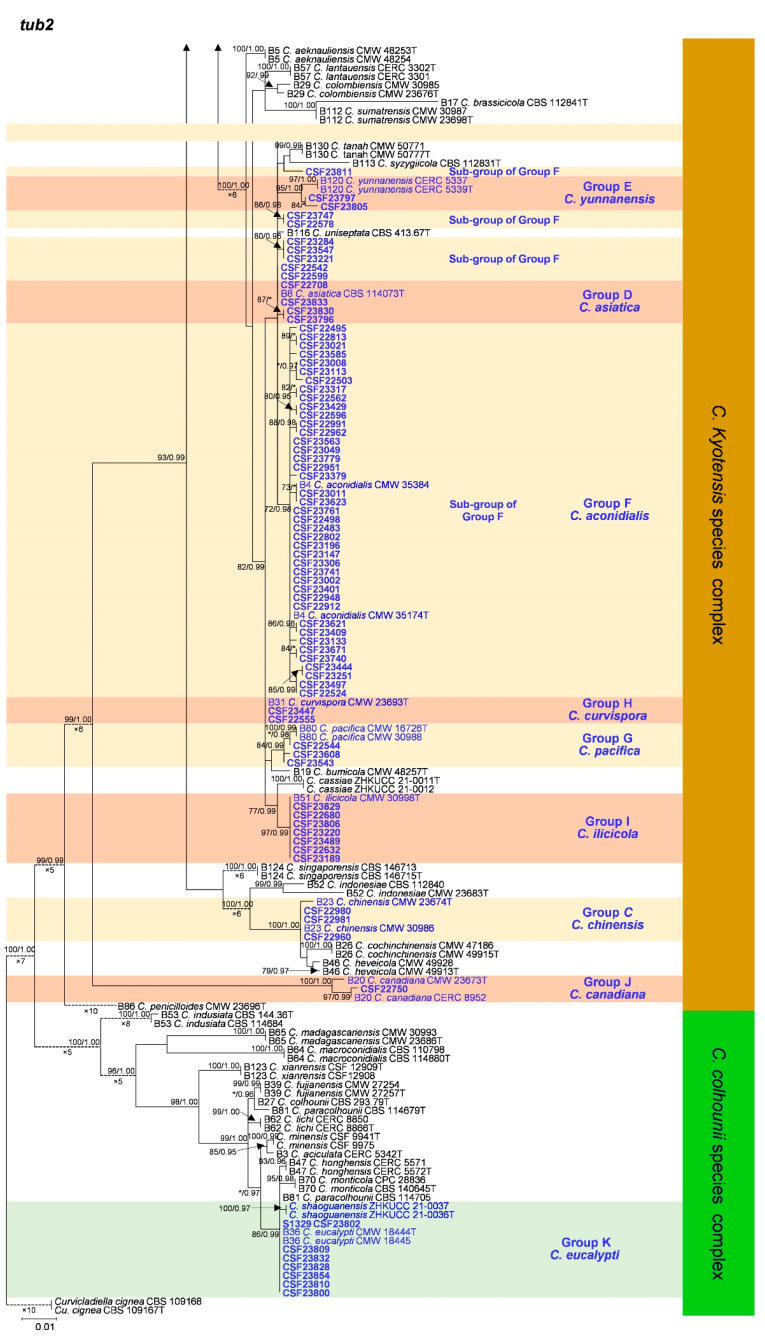
Phylogenetic tree of *Calonectria* species based on maximum likelihood (ML) analysis of the DNA dataset of the *tub2* gene sequences. Bootstrap support values ≥ 70% from ML analysis and posterior probabilities values ≥ 0.95 obtained from Bayesian inference (BI) are indicated at the nodes as ML/BI. Bootstrap values < 70% or posterior probabilities values < 0.95 are marked with “*”, and absent analysis values are marked with “-”. “*/*”, “*/-”, “-/*”, and “-/-” are not displayed. Isolates obtained in this study are highlighted in blue and bold. Ex-type isolates are indicated with “T”. The “B” species codes are consistent with the recently published results of Liu and co-authors [30]. *Curvicladiella cignea* (CBS 109167 and CBS 109168) was used as the outgroup taxon.

**Figure A3:**
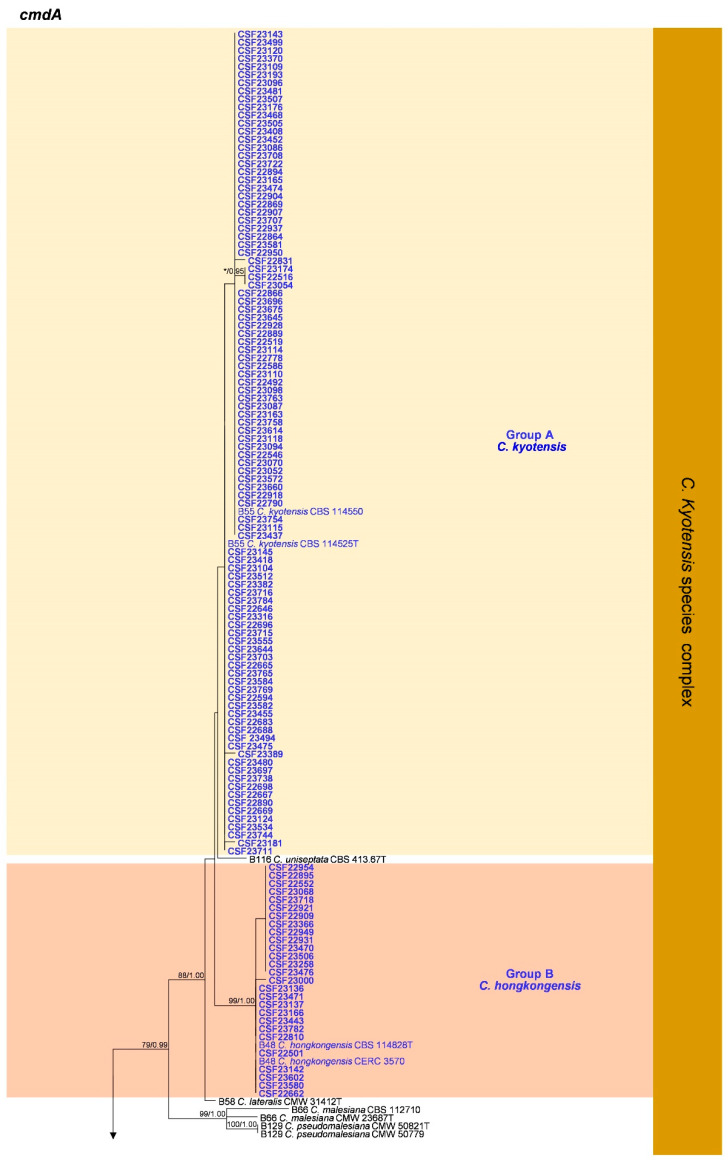

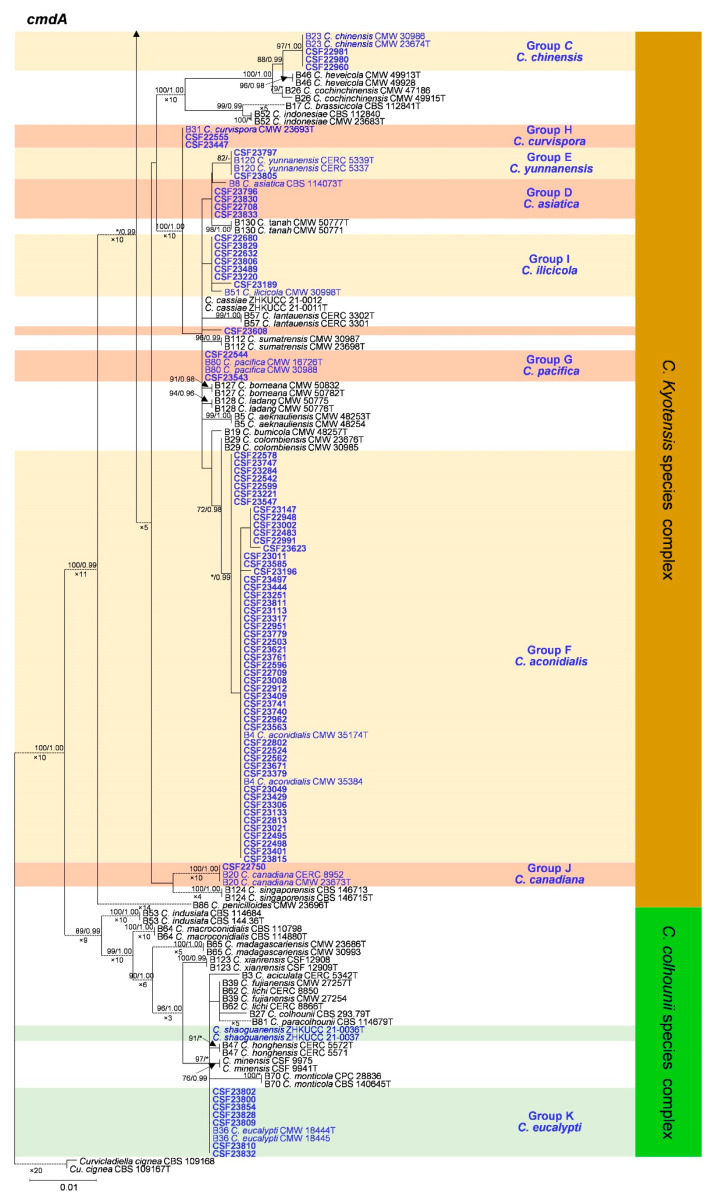
Phylogenetic tree of *Calonectria* species based on maximum likelihood (ML) analysis of the DNA dataset of the *cmdA* gene sequences. Bootstrap support values ≥ 70% from ML analysis and posterior probabilities values ≥ 0.95 obtained from Bayesian inference (BI) are indicated at the nodes as ML/BI. Bootstrap values < 70% or posterior probabilities values < 0.95 are marked with “*”, and absent analysis values are marked with “-”. “*/*”, “*/-”, “-/*”, and “-/-” are not displayed. Isolates obtained in this study are highlighted in blue and bold. Ex-type isolates are indicated with “T”. The “B” species codes are consistent with the recently published results of Liu and co-authors [30]. *Curvicladiella cignea* (CBS 109167 and CBS 109168) was used as the outgroup taxon.

**Figure A4:**
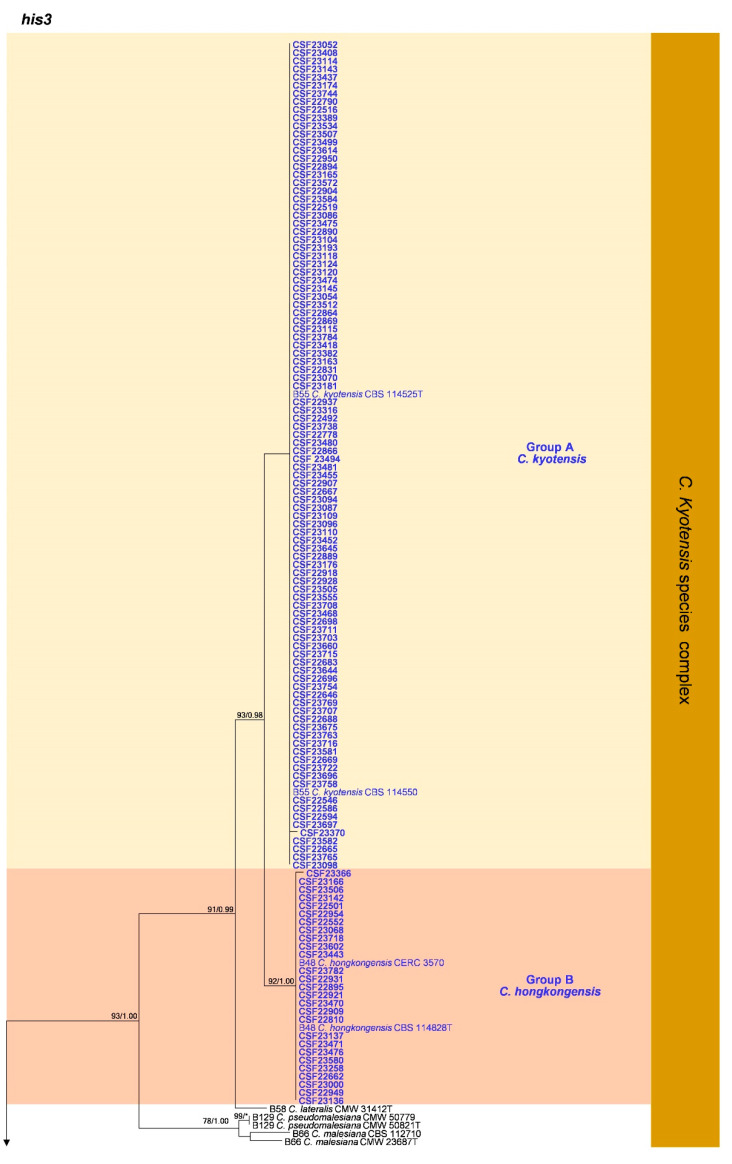

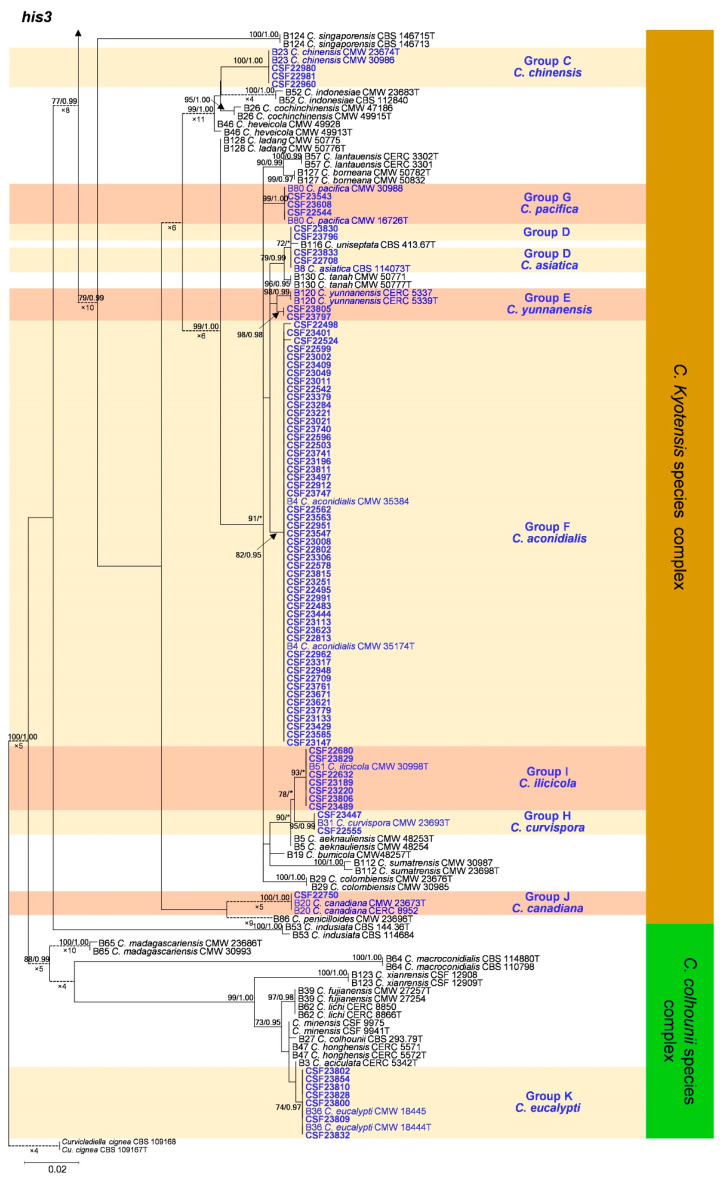
Phylogenetic tree of *Calonectria* species based on maximum likelihood (ML) analysis of the DNA dataset of the *his3* gene sequences. Bootstrap support values ≥ 70% from ML analysis and posterior probabilities values ≥ 0.95 obtained from Bayesian inference (BI) are indicated at the nodes as ML/BI. Bootstrap values < 70% or posterior probabilities values < 0.95 are marked with “*”, and absent analysis values are marked with “-”. “*/*”, “*/-”, “-/*”, and “-/-” are not displayed. Isolates obtained in this study are highlighted in blue and bold. Ex-type isolates are indicated with “T”. The “B” species codes are consistent with the recently published results of Liu and co-authors [30]. *Curvicladiella cignea* (CBS 109167 and CBS 109168) was used as the outgroup taxon.

**Figure A5:**
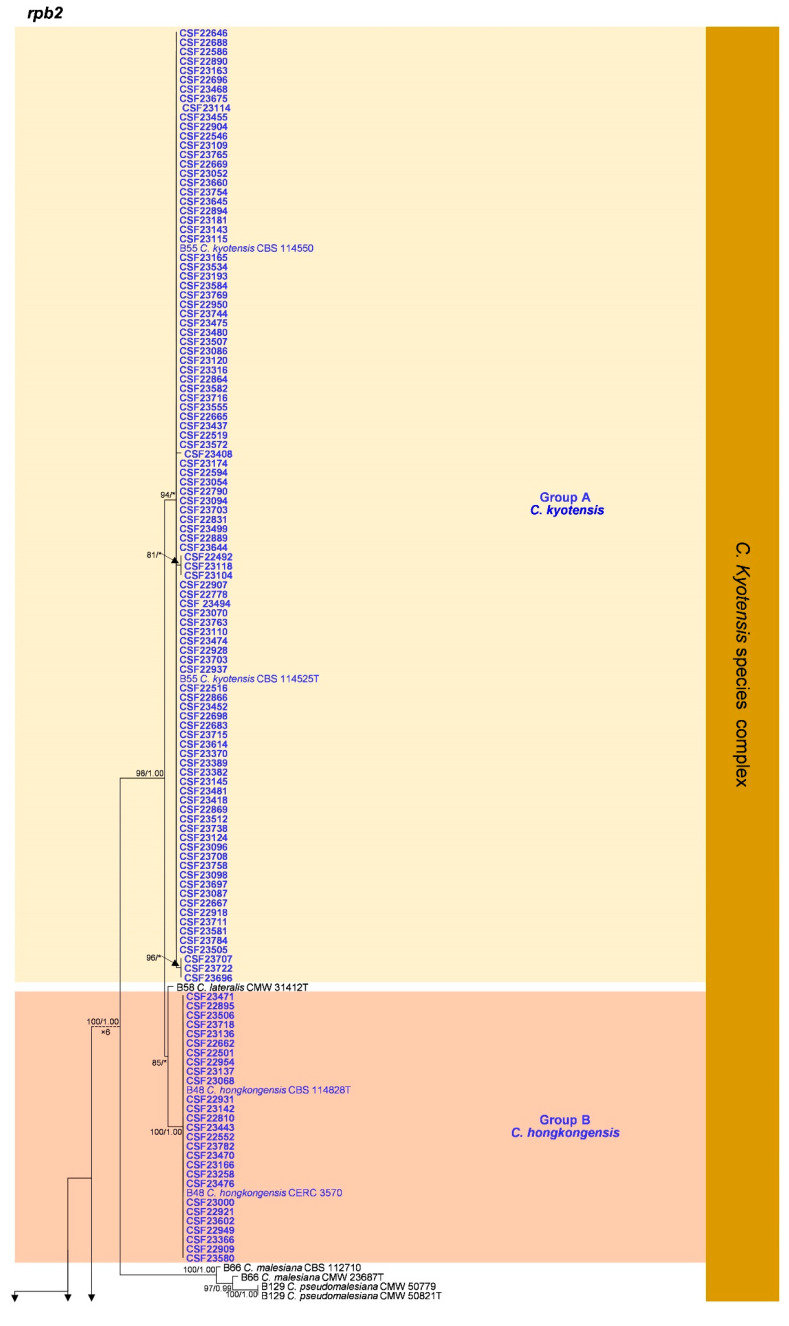

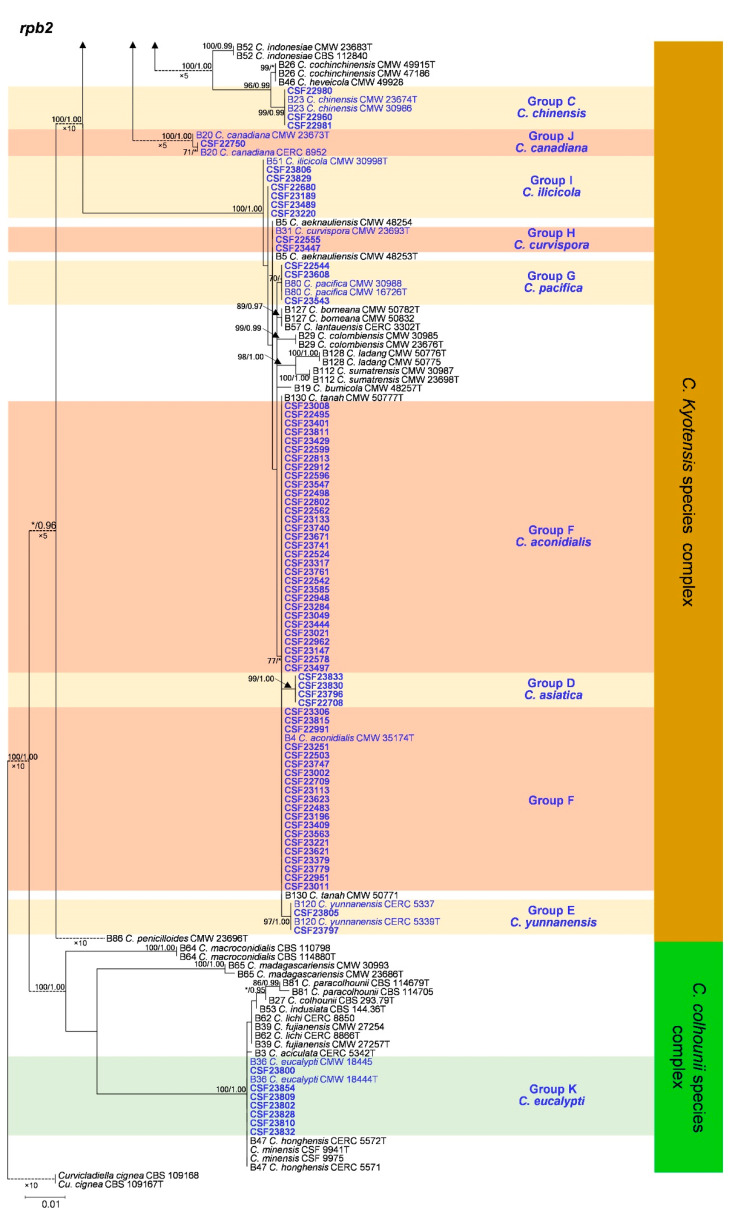
Phylogenetic tree of *Calonectria* species based on maximum likelihood (ML) analyses of the DNA dataset of the *rpb2* gene sequences. Bootstrap support values ≥ 70% from ML analysis and posterior probabilities values ≥ 0.95 obtained from Bayesian inference (BI) are indicated at the nodes as ML/BI. Bootstrap values < 70% or posterior probabilities values < 0.95 are marked with “*”, and absent analysis values are marked with “-”. “*/*”, “*/-”, “-/*”, and “-/-” are not displayed. Isolates obtained in this study are highlighted in blue and bold. Ex-type isolates are indicated with “T”. The “B” species codes are consistent with the recently published results of Liu and co-authors [30]. *Curvicladiella cignea* (CBS 109167 and CBS 109168) was used as the outgroup taxon.

**Figure A6:**
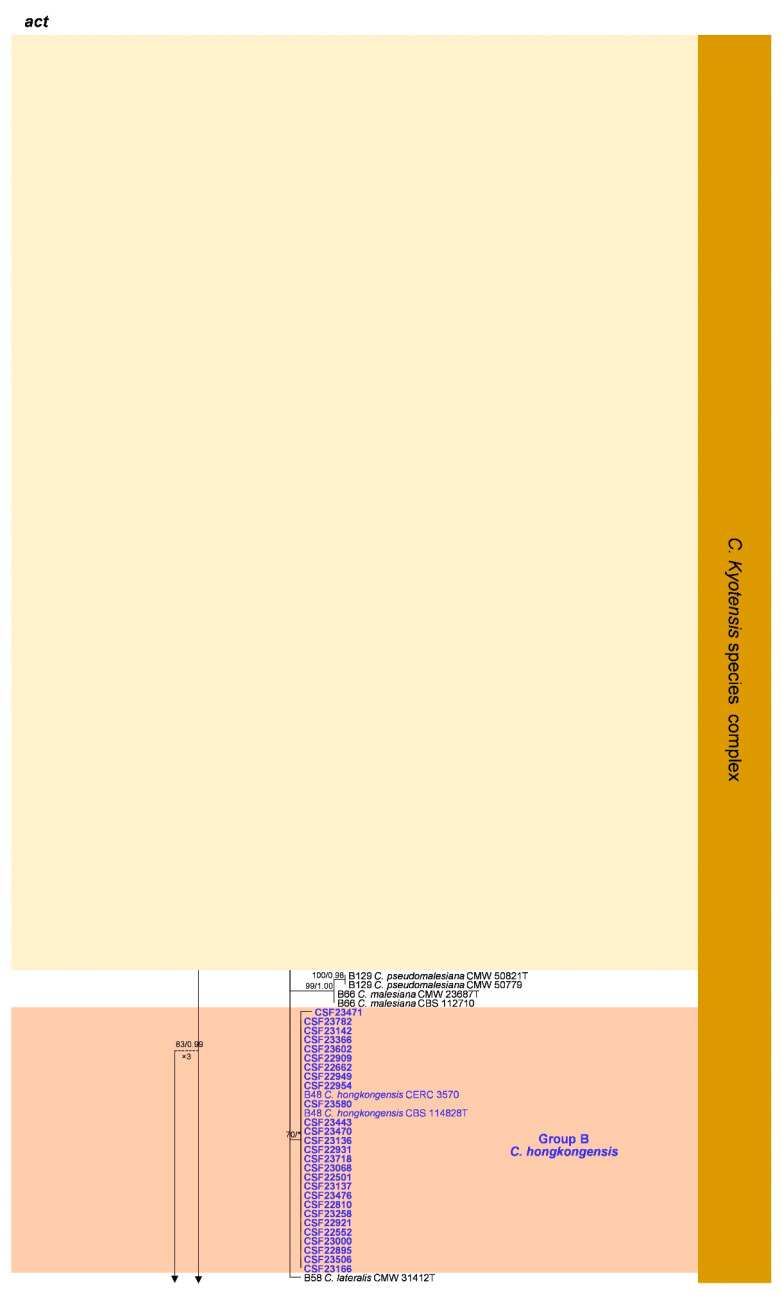

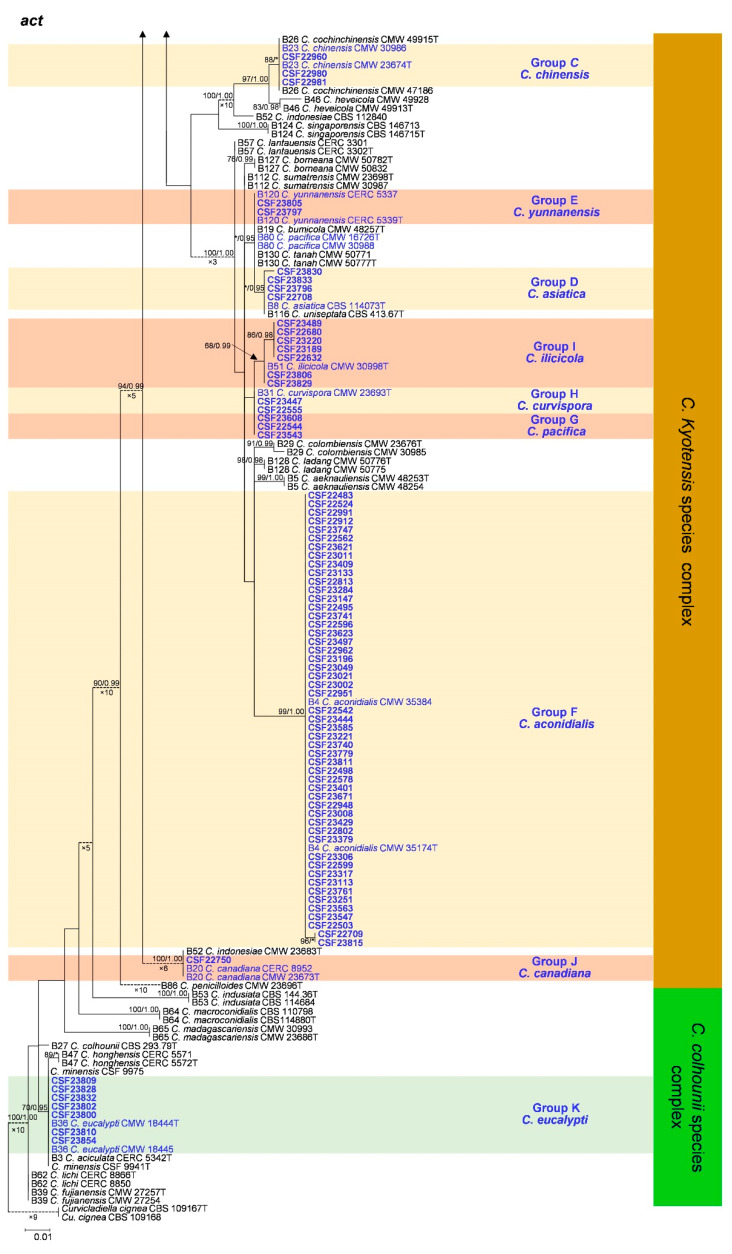
Phylogenetic tree of *Calonectria* species based on maximum likelihood (ML) analysis of the DNA dataset of the *act* gene sequences. Bootstrap support values ≥ 70% from ML analyses and posterior probabilities values ≥ 0.95 obtained from Bayesian inference (BI) are indicated at the nodes as ML/BI. Bootstrap values < 70% or posterior probabilities values < 0.95 are marked with “*”, and absent analysis values are marked with “-”. “*/*”, “*/-”, “-/*”, and “-/-” are not displayed. Isolates obtained in this study are highlighted in blue and bold. Ex-type isolates are indicated with “T”. The “B” species codes are consistent with the recently published results of Liu and co-authors [30]. *Curvicladiella cignea* (CBS 109167 and CBS 109168) was used as the outgroup taxon.

Phylogenetic analyses based on the six individual gene regions and the combination dataset for those six gene regions were conducted using both ML and BI methods. The overall topologies generated from the BI analyses were essentially similar to those from the ML analyses for each dataset. Consequently, only the ML tree with bootstrap support values of ML and posterior probabilities of BI was presented. The ML tree generated based on a combination of six gene sequences is presented in Figure 3, and the ML trees generated based on each of the six gene sequences were presented in Appendix F Figure A1, Figure A2, Figure A3, Figure A4, Figure A5 and Figure A6. Phylogenetic analyses showed that the 207 *Calonectria* isolates were clustered in 11 groups (Groups A–K) based on combined *tef1*/*tub2*/*cmdA*/*his3*/*rpb2*/*act* gene sequence analyses (Figure 3). The analyses showed that isolates in Groups A–J belong to the *C*. *kyotensis* species complex and that isolates in Group K belong to the *C*. *colhounii* species complex (Figure 3, Appendix F Figure A1, Figure A2, Figure A3, Figure A4, Figure A5 and Figure A6.

#### 3.3.1. Isolates in the *Calonectria kyotensis* Species Complex

Isolates in Groups A and B were clustered with *C*. *kyotensis* and *C*. *hongkongensis*, respectively, based on the *tef1*, *tub2*, *cmdA*, *his3*, *rpb2*, *act*, and combined *tef1*/*tub2*/*cmdA*/*his3*/*rpb2*/*act* trees (Figure 3, Appendix F Figure A1, Figure A2, Figure A3, Figure A4, Figure A5 and Figure A6). Therefore, isolates in Groups A and B were identified as *C*. *kyotensis* and *C*. *hongkongensis*, respectively.

Isolates in Group C were clustered with *C*. *chinensis* based on the *tef1*, *cmdA*, *his3*, and *rpb2* trees (Appendix F Figure A1, Figure A3, Figure A4 and Figure A5), closest to *C*. *chinensis* in the *tub2* tree (Appendix F Figure A2), and clustered with *C*. *chinensis* and *C*. *cochinchinensis* in the *act* tree (Appendix F Figure A6). These isolates were clustered with *C*. *chinensis* based on the combined *tef1*/*tub2*/*cmdA*/*his3*/*rpb2*/*act* tree (Figure 3). Isolates in Group C were identified as *C*. *chinensis*.

Isolates in Group D were clustered with *C*. *asiatica* in the *tef1* and *his3* trees (Appendix F Figure A1 and Figure A4), clustered with or closest to *C*. *asiatica* in the *tub2* tree (Appendix F Figure A2), closest to *C*. *asiatica* in the *cmdA* tree (Appendix F Figure A3), and clustered with or closest to *C*. *asiatica* and *C*. *uniseptate* in the *act* tree (Appendix F Figure A6). These isolates formed one independent clade in the *rpb2* tree (the *rpb2* sequence of the *C*. *asiatica* ex-type strain was not available) (Appendix F Figure A5). These isolates were clustered closest to *C*. *asiatica* based on the combined *tef1*/*tub2*/*cmdA*/*his3*/*rpb2*/*act* tree (Figure 3). Isolates in Group D were identified as *C*. *asiatica*.

Isolates in Group E were clustered with *C*. *yunnanensis* in the *tef1*, *tub2*, *cmdA*, *his3*, and *rpb2* trees (Appendix F Figure A1, Figure A2, Figure A3, Figure A4 and Figure A5) and clustered with *C*. *yunnanensis*, *C*. *bumicola*, *C*. *pacifica*, and *C*. *tanah* in the *act* tree (Appendix F Figure A6). These isolates were clustered with *C*. *yunnanensis* based on the combined *tef1*/*tub2*/*cmdA*/*his3*/*rpb2*/*act* tree (Figure 3). The isolates in Group E were identified as *C*. *yunnanensis*.

Isolates in Group F were clustered with *C*. *aconidialis* in *tef1*, *cmdA*, *his3*, and *act* trees (Appendix F Figure A1, Figure A3, Figure A4 and Figure A6). These isolates were clustered with or close to *C*. *aconidialis*, *C*. *asiatica*, and *C*. *uniseptate* in the *tub2* tree (Appendix F Figure A2) and clustered with *C*. *aconidialis* and *C*. *tanah* in the *rpb2* tree (Appendix F Figure A5). These isolates were clustered with *C*. *aconidialis* based on the combined *tef1*/*tub2*/*cmdA*/*his3*/*rpb2*/*act* tree (Figure 3). Isolates in Group F were identified as *C*. *aconidialis*.

Isolates in Group G were clustered with or close to *C*. *curvispora* and *C*. *pacifica* in the *tef1* tree (Appendix F Figure A1) and clustered with *C*. *pacifica* in the *tub2*, *his3*, and *rpb2* trees (Appendix F Figure A2, Figure A4 and Figure A5). These isolates were clustered with or close to *C*. *pacifica* and *C*. *cassia* in the *cmdA* tree (Appendix F Figure A3). These isolates were clustered with *C*. *curvispora* in the *act* tree (Appendix F Figure A6). The combined *tef1*/*tub2*/*cmdA*/*his3*/*rpb2*/*act* tree showed that these isolates clustered with *C*. *pacifica* (Figure 3). Isolates in Group G were identified as *C*. *pacifica*.

Isolates in Group H were clustered with *C*. *curvispora* in the *tef1*, *tub2*, *cmdA*, *his3*, and *act* trees (Appendix F Figure A1, Figure A2, Figure A3, Figure A4 and Figure A6) and clustered with *C*. *curvispora* and *C*. *aeknauliensis* in the *rpb2* tree (Appendix F Figure A5). These isolates were clustered with *C*. *curvispora* in the combined *tef1*/*tub2*/*cmdA*/*his3*/*rpb2*/*act* tree (Figure 3). Isolates in Group C were identified as *C*. *curvispora*.

Isolates in Group I were clustered with or close to *C*. *ilicicola and C*. *cassiae* in the *tef1* tree (Appendix F Figure A1). These isolates were clustered with *C*. *ilicicola* in the *tub2*, *cmdA*, *his3*, *rpb2*, *act*, and combined *tef1*/*tub2*/*cmdA*/*his3*/*rpb2*/*act* trees (Figure 3, Appendix F Figure A2, Figure A3, Figure A4, Figure A5 and Figure A6). Isolates in Group I were identified as *C*. *ilicicola*.

Isolates in Group J were clustered with *C*. *canadiana* in the *tef1*, *tub2*, *cmdA*, *his3*, and *rpb2* trees (Appendix F Figure A1, Figure A2, Figure A3, Figure A4 and Figure A5). These isolates were clustered with *C*. *canadiana* and *C*. *indonesiae* in the *act* tree (Appendix F Figure A6). These isolates were clustered with *C*. *canadiana* in the combined *tef1*/*tub2*/*cmdA*/*his3*/*rpb2*/*act* tree (Figure 3). Isolates in Group J were identified as *C*. *canadiana*.

#### 3.3.2. Isolates in the *Calonectria colhounii* Species Complex

Isolates in Group K were clustered with or close to *C*. *eucalypti*, *C*. *shaoguanensis*, *C*. *aciculata*, and *C*. *honghensis* in the *tef1* tree (Appendix F Figure A1), clustered with *C*. *eucalypti* and *C*. *paracolhounii* in the *tub2* tree (Appendix F Figure A2), clustered with *C*. *eucalypti* and *C*. *shaoguanensis* in the *cmdA* tree (Appendix F Figure A3), clustered with *C*. *eucalypti* in the *his3* tree (Appendix F Figure A4), clustered with *C*. *eucalypti*, *C*. *honghensis*, and *C*. *minesis* in the *rpb2* tree (Appendix F Figure A5), and clustered with *C*. *eucalypti*, *C*. *aciculata*, and *C*. *minesis* in the *act* tree (Appendix F Figure A6). The isolates were clustered with or close to *C*. *eucalypti*, *C*. *shaoguanensis*, and *C*. *honghensis* in the combined *tef1*/*tub2*/*cmdA*/*his3*/*rpb2*/*act* tree (Figure 3). The isolates in Group K were consistently clustered with or close to *C*. *eucalypti* in all analyses (Figure 3, Appendix F Figure A1, Figure A2, Figure A3, Figure A4, Figure A5 and Figure A6). Isolates in Group F were identified as *C*. *eucalypti*.

### 3.4. Taxonomy

Based on the results of multi-gene phylogenetic analyses and consideration of the morphological characteristics, *C*. *shaoguanensis* recently described in Zhang and co-authors [33] is reduced to synonymy with existing taxon as follows:

*Calonectria eucalypti* L. Lombard, M.J. Wingf. and Crous, Studies in Mycology 66: 31–69. 2010.

MycoBank MB 515530.

Synonym: *Calonectria shaoguanensis* Y. X. Zhang et al., Journal of Fungi 8: 719. 2022.

Index Fungorum number: IF 555217.

In: *Calonectria colhounii* species complex.

Typus: PREM 60298 holotype.

Ex-type culture: CBS 125275 = CMW 18444.

Type locality: Indonesia, Sumatra Utara, Aek Nauli.

Type substrate: *Eucalyptus grandis*.

Barcodes: *act* = MT335013; *cmdA* = MT335243; *his3* = MT335483; *rpb2* = MT412545; *tef1* = MT412774; *tub2* = MT412992 (alternative markers: ITS = MT359704; LSU = MT359464).

Notes: *Calonectria shaoguanensis* was identified as a new species based on DNA sequence comparisons of the *tef1*, *tub2*, and *cmdA* gene regions and the morphological characteristics in Zhang and co-authors [33]. *Calonectria shaoguanensis* was treated as a synonym with *C*. *eucalypti* in this study. In comparison of DNA sequences for the *tef1*, *tub2*, and *cmdA* gene regions, there was only one base difference between the ex-type isolate of *C*. *shaoguanensis* (ZHKUCC 21-0036) and the ex-type isolate of *C*. *eucalypti* (CMW 18444 = CBS 125275) in the *tub2* sequences. Both of the species produce clavate vesicles with overlapping dimensions (C. *shaoguanensis*: 2–7 μm [33]; C. *eucalypti*: 4–6 μm [44]). The macroconidia of C. *shaoguanensis* (av. 65 × 6.5 μm) are shorter than those of *C*. *eucalypti* (av. 72 × 6 μm) [33,44], which were considered to represent intraspecific variation justifying this synonymy.

### 3.5. Calonectria Richness in Soils from Four Provinces and Plantations of Three Tree Species

**Figure 4:**
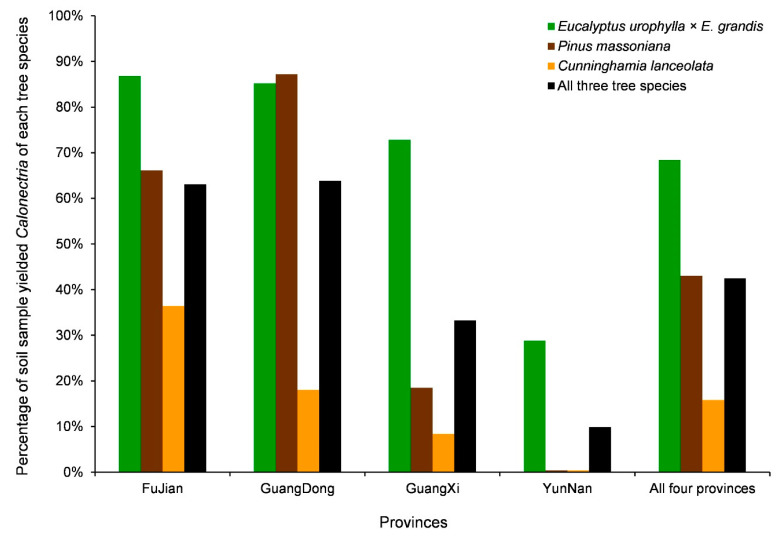
Percentage of soil samples that yielded *Calonectria* in plantations of three tree species in four regions (provinces).

A total of 1270 isolates of *Calonectria* were obtained from 2991 soil samples collected from 12 sampling sites of three plantations in four provinces (Table 5, Figure 4). *Calonectria* isolates were obtained from 42.5% of the soil samples (Table 5, Figure 4). When considering the 12 sampling sites, 0.4 to 87.2% of the soil samples yielded *Calonectria* (Figure 4); the highest percentage of soil samples that yielded *Calonectria* was *P*. *massoniana* in GuangDong (87.2%), followed by *E*. *urophylla* × *E*. *grandis* from FuJian (86.8%) and *E*. *urophylla* × *E*. *grandis* from GuangDong (85.2%); the lowest percentages of soil samples that yielded *Calonectria* were from *P*. *massoniana* (0.4%) and *C*. *lanceolata* (0.4%) in YunNan (Table 5, Figure 4).

When considering the four sampled geographic regions, the percentage of soil samples that yielded *Calonectria* decreased from regions in the east to the west (Figure 2 and Figure 4); a higher percentage of soil samples that yielded *Calonectria* was obtained in GuangDong (63.8%) and FuJian (63.1%), with less in GuangXi (33.2%), and the lowest percentage of soil samples that yielded *Calonectria* was in YunNan (9.9%) (Table 5, Figure 4). When considering the three tree species, the highest percentage of soil samples that yielded *Calonectria* were from *E*. *urophylla* × *E*. *grandis* plantations (68.4%), followed by *P*. *massoniana* plantations (43%) and *C*. *lanceolata* (15.8%) (Table 5, Figure 4).

### 3.6. Calonectria Species Diversity in Four Provinces and Plantations of Three Tree Species

**Figure 5:**
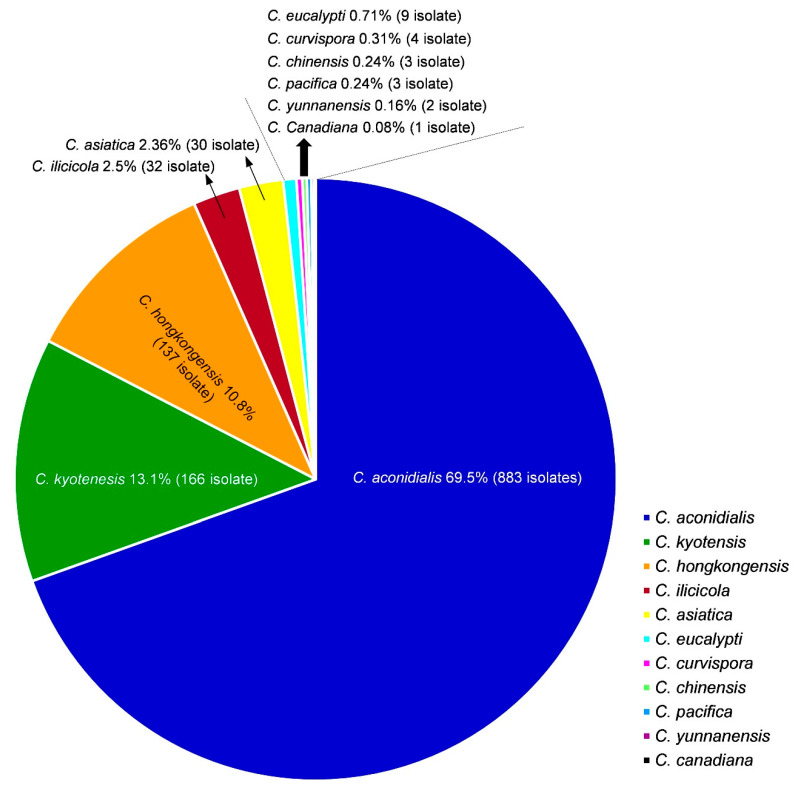
Percentage of each *Calonectria* species obtained from all sampling sites in this study. Different *Calonectria* species are indicated by numbers with different colors.

Based on the sequence comparisons of *act*, *cmdA*, *his3*, *rpb2*, *tef1*, and *tub2* sequences, the 1270 *Calonectria* isolates were identified as 11 species. These species were *C*. *aconidialis* (883 isolates; 69.50%), *C*. *kyotensis* (166 isolates; 13.10%), *C*. *hongkongensis* (137 isolates; 10.80%), *C*. *ilicicola* (32 isolates; 2.50%), *C*. *asiatica* (30 isolates; 2.36%), *C*. *eucalypti* (9 isolates; 0.71%), *C*. *curvispora* (4 isolates; 0.31%), *C*. *chinensis* (3 isolates; 0.24%), *C*. *pacifica* (3 isolates; 0.24%), *C*. *yunnanensis* (2 isolates; 0.16%), and *C*. *canadiana* (1 isolate; 0.08%) (Table 6, Figure 5). *Calonectria aconidialis* was most dominant, followed by *C*. *kyotensis* and *C*. *hongkongensis*. Three species accounted for 93.4% of all *Calonectria* isolates obtained in this study (Figure 5). These three species were regarded as the dominant species (Table 6, Figure 5). A relatively small number of isolates were obtained for *C*. *ilicicola* and *C*. *asiatica*. Less than 10 isolates were obtained for each of the remaining six species (Table 6, Figure 5).

When considering the 12 sampling sites, each of *C*. *aconidialis*, *C*. *kyotensis, C*. *hongkongensis* and *C*. *ilicicola* was isolated from more than half of all the 12 sampling sites. *Calonectria aconidialis* and *C*. *ilicicola* were distributed at all sampling sites in four provinces, with the exception of *P*. *massoniana* and *C*. *lanceolata* plantations in YunNan. *Calonectria kyotensis* was distributed at all sampling sites in FuJian, GuangDong, and GuangXi Provinces, with the exception of *C*. *lanceolata* plantations in FuJian and GuangXi. *Calonectria hongkongensis* was distributed at all sampling sites in FuJian, GuangDong, and GuangXi Provinces, with the exception of *C*. *lanceolata* plantations in GuangDong and GuangXi. The remaining seven species were isolated only from the soils of one or two tree species plantations in a single province (Table 6, Figure 2b–m).

**Figure 6:**
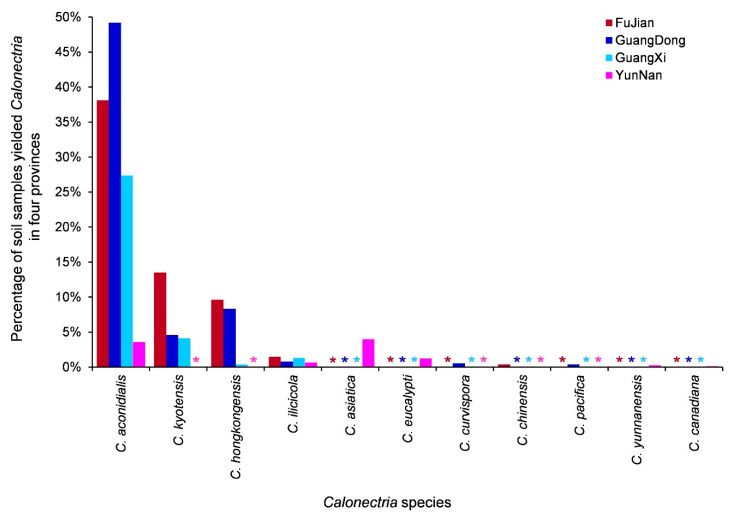
The percentage of soil samples that yielded each of the 12 *Calonectria* species in the four provinces. “*” means zero.

When considering the four sampled geographic regions, five, six, four, and six *Calonectria* species were isolated from soil samples in FuJian, GuangDong, GuangXi, and YunNan, respectively (Table 6). *Calonectria aconidialis* and *C*. *ilicicola* were found in all four provinces. *Calonectria kyotensis* and *C*. *hongkongensis* were found in three provinces, excluding YunNan. Each of the remaining seven species was found in only one province (Table 6, Figure 2 and Figure 6). For *C*. *aconidialis*, the percentage of soil samples that yielded *Calonectria* decreased from the eastern to the western provinces, with the exception of GuangDong Province (Table 6, Figure 6). For each species of *C*. *kyotensis* and *C*. *hongkongensis*, the percentage of soil samples that yielded *Calonectria* decreased from regions in the eastern to the western provinces (Table 6, Figure 6). The percentages of soil samples containing *C*. *ilicicola* in regions in the eastern and western provinces were similar (Table 6, Figure 6).

**Figure 7:**
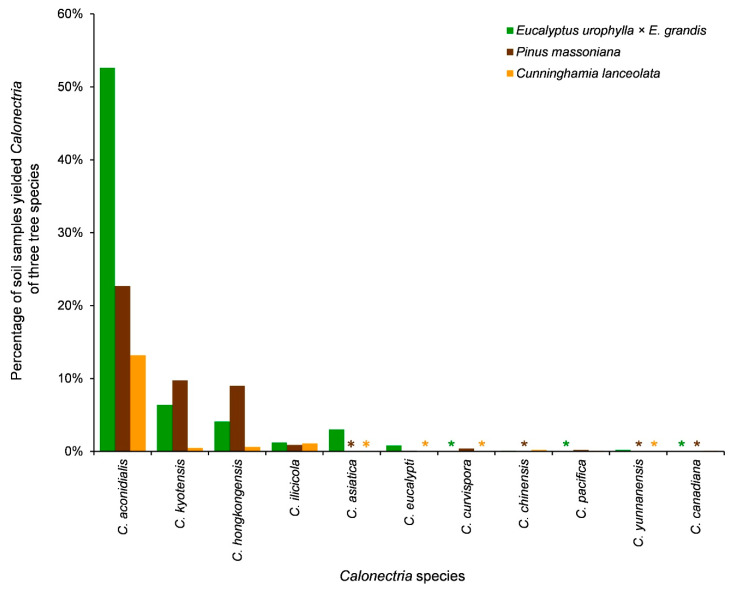
The percentage of soil samples that yielded each of the 12 *Calonectria* species from the plantations of the three tree species. “*” means zero.

When considering the plantation tree species, eight, seven, and seven species were identified in *E*. *urophylla* × *E*. *grandis*, *P*. *massoniana*, and *C*. *lanceolata* plantations, respectively (Table 6). *Calonectria aconidialis*, *C*. *kyotensis*, *C*. *hongkongensis*, and *C*. *ilicicola* were isolated from soils in all three tree species. Each of the remaining seven species was isolated only from soils with one or two tree species (Table 6, Figure 2 and Figure 7). For *C*. *aconidialis*, the highest percentage of soil samples that yielded *Calonectria* was in *E*. *urophylla* × *E*. *grandis* plantations, followed by *P*. *massoniana* plantations and *C*. *lanceolata* plantations (Table 6, Figure 7). For each species of *C*. *kyotensis* and *C*. *hongkongensis*, the percentage of soil samples that yielded *Calonectria* was highest in *P*. *massoniana* plantations, followed by *E*. *urophylla* × *E*. *grandis* plantations and *C*. *lanceolata* plantations (Table 6, Figure 7). For *C*. *ilicicola*, the percentage of soil samples that yielded *Calonectria* was similar among the plantations of three tree species (Table 6, Figure 7).

### 3.7. Genotyping of Isolates within each Calonectria Species

The genotypes of 1270 *Calonectria* isolates obtained in this study were determined by *tef1* and *tub2* sequences. There were 28, 41, 10, 3, 2, 4, 1, 2, 3, 2, and 1 genotype(s) of *C*. *aconidialis*, *C*. *kyotensis*, *C*. *hongkongensis*, *C*. *ilicicola*, *C*. *asiatica*, *C*. *eucalypti*, *C*. *curvispora*, *C*. *chinensis*, *C*. *pacifica*, *C*. *yunnanensis*, and *C*. *canadiana*, respectively (Table 2). The three dominant *Calonectria* species, *C*. *aconidialis*, *C*. *kyotensis*, and *C*. *hongkongensis*, had more genotypes than the other species (Table 2). The ratio of genotype number to isolate number of *C*. *kyotensis* was highest within the three dominant species (Table 2).

The *tef1*-*tub2* genotypes of each *Calonectria* species in each of the 12 sampling sites are listed in Appendix B Table A2 and Table 7. For the three dominant species, *C*. *aconidialis*, *C*. *kyotensis*, and *C*. *hongkongensis*, the overall data showed that the number of genotypes of each *Calonectria* species at each sampling site positively correlated with the number of isolates (Table 6, Table 7 and Appendix B Table A2). For each species of *C*. *aconidialis* and *C*. *hongkongensis*, the dominant genotype (genotype AA) existed in most of the sampling sites (Appendix B Table A2). For example, the dominant genotype AA accounted for 61.7 to 100% of the *C*. *aconidialis* isolates obtained from sampling sites 1–9 (Appendix B Table A2). There was no dominant genotype for *C*. *kyotensis* from the seven sampling sites that had *Calonectria* (Appendix B Table A2).

### 3.8. Genotype Diversity of Calonectria Species in Four Provinces and Plantations of Three Tree Species

*Calonectria aconidialis*, *C*. *kyotensis,* and *C*. *hongkongensis* were the dominant species in this study. The statistical results of the number of shared genotypes of *C*. *aconidialis* isolates indicated that the ratio of shared genotypes among the sites of “the same region but different plantation tree species” (30 shared genotypes/12 pairs of comparison sampling sites = 2.5) was much bigger than that of the sites of “different geographical region but the same plantation tree species” (12 shared genotypes/18 pairs of comparison sampling sites = 0.67) and also bigger than that of the sites of “different geographical region and different plantation tree species” (24 shared genotypes/36 pairs of comparison sampling sites = 0.67) (Appendix C Table A3). The statistical results of the number of shared genotypes of *C*. *kyotensis* isolates indicated that the ratio of shared genotypes among the sites of “the same region but different plantation tree species” (19 shared genotypes/12 pairs of comparison sampling sites = 1.58) was much bigger than that of the sites of “different geographical region but the same plantation tree species” (12 shared genotypes/18 pairs of comparison sampling sites = 0.67) and also bigger than that of the sites of “different geographical region and different plantation tree species” (15 shared genotypes/36 pairs of comparison sampling sites = 0.42) (Appendix D Table A4). The statistical results of the number of shared genotypes of *C*. *hongkongensis* isolates indicated that the ratio of shared genotypes among the sites of “the same region but different plantation tree species” (13 shared genotypes/12 pairs of comparison sampling sites = 1.08) was much bigger than that of the sites of “different geographical region but the same plantation tree species” (11 shared genotypes/18 pairs of comparison sampling sites = 0.61) and also bigger than that of the sites of “different geographical region and different plantation tree species” (17 shared genotypes/36 pairs of comparison sampling sites = 0.47) (Appendix E Table A5). These results suggest that the genetic variations of each species of *C*. *aconidialis*, *C*. *kyotensis*, and *C*. *hongkongensis* are likely to be more affected by geographical region than plantation tree species.

## 4. Discussion

In this study, a relatively large number of soil samples were collected from 12 plantations of *E*. *urophylla* × *E*. *grandis*, *P*. *massoniana*, and *C*. *lanceolate* in FuJian, GuangDong, GuangXi, and YunNan Provinces in southern China. A total of 1270 *Calonectria* isolates were obtained. Based on multi-gene sequence phylogenetic analyses, these isolates were identified as 11 *Calonectria* species. Except for *C*. *eucalypti*, which resides in the *C*. *colhounii* species complex, the remaining 10 species belong to the *C*. *kyotensis* species complex. The most dominant species was *C*. *aconidialis*, followed by *C*. *kyotensis* and *C*. *hongkongensis*.

The richness of *Calonectria* in soils (percentage of soil samples that yielded *Calonectria*) among the four geographical regions, as well as among the three tree species, differed. *Calonectria* richness in the eastern regions was higher than that in the western regions. A possible reason for this phenomenon is that the annual rainfall in the eastern regions was greater than in the western regions, where the soil in plantations in the eastern regions was under continuous high humidity [59,60]. Previous research results have shown that *Calonectria* species are more likely to exist in soils with consistently high levels of moisture [61]. The richness of *Calonectria* in soils of *E*. *urophylla* × *E*. *grandis*, *P*. *massoniana*, and *C*. *lanceolata* plantations decreased gradually. The richness of *Calonectria* in soils is probably affected by the litter of different tree species [62,63,64].

This study indicated that *Calonectria* species are widely distributed in soils of *E*. *urophylla* × *E*. *grandis*, *P*. *massoniana*, and *C*. *lanceolata* plantations. Previous research results have shown that *Calonectria* species, especially those in the *C*. *kyotensis* species complex, are widely distributed in the soils of *Eucalyptus* plantations in southern China [19,31]. Recent research results have indicated that *Calonectria* is also frequently isolated from soils in plantations of multiple tree species [32]. We suppose that *Calonectria* species are widely distributed in forest soils in southern China.

The distribution characteristics of the 11 *Calonectria* species at 12 sampling sites from 12 plantations of three tree species in four provinces differed. The three dominant species, *C*. *aconidialis*, *C*. *kyotensis*, and *C*. *hongkongensis*, as well as *C*. *ilicicola*, were distributed much more widely than the remaining seven species. This is consistent with recent research results [31,32]. Both *C*. *aconidialis* and *C*. *ilicicola* were isolated from 10 of the 12 sampling sites, while the richness of *C*. *aconidialis* at these sites was much higher than that of *C*. *ilicicola*. These results highlight the distribution differences in *Calonectria* species in soils. This study resulted in the first report of *C*. *curvispora* in China, and it was isolated only from soils in *P*. *massoniana* plantations in GuangDong Province. Our results suggest that *C*. *curvispora* may not be widely distributed in plantation soil in southern China.

The distribution of the three dominant species, *C*. *aconidialis*, *C*. *kyotensis*, and *C*. *hongkongensis*, was affected by geographic regions and plantation tree species, although their distribution patterns were not the same. The richness of these three species was generally higher in eastern regions than in western regions. However, the influencing characteristics of species richness, affected by plantation tree species, were not the same. Species richness was highest in *E*. *urophylla* × *E*. *grandis* plantations for *C*. *aconidialis*, while richness was highest in *P*. *massoniana* plantations for both *C*. *kyotensis* and *C*. *hongkongensis*. Species richness was lowest for these three species in the *C*. *lanceolate* plantations. Our research results suggest that the distribution patterns differ among *Calonectria* species associated with soils in angiosperm and gymnosperm plants [32].

*Calonectria aconidialis* is the most dominant species obtained from forest soils in this study. Since this species was first isolated and described from soils in *Eucalyptus* plantation in HaiNan Province in southern China [18], it has been frequently isolated from soils in *Eucalyptus* plantations in GuangXi, GuangDong and FuJian Provinces [15,19,31,32]. Besides *Eucalyptus*, *C. aconidialis* was also obtained from soils in *C. lanceolata*, *Phyllostachys heterocycle* and natural forests [32]. We speculate that *C. aconidialis* is widely distributed in soils in forests of multiple tree species in southern China and neighboring countries.

The distribution characteristics of *Calonectria* in YunNan differed from those in FuJian, GuangDong, and GuangXi. The percentages of soil samples that yielded *Calonectria* in plantations of *E*. *urophylla* × *E*. *grandis*, *P*. *massoniana*, and *C*. *lanceolata* in YunNan were significantly lower than those in the other three provinces. A possible reason is that the climate in YunNan is relatively drier than that of the other three provinces [65]. Among the 11 species identified in this study, *C*. *asiatica*, *C*. *yunnanensis*, *C*. *eucalypti*, and *C*. *canadiana* were isolated only from YunNan Province. Based on several previous studies conducted on *Calonectria* in China, *C*. *asiatica* and *C*. *yunnanensis* have been collected only from soils in *Eucalyptus* plantations in YunNan [10,15,19,29,30,31,32]. *Calonectria eucalypti* has been isolated only from the leaves of *Eucalyptus* plantations in the FuJian and YunNan Provinces [15,16]. This study reported the first record of *C*. *eucalypti* isolated from soils. In China, *C*. *canadiana* has only been isolated from soil in northern regions, including HeNan, HeiLongJiang, and HeBei Provinces [28,66,67]. *Calonectria canadiana* is considered a temperate climate-distributed species. In this study, it was isolated from YunNan Province in southern China. For the region in YunNan Province where *C*. *canadiana* was obtained, the climate was similar to these regions in northern China, since the region in YunNan in this study is located in the Yunnan-Guizhou Plateau, and the average annual temperature in this region is relatively low. A possible reason for the differences in *Calonectria* richness and species diversity between YunNan and the other provinces is the special climate in YunNan compared with the other three provinces [68].

The distribution patterns of *Calonectria* fungi in forest soils in different continents and countries are not consistent. In South America, the majority of *Calonectria* fungi isolated from forest soils resided in the *C. brassicae* and *C. candelabrum* species complexes [30,41]. In Asia, *Calonectria* species in the *C. kyotensis*, *C. reteaudii*, *C. colhounii*, *C. cylindrospora* and *C. brassicae* species complexes were isolated from forest soils in China and southeastern Asian countries; most of these obtained species resided in the *C. kyotensis* species complex [9,15,19,31,32,42]. In this study, ten of eleven obtained *Calonectria* species resided in *C. kyotensis* species complex. All the three, eight of eleven, and six of eight *Calonectria* species isolated from soils resided in *C. kyotensis* species complex in Indonesia, Vietnam and Malaysia, respectively [9,42]. The dominant species in the *C. kyotensis* species complex among different Asian countries were not the same. For example, *C. aconidialis* is the most dominant species isolated from forest soils in China, while this species has never been isolated from other Asian countries [9,15,19,30,31,32,42].

This study explored the richness, species diversity, and distribution characteristics of *Calonectria* from soils in the plantations of three tree species in four provinces in southern China. Our research results indicate that *Calonectria* richness is affected by geographic regions and plantation tree species. For the dominant species, their distribution patterns affected by geographic regions and plantation tree species are not the same, and their genetic variations may be more greatly affected by geographic region than by plantation tree species. For the dominant species, additional studies need to be conducted to clarify the genetic diversity and population differences among isolates from soils in different geographic regions and plantations of different tree species, which will help us to understand the influencing characteristics of geographic regions and plantation tree species on their genetic variations.

## Figures and Tables

**Table 1:** Plantation tree species, location details, and collection information of soil samples collected from plantations of three tree species in four provinces.

Site Number	Region Code	Province	Tree Species	Years of Trees Planted	Number of Soil Samples	Location Details	GPS Information	Collector	Collecting Date
1	Region A	FuJian	*Eucalyptus urophylla* × *E. grandis*	>10	250	ShaJian Town, HuaAn County, ZhangZhou Region, FuJian Province	Near site 24°46′2.6364″ N, 117°37′0.264″ E	S.F. Chen, Y. Liu, J.L. Han and L.L. Liu	26–27 May 2021
2	Region A	FuJian	*Pinus massoniana*	15	248	ShaJian Town, HuaAn County, ZhangZhou Region, FuJian Province	Near site 24°46′35.2524″ N, 117°36′2.8368″ E	S.F. Chen, Y. Liu, J.L. Han and L.L. Liu	24 May 2021
3	Region A	FuJian	*Cunninghamia lanceolata*	12	250	ShaJian Town, HuaAn County, ZhangZhou Region, FuJian Province	Near site 24°46′33.6936″ N, 117°37′5.4876″ E	S.F. Chen, Y. Liu, J.L. Han and L.L. Liu	25 May 2021
4	Region B	GuangDong	*E. urophylla* × *E. grandis*	>10	250	HeerKou Town, FengKai County, ZhaoQing Region, GuangDong Province	Near site 23°30′11.3688″ N, 111°50′43.5156″ E	S.F. Chen, Y. Liu, J.L. Han and W.X. Wu	7 June 2021
5	Region B	GuangDong	*P. massoniana*	15	250	HeerKou Town, FengKai County, ZhaoQing Region, GuangDong Province	Near site 23°30′8.5716″ N, 111°50′56.9616″ E	S.F. Chen, Y. Liu, J.L. Han and W.X. Wu	6 June 2021
6	Region B	GuangDong	*C. lanceolata*	10	244	HeerKou Town, FengKai County, ZhaoQing Region, GuangDong Province	Near site 23°27′48.6864″ N, 115°55′46.7472″ E	S.F. Chen, Y. Liu, J.L. Han and W.X. Wu	9 June 2021
7	Region C	GuangXi	*E. urophylla* × *E. grandis*	>10	250	XiaAo Town, DuAn County, HeChi Region, GuangXi Autonomous Region	Near site 24°19′20.1″ N, 107°56′29.3″ E	S.F. Chen	21–24 June 2021
8	Region C	GuangXi	*P. massoniana*	20	249	XiaAo Town, DuAn County, HeChi Region, GuangXi Autonomous Region	Near site 24°19′24.3″ N, 107°56′20″ E	S.F. Chen	21–22 June 2021
9	Region C	GuangXi	*C. lanceolata*	12	250	XiaAo Town, DuAn County, HeChi Region, GuangXi Autonomous Region	Near site 24°19′13″ N, 107°56′18″ E	S.F. Chen	21–23 June 2021
10	Region D	YunNan	*E. urophylla* × *E. grandis*	>11	250	YongPing Town, JingGu County, PuEr Region, YunNan Province	Near site 23°25′40.026″ N, 100°18′24.678″ E	S.F. Chen, Y. Liu, X.Y. Liang, L.Q. Lu and B.Y. Chen	8 July 2021
11	Region D	YunNan	*P. massoniana*	15	250	YongPing Town, JingGu County, PuEr Region, YunNan Province	Near site 23°24′54.666″ N, 100°17′45.0384″ E	S.F. Chen, Y. Liu, X.Y. Liang, L.Q. Lu and B.Y. Chen	9 July 2021
12	Region D	YunNan	*C. lanceolata*	15	250	YongPing Town, JingGu County, PuEr Region, YunNan Province	Near site 23°22′58.1916″ N, 100°9′5.436″ E	S.F. Chen, Y. Liu, X.Y. Liang, L.Q. Lu and B.Y. Chen	7 July 2021

**Table 2:** Isolate numbers of each genotype of each *Calonectria* species.

*Calonectria* Species	Genotype Determined by *tef1* Gene Sequences ^a^	Number of Isolates Based on *tef1* Genotype	Genotype Determined by *tub2* Gene Sequences ^a^	Number of Isolates Based on *tub2* Genotype	Genotype Determined by *tef1* and *tub2* Gene Sequences ^a^	Number of Isolates Based on *tef1* and *tub2* Genotype	Number of Genotype Determined by *tef1* and *tub2* Gene Sequences of Each Species
*C. aconidialis*	A	832	A	689	AA	641	28
	B	1	B	3	AB	3	
	C	36	C	3	AC	3	
	D	1	D	5	AD	5	
	E	3	E	1	AE	1	
	F	1	F	8	AF	8	
	G	9	G	1	AG	1	
			H	2	AH	2	
			I	29	AI	29	
			J	1	AJ	1	
			K	26	AK	26	
			L	10	AL	10	
			M	5	AM	5	
			N	1	AN	1	
			O	4	AO	1	
			P	1	AP	1	
			Q	1	AQ	1	
			R	5	AR	5	
			S	2	AS	2	
			T	56	AT	56	
			U	30	AU	30	
					BA	1	
					CA	36	
					DA	1	
					EA	3	
					FA	1	
					GA	6	
					GO	3	
*C. kyotensis*	A	46	A	21	AA	5	41
	B	1	B	1	AD	4	
	C	39	C	1	AF	9	
	D	77	D	10	AI	13	
	E	3	E	3	AK	2	
			F	26	AL	4	
			G	1	AO	6	
			H	1	AP	1	
			I	27	AR	2	
			J	1	BR	1	
			K	12	CA	6	
			L	10	CB	1	
			M	11	CE	2	
			N	2	CF	4	
			O	22	CH	1	
			P	1	CI	1	
			Q	1	CM	4	
			R	9	CN	2	
			S	2	CO	9	
			T	1	CR	4	
			U	2	CS	2	
			V	1	CT	1	
					CU	2	
					DA	9	
					DC	1	
					DD	6	
					DE	1	
					DF	13	
					DG	1	
					DI	13	
					DJ	1	
					DK	10	
					DL	5	
					DM	7	
					DO	6	
					DQ	1	
					DR	2	
					DV	1	
					EA	1	
					EL	1	
					EO	1	
*C. hongkongensis*	A	136	A	92	AA	92	10
	B	1	B	8	AB	8	
			C	3	AC	3	
			D	6	AD	6	
			E	3	AE	3	
			F	19	AF	18	
			G	3	AG	3	
			H	2	AH	2	
			I	1	AI	1	
					BF	1	
*C. ilicicola*	A	24	A	32	AA	24	3
	B	3			BA	3	
	C	5			CA	5	
*C. asiatica*	A	30	A	21	AA	21	2
			B	9	AB	9	
*C. eucalypti*	A	4	A	9	AA	4	4
	B	2			BA	2	
	C	1			CA	1	
	D	2			DA	2	
*C. curvispora*	A	4	A	4	AA	4	1
*C. chinensis*	A	2	A	3	AA	2	2
	B	1			BA	1	
*C. pacifica*	A	2	A	1	AA	1	3
	B	1	B	1	AC	1	
			C	1	BB	1	
*C. yunnanensis*	A	2	A	1	AA	1	2
			B	1	AB	1	
*C. canadiana*	A	1	A	1	AA	1	1

^a^ Different letters indicate different genotypes.

**Table 3:** Isolates sequenced and used for phylogenetic analyses in this study.

Species Complex	Species	Genotype ^a^	Site and Tree Species Code ^b^	Isolate No. ^c^	Sample and Isolate Information ^d^	GenBank Accession No. ^e^					
						*tef1*	*tub2*	*cmdA*	*his3*	*rpb2*	*act*
*C. kyotensis*	*C. aconidialis*	AAABAA	3. FuJian-Cun.	CSF22498	20210525-1-(4)	OQ188649	OQ260624	OQ261463	OQ302898	OQ303105	OQ303311
*C. kyotensis*	*C. aconidialis*	ABAAAA	4. GuangDong-Euc.	CSF23317	20210607-1-(154)	OQ188839	OQ260814	OQ261464	OQ302899	OQ303106	OQ303312
*C. kyotensis*	*C. aconidialis*	ABAAAA	5. GuangDong-Pin.	CSF22562	20210606-1-(17)	OQ189007	OQ260982	OQ261465	OQ302900	OQ303107	OQ303313
*C. kyotensis*	*C. aconidialis*	ACAAAA	7. GuangXi-Euc.	CSF23671	20210624-1-(25)	OQ189296	OQ261271	OQ261466	OQ302901	OQ303108	OQ303314
*C. kyotensis*	*C. aconidialis*	ACAAAA	7. GuangXi-Euc.	CSF23740	20210624-1-(128)	OQ189297	OQ261272	OQ261467	OQ302902	OQ303109	OQ303315
*C. kyotensis*	*C. aconidialis*	ADAAAA	1. FuJian-Euc.	CSF22962	20210526-1-(73)	OQ188313	OQ260288	OQ261468	OQ302903	OQ303110	OQ303316
*C. kyotensis*	*C. aconidialis*	ADDAAA	3. FuJian-Cun.	CSF22991	20210525-1-(74)	OQ188650	OQ260625	OQ261469	OQ302904	OQ303111	OQ303317
*C. kyotensis*	*C. aconidialis*	AEAAAA	4. GuangDong-Euc.	CSF23379	20210607-1-(231)	OQ188840	OQ260815	OQ261470	OQ302905	OQ303112	OQ303318
*C. kyotensis*	*C. aconidialis*	AFAAAA	3. FuJian-Cun.	CSF23011	20210525-1-(141)	OQ188651	OQ260626	OQ261471	OQ302906	OQ303113	OQ303319
*C. kyotensis*	*C. aconidialis*	AFEAAA	5. GuangDong-Pin.	CSF23623	20210606-1-(238)	OQ189014	OQ260989	OQ261472	OQ302907	OQ303114	OQ303320
*C. kyotensis*	*C. aconidialis*	AGAAAA	1. FuJian-Euc.	CSF22495	20210527-1-(24)	OQ188314	OQ260289	OQ261473	OQ302908	OQ303115	OQ303321
*C. kyotensis*	*C. aconidialis*	AHAAAA	5. GuangDong-Pin.	CSF23621	20210606-1-(236)	OQ189015	OQ260990	OQ261474	OQ302909	OQ303116	OQ303322
*C. kyotensis*	*C. aconidialis*	AHAAAA	6. GuangDong-Cun.	CSF23409	20210609-1-(92)	OQ189151	OQ261126	OQ261475	OQ302910	OQ303117	OQ303323
*C. kyotensis*	*C. aconidialis*	AIAAAA	2. FuJian-Pin.	CSF23113	20210524-1-(135)	OQ188476	OQ260451	OQ261476	OQ302911	OQ303118	OQ303324
*C. kyotensis*	*C. aconidialis*	AIAAAA	3. FuJian-Cun.	CSF23008	20210525-1-(137)	OQ188656	OQ260631	OQ261477	OQ302912	OQ303119	OQ303325
*C. kyotensis*	*C. aconidialis*	AJAAAA	5. GuangDong-Pin.	CSF23585	20210606-1-(198)	OQ189016	OQ260991	OQ261478	OQ302913	OQ303120	OQ303326
*C. kyotensis*	*C. aconidialis*	AKAAAB	10. YunNan-Euc.	CSF22709	20210708-1-(11)	OQ189438	OQ261413	OQ261479	OQ302914	OQ303121	OQ303327
*C. kyotensis*	*C. aconidialis*	AKAAAB	10. YunNan-Euc.	CSF23815	20210708-1-(133)	OQ189439	OQ261414	OQ261480	OQ302915	OQ303122	OQ303328
*C. kyotensis*	*C. aconidialis*	ALCAAA	7. GuangXi-Euc.	CSF22578	20210621-1-(5)	OQ189306	OQ261281	OQ261481	OQ302916	OQ303123	OQ303329
*C. kyotensis*	*C. aconidialis*	ALCAAA	7. GuangXi-Euc.	CSF23747	20210624-1-(144)	OQ189307	OQ261282	OQ261482	OQ302917	OQ303124	OQ303330
*C. kyotensis*	*C. aconidialis*	AMCAAA	6. GuangDong-Cun.	CSF22542	20210609-1-(3)	OQ189152	OQ261127	OQ261483	OQ302918	OQ303125	OQ303331
*C. kyotensis*	*C. aconidialis*	AMCDAA	7. GuangXi-Euc.	CSF22599	20210621-1-(15)	OQ189308	OQ261283	OQ261484	OQ302919	OQ303126	OQ303332
*C. kyotensis*	*C. aconidialis*	ANAAAA	10. YunNan-Euc.	CSF23811	20210708-1-(103)	OQ189440	OQ261415	OQ261485	OQ302920	OQ303127	OQ303333
*C. kyotensis*	*C. aconidialis*	AOCAAA	4. GuangDong-Euc.	CSF23284	20210607-1-(112)	OQ188844	OQ260819	OQ261486	OQ302921	OQ303128	OQ303334
*C. kyotensis*	*C. aconidialis*	APAAAA	2. FuJian-Pin.	CSF23133	20210524-1-(158)	OQ188477	OQ260452	OQ261487	OQ302922	OQ303129	OQ303335
*C. kyotensis*	*C. aconidialis*	AQAAAA	3. FuJian-Cun.	CSF22503	20210525-1-(12)	OQ188657	OQ260632	OQ261488	OQ302923	OQ303130	OQ303336
*C. kyotensis*	*C. aconidialis*	ARAAAA	4. GuangDong-Euc.	CSF23251	20210607-1-(79)	OQ188848	OQ260823	OQ261489	OQ302924	OQ303131	OQ303337
*C. kyotensis*	*C. aconidialis*	ARAAAA	5. GuangDong-Pin.	CSF23444	20210606-1-(35)	OQ189017	OQ260992	OQ261490	OQ302925	OQ303132	OQ303338
*C. kyotensis*	*C. aconidialis*	ASAAAA	5. GuangDong-Pin.	CSF23497	20210606-1-(96)	OQ189018	OQ260993	OQ261491	OQ302926	OQ303133	OQ303339
*C. kyotensis*	*C. aconidialis*	ASACAA	4. GuangDong-Euc.	CSF22524	20210607-1-(1)	OQ188849	OQ260824	OQ261492	OQ302927	OQ303134	OQ303340
*C. kyotensis*	*C. aconidialis*	ATAAAA	6. GuangDong-Cun.	CSF23429	20210609-1-(213)	OQ189154	OQ261129	OQ261493	OQ302928	OQ303135	OQ303341
*C. kyotensis*	*C. aconidialis*	ATAAAA	7. GuangXi-Euc.	CSF22596	20210621-1-(14)	OQ189314	OQ261289	OQ261494	OQ302929	OQ303136	OQ303342
*C. kyotensis*	*C. aconidialis*	AUAAAA	1. FuJian-Euc.	CSF22813	20210527-1-(70)	OQ188349	OQ260324	OQ261495	OQ302930	OQ303137	OQ303343
*C. kyotensis*	*C. aconidialis*	AUAAAA	3. FuJian-Cun.	CSF23021	20210525-1-(185)	OQ188664	OQ260639	OQ261496	OQ302931	OQ303138	OQ303344
*C. kyotensis*	*C. aconidialis*	BAAAAA	7. GuangXi-Euc.	CSF23761	20210624-1-(162)	OQ189315	OQ261290	OQ261497	OQ302932	OQ303139	OQ303345
*C. kyotensis*	*C. aconidialis*	CAAAAA	1. FuJian-Euc.	CSF22912	20210526-1-(17)	OQ188363	OQ260338	OQ261498	OQ302933	OQ303140	OQ303346
*C. kyotensis*	*C. aconidialis*	CAAAAA	1. FuJian-Euc.	CSF22951	20210526-1-(60)	OQ188364	OQ260339	OQ261499	OQ302934	OQ303141	OQ303347
*C. kyotensis*	*C. aconidialis*	CAAAAA	1. FuJian-Euc.	CSF22802	20210527-1-(59)	OQ188362	OQ260337	OQ261500	OQ302935	OQ303142	OQ303348
*C. kyotensis*	*C. aconidialis*	CAAAAA	3. FuJian-Cun.	CSF23049	20210525-1-(250)	OQ188676	OQ260651	OQ261501	OQ302936	OQ303143	OQ303349
*C. kyotensis*	*C. aconidialis*	CABAAA	2. FuJian-Pin.	CSF23196	20210524-1-(243)	OQ188490	OQ260465	OQ261502	OQ302937	OQ303144	OQ303350
*C. kyotensis*	*C. aconidialis*	CADAAA	1. FuJian-Euc.	CSF22483	20210527-1-(12)	OQ188365	OQ260340	OQ261503	OQ302938	OQ303145	OQ303351
*C. kyotensis*	*C. aconidialis*	CADAAA	2. FuJian-Pin.	CSF23147	20210524-1-(177)	OQ188491	OQ260466	OQ261504	OQ302939	OQ303146	OQ303352
*C. kyotensis*	*C. aconidialis*	CADAAA	3. FuJian-Cun.	CSF23002	20210525-1-(110)	OQ188677	OQ260652	OQ261505	OQ302940	OQ303147	OQ303353
*C. kyotensis*	*C. aconidialis*	DADAAA	1. FuJian-Euc.	CSF22948	20210526-1-(57)	OQ188366	OQ260341	OQ261506	OQ302941	OQ303148	OQ303354
*C. kyotensis*	*C. aconidialis*	EAAAAA	7. GuangXi-Euc.	CSF23741	20210624-1-(132)	OQ189317	OQ261292	OQ261507	OQ302942	OQ303149	OQ303355
*C. kyotensis*	*C. aconidialis*	EAAAAA	7. GuangXi-Euc.	CSF23779	20210624-2-(9)	OQ189318	OQ261293	OQ261508	OQ302943	OQ303150	OQ303356
*C. kyotensis*	*C. aconidialis*	FAAAAA	6. GuangDong-Cun.	CSF23401	20210609-1-(52)	OQ189155	OQ261130	OQ261509	OQ302944	OQ303151	OQ303357
*C. kyotensis*	*C. aconidialis*	GAAAAA	4. GuangDong-Euc.	CSF23306	20210607-1-(141)	OQ188883	OQ260858	OQ261510	OQ302945	OQ303152	OQ303358
*C. kyotensis*	*C. aconidialis*	GAAAAA	5. GuangDong-Pin.	CSF23563	20210606-1-(173)	OQ189036	OQ261011	OQ261511	OQ302946	OQ303153	OQ303359
*C. kyotensis*	*C. aconidialis*	GOCAAA	4. GuangDong-Euc.	CSF23221	20210607-1-(46)	OQ188885	OQ260860	OQ261512	OQ302947	OQ303154	OQ303360
*C. kyotensis*	*C. aconidialis*	GOCAAA	5. GuangDong-Pin.	CSF23547	20210606-1-(156)	OQ189037	OQ261012	OQ261513	OQ302948	OQ303155	OQ303361
*C. kyotensis*	*C. asiatica*	AAAAAA	10. YunNan-Euc.	CSF22708	20210708-1-(9)	OQ189460	OQ261435	OQ261514	OQ302949	OQ303156	OQ303362
*C. kyotensis*	*C. asiatica*	AAAAAA	10. YunNan-Euc.	CSF23833	20210708-1-(201)	OQ189461	OQ261436	OQ261515	OQ302950	OQ303157	OQ303363
*C. kyotensis*	*C. asiatica*	ABAAAA	10. YunNan-Euc.	CSF23796	20210708-1-(28)	OQ189469	OQ261444	OQ261516	OQ302951	OQ303158	OQ303364
*C. kyotensis*	*C. asiatica*	ABAAAB	10. YunNan-Euc.	CSF23830	20210708-1-(180)	OQ189470	OQ261445	OQ261517	OQ302952	OQ303159	OQ303365
*C. kyotensis*	*C. canadiana*	AAAAAA	12. YunNan-Cun.	CSF22750	20210707-1-(141)	OQ189487	OQ261462	OQ261518	OQ302953	OQ303160	OQ303366
*C. kyotensis*	*C. chinensis*	AAAAAA	1. FuJian-Euc.	CSF22960	20210526-1-(70)	OQ188367	OQ260342	OQ261519	OQ302954	OQ303161	OQ303367
*C. kyotensis*	*C. chinensis*	AAAAAA	3. FuJian-Cun.	CSF22980	20210525-1-(41)	OQ188678	OQ260653	OQ261520	OQ302955	OQ303162	OQ303368
*C. kyotensis*	*C. chinensis*	BAAAAA	3. FuJian-Cun.	CSF22981	20210525-1-(43)	OQ188679	OQ260654	OQ261521	OQ302956	OQ303163	OQ303369
*C. kyotensis*	*C. curvispora*	AAAAAA	5. GuangDong-Pin.	CSF22555	20210606-1-(9)	OQ189040	OQ261015	OQ261522	OQ302957	OQ303164	OQ303370
*C. kyotensis*	*C. curvispora*	AAAAAA	5. GuangDong-Pin.	CSF23447	20210606-1-(38)	OQ189041	OQ261016	OQ261523	OQ302958	OQ303165	OQ303371
*C. kyotensis*	*C. hongkongensis*	AAAAAA	1. FuJian-Euc.	CSF22931	20210526-1-(38)	OQ188388	OQ260363	OQ261524	OQ302959	OQ303166	OQ303372
*C. kyotensis*	*C. hongkongensis*	AAAAAA	5. GuangDong-Pin.	CSF22552	20210606-1-(6)	OQ189076	OQ261051	OQ261525	OQ302960	OQ303167	OQ303373
*C. kyotensis*	*C. hongkongensis*	ABAAAA	1. FuJian-Euc.	CSF22895	20210526-2-(43)	OQ188389	OQ260364	OQ261526	OQ302961	OQ303168	OQ303374
*C. kyotensis*	*C. hongkongensis*	ABBAAA	5. GuangDong-Pin.	CSF23580	20210606-1-(191)	OQ189079	OQ261054	OQ261527	OQ302962	OQ303169	OQ303375
*C. kyotensis*	*C. hongkongensis*	ACAAAA	4. GuangDong-Euc.	CSF23258	20210607-1-(86)	OQ188895	OQ260870	OQ261528	OQ302963	OQ303170	OQ303376
*C. kyotensis*	*C. hongkongensis*	ACAAAA	5. GuangDong-Pin.	CSF23476	20210606-1-(73)	OQ189080	OQ261055	OQ261529	OQ302964	OQ303171	OQ303377
*C. kyotensis*	*C. hongkongensis*	ADBAAA	3. FuJian-Cun.	CSF22501	20210525-1-(10)	OQ188684	OQ260659	OQ261530	OQ302965	OQ303172	OQ303378
*C. kyotensis*	*C. hongkongensis*	ADBAAB	5. GuangDong-Pin.	CSF23471	20210606-1-(64)	OQ189083	OQ261058	OQ261531	OQ302966	OQ303173	OQ303379
*C. kyotensis*	*C. hongkongensis*	AEBAAA	2. FuJian-Pin.	CSF23136	20210524-1-(161)	OQ188521	OQ260496	OQ261532	OQ302967	OQ303174	OQ303380
*C. kyotensis*	*C. hongkongensis*	AEBAAA	5. GuangDong-Pin.	CSF23443	20210606-1-(34)	OQ189085	OQ261060	OQ261533	OQ302968	OQ303175	OQ303381
*C. kyotensis*	*C. hongkongensis*	AFAAAA	1. FuJian-Euc.	CSF22909	20210526-1-(14)	OQ188390	OQ260365	OQ261534	OQ302969	OQ303176	OQ303382
*C. kyotensis*	*C. hongkongensis*	AFAAAA	1. FuJian-Euc.	CSF22949	20210526-1-(58)	OQ188391	OQ260366	OQ261535	OQ302970	OQ303177	OQ303383
*C. kyotensis*	*C. hongkongensis*	AFAAAA	2. FuJian-Pin.	CSF23068	20210524-1-(59)	OQ188527	OQ260502	OQ261536	OQ302971	OQ303178	OQ303384
*C. kyotensis*	*C. hongkongensis*	AFAAAA	5. GuangDong-Pin.	CSF23470	20210606-1-(63)	OQ189089	OQ261064	OQ261537	OQ302972	OQ303179	OQ303385
*C. kyotensis*	*C. hongkongensis*	AFAAAA	7. GuangXi-Euc.	CSF23718	20210624-1-(83)	OQ189319	OQ261294	OQ261538	OQ302973	OQ303180	OQ303386
*C. kyotensis*	*C. hongkongensis*	AFABAA	4. GuangDong-Euc.	CSF23366	20210607-1-(214)	OQ188897	OQ260872	OQ261539	OQ302974	OQ303181	OQ303387
*C. kyotensis*	*C. hongkongensis*	AFBAAA	1. FuJian-Euc.	CSF22810	20210527-1-(67)	OQ188392	OQ260367	OQ261540	OQ302975	OQ303182	OQ303388
*C. kyotensis*	*C. hongkongensis*	AFBAAA	2. FuJian-Pin.	CSF23142	20210524-1-(168)	OQ188528	OQ260503	OQ261541	OQ302976	OQ303183	OQ303389
*C. kyotensis*	*C. hongkongensis*	AFBAAA	5. GuangDong-Pin.	CSF23602	20210606-1-(217)	OQ189090	OQ261065	OQ261542	OQ302977	OQ303184	OQ303390
*C. kyotensis*	*C. hongkongensis*	AFCAAA	3. FuJian-Cun.	CSF23000	20210525-1-(98)	OQ188685	OQ260660	OQ261543	OQ302978	OQ303185	OQ303391
*C. kyotensis*	*C. hongkongensis*	AGBAAA	2. FuJian-Pin.	CSF23137	20210524-1-(162)	OQ188529	OQ260504	OQ261544	OQ302979	OQ303186	OQ303392
*C. kyotensis*	*C. hongkongensis*	AGBAAA	2. FuJian-Pin.	CSF23166	20210524-1-(200)	OQ188530	OQ260505	OQ261545	OQ302980	OQ303187	OQ303393
*C. kyotensis*	*C. hongkongensis*	AGBAAA	8. GuangXi-Pin.	CSF22662	20210622-1-(21)	OQ189381	OQ261356	OQ261546	OQ302981	OQ303188	OQ303394
*C. kyotensis*	*C. hongkongensis*	AHAAAA	1. FuJian-Euc.	CSF22921	20210526-1-(26)	OQ188393	OQ260368	OQ261547	OQ302982	OQ303189	OQ303395
*C. kyotensis*	*C. hongkongensis*	AHAAAA	5. GuangDong-Pin.	CSF23506	20210606-1-(108)	OQ189091	OQ261066	OQ261548	OQ302983	OQ303190	OQ303396
*C. kyotensis*	*C. hongkongensis*	AIAAAA	1. FuJian-Euc.	CSF22954	20210526-1-(64)	OQ188394	OQ260369	OQ261549	OQ302984	OQ303191	OQ303397
*C. kyotensis*	*C. hongkongensis*	BFBAAA	7. GuangXi-Euc.	CSF23782	20210624-2-(13)	OQ189320	OQ261295	OQ261550	OQ302985	OQ303192	OQ303398
*C. kyotensis*	*C. ilicicola*	AAAAAA	8. GuangXi-Pin.	CSF22680	20210622-1-(55)	OQ189384	OQ261359	OQ261551	OQ302986	OQ303193	OQ303399
*C. kyotensis*	*C. ilicicola*	AAAA-A	9. GuangXi-Cun.	CSF22632	20210623-1-(96)	OQ189413	OQ261388	OQ261552	OQ302987	– ^f^	OQ303400
*C. kyotensis*	*C. ilicicola*	AABAAA	2. FuJian-Pin.	CSF23189	20210524-1-(231)	OQ188534	OQ260509	OQ261553	OQ302988	OQ303194	OQ303401
*C. kyotensis*	*C. ilicicola*	BAAAAA	4. GuangDong-Euc.	CSF23220	20210607-1-(45)	OQ188898	OQ260873	OQ261554	OQ302989	OQ303195	OQ303402
*C. kyotensis*	*C. ilicicola*	BAAAAA	5. GuangDong-Pin.	CSF23489	20210606-1-(88)	OQ189093	OQ261068	OQ261555	OQ302990	OQ303196	OQ303403
*C. kyotensis*	*C. ilicicola*	CAAABB	10. YunNan-Euc.	CSF23806	20210708-1-(59)	OQ189474	OQ261449	OQ261556	OQ302991	OQ303197	OQ303404
*C. kyotensis*	*C. ilicicola*	CAAABB	10. YunNan-Euc.	CSF23829	20210708-1-(178)	OQ189475	OQ261450	OQ261557	OQ302992	OQ303198	OQ303405
*C. kyotensis*	*C. kyotensis*	AAAAAA	1. FuJian-Euc.	CSF22937	20210526-1-(44)	OQ188399	OQ260374	OQ261558	OQ302993	OQ303199	OQ303406
*C. kyotensis*	*C. kyotensis*	AAAAAA	2. FuJian-Pin.	CSF23086	20210524-1-(93)	OQ188536	OQ260511	OQ261559	OQ302994	OQ303200	OQ303407
*C. kyotensis*	*C. kyotensis*	ADAAAA	1. FuJian-Euc.	CSF22894	20210526-2-(42)	OQ188400	OQ260375	OQ261560	OQ302995	OQ303201	OQ303408
*C. kyotensis*	*C. kyotensis*	ADAAAA	2. FuJian-Pin.	CSF23115	20210524-1-(137)	OQ188537	OQ260512	OQ261561	OQ302996	OQ303202	OQ303409
*C. kyotensis*	*C. kyotensis*	ADAAAA	2. FuJian-Pin.	CSF23120	20210524-1-(142)	OQ188538	OQ260513	OQ261562	OQ302997	OQ303203	OQ303410
*C. kyotensis*	*C. kyotensis*	ADAAAA	5. GuangDong-Pin.	CSF23614	20210606-1-(229)	OQ189095	OQ261070	OQ261563	OQ302998	OQ303204	OQ303411
*C. kyotensis*	*C. kyotensis*	AFAAAA	1. FuJian-Euc.	CSF22869	20210526-2-(7)	OQ188405	OQ260380	OQ261564	OQ302999	OQ303205	OQ303412
*C. kyotensis*	*C. kyotensis*	AFAAAA	2. FuJian-Pin.	CSF23163	20210524-1-(197)	OQ188542	OQ260517	OQ261565	OQ303000	OQ303206	OQ303413
*C. kyotensis*	*C. kyotensis*	AIAAAA	1. FuJian-Euc.	CSF22904	20210526-1-(8)	OQ188407	OQ260382	OQ261566	OQ303001	OQ303207	OQ303414
*C. kyotensis*	*C. kyotensis*	AIAAAA	1. FuJian-Euc.	CSF22866	20210526-2-(3)	OQ188408	OQ260383	OQ261567	OQ303002	OQ303208	OQ303415
*C. kyotensis*	*C. kyotensis*	AIDAAA	4. GuangDong-Euc.	CSF23316	20210607-1-(153)	OQ188899	OQ260874	OQ261568	OQ303003	OQ303209	OQ303416
*C. kyotensis*	*C. kyotensis*	AIDAAA	5. GuangDong-Pin.	CSF23480	20210606-1-(79)	OQ189096	OQ261071	OQ261569	OQ303004	OQ303210	OQ303417
*C. kyotensis*	*C. kyotensis*	AIDAAA	5. GuangDong-Pin.	CSF23555	20210606-1-(164)	OQ189097	OQ261072	OQ261570	OQ303005	OQ303211	OQ303418
*C. kyotensis*	*C. kyotensis*	AIDABA	2. FuJian-Pin.	CSF23104	20210524-1-(123)	OQ188548	OQ260523	OQ261571	OQ303006	OQ303212	OQ303419
*C. kyotensis*	*C. kyotensis*	AIFAAA	2. FuJian-Pin.	CSF23181	20210524-1-(222)	OQ188549	OQ260524	OQ261572	OQ303007	OQ303213	OQ303420
*C. kyotensis*	*C. kyotensis*	AKAAAA	2. FuJian-Pin.	CSF23070	20210524-1-(68)	OQ188550	OQ260525	OQ261573	OQ303008	OQ303214	OQ303421
*C. kyotensis*	*C. kyotensis*	AKAAAA	2. FuJian-Pin.	CSF23096	20210524-1-(108)	OQ188551	OQ260526	OQ261574	OQ303009	OQ303215	OQ303422
*C. kyotensis*	*C. kyotensis*	ALABAA	2. FuJian-Pin.	CSF23098	20210524-1-(112)	OQ188554	OQ260529	OQ261575	OQ303010	OQ303216	OQ303423
*C. kyotensis*	*C. kyotensis*	ALBAAA	2. FuJian-Pin.	CSF22516	20210524-1-(14)	OQ188555	OQ260530	OQ261576	OQ303011	OQ303217	OQ303424
*C. kyotensis*	*C. kyotensis*	AOAAAA	2. FuJian-Pin.	CSF23094	20210524-1-(104)	OQ188556	OQ260531	OQ261577	OQ303012	OQ303218	OQ303425
*C. kyotensis*	*C. kyotensis*	AOAAAA	5. GuangDong-Pin.	CSF23468	20210606-1-(60)	OQ189098	OQ261073	OQ261578	OQ303013	OQ303219	OQ303426
*C. kyotensis*	*C. kyotensis*	AOAAAA	5. GuangDong-Pin.	CSF23481	20210606-1-(80)	OQ189099	OQ261074	OQ261579	OQ303014	OQ303220	OQ303427
*C. kyotensis*	*C. kyotensis*	AOAAAA	5. GuangDong-Pin.	CSF23572	20210606-1-(182)	OQ189100	OQ261075	OQ261580	OQ303015	OQ303221	OQ303428
*C. kyotensis*	*C. kyotensis*	AODAAA	5. GuangDong-Pin.	CSF23455	20210606-1-(47)	OQ189101	OQ261076	OQ261581	OQ303016	OQ303222	OQ303429
*C. kyotensis*	*C. kyotensis*	AODAAA	5. GuangDong-Pin.	CSF23584	20210606-1-(196)	OQ189102	OQ261077	OQ261582	OQ303017	OQ303223	OQ303430
*C. kyotensis*	*C. kyotensis*	APAAAA	5. GuangDong-Pin.	CSF23505	20210606-1-(107)	OQ189103	OQ261078	OQ261583	OQ303018	OQ303224	OQ303431
*C. kyotensis*	*C. kyotensis*	ARAAAA	1. FuJian-Euc.	CSF22950	20210526-1-(59)	OQ188409	OQ260384	OQ261584	OQ303019	OQ303225	OQ303432
*C. kyotensis*	*C. kyotensis*	ARAAAA	5. GuangDong-Pin.	CSF23437	20210606-1-(27)	OQ189104	OQ261079	OQ261585	OQ303020	OQ303226	OQ303433
*C. kyotensis*	*C. kyotensis*	BRAAAA	1. FuJian-Euc.	CSF22889	20210526-2-(35)	OQ188410	OQ260385	OQ261586	OQ303021	OQ303227	OQ303434
*C. kyotensis*	*C. kyotensis*	CAABAA	7. GuangXi-Euc.	CSF22586	20210621-1-(9)	OQ189324	OQ261299	OQ261587	OQ303022	OQ303228	OQ303435
*C. kyotensis*	*C. kyotensis*	CADAAA	7. GuangXi-Euc.	CSF23738	20210624-1-(121)	OQ189325	OQ261300	OQ261588	OQ303023	OQ303229	OQ303436
*C. kyotensis*	*C. kyotensis*	CADAAA	7. GuangXi-Euc.	CSF23784	20210624-2-(15)	OQ189326	OQ261301	OQ261589	OQ303024	OQ303230	OQ303437
*C. kyotensis*	*C. kyotensis*	CADBAA	7. GuangXi-Euc.	CSF23716	20210624-1-(81)	OQ189327	OQ261302	OQ261590	OQ303025	OQ303231	OQ303438
*C. kyotensis*	*C. kyotensis*	CADDAA	7. GuangXi-Euc.	CSF23644	20210621-1-(37)	OQ189328	OQ261303	OQ261591	OQ303026	OQ303232	OQ303439
*C. kyotensis*	*C. kyotensis*	CADDAA	8. GuangXi-Pin.	CSF22683	20210622-1-(58)	OQ189385	OQ261360	OQ261592	OQ303027	OQ303233	OQ303440
*C. kyotensis*	*C. kyotensis*	CBAAAA	2. FuJian-Pin.	CSF23110	20210524-1-(132)	OQ188557	OQ260532	OQ261593	OQ303028	OQ303234	OQ303441
*C. kyotensis*	*C. kyotensis*	CEADAA	7. GuangXi-Euc.	CSF23660	20210624-1-(3)	OQ189329	OQ261304	OQ261594	OQ303029	OQ303235	OQ303442
*C. kyotensis*	*C. kyotensis*	CEDDAA	7. GuangXi-Euc.	CSF23711	20210624-1-(76)	OQ189330	OQ261305	OQ261595	OQ303030	OQ303236	OQ303443
*C. kyotensis*	*C. kyotensis*	CFAAAA	1. FuJian-Euc.	CSF22907	20210526-1-(12)	OQ188412	OQ260387	OQ261596	OQ303031	OQ303237	OQ303444
*C. kyotensis*	*C. kyotensis*	CFAAAA	2. FuJian-Pin.	CSF23114	20210524-1-(136)	OQ188559	OQ260534	OQ261597	OQ303032	OQ303238	OQ303445
*C. kyotensis*	*C. kyotensis*	CHDBAA	7. GuangXi-Euc.	CSF23697	20210624-1-(53)	OQ189331	OQ261306	OQ261598	OQ303033	OQ303239	OQ303446
*C. kyotensis*	*C. kyotensis*	CIAAAA	2. FuJian-Pin.	CSF23176	20210524-1-(214)	OQ188560	OQ260535	OQ261599	OQ303034	OQ303240	OQ303447
*C. kyotensis*	*C. kyotensis*	CMAAAA	1. FuJian-Euc.	CSF22778	20210527-1-(29)	OQ188413	OQ260388	OQ261600	OQ303035	OQ303241	OQ303448
*C. kyotensis*	*C. kyotensis*	CMDBAA	7. GuangXi-Euc.	CSF23765	20210624-1-(166)	OQ189332	OQ261307	OQ261601	OQ303036	OQ303242	OQ303449
*C. kyotensis*	*C. kyotensis*	CMDBAA	7. GuangXi-Euc.	CSF23769	20210624-1-(170)	OQ189333	OQ261308	OQ261602	OQ303037	OQ303243	OQ303450
*C. kyotensis*	*C. kyotensis*	CMDDAA	7. GuangXi-Euc.	CSF23715	20210624-1-(80)	OQ189334	OQ261309	OQ261603	OQ303038	OQ303244	OQ303451
*C. kyotensis*	*C. kyotensis*	CNDBAA	7. GuangXi-Euc.	CSF22594	20210621-1-(13)	OQ189335	OQ261310	OQ261604	OQ303039	OQ303245	OQ303452
*C. kyotensis*	*C. kyotensis*	CNDBAA	8. GuangXi-Pin.	CSF22646	20210621-3-(21)	OQ189386	OQ261361	OQ261605	OQ303040	OQ303246	OQ303453
*C. kyotensis*	*C. kyotensis*	COAAAA	7. GuangXi-Euc.	CSF23708	20210624-1-(70)	OQ189336	OQ261311	OQ261606	OQ303041	OQ303247	OQ303454
*C. kyotensis*	*C. kyotensis*	COABAA	7. GuangXi-Euc.	CSF23675	20210624-1-(30)	OQ189337	OQ261312	OQ261607	OQ303042	OQ303248	OQ303455
*C. kyotensis*	*C. kyotensis*	COABAA	7. GuangXi-Euc.	CSF23754	20210624-1-(154)	OQ189338	OQ261313	OQ261608	OQ303043	OQ303249	OQ303456
*C. kyotensis*	*C. kyotensis*	COABAA	7. GuangXi-Euc.	CSF23758	20210624-1-(158)	OQ189339	OQ261314	OQ261609	OQ303044	OQ303250	OQ303457
*C. kyotensis*	*C. kyotensis*	COABAA	7. GuangXi-Euc.	CSF23763	20210624-1-(164)	OQ189340	OQ261315	OQ261610	OQ303045	OQ303251	OQ303458
*C. kyotensis*	*C. kyotensis*	CODAAB	2. FuJian-Pin.	CSF23124	20210524-1-(146)	OQ188561	OQ260536	OQ261611	OQ303046	OQ303252	OQ303459
*C. kyotensis*	*C. kyotensis*	CODBAA	8. GuangXi-Pin.	CSF22665	20210622-1-(24)	OQ189387	OQ261362	OQ261612	OQ303047	OQ303253	OQ303460
*C. kyotensis*	*C. kyotensis*	CODDAA	7. GuangXi-Euc.	CSF23703	20210624-1-(64)	OQ189341	OQ261316	OQ261613	OQ303048	OQ303254	OQ303461
*C. kyotensis*	*C. kyotensis*	CODDAA	8. GuangXi-Pin.	CSF22698	20210622-1-(129)	OQ189388	OQ261363	OQ261614	OQ303049	OQ303255	OQ303462
*C. kyotensis*	*C. kyotensis*	CRAACA	6. GuangDong-Cun.	CSF23408	20210609-1-(84)	OQ189159	OQ261134	OQ261615	OQ303050	OQ303256	OQ303463
*C. kyotensis*	*C. kyotensis*	CRABDA	7. GuangXi-Euc.	CSF23696	20210624-1-(52)	OQ189342	OQ261317	OQ261616	OQ303051	OQ303257	OQ303464
*C. kyotensis*	*C. kyotensis*	CRABDA	7. GuangXi-Euc.	CSF23707	20210624-1-(69)	OQ189343	OQ261318	OQ261617	OQ303052	OQ303258	OQ303465
*C. kyotensis*	*C. kyotensis*	CRABDA	7. GuangXi-Euc.	CSF23722	20210624-1-(89)	OQ189344	OQ261319	OQ261618	OQ303053	OQ303259	OQ303466
*C. kyotensis*	*C. kyotensis*	CSDBAA	8. GuangXi-Pin.	CSF22688	20210622-1-(66)	OQ189389	OQ261364	OQ261619	OQ303054	OQ303260	OQ303467
*C. kyotensis*	*C. kyotensis*	CSDBAA	8. GuangXi-Pin.	CSF22696	20210622-1-(106)	OQ189390	OQ261365	OQ261620	OQ303055	OQ303261	OQ303468
*C. kyotensis*	*C. kyotensis*	CTDBAA	8. GuangXi-Pin.	CSF22669	20210622-1-(38)	OQ189391	OQ261366	OQ261621	OQ303056	OQ303262	OQ303469
*C. kyotensis*	*C. kyotensis*	CUDAAA	7. GuangXi-Euc.	CSF23744	20210624-1-(139)	OQ189345	OQ261320	OQ261622	OQ303057	OQ303263	OQ303470
*C. kyotensis*	*C. kyotensis*	CUDAAA	8. GuangXi-Pin.	CSF22667	20210622-1-(34)	OQ189392	OQ261367	OQ261623	OQ303058	OQ303264	OQ303471
*C. kyotensis*	*C. kyotensis*	DAAAAA	2. FuJian-Pin.	CSF23143	20210524-1-(169)	OQ188566	OQ260541	OQ261624	OQ303059	OQ303265	OQ303472
*C. kyotensis*	*C. kyotensis*	DADAAA	6. GuangDong-Cun.	CSF23418	20210609-1-(141)	OQ189161	OQ261136	OQ261625	OQ303060	OQ303266	OQ303473
*C. kyotensis*	*C. kyotensis*	DCABAA	5. GuangDong-Pin.	CSF23581	20210606-1-(193)	OQ189107	OQ261082	OQ261626	OQ303061	OQ303267	OQ303474
*C. kyotensis*	*C. kyotensis*	DDAABA	2. FuJian-Pin.	CSF23118	20210524-1-(140)	OQ188569	OQ260544	OQ261627	OQ303062	OQ303268	OQ303475
*C. kyotensis*	*C. kyotensis*	DDABAA	6. GuangDong-Cun.	CSF22546	20210609-1-(17)	OQ189162	OQ261137	OQ261628	OQ303063	OQ303269	OQ303476
*C. kyotensis*	*C. kyotensis*	DDDAAA	2. FuJian-Pin.	CSF23145	20210524-1-(175)	OQ188570	OQ260545	OQ261629	OQ303064	OQ303270	OQ303477
*C. kyotensis*	*C. kyotensis*	DDDAAA	4. GuangDong-Euc.	CSF23382	20210607-1-(234)	OQ188900	OQ260875	OQ261630	OQ303065	OQ303271	OQ303478
*C. kyotensis*	*C. kyotensis*	DEAAAA	7. GuangXi-Euc.	CSF23645	20210621-1-(38)	OQ189346	OQ261321	OQ261631	OQ303066	OQ303272	OQ303479
*C. kyotensis*	*C. kyotensis*	DFAAAA	1. FuJian-Euc.	CSF22928	20210526-1-(33)	OQ188419	OQ260394	OQ261632	OQ303067	OQ303273	OQ303480
*C. kyotensis*	*C. kyotensis*	DFBAAA	2. FuJian-Pin.	CSF23174	20210524-1-(211)	OQ188577	OQ260552	OQ261633	OQ303068	OQ303274	OQ303481
*C. kyotensis*	*C. kyotensis*	DGDAAA	5. GuangDong-Pin.	CSF23494	20210606-1-(93)	OQ189108	OQ261083	OQ261634	OQ303069	OQ303275	OQ303482
*C. kyotensis*	*C. kyotensis*	DIAAAA	2. FuJian-Pin.	CSF23052	20210524-1-(28)	OQ188585	OQ260560	OQ261635	OQ303070	OQ303276	OQ303483
*C. kyotensis*	*C. kyotensis*	DIEAAA	4. GuangDong-Euc.	CSF23389	20210607-1-(242)	OQ188901	OQ260876	OQ261636	OQ303071	OQ303277	OQ303484
*C. kyotensis*	*C. kyotensis*	DJBAAA	2. FuJian-Pin.	CSF23054	20210524-1-(31)	OQ188586	OQ260561	OQ261637	OQ303072	OQ303278	OQ303485
*C. kyotensis*	*C. kyotensis*	DKAAAA	1. FuJian-Euc.	CSF22918	20210526-1-(23)	OQ188428	OQ260403	OQ261638	OQ303073	OQ303279	OQ303486
*C. kyotensis*	*C. kyotensis*	DKAAAA	1. FuJian-Euc.	CSF22790	20210527-1-(43)	OQ188427	OQ260402	OQ261639	OQ303074	OQ303280	OQ303487
*C. kyotensis*	*C. kyotensis*	DKAAAA	2. FuJian-Pin.	CSF23109	20210524-1-(131)	OQ188588	OQ260563	OQ261640	OQ303075	OQ303281	OQ303488
*C. kyotensis*	*C. kyotensis*	DKAAAA	2. FuJian-Pin.	CSF23165	20210524-1-(199)	OQ188589	OQ260564	OQ261641	OQ303076	OQ303282	OQ303489
*C. kyotensis*	*C. kyotensis*	DKDAAA	1. FuJian-Euc.	CSF22890	20210526-2-(36)	OQ188429	OQ260404	OQ261642	OQ303077	OQ303283	OQ303490
*C. kyotensis*	*C. kyotensis*	DLAAAA	5. GuangDong-Pin.	CSF23507	20210606-1-(109)	OQ189110	OQ261085	OQ261643	OQ303078	OQ303284	OQ303491
*C. kyotensis*	*C. kyotensis*	DLCAAA	1. FuJian-Euc.	CSF22831	20210527-1-(90)	OQ188430	OQ260405	OQ261644	OQ303079	OQ303285	OQ303492
*C. kyotensis*	*C. kyotensis*	DMAAAA	1. FuJian-Euc.	CSF22864	20210526-2-(1)	OQ188433	OQ260408	OQ261645	OQ303080	OQ303286	OQ303493
*C. kyotensis*	*C. kyotensis*	DMAAAA	2. FuJian-Pin.	CSF23193	20210524-1-(239)	OQ188595	OQ260570	OQ261646	OQ303081	OQ303287	OQ303494
*C. kyotensis*	*C. kyotensis*	DOAAAA	2. FuJian-Pin.	CSF22519	20210524-1-(18)	OQ188597	OQ260572	OQ261647	OQ303082	OQ303288	OQ303495
*C. kyotensis*	*C. kyotensis*	DOAAAA	2. FuJian-Pin.	CSF23087	20210524-1-(94)	OQ188598	OQ260573	OQ261648	OQ303083	OQ303289	OQ303496
*C. kyotensis*	*C. kyotensis*	DOAAAA	5. GuangDong-Pin.	CSF23452	20210606-1-(43)	OQ189112	OQ261087	OQ261649	OQ303084	OQ303290	OQ303497
*C. kyotensis*	*C. kyotensis*	DODBAA	5. GuangDong-Pin.	CSF23582	20210606-1-(194)	OQ189113	OQ261088	OQ261650	OQ303085	OQ303291	OQ303498
*C. kyotensis*	*C. kyotensis*	DQAABA	1. FuJian-Euc.	CSF22492	20210527-1-(21)	OQ188434	OQ260409	OQ261651	OQ303086	OQ303292	OQ303499
*C. kyotensis*	*C. kyotensis*	DRDAAA	5. GuangDong-Pin.	CSF23475	20210606-1-(71)	OQ189114	OQ261089	OQ261652	OQ303087	OQ303293	OQ303500
*C. kyotensis*	*C. kyotensis*	DRDAAA	5. GuangDong-Pin.	CSF23534	20210606-1-(143)	OQ189115	OQ261090	OQ261653	OQ303088	OQ303294	OQ303501
*C. kyotensis*	*C. kyotensis*	DVACAA	4. GuangDong-Euc.	CSF23370	20210607-1-(219)	OQ188902	OQ260877	OQ261654	OQ303089	OQ303295	OQ303502
*C. kyotensis*	*C. kyotensis*	EADAAA	5. GuangDong-Pin.	CSF23512	20210606-1-(116)	OQ189116	OQ261091	OQ261655	OQ303090	OQ303296	OQ303503
*C. kyotensis*	*C. kyotensis*	ELAAAA	5. GuangDong-Pin.	CSF23499	20210606-1-(98)	OQ189117	OQ261092	OQ261656	OQ303091	OQ303297	OQ303504
*C. kyotensis*	*C. kyotensis*	EOAAAA	5. GuangDong-Pin.	CSF23474	20210606-1-(70)	OQ189118	OQ261093	OQ261657	OQ303092	OQ303298	OQ303505
*C. kyotensis*	*C. pacifica*	AAAAAA	5. GuangDong-Pin.	CSF23543	20210606-1-(151)	OQ189119	OQ261094	OQ261658	OQ303093	OQ303299	OQ303506
*C. kyotensis*	*C. pacifica*	ACAAAA	6. GuangDong-Cun.	CSF22544	20210609-1-(11)	OQ189164	OQ261139	OQ261659	OQ303094	OQ303300	OQ303507
*C. kyotensis*	*C. pacifica*	BBBAAA	5. GuangDong-Pin.	CSF23608	20210606-1-(223)	OQ189120	OQ261095	OQ261660	OQ303095	OQ303301	OQ303508
*C. kyotensis*	*C. yunnanensis*	AAAAAA	10. YunNan-Euc.	CSF23797	20210708-1-(31)	OQ189476	OQ261451	OQ261661	OQ303096	OQ303302	OQ303509
*C. kyotensis*	*C. yunnanensis*	ABAAAA	10. YunNan-Euc.	CSF23805	20210708-1-(47)	OQ189477	OQ261452	OQ261662	OQ303097	OQ303303	OQ303510
*C. colhounii*	*C. eucalypti*	AAAAAA	10. YunNan-Euc.	CSF23802	20210708-1-(41)	OQ189480	OQ261455	OQ261663	OQ303098	OQ303304	OQ303511
*C. colhounii*	*C. eucalypti*	AAAAAA	10. YunNan-Euc.	CSF23828	20210708-1-(162)	OQ189481	OQ261456	OQ261664	OQ303099	OQ303305	OQ303512
*C. colhounii*	*C. eucalypti*	BAAAAA	10. YunNan-Euc.	CSF23809	20210708-1-(88)	OQ189482	OQ261457	OQ261665	OQ303100	OQ303306	OQ303513
*C. colhounii*	*C. eucalypti*	BAAAAA	10. YunNan-Euc.	CSF23832	20210708-1-(197)	OQ189483	OQ261458	OQ261666	OQ303101	OQ303307	OQ303514
*C. colhounii*	*C. eucalypti*	CAAAAA	10. YunNan-Euc.	CSF23800	20210708-1-(37)	OQ189484	OQ261459	OQ261667	OQ303102	OQ303308	OQ303515
*C. colhounii*	*C. eucalypti*	DAAAAA	10. YunNan-Euc.	CSF23810	20210708-1-(99)	OQ189485	OQ261460	OQ261668	OQ303103	OQ303309	OQ303516
*C. colhounii*	*C. eucalypti*	DAAAAA	11. YunNan-Pin.	CSF23854	20210709-1-(224)	OQ189486	OQ261461	OQ261669	OQ303104	OQ303310	OQ303517

^a^ Genotype within each *Calonectria* species, determined by sequences of the *tef1*, *tub2*, *cmdA*, *his3*, *rpb2* and *act* regions; “-” means not available. ^b^ Code of 12 sampling sites connecting to “Site and Tree species code” in Table 1. ^c^ CSF: Culture Collection located at Research Institute of Fasting-growing Trees (RIFT), Chinese Academy of Forestry, ZhanJiang, GuangDong Province, China. ^d^ Information associated with sample point and isolate, for example, “20210525-1-(4)” indicates sample number “20210525-1-(4)” and isolate from this sample. ^e^ *tef1* = translation elongation factor 1-alpha; *tub2* = β-tubulin; *cmdA* = calmodulin; *his3* = histone H3; *rpb2* = the DNA-directed RNA polymerase II second largest subunit; *act* = actin. ^f^ “–” represents the relative locus that was not successfully amplified in this study.

**Table 4:** Isolates from other studies used in phylogenetic analyses in this study.

Species Code ^a^	Species	Isolate No. ^b,c^	Other Collection Number ^c^	Hosts	Area of Occurrence	Collector	GenBank Accession Numbers ^d^						References or Source of Data
							*act*	*cmdA*	*his3*	*rpb2*	*tef1*	*tub2*	
Species in *Calonectria kyotensis* species complex													
B4	*C. aconidialis*	CMW 35174^T^	CBS 136086; CERC 1850	Soil (*Eucalyptus* plantation)	HaiNan, China	X. Mou and S.F. Chen	MT334938	MT335165	MT335404	MT412479	MT412695	N/A ^e^	[18,30]
		CMW 35384	CBS 136091; CERC 1886	Soil (*Eucalyptus* plantation)	HaiNan, China	X. Mou and S.F. Chen	MT334939	MT335166	MT335405	N/A	MT412696	N/A	[18,30]
B5	*C. aeknauliensis*	CMW 48253^T^	CBS 143559	Soil (*Eucalyptus* plantation)	Aek Nauli, North Sumatra, Indonesia	M.J. Wingfield	MT334953	MT335180	MT335419	MT412486	MT412710	N/A	[9,30]
		CMW 48254	CBS 143560	Soil (*Eucalyptus* plantation)	Aek Nauli, North Sumatra, Indonesia	M.J. Wingfield	MT334954	MT335181	MT335420	MT412487	MT412711	N/A	[9,30]
B8	*C. asiatica*	CBS 114073^T^	CMW 23782; CPC 3900	Debris (leaf litter)	Prathet Thai, Thailand	M.J. Wingfield	GQ280428	AY725741	AY725658	N/A	AY725705	AY725616	[43,44]
B17	*C. brassicicola*	CBS 112841^T^	CMW 51206; CPC 4552	Soil (*Brassica* sp.)	Indonesia	M.J. Wingfield	N/A	KX784561	N/A	N/A	KX784689	KX784619	[45]
B19	*C. bumicola*	CMW 48257^T^	CBS 143575	Soil (*Eucalyptus* plantation)	Aek Nauli, North Sumatra, Indonesia	M.J. Wingfield	MT334975	MT335205	MT335445	MT412509	MT412736	N/A	[9,30]
B20	*C. canadiana*	CMW 23673^T^	CBS 110817; STE-U 499	*Picea* sp.	Canada	S. Greifenhagen	MT334976	MT335206	MT335446	MT412510	MT412737	MT412958	[1,30,46,47]
		CERC 8952	–	Soil	HeNan, China	S.F. Chen	MT335058	MT335290	MT335530	MT412587	MT412821	MT413035	[28,30]
B23	*C. chinensis*	CMW 23674^T^	CBS 114827; CPC 4101	Soil	Hong Kong, China	E.C.Y. Liew	MT334990	MT335220	MT335460	MT412524	MT412751	MT412972	[30,43,44]
		CMW 30986	CBS 112744; CPC 4104	Soil	Hong Kong, China	E.C.Y. Liew	MT334991	MT335221	MT335461	MT412525	MT412752	MT412973	[30,43,44]
B26	*C. cochinchinensis*	CMW 49915^T^	CBS 143567	Soil (*Hevea brasiliensis* plantation)	Duong Minh Chau, Tay Ninh, Vietnam	N.Q. Pham, Q.N. Dang and T.Q. Pham	MT334995	MT335225	MT335465	MT412529	MT412756	MT412977	[9,30]
		CMW 47186	CBS 143568	Soil (*A. auriculiformis* plantation)	Song May, Dong Nai, Vietnam	N.Q. Pham and T.Q. Pham	MT334996	MT335226	MT335466	MT412530	MT412757	MT412978	[9,30]
B29	*C. colombiensis*	CMW 23676^T^	CBS 112220; CPC 723	Soil (*E. grandis* trees)	La Selva, Colombia	M.J. Wingfield	MT334998	MT335228	MT335468	MT412532	MT412759	MT412980	[30,43]
		CMW 30985	CBS 112221; CPC 724	Soil (*E. grandis* trees)	La Selva, Colombia	M.J. Wingfield	MT334999	MT335229	MT335469	MT412533	MT412760	MT412981	[30,43]
B31	*C. curvispora*	CMW 23693^T^	CBS 116159; CPC 765	Soil	Tamatave, Madagascar	P.W. Crous	MT335002	MT335232	MT335472	MT412536	MT412763	N/A	[1,18,30,44,48]
		CMW 48245	CBS 143565	Soil (*Eucalyptus* plantation)	Aek Nauli, North Sumatra, Indonesia	M.J. Wingfield	MT335003	MT335233	MT335473	MT412537	MT412764	N/A	[9,30]
B46	*C. heveicola*	CMW 49913^T^	CBS 143570	Soil (*Hevea brasiliensis* plantation)	Bau Bang, Binh Duong, Vietnam	N.Q. Pham, Q.N. Dang and T.Q. Pham	MT335025	MT335255	MT335495	N/A	MT412786	MT413004	[9,30]
		CMW 49928	CBS 143571	Soil	Bu Gia Map National Park, Binh Phuoc, Vietnam	N.Q. Pham, Q.N. Dang and T.Q. Pham	MT335048	MT335280	MT335520	MT412577	MT412811	MT413025	[9,30]
B48	*C. hongkongensis*	CBS 114828^T^	CMW 51217; CPC 4670	Soil	Hong Kong, China	M.J. Wingfield	MT335028	MT335258	MT335498	MT412559	MT412789	MT413007	[30,43]
		CERC 3570	CMW 47271	Soil (*Eucalyptus* plantation)	BeiHai, GuangXi, China	S.F. Chen, J.Q. Li and G.Q. Li	MT335030	MT335260	MT335500	MT412561	MT412791	MT413009	[15,30]
B51	*C. ilicicola*	CMW 30998^T^	CBS 190.50; IMI 299389; STE-U 2482	*Solanum tuberosum*	Bogor, Java, Indonesia	K.B. Boedijn and J. Reitsma	MT335036	MT335266	MT335506	MT412564	MT412797	N/A	[1,30,44,49]
B52	*C. indonesiae*	CMW 23683^T^	CBS 112823; CPC 4508	*Syzygium aromaticum*	Warambunga, Indonesia	M.J. Wingfield	MT335037	MT335267	MT335507	MT412565	MT412798	MT413015	[30,43]
		CBS 112840	CMW 51205; CPC 4554	*S. aromaticum*	Warambunga, Indonesia	M.J. Wingfield	MT335038	MT335268	MT335508	MT412566	MT412799	MT413016	[30,43]
B55	*C. kyotensis*	CBS 114525^T^	ATCC 18834; CMW 51824; CPC 2367	*Robinia pseudoacacia*	Japan	T. Terashita	MT335039	MT335271	MT335511	MT412569	MT412802	MT413019	[1,30,45,50]
		CBS 114550	CMW 51825; CPC 2351	Soil	China	M.J. Wingfield	MT335016	MT335246	MT335486	MT412548	MT412777	MT412995	[30,45]
B57	*C. lantauensis*	CERC 3302^T^	CBS 142888; CMW 47252	Soil	LiDao, Hong Kong, China	M.J. Wingfield and S.F. Chen	MT335040	MT335272	MT335512	MT412570	MT412803	N/A	[15,30]
		CERC 3301	CBS 142887; CMW 47251	Soil	LiDao, Hong Kong, China	M.J. Wingfield and S.F. Chen	MT335041	MT335273	MT335513	N/A	MT412804	N/A	[15,30]
B58	*C. lateralis*	CMW 31412^T^	CBS 136629	Soil (*Eucalyptus* plantation)	GuangXi, China	X. Zhou, G. Zhao and F. Han	MT335042	MT335274	MT335514	MT412571	MT412805	MT413020	[18,30]
B66	*C. malesiana*	CMW 23687^T^	CBS 112752; CPC 4223	Soil	Northern Sumatra, Indonesia	M.J. Wingfield	MT335054	MT335286	MT335526	MT412583	MT412817	MT413031	[30,43]
		CBS 112710	CMW 51199; CPC 3899	Leaf litter	Prathet, Thailand	N.L. Hywel-Jones	MT335055	MT335287	MT335527	MT412584	MT412818	MT413032	[30,43]
B80	*C. pacifica*	CMW 16726^T^	A1568; CBS 109063; IMI 354528; STE-U 2534	*Araucaria heterophylla*	Hawaii, USA	M. Aragaki	MT335079	MT335311	MT335551	MT412604	MT412842	N/A	[1,30,43,46]
		CMW 30988	CBS 114038	*Ipomoea aquatica*	Auckland, New Zealand	C.F. Hill	MT335080	MT335312	MT335552	MT412605	MT412843	N/A	[1,30,43,44]
B86	*C. penicilloides*	CMW 23696^T^	CBS 174. 55; STE-U 2388	*Prunus* sp.	Hatizyo Island, Japan	M. Ookubu	MT335106	MT335338	MT335578	MT412631	MT412869	MT413081	[1,30,51]
B112	*C. sumatrensis*	CMW 23698^T^	CBS 112829; CPC 4518	Soil	Northern Sumatra, Indonesia	M.J. Wingfield	MT335145	MT335382	MT335622	MT412674	MT412913	N/A	[30,43]
		CMW 30987	CBS 112934; CPC 4516	Soil	Northern Sumatra, Indonesia	M.J. Wingfield	MT335146	MT335383	MT335623	MT412675	MT412914	N/A	[30,43]
B113	*C. syzygiicola*	CBS 112831^T^	CMW 51204; CPC 4511	*Syzygium aromaticum*	Sumatra, Indonesia	M.J. Wingfield	N/A	N/A	N/A	N/A	KX784736	KX784663	[45]
B116	*C. uniseptata*	CBS 413.67^T^	CMW 23678; CPC 2391; IMI 299577	*Paphiopedilum callosum*	Celle, Germany	W. Gerlach	GQ280451	GQ267379	GQ267248	N/A	GQ267307	GQ267208	[45]
B120	*C. yunnanensis*	CERC 5339^T^	CBS 142897; CMW 47644	Soil (*Eucalyptus* plantation)	YunNan, China	S.F. Chen and J.Q. Li	MT335157	MT335396	MT335636	MT412687	MT412927	MT413134	[15,30]
		CERC 5337	CBS 142895; CMW 47642	Soil (*Eucalyptus* plantation)	YunNan, China	S.F. Chen and J.Q. Li	MT335158	MT335397	MT335637	MT412688	MT412928	MT413135	[15,30]
B124	*C. singaporensis*	CBS 146715^T^	MUCL 048320	leaf litter submerged in a small stream	Mac Ritchie Reservoir, Singapore	C. Decock	MW890022.1	MW890042.1	MW890055.1	N/A	MW890086.1	MW890124.1	[40]
		CBS 146713	MUCL 048171	leaf litter submerged in a small stream	Mac Ritchie Reservoir, Singapore	C. Decock	MW890020.1	MW890040.1	MW890053.1	N/A	MW890084.1	MW890123.1	[40]
B127	*C. borneana*	CMW 50782^T^	CBS 144553	Soil (*Eucalyptus* plantation)	Brumas, Tawau, Sabah, Malaysia.	M.R.B.A Rauf	OL635115	OL635067	OL635043	OL635091	OL635019	N/A	[42]
		CMW 50832	CBS 144551	Soil (*Eucalyptus* plantation)	Brumas, Tawau, Sabah, Malaysia.	M.R.B.A Rauf	OL635113	OL635065	OL635041	OL635089	OL635017	N/A	[42]
B128	*C. ladang*	CMW 50776^T^	CBS 144550	Soil (*Eucalyptus* plantation)	Brumas, Tawau, Sabah, Malaysia.	M.R.B.A Rauf	OL635122	OL635075	OL635051	OL635099	OL635027	N/A	[42]
		CMW 50775	CBS 144549	Soil (*Eucalyptus* plantation)	Brumas, Tawau, Sabah, Malaysia.	M.R.B.A Rauf	OL635121	OL635074	OL635050	OL635098	OL635026	N/A	[42]
B129	*C. pseudomalesiana*	CMW 50821^T^	CBS 144563	Soil (*Eucalyptus* plantation)	Brumas, Tawau, Sabah, Malaysia.	M.J. Wingfield	OL635123	OL635076	OL635052	OL635100	OL635028	OL635137	[42]
		CMW 50779	CBS 144668	Soil (*Eucalyptus* plantation)	Brumas, Tawau, Sabah, Malaysia.	M.R.B.A Rauf	OL635124	OL635077	OL635053	OL635101	OL635029	OL635138	[42]
B130	*C. tanah*	CMW 50777^T^	CBS 144562	Soil (*Eucalyptus* plantation)	Brumas, Tawau, Sabah, Malaysia.	M.R.B.A Rauf	OL635134	OL635088	OL635064	OL635112	OL635040	OL635146	[42]
		CMW 50771	CBS 144560	Soil (*Eucalyptus* plantation)	Brumas, Tawau, Sabah, Malaysia.	M.R.B.A Rauf	OL635132	OL635086	OL635062	OL635110	OL635038	OL635144	[42]
	*C. cassiae*	ZHKUCC21-0011^T^	–	*Cassia surattensi*	GuangZhou, GuangDong, China	Y.X. Zhang	N/A	ON260790	N/A	N/A	MZ516860	MZ516863	[33]
		ZHKUCC21-0012	–	*Cassia surattensi*	GuangZhou, GuangDong, China	Y.X. Zhang	N/A	ON260791	N/A	N/A	MZ516861	MZ516864	[33]
Species in *Calonectria colhounii* species complex													
B3	*C. aciculata*	CERC 5342^T^	CBS 142883; CMW 47645	*Eucalyptus urophylla × E. grandis*	YunNan, China	S.F. Chen and J.Q. Li	MT334937	MT335164	MT335403	MT412478	MT412694	MT412934	[15,30]
B27	*C. colhounii*	CBS 293.79^T^	CMW 30999	*Camellia sinensis*	Mauritius	A. Peerally	GQ280443	GQ267373	DQ190639	KY653376	GQ267301	DQ190564	[1,44,52,53]
B36	*C. eucalypti*	CMW 18444^T^	CBS 125275	*E. grandis*	Aek Nauli, Sumatra Utara, Indonesia	M.J. Wingfield	MT335013	MT335243	MT335483	MT412545	MT412774	MT412992	[30,44]
		CMW 18445	CBS 125276	*E. grandis*	Aek Nauli, Sumatra Utara, Indonesia	M.J. Wingfield	MT335014	MT335244	MT335484	MT412546	MT412775	MT412993	[30,44]
B39	*C. fujianensis*	CMW 27257^T^	CBS 127201	*E. grandis*	FuJian, China	M.J. Wingfield	MT335019	MT335249	MT335489	MT412551	MT412780	MT412998	[16,30]
		CMW 27254	CBS 127200	*E. grandis*	FuJian, China	M.J. Wingfield	MT335020	MT335250	MT335490	MT412552	MT412781	MT41299	[16,30]
B47	*C. honghensis*	CERC 5572^T^	CBS 142885; CMW 47669	Soil (*Eucalyptus* plantation)	HongHe, YunNan, China	S.F. Chen and J.Q. Li	MT335026	MT335256	MT335496	MT412557	MT412787	MT413005	[15,30]
		CERC 5571	CBS 142884; CMW 47668	Soil (*Eucalyptus* plantation)	HongHe, YunNan, China	S.F. Chen and J.Q. Li	MT335027	MT335257	MT335497	MT412558	MT412788	MT413006	[15,30]
B53	*C. indusiata*	CBS 144.36^T^	CMW 23699	*Camellia sinensis*	Sri lanka	Unknown	GQ280536	GQ267453	GQ267262	KY653396	GQ267332	GQ267239	[1,44,45,54]
		CBS 114684	CMW 51213; CPC 2446; UFV16	*Rhododendron* sp.	Florida, USA	N.E. El-Gholl	GQ280537	GQ267454	DQ190653	N/A	GQ267333	AF232862	[1,53,55]
B62	*C. lichi*	CERC 8866^T^	–	Soil	HeNan, China	S.F. Chen	MT335046	MT335278	MT335518	MT412575	MT412809	MT413023	[28,30]
		CERC 8850	–	Soil	HeNan, China	S.F. Chen	MT335047	MT335279	MT335519	MT412576	MT412810	MT413024	[28,30]
B64	*C. macroaconidialis*	CBS 114880^T^	CMW 51219; CPC 307; PPRI 4000	*E. grandis*	Sabie, Mpumalanga, South Africa	P. W. Crous	MT335050	MT335282	MT335522	MT412579	MT412813	MT413027	[1,30,44,56]
B65	*C. madagascariensis*	CMW 23686^T^	CBS 114572; CPC 2252	Soil	Rona, Madagascar	J.E. Taylor	MT335052	MT335284	MT335524	MT412581	MT412815	MT413029	[1,30,44,53]
		CMW 30993	CBS 114571; CPC 2253	Soil	Rona, Madagascar	J.E. Taylor	MT335053	MT335285	MT335525	MT412582	MT412816	MT413030	[1,30,44,53]
B70	*C. monticola*	CBS 140645^T^	CPC 28835	Soil	Chiang Mai, Thailand	P. W. Crous	N/A	KT964771	N/A	N/A	KT964773	KT964769	[57]
		CPC 28836	–	Soil	Chiang Mai, Thailand	P. W. Crous	N/A	KT964772	N/A	N/A	KT964774	KT964770	[57]
B81	*C. paracolhounii*	CBS 114679^T^	CMW 51212; CPC 2445	N/A	USA	A.Y. Rossman	N/A	KX784582	N/A	KY653423	KX784714	KX784644	[45,54]
		CBS 114705	CMW 51215; CPC 2423	Fruit of *Annona reticulata*	Australia	D. Hutton	N/A	N/A	N/A	KY653424	KX784715	KX784645	[45,54]
B123	*C. xianrenensis*	CSF12909^T^	CGMCC3.19584	Soil (near *Eucalyptus* plantation)	Dacheng Town, Gaozhou County, Maoming Region, GuangDong, China	S.F. Chen, Q.C. Wang and W. Wang	N/A	MK962845	MK962857	N/A	MK962869	MK962833	[29]
		CSF12908	CGMCC3.19518	Soil (near *Eucalyptus* plantation)	Dacheng Town, Gaozhou County, Maoming Region, GuangDong, China	S.F. Chen, Q.C. Wang and W. Wang	N/A	MK962844	MK962856	N/A	MK962868	MK962832	[29]
	*C. minensis*	CSF9941^T^	CGMCC3.18877	Soil (*Eucalyptus* plantation)	XinLuo, LongYan, ShaoGuan, FuJian, China	S.F. Chen, Q.L. Liu and F.F. Liu	OK253121	OK253259	OK253403	OK253477	OK253814	OK253967	[32]
		CSF9975	CGMCC3.18881	Soil (*Eucalyptus* plantation)	LianChen, LongYan, ShaoGuan, FuJian, China	S.F. Chen, Q.L. Liu and F.F. Liu	OK253123	OK253261	OK253405	OK253479	OK253816	OK253969	[32]
	*C. shaoguanensis*	ZHKUCC21-0036^T^	–	*Callistemon rigidus*	ShaoGuan, GuangDong, China	Y.X. Zhang	N/A	MZ491112	N/A	N/A	MZ491134	MZ491156	[33]
		ZHKUCC21-0037	–	*Callistemon rigidus*	ShaoGuan, GuangDong, China	Y.X. Zhang	N/A	MZ491113	N/A	N/A	MZ491135	MZ491157	[33]
Outgroups													
	*Curvicladiella cignea*	CBS 109167^T^	CPC 1595; MUCL 40269	Decaying leaf	French Guiana	C. Decock	KM231122	KM231287	KM231461	KM232311	KM231867	KM232002	[18,53,58]
		CBS 109168	CPC 1594; MUCL 40268	Decaying seed	French Guiana	C. Decock	KM231121	KM231286	KM231460	KM232312	KM231868	KM232003	[18,53,58]

^a^ Codes (B1 to B120) of the 120 accepted *Calonectria* species resulting from Liu and co-authors [30]. ^b^ T: ex-type isolates of the species. ^c^ ATCC: American Type Culture Collection, Virginia, USA; CBS: Westerdijk Fungal Biodiversity Institute, Utrecht, The Netherlands; CERC: China Eucalypt Research Centre, ZhanJiang, GuangDong Province, China; CGMCC: China General Microbiological Culture Collection Center, Beijing, China; CMW: Culture collection of the Forestry and Agricultural Biotechnology Institute (FABI), University of Pretoria, Pretoria, South Africa; CPC: Pedro Crous working collection housed at Westerdijk Fungal Biodiversity Institute; CSF: Culture Collection from Southern Forests (CSF), ZhanJiang, GuangDong Province, China; IMI: IMI: International Mycological Institute, CABI Bioscience, Egham, Bakeham Lane, UK; MUCL: Mycotheque, Laboratoire de Mycologie Systematique st Appliqee, I’Universite, Louvian-la-Neuve, Belgium; PPRI: Plant Protection Research Institute, Pretoria, South Africa; STE-U: Department of Plant Pathology, University of Stellenbosch, South Africa; UFV: Universidade Federal de Viçsa, Viçsa, Brazil; ZHKUCC: The culture collection of Zhongkai University of Agriculture and Engineering; –: no other collection. ^d^ *act*: actin; *cmdA*: calmodulin; *his3*: histone H3; *rpb2*: the second largest subunit of RNA polymerase; *tef1*: translation elongation factor 1-alpha; *tub2*: β-tubulin. ^e^ N/A: information not available.

**Table 5:** Number of soil samples collected and *Calonectria* isolates obtained from plantations of three tree species in four provinces.

Province	*Eucalyptus urophylla* × *E*. *grandis*		*Pinus massoniana*		*Cunninghamia lanceolata*		All Three Tree Species	
	Number of Soil Sample	Number of Soil Sample Yielded *Calonectria*	Number of Soil Sample	Number of Soil Sample Yielded *Calonectria*	Number of Soil Sample	Number of Soil Sample Yielded *Calonectria*	Number of Soil Sample	Number of Soil Sample Yielded *Calonectria*
FuJian	250	217	248	164	250	91	748	472
GuangDong	250	213	250	218	244	44	744	475
GuangXi	250	182	249	46	250	21	749	249
YunNan	250	72	250	1	250	1	750	74
All four provinces	1000	684	997	429	994	157	2991	1270

**Table 6:** Number of isolates of each *Calonectria* species obtained from plantations of three tree species in four provinces.

	**FuJian**					**GuangDong**					**GuangXi**					**YunNan**			
	***E. urophylla* × *E. grandis***	***P. massoniana***		***C. lanceolata***		***E. urophylla* × *E. grandis***	***P. massoniana***		***C. lanceolata***		***E. urophylla* × *E. grandis***		***P. massoniana***	***C. lanceolata***		***E. urophylla* × *E. grandis***		***P. massoniana***	***C. lanceolata***
*C. aconidialis*	149	57		79		196	135		35		154		34	17		27		0	0
*C. kyotensis*	37	64		0		4	25		5		23		8	0		0		0	0
*C. hongkongensis*	27	39		6		12	50		0		2		1	0		0		0	0
*C. ilicicola*	3	4		4		1	2		3		3		3	4		5		0	0
*C. asiatica*	0	0		0		0	0		0		0		0	0		30		0	0
*C. eucalypti*	0	0		0		0	0		0		0		0	0		8		1	0
*C. curvispora*	0	0		0		0	4		0		0		0	0		0		0	0
*C. chinensis*	1	0		2		0	0		0		0		0	0		0		0	0
*C. pacifica*	0	0		0		0	2		1		0		0	0		0		0	0
*C. yunnanensis*	0	0		0		0	0		0		0		0	0		2		0	0
*C. canadiana*	0	0		0		0	0		0		0		0	0		0		0	1
All 11 *Calonectria* species	217	164		91		213	218		44		182		46	21		72		1	1
	***E. urophylla* × *E. grandis, P. massoniana and C. lanceolata***									**FuJian, GuangDong, GuangXi and YunNan**							**All three tree species in four provinces**		
	**FuJian**		**GuangDong**		**GuangXi**			**YunNan**		***E. urophylla* × *E. grandis***		***P. massoniana***			***C. lanceolata***				
*C. aconidialis*	285		366		205			27		526		226			131		883		
*C. kyotensis*	101		34		31			0		64		97			5		166		
*C. hongkongensis*	72		62		3			0		41		90			6		137		
*C. ilicicola*	11		6		10			5		12		9			11		32		
*C. asiatica*	0		0		0			30		30		0			0		30		
*C. eucalypti*	0		0		0			9		8		1			0		9		
*C. curvispora*	0		4		0			0		0		4			0		4		
*C. chinensis*	3		0		0			0		1		0			2		3		
*C. pacifica*	0		3		0			0		0		2			1		3		
*C. yunnanensis*	0		0		0			2		2		0			0		2		
*C. canadiana*	0		0		0			1		0		0			1		1		
All 11 *Calonectria* species	472		475		249			74		684		429			157		1270		

**Table 7:** Number of genotypes of each *Calonectria* species obtained from plantations of three tree species in four provinces, as determined by *tef1*-*tub2* gene sequences.

	**FuJian**					**GuangDong**					**GuangXi**					**YunNan**			
	***E. urophylla* × *E. grandis***	***P. massoniana***		***C. lanceolata***		***E. urophylla* × *E. grandis***	***P. massoniana***		***C. lanceolata***		***E. urophylla* × *E. grandis***		***P. massoniana***	***C. lanceolata***		***E. urophylla* × *E. grandis***		***P. massoniana***	***C. lanceolata***
*C. aconidialis*	7	5		7		10	10		5		7		2	1		2		0	0
*C. kyotensis*	14	20		0		4	16		4		9		6	0		0		0	0
*C. hongkongensis*	5	6		3		5	7		0		2		1	0		0		0	0
*C. ilicicola*	1	1		1		1	1		1		1		1	1		1		0	0
*C. asiatica*	0	0		0		0	0		0		0		0	0		2		0	0
*C. eucalypti*	0	0		0		0	0		0		0		0	0		4		1	0
*C. curvispora*	0	0		0		0	1		0		0		0	0		0		0	0
*C. chinensis*	1	0		2		0	0		0		0		0	0		0		0	0
*C. pacifica*	0	0		0		0	2		1		0		0	0		0		0	0
*C. yunnanensis*	0	0		0		0	0		0		0		0	0		2		0	0
*C. canadiana*	0	0		0		0	0		0		0		0	0		0		0	1
All 11 *Calonectria* species	28	32		13		20	37		11		19		10	2		11		1	1
	***E. urophylla* × *E. grandis*, *P. massoniana* and *C. lanceolata***									**FuJian, GuangDong, GuangXi and YunNan**							**All three tree species in four provinces**		
	**FuJian**		**GuangDong**		**GuangXi**			**YunNan**		***E. urophylla* × *E. grandis***		***P. massoniana***			***C. lanceolata***				
*C. aconidialis*	10		14		7			2		22		14			11		28		
*C. kyotensis*	24		19		11			0		24		33			4		41		
*C. hongkongensis*	8		7		3			0		8		8			3		10		
*C. ilicicola*	1		2		1			1		3		2			1		3		
*C. asiatica*	0		0		0			2		2		0			0		2		
*C. eucalypti*	0		0		0			4		4		1			0		4		
*C. curvispora*	0		1		0			0		0		1			0		1		
*C. chinensis*	2		0		0			0		1		0			2		2		
*C. pacifica*	0		3		0			0		0		2			1		3		
*C. yunnanensis*	0		0		0			2		2		0			0		2		
*C. canadiana*	0		0		0			1		0		0			1		1		
All 11 *Calonectria* species	45		46		22			12		66		61			23		97		

## Data Availability

Data are contained within the article.

## Appendix A. All 1270 *Calonectria* Isolates Obtained and Sequenced in This Study

**Table A1:** All 1270 *Calonectria* isolates obtained and sequenced in this study.

Site and Tree Species Code ^a^	Species Complex	Species	Genotype ^b^	Isolate No. ^c^	Sample and Isolate Information ^d^	GenBank Accession No. ^e^					
						*tef1*	*tub2*	*cmdA*	*his3*	*rpb2*	*act*
1. FuJian-Euc.	*C. kyotensis*	*C. aconidialis*	AA----	CSF22474	20210527-1-(1)	OQ188218	OQ260193	– ^f^	–	–	–
1. FuJian-Euc.	*C. kyotensis*	*C. aconidialis*	AA----	CSF22475	20210527-1-(2)	OQ188219	OQ260194	–	–	–	–
1. FuJian-Euc.	*C. kyotensis*	*C. aconidialis*	AA----	CSF22477	20210527-1-(6)	OQ188220	OQ260195	–	–	–	–
1. FuJian-Euc.	*C. kyotensis*	*C. aconidialis*	AA----	CSF22479	20210527-1-(8)	OQ188221	OQ260196	–	–	–	–
1. FuJian-Euc.	*C. kyotensis*	*C. aconidialis*	AA----	CSF22484	20210527-1-(13)	OQ188222	OQ260197	–	–	–	–
1. FuJian-Euc.	*C. kyotensis*	*C. aconidialis*	AA----	CSF22486	20210527-1-(15)	OQ188223	OQ260198	–	–	–	–
1. FuJian-Euc.	*C. kyotensis*	*C. aconidialis*	AA----	CSF22487	20210527-1-(16)	OQ188224	OQ260199	–	–	–	–
1. FuJian-Euc.	*C. kyotensis*	*C. aconidialis*	AA----	CSF22490	20210527-1-(19)	OQ188225	OQ260200	–	–	–	–
1. FuJian-Euc.	*C. kyotensis*	*C. aconidialis*	AA----	CSF22496	20210527-1-(25)	OQ188226	OQ260201	–	–	–	–
1. FuJian-Euc.	*C. kyotensis*	*C. aconidialis*	AA----	CSF22776	20210527-1-(27)	OQ188227	OQ260202	–	–	–	–
1. FuJian-Euc.	*C. kyotensis*	*C. aconidialis*	AA----	CSF22780	20210527-1-(31)	OQ188228	OQ260203	–	–	–	–
1. FuJian-Euc.	*C. kyotensis*	*C. aconidialis*	AA----	CSF22783	20210527-1-(35)	OQ188229	OQ260204	–	–	–	–
1. FuJian-Euc.	*C. kyotensis*	*C. aconidialis*	AA----	CSF22787	20210527-1-(40)	OQ188230	OQ260205	–	–	–	–
1. FuJian-Euc.	*C. kyotensis*	*C. aconidialis*	AA----	CSF22789	20210527-1-(42)	OQ188231	OQ260206	–	–	–	–
1. FuJian-Euc.	*C. kyotensis*	*C. aconidialis*	AA----	CSF22792	20210527-1-(47)	OQ188232	OQ260207	–	–	–	–
1. FuJian-Euc.	*C. kyotensis*	*C. aconidialis*	AA----	CSF22794	20210527-1-(50)	OQ188233	OQ260208	–	–	–	–
1. FuJian-Euc.	*C. kyotensis*	*C. aconidialis*	AA----	CSF22795	20210527-1-(51)	OQ188234	OQ260209	–	–	–	–
1. FuJian-Euc.	*C. kyotensis*	*C. aconidialis*	AA----	CSF22797	20210527-1-(53)	OQ188235	OQ260210	–	–	–	–
1. FuJian-Euc.	*C. kyotensis*	*C. aconidialis*	AA----	CSF22798	20210527-1-(55)	OQ188236	OQ260211	–	–	–	–
1. FuJian-Euc.	*C. kyotensis*	*C. aconidialis*	AA----	CSF22800	20210527-1-(57)	OQ188237	OQ260212	–	–	–	–
1. FuJian-Euc.	*C. kyotensis*	*C. aconidialis*	AA----	CSF22801	20210527-1-(58)	OQ188238	OQ260213	–	–	–	–
1. FuJian-Euc.	*C. kyotensis*	*C. aconidialis*	AA----	CSF22805	20210527-1-(62)	OQ188239	OQ260214	–	–	–	–
1. FuJian-Euc.	*C. kyotensis*	*C. aconidialis*	AA----	CSF22811	20210527-1-(68)	OQ188240	OQ260215	–	–	–	–
1. FuJian-Euc.	*C. kyotensis*	*C. aconidialis*	AA----	CSF22815	20210527-1-(72)	OQ188241	OQ260216	–	–	–	–
1. FuJian-Euc.	*C. kyotensis*	*C. aconidialis*	AA----	CSF22816	20210527-1-(73)	OQ188242	OQ260217	–	–	–	–
1. FuJian-Euc.	*C. kyotensis*	*C. aconidialis*	AA----	CSF22817	20210527-1-(74)	OQ188243	OQ260218	–	–	–	–
1. FuJian-Euc.	*C. kyotensis*	*C. aconidialis*	AA----	CSF22818	20210527-1-(75)	OQ188244	OQ260219	–	–	–	–
1. FuJian-Euc.	*C. kyotensis*	*C. aconidialis*	AA----	CSF22819	20210527-1-(77)	OQ188245	OQ260220	–	–	–	–
1. FuJian-Euc.	*C. kyotensis*	*C. aconidialis*	AA----	CSF22820	20210527-1-(78)	OQ188246	OQ260221	–	–	–	–
1. FuJian-Euc.	*C. kyotensis*	*C. aconidialis*	AA----	CSF22821	20210527-1-(79)	OQ188247	OQ260222	–	–	–	–
1. FuJian-Euc.	*C. kyotensis*	*C. aconidialis*	AA----	CSF22822	20210527-1-(80)	OQ188248	OQ260223	–	–	–	–
1. FuJian-Euc.	*C. kyotensis*	*C. aconidialis*	AA----	CSF22823	20210527-1-(81)	OQ188249	OQ260224	–	–	–	–
1. FuJian-Euc.	*C. kyotensis*	*C. aconidialis*	AA----	CSF22825	20210527-1-(83)	OQ188250	OQ260225	–	–	–	–
1. FuJian-Euc.	*C. kyotensis*	*C. aconidialis*	AA----	CSF22826	20210527-1-(84)	OQ188251	OQ260226	–	–	–	–
1. FuJian-Euc.	*C. kyotensis*	*C. aconidialis*	AA----	CSF22827	20210527-1-(85)	OQ188252	OQ260227	–	–	–	–
1. FuJian-Euc.	*C. kyotensis*	*C. aconidialis*	AA----	CSF22828	20210527-1-(87)	OQ188253	OQ260228	–	–	–	–
1. FuJian-Euc.	*C. kyotensis*	*C. aconidialis*	AA----	CSF22830	20210527-1-(89)	OQ188254	OQ260229	–	–	–	–
1. FuJian-Euc.	*C. kyotensis*	*C. aconidialis*	AA----	CSF22833	20210527-1-(92)	OQ188255	OQ260230	–	–	–	–
1. FuJian-Euc.	*C. kyotensis*	*C. aconidialis*	AA----	CSF22835	20210527-1-(94)	OQ188256	OQ260231	–	–	–	–
1. FuJian-Euc.	*C. kyotensis*	*C. aconidialis*	AA----	CSF22838	20210527-1-(97)	OQ188257	OQ260232	–	–	–	–
1. FuJian-Euc.	*C. kyotensis*	*C. aconidialis*	AA----	CSF22839	20210527-1-(98)	OQ188258	OQ260233	–	–	–	–
1. FuJian-Euc.	*C. kyotensis*	*C. aconidialis*	AA----	CSF22841	20210527-1-(100)	OQ188259	OQ260234	–	–	–	–
1. FuJian-Euc.	*C. kyotensis*	*C. aconidialis*	AA----	CSF22844	20210527-1-(102)	OQ188260	OQ260235	–	–	–	–
1. FuJian-Euc.	*C. kyotensis*	*C. aconidialis*	AA----	CSF22845	20210527-1-(103)	OQ188261	OQ260236	–	–	–	–
1. FuJian-Euc.	*C. kyotensis*	*C. aconidialis*	AA----	CSF22846	20210527-1-(105)	OQ188262	OQ260237	–	–	–	–
1. FuJian-Euc.	*C. kyotensis*	*C. aconidialis*	AA----	CSF22848	20210527-1-(107)	OQ188263	OQ260238	–	–	–	–
1. FuJian-Euc.	*C. kyotensis*	*C. aconidialis*	AA----	CSF22849	20210527-1-(108)	OQ188264	OQ260239	–	–	–	–
1. FuJian-Euc.	*C. kyotensis*	*C. aconidialis*	AA----	CSF22852	20210527-1-(111)	OQ188265	OQ260240	–	–	–	–
1. FuJian-Euc.	*C. kyotensis*	*C. aconidialis*	AA----	CSF22854	20210527-1-(113)	OQ188266	OQ260241	–	–	–	–
1. FuJian-Euc.	*C. kyotensis*	*C. aconidialis*	AA----	CSF22855	20210527-1-(114)	OQ188267	OQ260242	–	–	–	–
1. FuJian-Euc.	*C. kyotensis*	*C. aconidialis*	AA----	CSF22858	20210527-1-(117)	OQ188268	OQ260243	–	–	–	–
1. FuJian-Euc.	*C. kyotensis*	*C. aconidialis*	AA----	CSF22863	20210527-1-(122)	OQ188269	OQ260244	–	–	–	–
1. FuJian-Euc.	*C. kyotensis*	*C. aconidialis*	AA----	CSF22903	20210526-1-(7)	OQ188270	OQ260245	–	–	–	–
1. FuJian-Euc.	*C. kyotensis*	*C. aconidialis*	AA----	CSF22905	20210526-1-(10)	OQ188271	OQ260246	–	–	–	–
1. FuJian-Euc.	*C. kyotensis*	*C. aconidialis*	AA----	CSF22906	20210526-1-(11)	OQ188272	OQ260247	–	–	–	–
1. FuJian-Euc.	*C. kyotensis*	*C. aconidialis*	AA----	CSF22908	20210526-1-(13)	OQ188273	OQ260248	–	–	–	–
1. FuJian-Euc.	*C. kyotensis*	*C. aconidialis*	AA----	CSF22916	20210526-1-(21)	OQ188274	OQ260249	–	–	–	–
1. FuJian-Euc.	*C. kyotensis*	*C. aconidialis*	AA----	CSF22917	20210526-1-(22)	OQ188275	OQ260250	–	–	–	–
1. FuJian-Euc.	*C. kyotensis*	*C. aconidialis*	AA----	CSF22919	20210526-1-(24)	OQ188276	OQ260251	–	–	–	–
1. FuJian-Euc.	*C. kyotensis*	*C. aconidialis*	AA----	CSF22920	20210526-1-(25)	OQ188277	OQ260252	–	–	–	–
1. FuJian-Euc.	*C. kyotensis*	*C. aconidialis*	AA----	CSF22922	20210526-1-(27)	OQ188278	OQ260253	–	–	–	–
1. FuJian-Euc.	*C. kyotensis*	*C. aconidialis*	AA----	CSF22923	20210526-1-(28)	OQ188279	OQ260254	–	–	–	–
1. FuJian-Euc.	*C. kyotensis*	*C. aconidialis*	AA----	CSF22924	20210526-1-(29)	OQ188280	OQ260255	–	–	–	–
1. FuJian-Euc.	*C. kyotensis*	*C. aconidialis*	AA----	CSF22927	20210526-1-(32)	OQ188281	OQ260256	–	–	–	–
1. FuJian-Euc.	*C. kyotensis*	*C. aconidialis*	AA----	CSF22930	20210526-1-(36)	OQ188282	OQ260257	–	–	–	–
1. FuJian-Euc.	*C. kyotensis*	*C. aconidialis*	AA----	CSF22933	20210526-1-(40)	OQ188283	OQ260258	–	–	–	–
1. FuJian-Euc.	*C. kyotensis*	*C. aconidialis*	AA----	CSF22935	20210526-1-(42)	OQ188284	OQ260259	–	–	–	–
1. FuJian-Euc.	*C. kyotensis*	*C. aconidialis*	AA----	CSF22940	20210526-1-(47)	OQ188285	OQ260260	–	–	–	–
1. FuJian-Euc.	*C. kyotensis*	*C. aconidialis*	AA----	CSF22943	20210526-1-(50)	OQ188286	OQ260261	–	–	–	–
1. FuJian-Euc.	*C. kyotensis*	*C. aconidialis*	AA----	CSF22944	20210526-1-(51)	OQ188287	OQ260262	–	–	–	–
1. FuJian-Euc.	*C. kyotensis*	*C. aconidialis*	AA----	CSF22945	20210526-1-(53)	OQ188288	OQ260263	–	–	–	–
1. FuJian-Euc.	*C. kyotensis*	*C. aconidialis*	AA----	CSF22947	20210526-1-(56)	OQ188289	OQ260264	–	–	–	–
1. FuJian-Euc.	*C. kyotensis*	*C. aconidialis*	AA----	CSF22956	20210526-1-(66)	OQ188290	OQ260265	–	–	–	–
1. FuJian-Euc.	*C. kyotensis*	*C. aconidialis*	AA----	CSF22957	20210526-1-(67)	OQ188291	OQ260266	–	–	–	–
1. FuJian-Euc.	*C. kyotensis*	*C. aconidialis*	AA----	CSF22961	20210526-1-(72)	OQ188292	OQ260267	–	–	–	–
1. FuJian-Euc.	*C. kyotensis*	*C. aconidialis*	AA----	CSF22964	20210526-1-(77)	OQ188293	OQ260268	–	–	–	–
1. FuJian-Euc.	*C. kyotensis*	*C. aconidialis*	AA----	CSF22966	20210526-1-(79)	OQ188294	OQ260269	–	–	–	–
1. FuJian-Euc.	*C. kyotensis*	*C. aconidialis*	AA----	CSF22968	20210526-1-(82)	OQ188295	OQ260270	–	–	–	–
1. FuJian-Euc.	*C. kyotensis*	*C. aconidialis*	AA----	CSF22865	20210526-2-(2)	OQ188296	OQ260271	–	–	–	–
1. FuJian-Euc.	*C. kyotensis*	*C. aconidialis*	AA----	CSF22867	20210526-2-(5)	OQ188297	OQ260272	–	–	–	–
1. FuJian-Euc.	*C. kyotensis*	*C. aconidialis*	AA----	CSF22868	20210526-2-(6)	OQ188298	OQ260273	–	–	–	–
1. FuJian-Euc.	*C. kyotensis*	*C. aconidialis*	AA----	CSF22872	20210526-2-(10)	OQ188299	OQ260274	–	–	–	–
1. FuJian-Euc.	*C. kyotensis*	*C. aconidialis*	AA----	CSF22873	20210526-2-(12)	OQ188300	OQ260275	–	–	–	–
1. FuJian-Euc.	*C. kyotensis*	*C. aconidialis*	AA----	CSF22876	20210526-2-(17)	OQ188301	OQ260276	–	–	–	–
1. FuJian-Euc.	*C. kyotensis*	*C. aconidialis*	AA----	CSF22878	20210526-2-(21)	OQ188302	OQ260277	–	–	–	–
1. FuJian-Euc.	*C. kyotensis*	*C. aconidialis*	AA----	CSF22882	20210526-2-(25)	OQ188303	OQ260278	–	–	–	–
1. FuJian-Euc.	*C. kyotensis*	*C. aconidialis*	AA----	CSF22883	20210526-2-(26)	OQ188304	OQ260279	–	–	–	–
1. FuJian-Euc.	*C. kyotensis*	*C. aconidialis*	AA----	CSF22884	20210526-2-(27)	OQ188305	OQ260280	–	–	–	–
1. FuJian-Euc.	*C. kyotensis*	*C. aconidialis*	AA----	CSF22886	20210526-2-(30)	OQ188306	OQ260281	–	–	–	–
1. FuJian-Euc.	*C. kyotensis*	*C. aconidialis*	AA----	CSF22888	20210526-2-(34)	OQ188307	OQ260282	–	–	–	–
1. FuJian-Euc.	*C. kyotensis*	*C. aconidialis*	AA----	CSF22891	20210526-2-(37)	OQ188308	OQ260283	–	–	–	–
1. FuJian-Euc.	*C. kyotensis*	*C. aconidialis*	AA----	CSF22892	20210526-2-(38)	OQ188309	OQ260284	–	–	–	–
1. FuJian-Euc.	*C. kyotensis*	*C. aconidialis*	AD----	CSF22476	20210527-1-(3)	OQ188310	OQ260285	–	–	–	–
1. FuJian-Euc.	*C. kyotensis*	*C. aconidialis*	AD----	CSF22478	20210527-1-(7)	OQ188311	OQ260286	–	–	–	–
1. FuJian-Euc.	*C. kyotensis*	*C. aconidialis*	AD----	CSF22842	20210527-1-(101)	OQ188312	OQ260287	–	–	–	–
1. FuJian-Euc.	*C. kyotensis*	*C. aconidialis*	ADAAAA	CSF22962	20210526-1-(73)	OQ188313	OQ260288	OQ261468	OQ302903	OQ303110	OQ303316
1. FuJian-Euc.	*C. kyotensis*	*C. aconidialis*	AGAAAA	CSF22495	20210527-1-(24)	OQ188314	OQ260289	OQ261473	OQ302908	OQ303115	OQ303321
1. FuJian-Euc.	*C. kyotensis*	*C. aconidialis*	AI----	CSF22485	20210527-1-(14)	OQ188315	OQ260290	–	–	–	–
1. FuJian-Euc.	*C. kyotensis*	*C. aconidialis*	AI----	CSF22489	20210527-1-(18)	OQ188316	OQ260291	–	–	–	–
1. FuJian-Euc.	*C. kyotensis*	*C. aconidialis*	AI----	CSF22491	20210527-1-(20)	OQ188317	OQ260292	–	–	–	–
1. FuJian-Euc.	*C. kyotensis*	*C. aconidialis*	AI----	CSF22493	20210527-1-(22)	OQ188318	OQ260293	–	–	–	–
1. FuJian-Euc.	*C. kyotensis*	*C. aconidialis*	AI----	CSF22775	20210527-1-(26)	OQ188319	OQ260294	–	–	–	–
1. FuJian-Euc.	*C. kyotensis*	*C. aconidialis*	AI----	CSF22777	20210527-1-(28)	OQ188320	OQ260295	–	–	–	–
1. FuJian-Euc.	*C. kyotensis*	*C. aconidialis*	AI----	CSF22784	20210527-1-(37)	OQ188321	OQ260296	–	–	–	–
1. FuJian-Euc.	*C. kyotensis*	*C. aconidialis*	AI----	CSF22803	20210527-1-(60)	OQ188322	OQ260297	–	–	–	–
1. FuJian-Euc.	*C. kyotensis*	*C. aconidialis*	AI----	CSF22809	20210527-1-(66)	OQ188323	OQ260298	–	–	–	–
1. FuJian-Euc.	*C. kyotensis*	*C. aconidialis*	AI----	CSF22812	20210527-1-(69)	OQ188324	OQ260299	–	–	–	–
1. FuJian-Euc.	*C. kyotensis*	*C. aconidialis*	AI----	CSF22814	20210527-1-(71)	OQ188325	OQ260300	–	–	–	–
1. FuJian-Euc.	*C. kyotensis*	*C. aconidialis*	AI----	CSF22851	20210527-1-(110)	OQ188326	OQ260301	–	–	–	–
1. FuJian-Euc.	*C. kyotensis*	*C. aconidialis*	AI----	CSF22857	20210527-1-(116)	OQ188327	OQ260302	–	–	–	–
1. FuJian-Euc.	*C. kyotensis*	*C. aconidialis*	AI----	CSF22862	20210527-1-(121)	OQ188328	OQ260303	–	–	–	–
1. FuJian-Euc.	*C. kyotensis*	*C. aconidialis*	AI----	CSF22900	20210526-1-(4)	OQ188329	OQ260304	–	–	–	–
1. FuJian-Euc.	*C. kyotensis*	*C. aconidialis*	AI----	CSF22955	20210526-1-(65)	OQ188330	OQ260305	–	–	–	–
1. FuJian-Euc.	*C. kyotensis*	*C. aconidialis*	AI----	CSF22959	20210526-1-(69)	OQ188331	OQ260306	–	–	–	–
1. FuJian-Euc.	*C. kyotensis*	*C. aconidialis*	AI----	CSF22967	20210526-1-(80)	OQ188332	OQ260307	–	–	–	–
1. FuJian-Euc.	*C. kyotensis*	*C. aconidialis*	AI----	CSF22874	20210526-2-(13)	OQ188333	OQ260308	–	–	–	–
1. FuJian-Euc.	*C. kyotensis*	*C. aconidialis*	AU----	CSF22480	20210527-1-(9)	OQ188334	OQ260309	–	–	–	–
1. FuJian-Euc.	*C. kyotensis*	*C. aconidialis*	AU----	CSF22494	20210527-1-(23)	OQ188335	OQ260310	–	–	–	–
1. FuJian-Euc.	*C. kyotensis*	*C. aconidialis*	AU----	CSF22785	20210527-1-(38)	OQ188336	OQ260311	–	–	–	–
1. FuJian-Euc.	*C. kyotensis*	*C. aconidialis*	AU----	CSF22786	20210527-1-(39)	OQ188337	OQ260312	–	–	–	–
1. FuJian-Euc.	*C. kyotensis*	*C. aconidialis*	AU----	CSF22793	20210527-1-(49)	OQ188338	OQ260313	–	–	–	–
1. FuJian-Euc.	*C. kyotensis*	*C. aconidialis*	AU----	CSF22806	20210527-1-(63)	OQ188339	OQ260314	–	–	–	–
1. FuJian-Euc.	*C. kyotensis*	*C. aconidialis*	AU----	CSF22834	20210527-1-(93)	OQ188340	OQ260315	–	–	–	–
1. FuJian-Euc.	*C. kyotensis*	*C. aconidialis*	AU----	CSF22853	20210527-1-(112)	OQ188341	OQ260316	–	–	–	–
1. FuJian-Euc.	*C. kyotensis*	*C. aconidialis*	AU----	CSF22861	20210527-1-(120)	OQ188342	OQ260317	–	–	–	–
1. FuJian-Euc.	*C. kyotensis*	*C. aconidialis*	AU----	CSF22946	20210526-1-(55)	OQ188343	OQ260318	–	–	–	–
1. FuJian-Euc.	*C. kyotensis*	*C. aconidialis*	AU----	CSF22963	20210526-1-(74)	OQ188344	OQ260319	–	–	–	–
1. FuJian-Euc.	*C. kyotensis*	*C. aconidialis*	AU----	CSF22965	20210526-1-(78)	OQ188345	OQ260320	–	–	–	–
1. FuJian-Euc.	*C. kyotensis*	*C. aconidialis*	AU----	CSF22870	20210526-2-(8)	OQ188346	OQ260321	–	–	–	–
1. FuJian-Euc.	*C. kyotensis*	*C. aconidialis*	AU----	CSF22885	20210526-2-(29)	OQ188347	OQ260322	–	–	–	–
1. FuJian-Euc.	*C. kyotensis*	*C. aconidialis*	AU----	CSF22893	20210526-2-(40)	OQ188348	OQ260323	–	–	–	–
1. FuJian-Euc.	*C. kyotensis*	*C. aconidialis*	AUAAAA	CSF22813	20210527-1-(70)	OQ188349	OQ260324	OQ261495	OQ302930	OQ303137	OQ303343
1. FuJian-Euc.	*C. kyotensis*	*C. aconidialis*	CA----	CSF22781	20210527-1-(32)	OQ188350	OQ260325	–	–	–	–
1. FuJian-Euc.	*C. kyotensis*	*C. aconidialis*	CA----	CSF22788	20210527-1-(41)	OQ188351	OQ260326	–	–	–	–
1. FuJian-Euc.	*C. kyotensis*	*C. aconidialis*	CA----	CSF22796	20210527-1-(52)	OQ188352	OQ260327	–	–	–	–
1. FuJian-Euc.	*C. kyotensis*	*C. aconidialis*	CA----	CSF22824	20210527-1-(82)	OQ188353	OQ260328	–	–	–	–
1. FuJian-Euc.	*C. kyotensis*	*C. aconidialis*	CA----	CSF22832	20210527-1-(91)	OQ188354	OQ260329	–	–	–	–
1. FuJian-Euc.	*C. kyotensis*	*C. aconidialis*	CA----	CSF22836	20210527-1-(95)	OQ188355	OQ260330	–	–	–	–
1. FuJian-Euc.	*C. kyotensis*	*C. aconidialis*	CA----	CSF22847	20210527-1-(106)	OQ188356	OQ260331	–	–	–	–
1. FuJian-Euc.	*C. kyotensis*	*C. aconidialis*	CA----	CSF22856	20210527-1-(115)	OQ188357	OQ260332	–	–	–	–
1. FuJian-Euc.	*C. kyotensis*	*C. aconidialis*	CA----	CSF22860	20210527-1-(119)	OQ188358	OQ260333	–	–	–	–
1. FuJian-Euc.	*C. kyotensis*	*C. aconidialis*	CA----	CSF22910	20210526-1-(15)	OQ188359	OQ260334	–	–	–	–
1. FuJian-Euc.	*C. kyotensis*	*C. aconidialis*	CA----	CSF22941	20210526-1-(48)	OQ188360	OQ260335	–	–	–	–
1. FuJian-Euc.	*C. kyotensis*	*C. aconidialis*	CA----	CSF22887	20210526-2-(32)	OQ188361	OQ260336	–	–	–	–
1. FuJian-Euc.	*C. kyotensis*	*C. aconidialis*	CAAAAA	CSF22802	20210527-1-(59)	OQ188362	OQ260337	OQ261500	OQ302935	OQ303142	OQ303348
1. FuJian-Euc.	*C. kyotensis*	*C. aconidialis*	CAAAAA	CSF22912	20210526-1-(17)	OQ188363	OQ260338	OQ261498	OQ302933	OQ303140	OQ303346
1. FuJian-Euc.	*C. kyotensis*	*C. aconidialis*	CAAAAA	CSF22951	20210526-1-(60)	OQ188364	OQ260339	OQ261499	OQ302934	OQ303141	OQ303347
1. FuJian-Euc.	*C. kyotensis*	*C. aconidialis*	CADAAA	CSF22483	20210527-1-(12)	OQ188365	OQ260340	OQ261503	OQ302938	OQ303145	OQ303351
1. FuJian-Euc.	*C. kyotensis*	*C. aconidialis*	DADAAA	CSF22948	20210526-1-(57)	OQ188366	OQ260341	OQ261506	OQ302941	OQ303148	OQ303354
1. FuJian-Euc.	*C. kyotensis*	*C. chinensis*	AAAAAA	CSF22960	20210526-1-(70)	OQ188367	OQ260342	OQ261519	OQ302954	OQ303161	OQ303367
1. FuJian-Euc.	*C. kyotensis*	*C. hongkongensis*	AA----	CSF22779	20210527-1-(30)	OQ188368	OQ260343	–	–	–	–
1. FuJian-Euc.	*C. kyotensis*	*C. hongkongensis*	AA----	CSF22782	20210527-1-(33)	OQ188369	OQ260344	–	–	–	–
1. FuJian-Euc.	*C. kyotensis*	*C. hongkongensis*	AA----	CSF22807	20210527-1-(64)	OQ188370	OQ260345	–	–	–	–
1. FuJian-Euc.	*C. kyotensis*	*C. hongkongensis*	AA----	CSF22829	20210527-1-(88)	OQ188371	OQ260346	–	–	–	–
1. FuJian-Euc.	*C. kyotensis*	*C. hongkongensis*	AA----	CSF22837	20210527-1-(96)	OQ188372	OQ260347	–	–	–	–
1. FuJian-Euc.	*C. kyotensis*	*C. hongkongensis*	AA----	CSF22840	20210527-1-(99)	OQ188373	OQ260348	–	–	–	–
1. FuJian-Euc.	*C. kyotensis*	*C. hongkongensis*	AA----	CSF22859	20210527-1-(118)	OQ188374	OQ260349	–	–	–	–
1. FuJian-Euc.	*C. kyotensis*	*C. hongkongensis*	AA----	CSF22902	20210526-1-(6)	OQ188375	OQ260350	–	–	–	–
1. FuJian-Euc.	*C. kyotensis*	*C. hongkongensis*	AA----	CSF22925	20210526-1-(30)	OQ188376	OQ260351	–	–	–	–
1. FuJian-Euc.	*C. kyotensis*	*C. hongkongensis*	AA----	CSF22926	20210526-1-(31)	OQ188377	OQ260352	–	–	–	–
1. FuJian-Euc.	*C. kyotensis*	*C. hongkongensis*	AA----	CSF22929	20210526-1-(34)	OQ188378	OQ260353	–	–	–	–
1. FuJian-Euc.	*C. kyotensis*	*C. hongkongensis*	AA----	CSF22932	20210526-1-(39)	OQ188379	OQ260354	–	–	–	–
1. FuJian-Euc.	*C. kyotensis*	*C. hongkongensis*	AA----	CSF22939	20210526-1-(46)	OQ188380	OQ260355	–	–	–	–
1. FuJian-Euc.	*C. kyotensis*	*C. hongkongensis*	AA----	CSF22952	20210526-1-(62)	OQ188381	OQ260356	–	–	–	–
1. FuJian-Euc.	*C. kyotensis*	*C. hongkongensis*	AA----	CSF22953	20210526-1-(63)	OQ188382	OQ260357	–	–	–	–
1. FuJian-Euc.	*C. kyotensis*	*C. hongkongensis*	AA----	CSF22958	20210526-1-(68)	OQ188383	OQ260358	–	–	–	–
1. FuJian-Euc.	*C. kyotensis*	*C. hongkongensis*	AA----	CSF22875	20210526-2-(15)	OQ188384	OQ260359	–	–	–	–
1. FuJian-Euc.	*C. kyotensis*	*C. hongkongensis*	AA----	CSF22879	20210526-2-(22)	OQ188385	OQ260360	–	–	–	–
1. FuJian-Euc.	*C. kyotensis*	*C. hongkongensis*	AA----	CSF22881	20210526-2-(24)	OQ188386	OQ260361	–	–	–	–
1. FuJian-Euc.	*C. kyotensis*	*C. hongkongensis*	AA----	CSF22896	20210526-2-(45)	OQ188387	OQ260362	–	–	–	–
1. FuJian-Euc.	*C. kyotensis*	*C. hongkongensis*	AAAAAA	CSF22931	20210526-1-(38)	OQ188388	OQ260363	OQ261524	OQ302959	OQ303166	OQ303372
1. FuJian-Euc.	*C. kyotensis*	*C. hongkongensis*	ABAAAA	CSF22895	20210526-2-(43)	OQ188389	OQ260364	OQ261526	OQ302961	OQ303168	OQ303374
1. FuJian-Euc.	*C. kyotensis*	*C. hongkongensis*	AFAAAA	CSF22909	20210526-1-(14)	OQ188390	OQ260365	OQ261534	OQ302969	OQ303176	OQ303382
1. FuJian-Euc.	*C. kyotensis*	*C. hongkongensis*	AFAAAA	CSF22949	20210526-1-(58)	OQ188391	OQ260366	OQ261535	OQ302970	OQ303177	OQ303383
1. FuJian-Euc.	*C. kyotensis*	*C. hongkongensis*	AFBAAA	CSF22810	20210527-1-(67)	OQ188392	OQ260367	OQ261540	OQ302975	OQ303182	OQ303388
1. FuJian-Euc.	*C. kyotensis*	*C. hongkongensis*	AHAAAA	CSF22921	20210526-1-(26)	OQ188393	OQ260368	OQ261547	OQ302982	OQ303189	OQ303395
1. FuJian-Euc.	*C. kyotensis*	*C. hongkongensis*	AIAAAA	CSF22954	20210526-1-(64)	OQ188394	OQ260369	OQ261549	OQ302984	OQ303191	OQ303397
1. FuJian-Euc.	*C. kyotensis*	*C. ilicicola*	AA----	CSF22482	20210527-1-(11)	OQ188395	OQ260370	–	–	–	–
1. FuJian-Euc.	*C. kyotensis*	*C. ilicicola*	AA----	CSF22804	20210527-1-(61)	OQ188396	OQ260371	–	–	–	–
1. FuJian-Euc.	*C. kyotensis*	*C. ilicicola*	AA----	CSF22850	20210527-1-(109)	OQ188397	OQ260372	–	–	–	–
1. FuJian-Euc.	*C. kyotensis*	*C. kyotensis*	AA----	CSF22897	20210526-1-(1)	OQ188398	OQ260373	–	–	–	–
1. FuJian-Euc.	*C. kyotensis*	*C. kyotensis*	AAAAAA	CSF22937	20210526-1-(44)	OQ188399	OQ260374	OQ261558	OQ302993	OQ303199	OQ303406
1. FuJian-Euc.	*C. kyotensis*	*C. kyotensis*	ADAAAA	CSF22894	20210526-2-(42)	OQ188400	OQ260375	OQ261560	OQ302995	OQ303201	OQ303408
1. FuJian-Euc.	*C. kyotensis*	*C. kyotensis*	AF----	CSF22799	20210527-1-(56)	OQ188401	OQ260376	–	–	–	–
1. FuJian-Euc.	*C. kyotensis*	*C. kyotensis*	AF----	CSF22808	20210527-1-(65)	OQ188402	OQ260377	–	–	–	–
1. FuJian-Euc.	*C. kyotensis*	*C. kyotensis*	AF----	CSF22898	20210526-1-(2)	OQ188403	OQ260378	–	–	–	–
1. FuJian-Euc.	*C. kyotensis*	*C. kyotensis*	AF----	CSF22913	20210526-1-(18)	OQ188404	OQ260379	–	–	–	–
1. FuJian-Euc.	*C. kyotensis*	*C. kyotensis*	AFAAAA	CSF22869	20210526-2-(7)	OQ188405	OQ260380	OQ261564	OQ302999	OQ303205	OQ303412
1. FuJian-Euc.	*C. kyotensis*	*C. kyotensis*	AI----	CSF22914	20210526-1-(19)	OQ188406	OQ260381	–	–	–	–
1. FuJian-Euc.	*C. kyotensis*	*C. kyotensis*	AIAAAA	CSF22904	20210526-1-(8)	OQ188407	OQ260382	OQ261566	OQ303001	OQ303207	OQ303414
1. FuJian-Euc.	*C. kyotensis*	*C. kyotensis*	AIAAAA	CSF22866	20210526-2-(3)	OQ188408	OQ260383	OQ261567	OQ303002	OQ303208	OQ303415
1. FuJian-Euc.	*C. kyotensis*	*C. kyotensis*	ARAAAA	CSF22950	20210526-1-(59)	OQ188409	OQ260384	OQ261584	OQ303019	OQ303225	OQ303432
1. FuJian-Euc.	*C. kyotensis*	*C. kyotensis*	BRAAAA	CSF22889	20210526-2-(35)	OQ188410	OQ260385	OQ261586	OQ303021	OQ303227	OQ303434
1. FuJian-Euc.	*C. kyotensis*	*C. kyotensis*	CF----	CSF22901	20210526-1-(5)	OQ188411	OQ260386	–	–	–	–
1. FuJian-Euc.	*C. kyotensis*	*C. kyotensis*	CFAAAA	CSF22907	20210526-1-(12)	OQ188412	OQ260387	OQ261596	OQ303031	OQ303237	OQ303444
1. FuJian-Euc.	*C. kyotensis*	*C. kyotensis*	CMAAAA	CSF22778	20210527-1-(29)	OQ188413	OQ260388	OQ261600	OQ303035	OQ303241	OQ303448
1. FuJian-Euc.	*C. kyotensis*	*C. kyotensis*	DF----	CSF22481	20210527-1-(10)	OQ188414	OQ260389	–	–	–	–
1. FuJian-Euc.	*C. kyotensis*	*C. kyotensis*	DF----	CSF22488	20210527-1-(17)	OQ188415	OQ260390	–	–	–	–
1. FuJian-Euc.	*C. kyotensis*	*C. kyotensis*	DF----	CSF22911	20210526-1-(16)	OQ188416	OQ260391	–	–	–	–
1. FuJian-Euc.	*C. kyotensis*	*C. kyotensis*	DF----	CSF22915	20210526-1-(20)	OQ188417	OQ260392	–	–	–	–
1. FuJian-Euc.	*C. kyotensis*	*C. kyotensis*	DF----	CSF22934	20210526-1-(41)	OQ188418	OQ260393	–	–	–	–
1. FuJian-Euc.	*C. kyotensis*	*C. kyotensis*	DFAAAA	CSF22928	20210526-1-(33)	OQ188419	OQ260394	OQ261632	OQ303067	OQ303273	OQ303480
1. FuJian-Euc.	*C. kyotensis*	*C. kyotensis*	DI----	CSF22936	20210526-1-(43)	OQ188420	OQ260395	–	–	–	–
1. FuJian-Euc.	*C. kyotensis*	*C. kyotensis*	DI----	CSF22942	20210526-1-(49)	OQ188421	OQ260396	–	–	–	–
1. FuJian-Euc.	*C. kyotensis*	*C. kyotensis*	DI----	CSF22969	20210526-1-(83)	OQ188422	OQ260397	–	–	–	–
1. FuJian-Euc.	*C. kyotensis*	*C. kyotensis*	DK----	CSF22899	20210526-1-(3)	OQ188423	OQ260398	–	–	–	–
1. FuJian-Euc.	*C. kyotensis*	*C. kyotensis*	DK----	CSF22938	20210526-1-(45)	OQ188424	OQ260399	–	–	–	–
1. FuJian-Euc.	*C. kyotensis*	*C. kyotensis*	DK----	CSF22871	20210526-2-(9)	OQ188425	OQ260400	–	–	–	–
1. FuJian-Euc.	*C. kyotensis*	*C. kyotensis*	DK----	CSF22877	20210526-2-(20)	OQ188426	OQ260401	–	–	–	–
1. FuJian-Euc.	*C. kyotensis*	*C. kyotensis*	DKAAAA	CSF22790	20210527-1-(43)	OQ188427	OQ260402	OQ261639	OQ303074	OQ303280	OQ303487
1. FuJian-Euc.	*C. kyotensis*	*C. kyotensis*	DKAAAA	CSF22918	20210526-1-(23)	OQ188428	OQ260403	OQ261638	OQ303073	OQ303279	OQ303486
1. FuJian-Euc.	*C. kyotensis*	*C. kyotensis*	DKDAAA	CSF22890	20210526-2-(36)	OQ188429	OQ260404	OQ261642	OQ303077	OQ303283	OQ303490
1. FuJian-Euc.	*C. kyotensis*	*C. kyotensis*	DLCAAA	CSF22831	20210527-1-(90)	OQ188430	OQ260405	OQ261644	OQ303079	OQ303285	OQ303492
1. FuJian-Euc.	*C. kyotensis*	*C. kyotensis*	DM----	CSF22791	20210527-1-(44)	OQ188431	OQ260406	–	–	–	–
1. FuJian-Euc.	*C. kyotensis*	*C. kyotensis*	DM----	CSF22880	20210526-2-(23)	OQ188432	OQ260407	–	–	–	–
1. FuJian-Euc.	*C. kyotensis*	*C. kyotensis*	DMAAAA	CSF22864	20210526-2-(1)	OQ188433	OQ260408	OQ261645	OQ303080	OQ303286	OQ303493
1. FuJian-Euc.	*C. kyotensis*	*C. kyotensis*	DQAABA	CSF22492	20210527-1-(21)	OQ188434	OQ260409	OQ261651	OQ303086	OQ303292	OQ303499
2. FuJian-Pin.	*C. kyotensis*	*C. aconidialis*	AA----	CSF22510	20210524-1-(1)	OQ188435	OQ260410	–	–	–	–
2. FuJian-Pin.	*C. kyotensis*	*C. aconidialis*	AA----	CSF22512	20210524-1-(3)	OQ188436	OQ260411	–	–	–	–
2. FuJian-Pin.	*C. kyotensis*	*C. aconidialis*	AA----	CSF22517	20210524-1-(15)	OQ188437	OQ260412	–	–	–	–
2. FuJian-Pin.	*C. kyotensis*	*C. aconidialis*	AA----	CSF22518	20210524-1-(17)	OQ188438	OQ260413	–	–	–	–
2. FuJian-Pin.	*C. kyotensis*	*C. aconidialis*	AA----	CSF22522	20210524-1-(22)	OQ188439	OQ260414	–	–	–	–
2. FuJian-Pin.	*C. kyotensis*	*C. aconidialis*	AA----	CSF23059	20210524-1-(40)	OQ188440	OQ260415	–	–	–	–
2. FuJian-Pin.	*C. kyotensis*	*C. aconidialis*	AA----	CSF23064	20210524-1-(52)	OQ188441	OQ260416	–	–	–	–
2. FuJian-Pin.	*C. kyotensis*	*C. aconidialis*	AA----	CSF23067	20210524-1-(58)	OQ188442	OQ260417	–	–	–	–
2. FuJian-Pin.	*C. kyotensis*	*C. aconidialis*	AA----	CSF23069	20210524-1-(63)	OQ188443	OQ260418	–	–	–	–
2. FuJian-Pin.	*C. kyotensis*	*C. aconidialis*	AA----	CSF23074	20210524-1-(74)	OQ188444	OQ260419	–	–	–	–
2. FuJian-Pin.	*C. kyotensis*	*C. aconidialis*	AA----	CSF23075	20210524-1-(75)	OQ188445	OQ260420	–	–	–	–
2. FuJian-Pin.	*C. kyotensis*	*C. aconidialis*	AA----	CSF23078	20210524-1-(79)	OQ188446	OQ260421	–	–	–	–
2. FuJian-Pin.	*C. kyotensis*	*C. aconidialis*	AA----	CSF23082	20210524-1-(84)	OQ188447	OQ260422	–	–	–	–
2. FuJian-Pin.	*C. kyotensis*	*C. aconidialis*	AA----	CSF23088	20210524-1-(96)	OQ188448	OQ260423	–	–	–	–
2. FuJian-Pin.	*C. kyotensis*	*C. aconidialis*	AA----	CSF23089	20210524-1-(97)	OQ188449	OQ260424	–	–	–	–
2. FuJian-Pin.	*C. kyotensis*	*C. aconidialis*	AA----	CSF23091	20210524-1-(101)	OQ188450	OQ260425	–	–	–	–
2. FuJian-Pin.	*C. kyotensis*	*C. aconidialis*	AA----	CSF23095	20210524-1-(107)	OQ188451	OQ260426	–	–	–	–
2. FuJian-Pin.	*C. kyotensis*	*C. aconidialis*	AA----	CSF23097	20210524-1-(110)	OQ188452	OQ260427	–	–	–	–
2. FuJian-Pin.	*C. kyotensis*	*C. aconidialis*	AA----	CSF23108	20210524-1-(128)	OQ188453	OQ260428	–	–	–	–
2. FuJian-Pin.	*C. kyotensis*	*C. aconidialis*	AA----	CSF23121	20210524-1-(143)	OQ188454	OQ260429	–	–	–	–
2. FuJian-Pin.	*C. kyotensis*	*C. aconidialis*	AA----	CSF23126	20210524-1-(150)	OQ188455	OQ260430	–	–	–	–
2. FuJian-Pin.	*C. kyotensis*	*C. aconidialis*	AA----	CSF23130	20210524-1-(154)	OQ188456	OQ260431	–	–	–	–
2. FuJian-Pin.	*C. kyotensis*	*C. aconidialis*	AA----	CSF23131	20210524-1-(155)	OQ188457	OQ260432	–	–	–	–
2. FuJian-Pin.	*C. kyotensis*	*C. aconidialis*	AA----	CSF23149	20210524-1-(181)	OQ188458	OQ260433	–	–	–	–
2. FuJian-Pin.	*C. kyotensis*	*C. aconidialis*	AA----	CSF23151	20210524-1-(183)	OQ188459	OQ260434	–	–	–	–
2. FuJian-Pin.	*C. kyotensis*	*C. aconidialis*	AA----	CSF23159	20210524-1-(192)	OQ188460	OQ260435	–	–	–	–
2. FuJian-Pin.	*C. kyotensis*	*C. aconidialis*	AA----	CSF23168	20210524-1-(204)	OQ188461	OQ260436	–	–	–	–
2. FuJian-Pin.	*C. kyotensis*	*C. aconidialis*	AA----	CSF23172	20210524-1-(209)	OQ188462	OQ260437	–	–	–	–
2. FuJian-Pin.	*C. kyotensis*	*C. aconidialis*	AA----	CSF23178	20210524-1-(218)	OQ188463	OQ260438	–	–	–	–
2. FuJian-Pin.	*C. kyotensis*	*C. aconidialis*	AA----	CSF23179	20210524-1-(219)	OQ188464	OQ260439	–	–	–	–
2. FuJian-Pin.	*C. kyotensis*	*C. aconidialis*	AA----	CSF23182	20210524-1-(223)	OQ188465	OQ260440	–	–	–	–
2. FuJian-Pin.	*C. kyotensis*	*C. aconidialis*	AA----	CSF23183	20210524-1-(224)	OQ188466	OQ260441	–	–	–	–
2. FuJian-Pin.	*C. kyotensis*	*C. aconidialis*	AA----	CSF23184	20210524-1-(226)	OQ188467	OQ260442	–	–	–	–
2. FuJian-Pin.	*C. kyotensis*	*C. aconidialis*	AA----	CSF23185	20210524-1-(227)	OQ188468	OQ260443	–	–	–	–
2. FuJian-Pin.	*C. kyotensis*	*C. aconidialis*	AA----	CSF23188	20210524-1-(230)	OQ188469	OQ260444	–	–	–	–
2. FuJian-Pin.	*C. kyotensis*	*C. aconidialis*	AA----	CSF23190	20210524-1-(232)	OQ188470	OQ260445	–	–	–	–
2. FuJian-Pin.	*C. kyotensis*	*C. aconidialis*	AA----	CSF23198	20210524-1-(245)	OQ188471	OQ260446	–	–	–	–
2. FuJian-Pin.	*C. kyotensis*	*C. aconidialis*	AI----	CSF23076	20210524-1-(76)	OQ188472	OQ260447	–	–	–	–
2. FuJian-Pin.	*C. kyotensis*	*C. aconidialis*	AI----	CSF23081	20210524-1-(83)	OQ188473	OQ260448	–	–	–	–
2. FuJian-Pin.	*C. kyotensis*	*C. aconidialis*	AI----	CSF23125	20210524-1-(148)	OQ188474	OQ260449	–	–	–	–
2. FuJian-Pin.	*C. kyotensis*	*C. aconidialis*	AI----	CSF23144	20210524-1-(174)	OQ188475	OQ260450	–	–	–	–
2. FuJian-Pin.	*C. kyotensis*	*C. aconidialis*	AIAAAA	CSF23113	20210524-1-(135)	OQ188476	OQ260451	OQ261476	OQ302911	OQ303118	OQ303324
2. FuJian-Pin.	*C. kyotensis*	*C. aconidialis*	APAAAA	CSF23133	20210524-1-(158)	OQ188477	OQ260452	OQ261487	OQ302922	OQ303129	OQ303335
2. FuJian-Pin.	*C. kyotensis*	*C. aconidialis*	AU----	CSF22514	20210524-1-(7)	OQ188478	OQ260453	–	–	–	–
2. FuJian-Pin.	*C. kyotensis*	*C. aconidialis*	AU----	CSF23093	20210524-1-(103)	OQ188479	OQ260454	–	–	–	–
2. FuJian-Pin.	*C. kyotensis*	*C. aconidialis*	AU----	CSF23134	20210524-1-(159)	OQ188480	OQ260455	–	–	–	–
2. FuJian-Pin.	*C. kyotensis*	*C. aconidialis*	AU----	CSF23150	20210524-1-(182)	OQ188481	OQ260456	–	–	–	–
2. FuJian-Pin.	*C. kyotensis*	*C. aconidialis*	AU----	CSF23153	20210524-1-(185)	OQ188482	OQ260457	–	–	–	–
2. FuJian-Pin.	*C. kyotensis*	*C. aconidialis*	AU----	CSF23157	20210524-1-(189)	OQ188483	OQ260458	–	–	–	–
2. FuJian-Pin.	*C. kyotensis*	*C. aconidialis*	AU----	CSF23200	20210524-1-(247)	OQ188484	OQ260459	–	–	–	–
2. FuJian-Pin.	*C. kyotensis*	*C. aconidialis*	CA----	CSF22511	20210524-1-(2)	OQ188485	OQ260460	–	–	–	–
2. FuJian-Pin.	*C. kyotensis*	*C. aconidialis*	CA----	CSF23062	20210524-1-(49)	OQ188486	OQ260461	–	–	–	–
2. FuJian-Pin.	*C. kyotensis*	*C. aconidialis*	CA----	CSF23107	20210524-1-(127)	OQ188487	OQ260462	–	–	–	–
2. FuJian-Pin.	*C. kyotensis*	*C. aconidialis*	CA----	CSF23146	20210524-1-(176)	OQ188488	OQ260463	–	–	–	–
2. FuJian-Pin.	*C. kyotensis*	*C. aconidialis*	CA----	CSF23167	20210524-1-(201)	OQ188489	OQ260464	–	–	–	–
2. FuJian-Pin.	*C. kyotensis*	*C. aconidialis*	CABAAA	CSF23196	20210524-1-(243)	OQ188490	OQ260465	OQ261502	OQ302937	OQ303144	OQ303350
2. FuJian-Pin.	*C. kyotensis*	*C. aconidialis*	CADAAA	CSF23147	20210524-1-(177)	OQ188491	OQ260466	OQ261504	OQ302939	OQ303146	OQ303352
2. FuJian-Pin.	*C. kyotensis*	*C. hongkongensis*	AA----	CSF22515	20210524-1-(8)	OQ188492	OQ260467	–	–	–	–
2. FuJian-Pin.	*C. kyotensis*	*C. hongkongensis*	AA----	CSF22521	20210524-1-(21)	OQ188493	OQ260468	–	–	–	–
2. FuJian-Pin.	*C. kyotensis*	*C. hongkongensis*	AA----	CSF23053	20210524-1-(30)	OQ188494	OQ260469	–	–	–	–
2. FuJian-Pin.	*C. kyotensis*	*C. hongkongensis*	AA----	CSF23060	20210524-1-(42)	OQ188495	OQ260470	–	–	–	–
2. FuJian-Pin.	*C. kyotensis*	*C. hongkongensis*	AA----	CSF23061	20210524-1-(46)	OQ188496	OQ260471	–	–	–	–
2. FuJian-Pin.	*C. kyotensis*	*C. hongkongensis*	AA----	CSF23063	20210524-1-(50)	OQ188497	OQ260472	–	–	–	–
2. FuJian-Pin.	*C. kyotensis*	*C. hongkongensis*	AA----	CSF23066	20210524-1-(56)	OQ188498	OQ260473	–	–	–	–
2. FuJian-Pin.	*C. kyotensis*	*C. hongkongensis*	AA----	CSF23071	20210524-1-(69)	OQ188499	OQ260474	–	–	–	–
2. FuJian-Pin.	*C. kyotensis*	*C. hongkongensis*	AA----	CSF23085	20210524-1-(92)	OQ188500	OQ260475	–	–	–	–
2. FuJian-Pin.	*C. kyotensis*	*C. hongkongensis*	AA----	CSF23090	20210524-1-(99)	OQ188501	OQ260476	–	–	–	–
2. FuJian-Pin.	*C. kyotensis*	*C. hongkongensis*	AA----	CSF23092	20210524-1-(102)	OQ188502	OQ260477	–	–	–	–
2. FuJian-Pin.	*C. kyotensis*	*C. hongkongensis*	AA----	CSF23101	20210524-1-(117)	OQ188503	OQ260478	–	–	–	–
2. FuJian-Pin.	*C. kyotensis*	*C. hongkongensis*	AA----	CSF23102	20210524-1-(118)	OQ188504	OQ260479	–	–	–	–
2. FuJian-Pin.	*C. kyotensis*	*C. hongkongensis*	AA----	CSF23116	20210524-1-(138)	OQ188505	OQ260480	–	–	–	–
2. FuJian-Pin.	*C. kyotensis*	*C. hongkongensis*	AA----	CSF23119	20210524-1-(141)	OQ188506	OQ260481	–	–	–	–
2. FuJian-Pin.	*C. kyotensis*	*C. hongkongensis*	AA----	CSF23122	20210524-1-(144)	OQ188507	OQ260482	–	–	–	–
2. FuJian-Pin.	*C. kyotensis*	*C. hongkongensis*	AA----	CSF23128	20210524-1-(152)	OQ188508	OQ260483	–	–	–	–
2. FuJian-Pin.	*C. kyotensis*	*C. hongkongensis*	AA----	CSF23129	20210524-1-(153)	OQ188509	OQ260484	–	–	–	–
2. FuJian-Pin.	*C. kyotensis*	*C. hongkongensis*	AA----	CSF23155	20210524-1-(187)	OQ188510	OQ260485	–	–	–	–
2. FuJian-Pin.	*C. kyotensis*	*C. hongkongensis*	AA----	CSF23158	20210524-1-(191)	OQ188511	OQ260486	–	–	–	–
2. FuJian-Pin.	*C. kyotensis*	*C. hongkongensis*	AA----	CSF23162	20210524-1-(196)	OQ188512	OQ260487	–	–	–	–
2. FuJian-Pin.	*C. kyotensis*	*C. hongkongensis*	AA----	CSF23169	20210524-1-(205)	OQ188513	OQ260488	–	–	–	–
2. FuJian-Pin.	*C. kyotensis*	*C. hongkongensis*	AA----	CSF23173	20210524-1-(210)	OQ188514	OQ260489	–	–	–	–
2. FuJian-Pin.	*C. kyotensis*	*C. hongkongensis*	AA----	CSF23186	20210524-1-(228)	OQ188515	OQ260490	–	–	–	–
2. FuJian-Pin.	*C. kyotensis*	*C. hongkongensis*	AA----	CSF23192	20210524-1-(234)	OQ188516	OQ260491	–	–	–	–
2. FuJian-Pin.	*C. kyotensis*	*C. hongkongensis*	AA----	CSF23197	20210524-1-(244)	OQ188517	OQ260492	–	–	–	–
2. FuJian-Pin.	*C. kyotensis*	*C. hongkongensis*	AB----	CSF23055	20210524-1-(32)	OQ188518	OQ260493	–	–	–	–
2. FuJian-Pin.	*C. kyotensis*	*C. hongkongensis*	AB----	CSF23164	20210524-1-(198)	OQ188519	OQ260494	–	–	–	–
2. FuJian-Pin.	*C. kyotensis*	*C. hongkongensis*	AD----	CSF23072	20210524-1-(70)	OQ188520	OQ260495	–	–	–	–
2. FuJian-Pin.	*C. kyotensis*	*C. hongkongensis*	AEBAAA	CSF23136	20210524-1-(161)	OQ188521	OQ260496	OQ261532	OQ302967	OQ303174	OQ303380
2. FuJian-Pin.	*C. kyotensis*	*C. hongkongensis*	AF----	CSF23050	20210524-1-(26)	OQ188522	OQ260497	–	–	–	–
2. FuJian-Pin.	*C. kyotensis*	*C. hongkongensis*	AF----	CSF23080	20210524-1-(82)	OQ188523	OQ260498	–	–	–	–
2. FuJian-Pin.	*C. kyotensis*	*C. hongkongensis*	AF----	CSF23156	20210524-1-(188)	OQ188524	OQ260499	–	–	–	–
2. FuJian-Pin.	*C. kyotensis*	*C. hongkongensis*	AF----	CSF23171	20210524-1-(208)	OQ188525	OQ260500	–	–	–	–
2. FuJian-Pin.	*C. kyotensis*	*C. hongkongensis*	AF----	CSF23180	20210524-1-(221)	OQ188526	OQ260501	–	–	–	–
2. FuJian-Pin.	*C. kyotensis*	*C. hongkongensis*	AFAAAA	CSF23068	20210524-1-(59)	OQ188527	OQ260502	OQ261536	OQ302971	OQ303178	OQ303384
2. FuJian-Pin.	*C. kyotensis*	*C. hongkongensis*	AFBAAA	CSF23142	20210524-1-(168)	OQ188528	OQ260503	OQ261541	OQ302976	OQ303183	OQ303389
2. FuJian-Pin.	*C. kyotensis*	*C. hongkongensis*	AGBAAA	CSF23137	20210524-1-(162)	OQ188529	OQ260504	OQ261544	OQ302979	OQ303186	OQ303392
2. FuJian-Pin.	*C. kyotensis*	*C. hongkongensis*	AGBAAA	CSF23166	20210524-1-(200)	OQ188530	OQ260505	OQ261545	OQ302980	OQ303187	OQ303393
2. FuJian-Pin.	*C. kyotensis*	*C. ilicicola*	AA----	CSF23083	20210524-1-(87)	OQ188531	OQ260506	–	–	–	–
2. FuJian-Pin.	*C. kyotensis*	*C. ilicicola*	AA----	CSF23127	20210524-1-(151)	OQ188532	OQ260507	–	–	–	–
2. FuJian-Pin.	*C. kyotensis*	*C. ilicicola*	AA----	CSF23195	20210524-1-(241)	OQ188533	OQ260508	–	–	–	–
2. FuJian-Pin.	*C. kyotensis*	*C. ilicicola*	AABAAA	CSF23189	20210524-1-(231)	OQ188534	OQ260509	OQ261553	OQ302988	OQ303194	OQ303401
2. FuJian-Pin.	*C. kyotensis*	*C. kyotensis*	AA----	CSF23138	20210524-1-(163)	OQ188535	OQ260510	–	–	–	–
2. FuJian-Pin.	*C. kyotensis*	*C. kyotensis*	AAAAAA	CSF23086	20210524-1-(93)	OQ188536	OQ260511	OQ261559	OQ302994	OQ303200	OQ303407
2. FuJian-Pin.	*C. kyotensis*	*C. kyotensis*	ADAAAA	CSF23115	20210524-1-(137)	OQ188537	OQ260512	OQ261561	OQ302996	OQ303202	OQ303409
2. FuJian-Pin.	*C. kyotensis*	*C. kyotensis*	ADAAAA	CSF23120	20210524-1-(142)	OQ188538	OQ260513	OQ261562	OQ302997	OQ303203	OQ303410
2. FuJian-Pin.	*C. kyotensis*	*C. kyotensis*	AF----	CSF23099	20210524-1-(115)	OQ188539	OQ260514	–	–	–	–
2. FuJian-Pin.	*C. kyotensis*	*C. kyotensis*	AF----	CSF23105	20210524-1-(124)	OQ188540	OQ260515	–	–	–	–
2. FuJian-Pin.	*C. kyotensis*	*C. kyotensis*	AF----	CSF23170	20210524-1-(206)	OQ188541	OQ260516	–	–	–	–
2. FuJian-Pin.	*C. kyotensis*	*C. kyotensis*	AFAAAA	CSF23163	20210524-1-(197)	OQ188542	OQ260517	OQ261565	OQ303000	OQ303206	OQ303413
2. FuJian-Pin.	*C. kyotensis*	*C. kyotensis*	AI----	CSF23057	20210524-1-(35)	OQ188543	OQ260518	–	–	–	–
2. FuJian-Pin.	*C. kyotensis*	*C. kyotensis*	AI----	CSF23103	20210524-1-(122)	OQ188544	OQ260519	–	–	–	–
2. FuJian-Pin.	*C. kyotensis*	*C. kyotensis*	AI----	CSF23117	20210524-1-(139)	OQ188545	OQ260520	–	–	–	–
2. FuJian-Pin.	*C. kyotensis*	*C. kyotensis*	AI----	CSF23123	20210524-1-(145)	OQ188546	OQ260521	–	–	–	–
2. FuJian-Pin.	*C. kyotensis*	*C. kyotensis*	AI----	CSF23199	20210524-1-(246)	OQ188547	OQ260522	–	–	–	–
2. FuJian-Pin.	*C. kyotensis*	*C. kyotensis*	AIDABA	CSF23104	20210524-1-(123)	OQ188548	OQ260523	OQ261571	OQ303006	OQ303212	OQ303419
2. FuJian-Pin.	*C. kyotensis*	*C. kyotensis*	AIFAAA	CSF23181	20210524-1-(222)	OQ188549	OQ260524	OQ261572	OQ303007	OQ303213	OQ303420
2. FuJian-Pin.	*C. kyotensis*	*C. kyotensis*	AKAAAA	CSF23070	20210524-1-(68)	OQ188550	OQ260525	OQ261573	OQ303008	OQ303214	OQ303421
2. FuJian-Pin.	*C. kyotensis*	*C. kyotensis*	AKAAAA	CSF23096	20210524-1-(108)	OQ188551	OQ260526	OQ261574	OQ303009	OQ303215	OQ303422
2. FuJian-Pin.	*C. kyotensis*	*C. kyotensis*	AL----	CSF23132	20210524-1-(157)	OQ188552	OQ260527	–	–	–	–
2. FuJian-Pin.	*C. kyotensis*	*C. kyotensis*	AL----	CSF23194	20210524-1-(240)	OQ188553	OQ260528	–	–	–	–
2. FuJian-Pin.	*C. kyotensis*	*C. kyotensis*	ALABAA	CSF23098	20210524-1-(112)	OQ188554	OQ260529	OQ261575	OQ303010	OQ303216	OQ303423
2. FuJian-Pin.	*C. kyotensis*	*C. kyotensis*	ALBAAA	CSF22516	20210524-1-(14)	OQ188555	OQ260530	OQ261576	OQ303011	OQ303217	OQ303424
2. FuJian-Pin.	*C. kyotensis*	*C. kyotensis*	AOAAAA	CSF23094	20210524-1-(104)	OQ188556	OQ260531	OQ261577	OQ303012	OQ303218	OQ303425
2. FuJian-Pin.	*C. kyotensis*	*C. kyotensis*	CBAAAA	CSF23110	20210524-1-(132)	OQ188557	OQ260532	OQ261593	OQ303028	OQ303234	OQ303441
2. FuJian-Pin.	*C. kyotensis*	*C. kyotensis*	CF----	CSF23112	20210524-1-(134)	OQ188558	OQ260533	–	–	–	–
2. FuJian-Pin.	*C. kyotensis*	*C. kyotensis*	CFAAAA	CSF23114	20210524-1-(136)	OQ188559	OQ260534	OQ261597	OQ303032	OQ303238	OQ303445
2. FuJian-Pin.	*C. kyotensis*	*C. kyotensis*	CIAAAA	CSF23176	20210524-1-(214)	OQ188560	OQ260535	OQ261599	OQ303034	OQ303240	OQ303447
2. FuJian-Pin.	*C. kyotensis*	*C. kyotensis*	CODAAB	CSF23124	20210524-1-(146)	OQ188561	OQ260536	OQ261611	OQ303046	OQ303252	OQ303459
2. FuJian-Pin.	*C. kyotensis*	*C. kyotensis*	DA----	CSF22513	20210524-1-(5)	OQ188562	OQ260537	–	–	–	–
2. FuJian-Pin.	*C. kyotensis*	*C. kyotensis*	DA----	CSF23073	20210524-1-(71)	OQ188563	OQ260538	–	–	–	–
2. FuJian-Pin.	*C. kyotensis*	*C. kyotensis*	DA----	CSF23160	20210524-1-(193)	OQ188564	OQ260539	–	–	–	–
2. FuJian-Pin.	*C. kyotensis*	*C. kyotensis*	DA----	CSF23177	20210524-1-(216)	OQ188565	OQ260540	–	–	–	–
2. FuJian-Pin.	*C. kyotensis*	*C. kyotensis*	DAAAAA	CSF23143	20210524-1-(169)	OQ188566	OQ260541	OQ261624	OQ303059	OQ303265	OQ303472
2. FuJian-Pin.	*C. kyotensis*	*C. kyotensis*	DD----	CSF23077	20210524-1-(78)	OQ188567	OQ260542	–	–	–	–
2. FuJian-Pin.	*C. kyotensis*	*C. kyotensis*	DD----	CSF23161	20210524-1-(194)	OQ188568	OQ260543	–	–	–	–
2. FuJian-Pin.	*C. kyotensis*	*C. kyotensis*	DDAABA	CSF23118	20210524-1-(140)	OQ188569	OQ260544	OQ261627	OQ303062	OQ303268	OQ303475
2. FuJian-Pin.	*C. kyotensis*	*C. kyotensis*	DDDAAA	CSF23145	20210524-1-(175)	OQ188570	OQ260545	OQ261629	OQ303064	OQ303270	OQ303477
2. FuJian-Pin.	*C. kyotensis*	*C. kyotensis*	DF----	CSF23079	20210524-1-(81)	OQ188571	OQ260546	–	–	–	–
2. FuJian-Pin.	*C. kyotensis*	*C. kyotensis*	DF----	CSF23084	20210524-1-(91)	OQ188572	OQ260547	–	–	–	–
2. FuJian-Pin.	*C. kyotensis*	*C. kyotensis*	DF----	CSF23148	20210524-1-(178)	OQ188573	OQ260548	–	–	–	–
2. FuJian-Pin.	*C. kyotensis*	*C. kyotensis*	DF----	CSF23152	20210524-1-(184)	OQ188574	OQ260549	–	–	–	–
2. FuJian-Pin.	*C. kyotensis*	*C. kyotensis*	DF----	CSF23175	20210524-1-(213)	OQ188575	OQ260550	–	–	–	–
2. FuJian-Pin.	*C. kyotensis*	*C. kyotensis*	DF----	CSF23187	20210524-1-(229)	OQ188576	OQ260551	–	–	–	–
2. FuJian-Pin.	*C. kyotensis*	*C. kyotensis*	DFBAAA	CSF23174	20210524-1-(211)	OQ188577	OQ260552	OQ261633	OQ303068	OQ303274	OQ303481
2. FuJian-Pin.	*C. kyotensis*	*C. kyotensis*	DI----	CSF22520	20210524-1-(20)	OQ188578	OQ260553	–	–	–	–
2. FuJian-Pin.	*C. kyotensis*	*C. kyotensis*	DI----	CSF23056	20210524-1-(33)	OQ188579	OQ260554	–	–	–	–
2. FuJian-Pin.	*C. kyotensis*	*C. kyotensis*	DI----	CSF23111	20210524-1-(133)	OQ188580	OQ260555	–	–	–	–
2. FuJian-Pin.	*C. kyotensis*	*C. kyotensis*	DI----	CSF23135	20210524-1-(160)	OQ188581	OQ260556	–	–	–	–
2. FuJian-Pin.	*C. kyotensis*	*C. kyotensis*	DI----	CSF23140	20210524-1-(165)	OQ188582	OQ260557	–	–	–	–
2. FuJian-Pin.	*C. kyotensis*	*C. kyotensis*	DI----	CSF23141	20210524-1-(166)	OQ188583	OQ260558	–	–	–	–
2. FuJian-Pin.	*C. kyotensis*	*C. kyotensis*	DI----	CSF23191	20210524-1-(233)	OQ188584	OQ260559	–	–	–	–
2. FuJian-Pin.	*C. kyotensis*	*C. kyotensis*	DIAAAA	CSF23052	20210524-1-(28)	OQ188585	OQ260560	OQ261635	OQ303070	OQ303276	OQ303483
2. FuJian-Pin.	*C. kyotensis*	*C. kyotensis*	DJBAAA	CSF23054	20210524-1-(31)	OQ188586	OQ260561	OQ261637	OQ303072	OQ303278	OQ303485
2. FuJian-Pin.	*C. kyotensis*	*C. kyotensis*	DK----	CSF23106	20210524-1-(126)	OQ188587	OQ260562	–	–	–	–
2. FuJian-Pin.	*C. kyotensis*	*C. kyotensis*	DKAAAA	CSF23109	20210524-1-(131)	OQ188588	OQ260563	OQ261640	OQ303075	OQ303281	OQ303488
2. FuJian-Pin.	*C. kyotensis*	*C. kyotensis*	DKAAAA	CSF23165	20210524-1-(199)	OQ188589	OQ260564	OQ261641	OQ303076	OQ303282	OQ303489
2. FuJian-Pin.	*C. kyotensis*	*C. kyotensis*	DL----	CSF23058	20210524-1-(37)	OQ188590	OQ260565	–	–	–	–
2. FuJian-Pin.	*C. kyotensis*	*C. kyotensis*	DL----	CSF23139	20210524-1-(164)	OQ188591	OQ260566	–	–	–	–
2. FuJian-Pin.	*C. kyotensis*	*C. kyotensis*	DM----	CSF22523	20210524-1-(24)	OQ188592	OQ260567	–	–	–	–
2. FuJian-Pin.	*C. kyotensis*	*C. kyotensis*	DM----	CSF23051	20210524-1-(27)	OQ188593	OQ260568	–	–	–	–
2. FuJian-Pin.	*C. kyotensis*	*C. kyotensis*	DM----	CSF23065	20210524-1-(54)	OQ188594	OQ260569	–	–	–	–
2. FuJian-Pin.	*C. kyotensis*	*C. kyotensis*	DMAAAA	CSF23193	20210524-1-(239)	OQ188595	OQ260570	OQ261646	OQ303081	OQ303287	OQ303494
2. FuJian-Pin.	*C. kyotensis*	*C. kyotensis*	DO----	CSF23154	20210524-1-(186)	OQ188596	OQ260571	–	–	–	–
2. FuJian-Pin.	*C. kyotensis*	*C. kyotensis*	DOAAAA	CSF22519	20210524-1-(18)	OQ188597	OQ260572	OQ261647	OQ303082	OQ303288	OQ303495
2. FuJian-Pin.	*C. kyotensis*	*C. kyotensis*	DOAAAA	CSF23087	20210524-1-(94)	OQ188598	OQ260573	OQ261648	OQ303083	OQ303289	OQ303496
3. FuJian-Cun.	*C. kyotensis*	*C. aconidialis*	AA----	CSF22499	20210525-1-(7)	OQ188599	OQ260574	–	–	–	–
3. FuJian-Cun.	*C. kyotensis*	*C. aconidialis*	AA----	CSF22500	20210525-1-(8)	OQ188600	OQ260575	–	–	–	–
3. FuJian-Cun.	*C. kyotensis*	*C. aconidialis*	AA----	CSF22504	20210525-1-(13)	OQ188601	OQ260576	–	–	–	–
3. FuJian-Cun.	*C. kyotensis*	*C. aconidialis*	AA----	CSF22505	20210525-1-(14)	OQ188602	OQ260577	–	–	–	–
3. FuJian-Cun.	*C. kyotensis*	*C. aconidialis*	AA----	CSF22506	20210525-1-(15)	OQ188603	OQ260578	–	–	–	–
3. FuJian-Cun.	*C. kyotensis*	*C. aconidialis*	AA----	CSF22507	20210525-1-(16)	OQ188604	OQ260579	–	–	–	–
3. FuJian-Cun.	*C. kyotensis*	*C. aconidialis*	AA----	CSF22970	20210525-1-(26)	OQ188605	OQ260580	–	–	–	–
3. FuJian-Cun.	*C. kyotensis*	*C. aconidialis*	AA----	CSF22972	20210525-1-(29)	OQ188606	OQ260581	–	–	–	–
3. FuJian-Cun.	*C. kyotensis*	*C. aconidialis*	AA----	CSF22973	20210525-1-(33)	OQ188607	OQ260582	–	–	–	–
3. FuJian-Cun.	*C. kyotensis*	*C. aconidialis*	AA----	CSF22974	20210525-1-(34)	OQ188608	OQ260583	–	–	–	–
3. FuJian-Cun.	*C. kyotensis*	*C. aconidialis*	AA----	CSF22975	20210525-1-(36)	OQ188609	OQ260584	–	–	–	–
3. FuJian-Cun.	*C. kyotensis*	*C. aconidialis*	AA----	CSF22977	20210525-1-(38)	OQ188610	OQ260585	–	–	–	–
3. FuJian-Cun.	*C. kyotensis*	*C. aconidialis*	AA----	CSF22979	20210525-1-(40)	OQ188611	OQ260586	–	–	–	–
3. FuJian-Cun.	*C. kyotensis*	*C. aconidialis*	AA----	CSF22982	20210525-1-(44)	OQ188612	OQ260587	–	–	–	–
3. FuJian-Cun.	*C. kyotensis*	*C. aconidialis*	AA----	CSF22984	20210525-1-(53)	OQ188613	OQ260588	–	–	–	–
3. FuJian-Cun.	*C. kyotensis*	*C. aconidialis*	AA----	CSF22985	20210525-1-(57)	OQ188614	OQ260589	–	–	–	–
3. FuJian-Cun.	*C. kyotensis*	*C. aconidialis*	AA----	CSF22986	20210525-1-(58)	OQ188615	OQ260590	–	–	–	–
3. FuJian-Cun.	*C. kyotensis*	*C. aconidialis*	AA----	CSF22988	20210525-1-(67)	OQ188616	OQ260591	–	–	–	–
3. FuJian-Cun.	*C. kyotensis*	*C. aconidialis*	AA----	CSF22989	20210525-1-(69)	OQ188617	OQ260592	–	–	–	–
3. FuJian-Cun.	*C. kyotensis*	*C. aconidialis*	AA----	CSF22990	20210525-1-(72)	OQ188618	OQ260593	–	–	–	–
3. FuJian-Cun.	*C. kyotensis*	*C. aconidialis*	AA----	CSF22992	20210525-1-(76)	OQ188619	OQ260594	–	–	–	–
3. FuJian-Cun.	*C. kyotensis*	*C. aconidialis*	AA----	CSF22994	20210525-1-(86)	OQ188620	OQ260595	–	–	–	–
3. FuJian-Cun.	*C. kyotensis*	*C. aconidialis*	AA----	CSF22995	20210525-1-(90)	OQ188621	OQ260596	–	–	–	–
3. FuJian-Cun.	*C. kyotensis*	*C. aconidialis*	AA----	CSF22996	20210525-1-(91)	OQ188622	OQ260597	–	–	–	–
3. FuJian-Cun.	*C. kyotensis*	*C. aconidialis*	AA----	CSF22997	20210525-1-(93)	OQ188623	OQ260598	–	–	–	–
3. FuJian-Cun.	*C. kyotensis*	*C. aconidialis*	AA----	CSF23003	20210525-1-(111)	OQ188624	OQ260599	–	–	–	–
3. FuJian-Cun.	*C. kyotensis*	*C. aconidialis*	AA----	CSF23005	20210525-1-(119)	OQ188625	OQ260600	–	–	–	–
3. FuJian-Cun.	*C. kyotensis*	*C. aconidialis*	AA----	CSF23007	20210525-1-(124)	OQ188626	OQ260601	–	–	–	–
3. FuJian-Cun.	*C. kyotensis*	*C. aconidialis*	AA----	CSF23012	20210525-1-(144)	OQ188627	OQ260602	–	–	–	–
3. FuJian-Cun.	*C. kyotensis*	*C. aconidialis*	AA----	CSF23013	20210525-1-(148)	OQ188628	OQ260603	–	–	–	–
3. FuJian-Cun.	*C. kyotensis*	*C. aconidialis*	AA----	CSF23014	20210525-1-(152)	OQ188629	OQ260604	–	–	–	–
3. FuJian-Cun.	*C. kyotensis*	*C. aconidialis*	AA----	CSF23015	20210525-1-(156)	OQ188630	OQ260605	–	–	–	–
3. FuJian-Cun.	*C. kyotensis*	*C. aconidialis*	AA----	CSF23016	20210525-1-(159)	OQ188631	OQ260606	–	–	–	–
3. FuJian-Cun.	*C. kyotensis*	*C. aconidialis*	AA----	CSF23017	20210525-1-(160)	OQ188632	OQ260607	–	–	–	–
3. FuJian-Cun.	*C. kyotensis*	*C. aconidialis*	AA----	CSF23019	20210525-1-(171)	OQ188633	OQ260608	–	–	–	–
3. FuJian-Cun.	*C. kyotensis*	*C. aconidialis*	AA----	CSF23025	20210525-1-(190)	OQ188634	OQ260609	–	–	–	–
3. FuJian-Cun.	*C. kyotensis*	*C. aconidialis*	AA----	CSF23026	20210525-1-(196)	OQ188635	OQ260610	–	–	–	–
3. FuJian-Cun.	*C. kyotensis*	*C. aconidialis*	AA----	CSF23027	20210525-1-(197)	OQ188636	OQ260611	–	–	–	–
3. FuJian-Cun.	*C. kyotensis*	*C. aconidialis*	AA----	CSF23028	20210525-1-(198)	OQ188637	OQ260612	–	–	–	–
3. FuJian-Cun.	*C. kyotensis*	*C. aconidialis*	AA----	CSF23029	20210525-1-(201)	OQ188638	OQ260613	–	–	–	–
3. FuJian-Cun.	*C. kyotensis*	*C. aconidialis*	AA----	CSF23030	20210525-1-(202)	OQ188639	OQ260614	–	–	–	–
3. FuJian-Cun.	*C. kyotensis*	*C. aconidialis*	AA----	CSF23031	20210525-1-(203)	OQ188640	OQ260615	–	–	–	–
3. FuJian-Cun.	*C. kyotensis*	*C. aconidialis*	AA----	CSF23033	20210525-1-(206)	OQ188641	OQ260616	–	–	–	–
3. FuJian-Cun.	*C. kyotensis*	*C. aconidialis*	AA----	CSF23039	20210525-1-(223)	OQ188642	OQ260617	–	–	–	–
3. FuJian-Cun.	*C. kyotensis*	*C. aconidialis*	AA----	CSF23040	20210525-1-(225)	OQ188643	OQ260618	–	–	–	–
3. FuJian-Cun.	*C. kyotensis*	*C. aconidialis*	AA----	CSF23041	20210525-1-(226)	OQ188644	OQ260619	–	–	–	–
3. FuJian-Cun.	*C. kyotensis*	*C. aconidialis*	AA----	CSF23042	20210525-1-(229)	OQ188645	OQ260620	–	–	–	–
3. FuJian-Cun.	*C. kyotensis*	*C. aconidialis*	AA----	CSF23043	20210525-1-(231)	OQ188646	OQ260621	–	–	–	–
3. FuJian-Cun.	*C. kyotensis*	*C. aconidialis*	AA----	CSF23045	20210525-1-(234)	OQ188647	OQ260622	–	–	–	–
3. FuJian-Cun.	*C. kyotensis*	*C. aconidialis*	AA----	CSF23048	20210525-1-(247)	OQ188648	OQ260623	–	–	–	–
3. FuJian-Cun.	*C. kyotensis*	*C. aconidialis*	AAABAA	CSF22498	20210525-1-(4)	OQ188649	OQ260624	OQ261463	OQ302898	OQ303105	OQ303311
3. FuJian-Cun.	*C. kyotensis*	*C. aconidialis*	ADDAAA	CSF22991	20210525-1-(74)	OQ188650	OQ260625	OQ261469	OQ302904	OQ303111	OQ303317
3. FuJian-Cun.	*C. kyotensis*	*C. aconidialis*	AFAAAA	CSF23011	20210525-1-(141)	OQ188651	OQ260626	OQ261471	OQ302906	OQ303113	OQ303319
3. FuJian-Cun.	*C. kyotensis*	*C. aconidialis*	AI----	CSF22987	20210525-1-(60)	OQ188652	OQ260627	–	–	–	–
3. FuJian-Cun.	*C. kyotensis*	*C. aconidialis*	AI----	CSF23006	20210525-1-(122)	OQ188653	OQ260628	–	–	–	–
3. FuJian-Cun.	*C. kyotensis*	*C. aconidialis*	AI----	CSF23034	20210525-1-(209)	OQ188654	OQ260629	–	–	–	–
3. FuJian-Cun.	*C. kyotensis*	*C. aconidialis*	AI----	CSF23044	20210525-1-(232)	OQ188655	OQ260630	–	–	–	–
3. FuJian-Cun.	*C. kyotensis*	*C. aconidialis*	AIAAAA	CSF23008	20210525-1-(137)	OQ188656	OQ260631	OQ261477	OQ302912	OQ303119	OQ303325
3. FuJian-Cun.	*C. kyotensis*	*C. aconidialis*	AQAAAA	CSF22503	20210525-1-(12)	OQ188657	OQ260632	OQ261488	OQ302923	OQ303130	OQ303336
3. FuJian-Cun.	*C. kyotensis*	*C. aconidialis*	AU----	CSF22978	20210525-1-(39)	OQ188658	OQ260633	–	–	–	–
3. FuJian-Cun.	*C. kyotensis*	*C. aconidialis*	AU----	CSF23001	20210525-1-(101)	OQ188659	OQ260634	–	–	–	–
3. FuJian-Cun.	*C. kyotensis*	*C. aconidialis*	AU----	CSF23022	20210525-1-(186)	OQ188660	OQ260635	–	–	–	–
3. FuJian-Cun.	*C. kyotensis*	*C. aconidialis*	AU----	CSF23023	20210525-1-(187)	OQ188661	OQ260636	–	–	–	–
3. FuJian-Cun.	*C. kyotensis*	*C. aconidialis*	AU----	CSF23024	20210525-1-(188)	OQ188662	OQ260637	–	–	–	–
3. FuJian-Cun.	*C. kyotensis*	*C. aconidialis*	AU----	CSF23046	20210525-1-(235)	OQ188663	OQ260638	–	–	–	–
3. FuJian-Cun.	*C. kyotensis*	*C. aconidialis*	AUAAAA	CSF23021	20210525-1-(185)	OQ188664	OQ260639	OQ261496	OQ302931	OQ303138	OQ303344
3. FuJian-Cun.	*C. kyotensis*	*C. aconidialis*	CA----	CSF22497	20210525-1-(1)	OQ188665	OQ260640	–	–	–	–
3. FuJian-Cun.	*C. kyotensis*	*C. aconidialis*	CA----	CSF22502	20210525-1-(11)	OQ188666	OQ260641	–	–	–	–
3. FuJian-Cun.	*C. kyotensis*	*C. aconidialis*	CA----	CSF22508	20210525-1-(17)	OQ188667	OQ260642	–	–	–	–
3. FuJian-Cun.	*C. kyotensis*	*C. aconidialis*	CA----	CSF22976	20210525-1-(37)	OQ188668	OQ260643	–	–	–	–
3. FuJian-Cun.	*C. kyotensis*	*C. aconidialis*	CA----	CSF22993	20210525-1-(85)	OQ188669	OQ260644	–	–	–	–
3. FuJian-Cun.	*C. kyotensis*	*C. aconidialis*	CA----	CSF22999	20210525-1-(95)	OQ188670	OQ260645	–	–	–	–
3. FuJian-Cun.	*C. kyotensis*	*C. aconidialis*	CA----	CSF23020	20210525-1-(184)	OQ188671	OQ260646	–	–	–	–
3. FuJian-Cun.	*C. kyotensis*	*C. aconidialis*	CA----	CSF23032	20210525-1-(204)	OQ188672	OQ260647	–	–	–	–
3. FuJian-Cun.	*C. kyotensis*	*C. aconidialis*	CA----	CSF23035	20210525-1-(212)	OQ188673	OQ260648	–	–	–	–
3. FuJian-Cun.	*C. kyotensis*	*C. aconidialis*	CA----	CSF23037	20210525-1-(218)	OQ188674	OQ260649	–	–	–	–
3. FuJian-Cun.	*C. kyotensis*	*C. aconidialis*	CA----	CSF23038	20210525-1-(221)	OQ188675	OQ260650	–	–	–	–
3. FuJian-Cun.	*C. kyotensis*	*C. aconidialis*	CAAAAA	CSF23049	20210525-1-(250)	OQ188676	OQ260651	OQ261501	OQ302936	OQ303143	OQ303349
3. FuJian-Cun.	*C. kyotensis*	*C. aconidialis*	CADAAA	CSF23002	20210525-1-(110)	OQ188677	OQ260652	OQ261505	OQ302940	OQ303147	OQ303353
3. FuJian-Cun.	*C. kyotensis*	*C. chinensis*	AAAAAA	CSF22980	20210525-1-(41)	OQ188678	OQ260653	OQ261520	OQ302955	OQ303162	OQ303368
3. FuJian-Cun.	*C. kyotensis*	*C. chinensis*	BAAAAA	CSF22981	20210525-1-(43)	OQ188679	OQ260654	OQ261521	OQ302956	OQ303163	OQ303369
3. FuJian-Cun.	*C. kyotensis*	*C. hongkongensis*	AA----	CSF22509	20210525-1-(24)	OQ188680	OQ260655	–	–	–	–
3. FuJian-Cun.	*C. kyotensis*	*C. hongkongensis*	AA----	CSF22971	20210525-1-(28)	OQ188681	OQ260656	–	–	–	–
3. FuJian-Cun.	*C. kyotensis*	*C. hongkongensis*	AA----	CSF22983	20210525-1-(45)	OQ188682	OQ260657	–	–	–	–
3. FuJian-Cun.	*C. kyotensis*	*C. hongkongensis*	AA----	CSF23018	20210525-1-(166)	OQ188683	OQ260658	–	–	–	–
3. FuJian-Cun.	*C. kyotensis*	*C. hongkongensis*	ADBAAA	CSF22501	20210525-1-(10)	OQ188684	OQ260659	OQ261530	OQ302965	OQ303172	OQ303378
3. FuJian-Cun.	*C. kyotensis*	*C. hongkongensis*	AFCAAA	CSF23000	20210525-1-(98)	OQ188685	OQ260660	OQ261543	OQ302978	OQ303185	OQ303391
3. FuJian-Cun.	*C. kyotensis*	*C. ilicicola*	AA----	CSF22998	20210525-1-(94)	OQ188686	OQ260661	–	–	–	–
3. FuJian-Cun.	*C. kyotensis*	*C. ilicicola*	AA----	CSF23004	20210525-1-(113)	OQ188687	OQ260662	–	–	–	–
3. FuJian-Cun.	*C. kyotensis*	*C. ilicicola*	AA----	CSF23036	20210525-1-(216)	OQ188688	OQ260663	–	–	–	–
3. FuJian-Cun.	*C. kyotensis*	*C. ilicicola*	AA----	CSF23047	20210525-1-(245)	OQ188689	OQ260664	–	–	–	–
4. GuangDong-Euc.	*C. kyotensis*	*C. aconidialis*	AA----	CSF22525	20210607-1-(2)	OQ188690	OQ260665	–	–	–	–
4. GuangDong-Euc.	*C. kyotensis*	*C. aconidialis*	AA----	CSF22526	20210607-1-(3)	OQ188691	OQ260666	–	–	–	–
4. GuangDong-Euc.	*C. kyotensis*	*C. aconidialis*	AA----	CSF22527	20210607-1-(5)	OQ188692	OQ260667	–	–	–	–
4. GuangDong-Euc.	*C. kyotensis*	*C. aconidialis*	AA----	CSF22528	20210607-1-(6)	OQ188693	OQ260668	–	–	–	–
4. GuangDong-Euc.	*C. kyotensis*	*C. aconidialis*	AA----	CSF22529	20210607-1-(8)	OQ188694	OQ260669	–	–	–	–
4. GuangDong-Euc.	*C. kyotensis*	*C. aconidialis*	AA----	CSF22531	20210607-1-(10)	OQ188695	OQ260670	–	–	–	–
4. GuangDong-Euc.	*C. kyotensis*	*C. aconidialis*	AA----	CSF22532	20210607-1-(13)	OQ188696	OQ260671	–	–	–	–
4. GuangDong-Euc.	*C. kyotensis*	*C. aconidialis*	AA----	CSF22533	20210607-1-(14)	OQ188697	OQ260672	–	–	–	–
4. GuangDong-Euc.	*C. kyotensis*	*C. aconidialis*	AA----	CSF22534	20210607-1-(16)	OQ188698	OQ260673	–	–	–	–
4. GuangDong-Euc.	*C. kyotensis*	*C. aconidialis*	AA----	CSF22538	20210607-1-(19)	OQ188699	OQ260674	–	–	–	–
4. GuangDong-Euc.	*C. kyotensis*	*C. aconidialis*	AA----	CSF22540	20210607-1-(22)	OQ188700	OQ260675	–	–	–	–
4. GuangDong-Euc.	*C. kyotensis*	*C. aconidialis*	AA----	CSF22541	20210607-1-(24)	OQ188701	OQ260676	–	–	–	–
4. GuangDong-Euc.	*C. kyotensis*	*C. aconidialis*	AA----	CSF23201	20210607-1-(26)	OQ188702	OQ260677	–	–	–	–
4. GuangDong-Euc.	*C. kyotensis*	*C. aconidialis*	AA----	CSF23202	20210607-1-(27)	OQ188703	OQ260678	–	–	–	–
4. GuangDong-Euc.	*C. kyotensis*	*C. aconidialis*	AA----	CSF23203	20210607-1-(28)	OQ188704	OQ260679	–	–	–	–
4. GuangDong-Euc.	*C. kyotensis*	*C. aconidialis*	AA----	CSF23204	20210607-1-(29)	OQ188705	OQ260680	–	–	–	–
4. GuangDong-Euc.	*C. kyotensis*	*C. aconidialis*	AA----	CSF23205	20210607-1-(30)	OQ188706	OQ260681	–	–	–	–
4. GuangDong-Euc.	*C. kyotensis*	*C. aconidialis*	AA----	CSF23206	20210607-1-(31)	OQ188707	OQ260682	–	–	–	–
4. GuangDong-Euc.	*C. kyotensis*	*C. aconidialis*	AA----	CSF23207	20210607-1-(32)	OQ188708	OQ260683	–	–	–	–
4. GuangDong-Euc.	*C. kyotensis*	*C. aconidialis*	AA----	CSF23208	20210607-1-(33)	OQ188709	OQ260684	–	–	–	–
4. GuangDong-Euc.	*C. kyotensis*	*C. aconidialis*	AA----	CSF23209	20210607-1-(34)	OQ188710	OQ260685	–	–	–	–
4. GuangDong-Euc.	*C. kyotensis*	*C. aconidialis*	AA----	CSF23211	20210607-1-(36)	OQ188711	OQ260686	–	–	–	–
4. GuangDong-Euc.	*C. kyotensis*	*C. aconidialis*	AA----	CSF23213	20210607-1-(38)	OQ188712	OQ260687	–	–	–	–
4. GuangDong-Euc.	*C. kyotensis*	*C. aconidialis*	AA----	CSF23215	20210607-1-(40)	OQ188713	OQ260688	–	–	–	–
4. GuangDong-Euc.	*C. kyotensis*	*C. aconidialis*	AA----	CSF23216	20210607-1-(41)	OQ188714	OQ260689	–	–	–	–
4. GuangDong-Euc.	*C. kyotensis*	*C. aconidialis*	AA----	CSF23217	20210607-1-(42)	OQ188715	OQ260690	–	–	–	–
4. GuangDong-Euc.	*C. kyotensis*	*C. aconidialis*	AA----	CSF23218	20210607-1-(43)	OQ188716	OQ260691	–	–	–	–
4. GuangDong-Euc.	*C. kyotensis*	*C. aconidialis*	AA----	CSF23219	20210607-1-(44)	OQ188717	OQ260692	–	–	–	–
4. GuangDong-Euc.	*C. kyotensis*	*C. aconidialis*	AA----	CSF23222	20210607-1-(47)	OQ188718	OQ260693	–	–	–	–
4. GuangDong-Euc.	*C. kyotensis*	*C. aconidialis*	AA----	CSF23223	20210607-1-(48)	OQ188719	OQ260694	–	–	–	–
4. GuangDong-Euc.	*C. kyotensis*	*C. aconidialis*	AA----	CSF23224	20210607-1-(49)	OQ188720	OQ260695	–	–	–	–
4. GuangDong-Euc.	*C. kyotensis*	*C. aconidialis*	AA----	CSF23225	20210607-1-(51)	OQ188721	OQ260696	–	–	–	–
4. GuangDong-Euc.	*C. kyotensis*	*C. aconidialis*	AA----	CSF23226	20210607-1-(53)	OQ188722	OQ260697	–	–	–	–
4. GuangDong-Euc.	*C. kyotensis*	*C. aconidialis*	AA----	CSF23228	20210607-1-(55)	OQ188723	OQ260698	–	–	–	–
4. GuangDong-Euc.	*C. kyotensis*	*C. aconidialis*	AA----	CSF23229	20210607-1-(56)	OQ188724	OQ260699	–	–	–	–
4. GuangDong-Euc.	*C. kyotensis*	*C. aconidialis*	AA----	CSF23232	20210607-1-(59)	OQ188725	OQ260700	–	–	–	–
4. GuangDong-Euc.	*C. kyotensis*	*C. aconidialis*	AA----	CSF23233	20210607-1-(60)	OQ188726	OQ260701	–	–	–	–
4. GuangDong-Euc.	*C. kyotensis*	*C. aconidialis*	AA----	CSF23234	20210607-1-(61)	OQ188727	OQ260702	–	–	–	–
4. GuangDong-Euc.	*C. kyotensis*	*C. aconidialis*	AA----	CSF23235	20210607-1-(62)	OQ188728	OQ260703	–	–	–	–
4. GuangDong-Euc.	*C. kyotensis*	*C. aconidialis*	AA----	CSF23236	20210607-1-(63)	OQ188729	OQ260704	–	–	–	–
4. GuangDong-Euc.	*C. kyotensis*	*C. aconidialis*	AA----	CSF23238	20210607-1-(65)	OQ188730	OQ260705	–	–	–	–
4. GuangDong-Euc.	*C. kyotensis*	*C. aconidialis*	AA----	CSF23239	20210607-1-(66)	OQ188731	OQ260706	–	–	–	–
4. GuangDong-Euc.	*C. kyotensis*	*C. aconidialis*	AA----	CSF23241	20210607-1-(68)	OQ188732	OQ260707	–	–	–	–
4. GuangDong-Euc.	*C. kyotensis*	*C. aconidialis*	AA----	CSF23242	20210607-1-(69)	OQ188733	OQ260708	–	–	–	–
4. GuangDong-Euc.	*C. kyotensis*	*C. aconidialis*	AA----	CSF23243	20210607-1-(70)	OQ188734	OQ260709	–	–	–	–
4. GuangDong-Euc.	*C. kyotensis*	*C. aconidialis*	AA----	CSF23246	20210607-1-(74)	OQ188735	OQ260710	–	–	–	–
4. GuangDong-Euc.	*C. kyotensis*	*C. aconidialis*	AA----	CSF23248	20210607-1-(76)	OQ188736	OQ260711	–	–	–	–
4. GuangDong-Euc.	*C. kyotensis*	*C. aconidialis*	AA----	CSF23249	20210607-1-(77)	OQ188737	OQ260712	–	–	–	–
4. GuangDong-Euc.	*C. kyotensis*	*C. aconidialis*	AA----	CSF23252	20210607-1-(80)	OQ188738	OQ260713	–	–	–	–
4. GuangDong-Euc.	*C. kyotensis*	*C. aconidialis*	AA----	CSF23253	20210607-1-(81)	OQ188739	OQ260714	–	–	–	–
4. GuangDong-Euc.	*C. kyotensis*	*C. aconidialis*	AA----	CSF23254	20210607-1-(82)	OQ188740	OQ260715	–	–	–	–
4. GuangDong-Euc.	*C. kyotensis*	*C. aconidialis*	AA----	CSF23255	20210607-1-(83)	OQ188741	OQ260716	–	–	–	–
4. GuangDong-Euc.	*C. kyotensis*	*C. aconidialis*	AA----	CSF23256	20210607-1-(84)	OQ188742	OQ260717	–	–	–	–
4. GuangDong-Euc.	*C. kyotensis*	*C. aconidialis*	AA----	CSF23257	20210607-1-(85)	OQ188743	OQ260718	–	–	–	–
4. GuangDong-Euc.	*C. kyotensis*	*C. aconidialis*	AA----	CSF23259	20210607-1-(87)	OQ188744	OQ260719	–	–	–	–
4. GuangDong-Euc.	*C. kyotensis*	*C. aconidialis*	AA----	CSF23260	20210607-1-(88)	OQ188745	OQ260720	–	–	–	–
4. GuangDong-Euc.	*C. kyotensis*	*C. aconidialis*	AA----	CSF23261	20210607-1-(89)	OQ188746	OQ260721	–	–	–	–
4. GuangDong-Euc.	*C. kyotensis*	*C. aconidialis*	AA----	CSF23264	20210607-1-(92)	OQ188747	OQ260722	–	–	–	–
4. GuangDong-Euc.	*C. kyotensis*	*C. aconidialis*	AA----	CSF23265	20210607-1-(93)	OQ188748	OQ260723	–	–	–	–
4. GuangDong-Euc.	*C. kyotensis*	*C. aconidialis*	AA----	CSF23266	20210607-1-(94)	OQ188749	OQ260724	–	–	–	–
4. GuangDong-Euc.	*C. kyotensis*	*C. aconidialis*	AA----	CSF23267	20210607-1-(95)	OQ188750	OQ260725	–	–	–	–
4. GuangDong-Euc.	*C. kyotensis*	*C. aconidialis*	AA----	CSF23268	20210607-1-(96)	OQ188751	OQ260726	–	–	–	–
4. GuangDong-Euc.	*C. kyotensis*	*C. aconidialis*	AA----	CSF23270	20210607-1-(98)	OQ188752	OQ260727	–	–	–	–
4. GuangDong-Euc.	*C. kyotensis*	*C. aconidialis*	AA----	CSF23271	20210607-1-(99)	OQ188753	OQ260728	–	–	–	–
4. GuangDong-Euc.	*C. kyotensis*	*C. aconidialis*	AA----	CSF23272	20210607-1-(100)	OQ188754	OQ260729	–	–	–	–
4. GuangDong-Euc.	*C. kyotensis*	*C. aconidialis*	AA----	CSF23273	20210607-1-(101)	OQ188755	OQ260730	–	–	–	–
4. GuangDong-Euc.	*C. kyotensis*	*C. aconidialis*	AA----	CSF23274	20210607-1-(102)	OQ188756	OQ260731	–	–	–	–
4. GuangDong-Euc.	*C. kyotensis*	*C. aconidialis*	AA----	CSF23275	20210607-1-(103)	OQ188757	OQ260732	–	–	–	–
4. GuangDong-Euc.	*C. kyotensis*	*C. aconidialis*	AA----	CSF23276	20210607-1-(104)	OQ188758	OQ260733	–	–	–	–
4. GuangDong-Euc.	*C. kyotensis*	*C. aconidialis*	AA----	CSF23277	20210607-1-(105)	OQ188759	OQ260734	–	–	–	–
4. GuangDong-Euc.	*C. kyotensis*	*C. aconidialis*	AA----	CSF23278	20210607-1-(106)	OQ188760	OQ260735	–	–	–	–
4. GuangDong-Euc.	*C. kyotensis*	*C. aconidialis*	AA----	CSF23279	20210607-1-(107)	OQ188761	OQ260736	–	–	–	–
4. GuangDong-Euc.	*C. kyotensis*	*C. aconidialis*	AA----	CSF23280	20210607-1-(108)	OQ188762	OQ260737	–	–	–	–
4. GuangDong-Euc.	*C. kyotensis*	*C. aconidialis*	AA----	CSF23281	20210607-1-(109)	OQ188763	OQ260738	–	–	–	–
4. GuangDong-Euc.	*C. kyotensis*	*C. aconidialis*	AA----	CSF23282	20210607-1-(110)	OQ188764	OQ260739	–	–	–	–
4. GuangDong-Euc.	*C. kyotensis*	*C. aconidialis*	AA----	CSF23283	20210607-1-(111)	OQ188765	OQ260740	–	–	–	–
4. GuangDong-Euc.	*C. kyotensis*	*C. aconidialis*	AA----	CSF23286	20210607-1-(114)	OQ188766	OQ260741	–	–	–	–
4. GuangDong-Euc.	*C. kyotensis*	*C. aconidialis*	AA----	CSF23288	20210607-1-(116)	OQ188767	OQ260742	–	–	–	–
4. GuangDong-Euc.	*C. kyotensis*	*C. aconidialis*	AA----	CSF23290	20210607-1-(119)	OQ188768	OQ260743	–	–	–	–
4. GuangDong-Euc.	*C. kyotensis*	*C. aconidialis*	AA----	CSF23291	20210607-1-(120)	OQ188769	OQ260744	–	–	–	–
4. GuangDong-Euc.	*C. kyotensis*	*C. aconidialis*	AA----	CSF23292	20210607-1-(122)	OQ188770	OQ260745	–	–	–	–
4. GuangDong-Euc.	*C. kyotensis*	*C. aconidialis*	AA----	CSF23293	20210607-1-(123)	OQ188771	OQ260746	–	–	–	–
4. GuangDong-Euc.	*C. kyotensis*	*C. aconidialis*	AA----	CSF23295	20210607-1-(128)	OQ188772	OQ260747	–	–	–	–
4. GuangDong-Euc.	*C. kyotensis*	*C. aconidialis*	AA----	CSF23296	20210607-1-(129)	OQ188773	OQ260748	–	–	–	–
4. GuangDong-Euc.	*C. kyotensis*	*C. aconidialis*	AA----	CSF23297	20210607-1-(130)	OQ188774	OQ260749	–	–	–	–
4. GuangDong-Euc.	*C. kyotensis*	*C. aconidialis*	AA----	CSF23298	20210607-1-(131)	OQ188775	OQ260750	–	–	–	–
4. GuangDong-Euc.	*C. kyotensis*	*C. aconidialis*	AA----	CSF23299	20210607-1-(132)	OQ188776	OQ260751	–	–	–	–
4. GuangDong-Euc.	*C. kyotensis*	*C. aconidialis*	AA----	CSF23300	20210607-1-(134)	OQ188777	OQ260752	–	–	–	–
4. GuangDong-Euc.	*C. kyotensis*	*C. aconidialis*	AA----	CSF23303	20210607-1-(138)	OQ188778	OQ260753	–	–	–	–
4. GuangDong-Euc.	*C. kyotensis*	*C. aconidialis*	AA----	CSF23305	20210607-1-(140)	OQ188779	OQ260754	–	–	–	–
4. GuangDong-Euc.	*C. kyotensis*	*C. aconidialis*	AA----	CSF23307	20210607-1-(142)	OQ188780	OQ260755	–	–	–	–
4. GuangDong-Euc.	*C. kyotensis*	*C. aconidialis*	AA----	CSF23308	20210607-1-(143)	OQ188781	OQ260756	–	–	–	–
4. GuangDong-Euc.	*C. kyotensis*	*C. aconidialis*	AA----	CSF23310	20210607-1-(146)	OQ188782	OQ260757	–	–	–	–
4. GuangDong-Euc.	*C. kyotensis*	*C. aconidialis*	AA----	CSF23311	20210607-1-(148)	OQ188783	OQ260758	–	–	–	–
4. GuangDong-Euc.	*C. kyotensis*	*C. aconidialis*	AA----	CSF23312	20210607-1-(149)	OQ188784	OQ260759	–	–	–	–
4. GuangDong-Euc.	*C. kyotensis*	*C. aconidialis*	AA----	CSF23313	20210607-1-(150)	OQ188785	OQ260760	–	–	–	–
4. GuangDong-Euc.	*C. kyotensis*	*C. aconidialis*	AA----	CSF23314	20210607-1-(151)	OQ188786	OQ260761	–	–	–	–
4. GuangDong-Euc.	*C. kyotensis*	*C. aconidialis*	AA----	CSF23318	20210607-1-(156)	OQ188787	OQ260762	–	–	–	–
4. GuangDong-Euc.	*C. kyotensis*	*C. aconidialis*	AA----	CSF23319	20210607-1-(157)	OQ188788	OQ260763	–	–	–	–
4. GuangDong-Euc.	*C. kyotensis*	*C. aconidialis*	AA----	CSF23320	20210607-1-(158)	OQ188789	OQ260764	–	–	–	–
4. GuangDong-Euc.	*C. kyotensis*	*C. aconidialis*	AA----	CSF23322	20210607-1-(161)	OQ188790	OQ260765	–	–	–	–
4. GuangDong-Euc.	*C. kyotensis*	*C. aconidialis*	AA----	CSF23326	20210607-1-(165)	OQ188791	OQ260766	–	–	–	–
4. GuangDong-Euc.	*C. kyotensis*	*C. aconidialis*	AA----	CSF23328	20210607-1-(167)	OQ188792	OQ260767	–	–	–	–
4. GuangDong-Euc.	*C. kyotensis*	*C. aconidialis*	AA----	CSF23331	20210607-1-(170)	OQ188793	OQ260768	–	–	–	–
4. GuangDong-Euc.	*C. kyotensis*	*C. aconidialis*	AA----	CSF23333	20210607-1-(172)	OQ188794	OQ260769	–	–	–	–
4. GuangDong-Euc.	*C. kyotensis*	*C. aconidialis*	AA----	CSF23334	20210607-1-(174)	OQ188795	OQ260770	–	–	–	–
4. GuangDong-Euc.	*C. kyotensis*	*C. aconidialis*	AA----	CSF23335	20210607-1-(175)	OQ188796	OQ260771	–	–	–	–
4. GuangDong-Euc.	*C. kyotensis*	*C. aconidialis*	AA----	CSF23336	20210607-1-(176)	OQ188797	OQ260772	–	–	–	–
4. GuangDong-Euc.	*C. kyotensis*	*C. aconidialis*	AA----	CSF23338	20210607-1-(178)	OQ188798	OQ260773	–	–	–	–
4. GuangDong-Euc.	*C. kyotensis*	*C. aconidialis*	AA----	CSF23342	20210607-1-(182)	OQ188799	OQ260774	–	–	–	–
4. GuangDong-Euc.	*C. kyotensis*	*C. aconidialis*	AA----	CSF23343	20210607-1-(184)	OQ188800	OQ260775	–	–	–	–
4. GuangDong-Euc.	*C. kyotensis*	*C. aconidialis*	AA----	CSF23344	20210607-1-(185)	OQ188801	OQ260776	–	–	–	–
4. GuangDong-Euc.	*C. kyotensis*	*C. aconidialis*	AA----	CSF23345	20210607-1-(186)	OQ188802	OQ260777	–	–	–	–
4. GuangDong-Euc.	*C. kyotensis*	*C. aconidialis*	AA----	CSF23348	20210607-1-(189)	OQ188803	OQ260778	–	–	–	–
4. GuangDong-Euc.	*C. kyotensis*	*C. aconidialis*	AA----	CSF23349	20210607-1-(190)	OQ188804	OQ260779	–	–	–	–
4. GuangDong-Euc.	*C. kyotensis*	*C. aconidialis*	AA----	CSF23350	20210607-1-(191)	OQ188805	OQ260780	–	–	–	–
4. GuangDong-Euc.	*C. kyotensis*	*C. aconidialis*	AA----	CSF23351	20210607-1-(192)	OQ188806	OQ260781	–	–	–	–
4. GuangDong-Euc.	*C. kyotensis*	*C. aconidialis*	AA----	CSF23352	20210607-1-(194)	OQ188807	OQ260782	–	–	–	–
4. GuangDong-Euc.	*C. kyotensis*	*C. aconidialis*	AA----	CSF23354	20210607-1-(196)	OQ188808	OQ260783	–	–	–	–
4. GuangDong-Euc.	*C. kyotensis*	*C. aconidialis*	AA----	CSF23356	20210607-1-(198)	OQ188809	OQ260784	–	–	–	–
4. GuangDong-Euc.	*C. kyotensis*	*C. aconidialis*	AA----	CSF23359	20210607-1-(202)	OQ188810	OQ260785	–	–	–	–
4. GuangDong-Euc.	*C. kyotensis*	*C. aconidialis*	AA----	CSF23360	20210607-1-(203)	OQ188811	OQ260786	–	–	–	–
4. GuangDong-Euc.	*C. kyotensis*	*C. aconidialis*	AA----	CSF23361	20210607-1-(205)	OQ188812	OQ260787	–	–	–	–
4. GuangDong-Euc.	*C. kyotensis*	*C. aconidialis*	AA----	CSF23362	20210607-1-(206)	OQ188813	OQ260788	–	–	–	–
4. GuangDong-Euc.	*C. kyotensis*	*C. aconidialis*	AA----	CSF23363	20210607-1-(207)	OQ188814	OQ260789	–	–	–	–
4. GuangDong-Euc.	*C. kyotensis*	*C. aconidialis*	AA----	CSF23364	20210607-1-(211)	OQ188815	OQ260790	–	–	–	–
4. GuangDong-Euc.	*C. kyotensis*	*C. aconidialis*	AA----	CSF23365	20210607-1-(213)	OQ188816	OQ260791	–	–	–	–
4. GuangDong-Euc.	*C. kyotensis*	*C. aconidialis*	AA----	CSF23367	20210607-1-(215)	OQ188817	OQ260792	–	–	–	–
4. GuangDong-Euc.	*C. kyotensis*	*C. aconidialis*	AA----	CSF23368	20210607-1-(216)	OQ188818	OQ260793	–	–	–	–
4. GuangDong-Euc.	*C. kyotensis*	*C. aconidialis*	AA----	CSF23369	20210607-1-(217)	OQ188819	OQ260794	–	–	–	–
4. GuangDong-Euc.	*C. kyotensis*	*C. aconidialis*	AA----	CSF23371	20210607-1-(220)	OQ188820	OQ260795	–	–	–	–
4. GuangDong-Euc.	*C. kyotensis*	*C. aconidialis*	AA----	CSF23372	20210607-1-(221)	OQ188821	OQ260796	–	–	–	–
4. GuangDong-Euc.	*C. kyotensis*	*C. aconidialis*	AA----	CSF23373	20210607-1-(222)	OQ188822	OQ260797	–	–	–	–
4. GuangDong-Euc.	*C. kyotensis*	*C. aconidialis*	AA----	CSF23374	20210607-1-(223)	OQ188823	OQ260798	–	–	–	–
4. GuangDong-Euc.	*C. kyotensis*	*C. aconidialis*	AA----	CSF23376	20210607-1-(226)	OQ188824	OQ260799	–	–	–	–
4. GuangDong-Euc.	*C. kyotensis*	*C. aconidialis*	AA----	CSF23377	20210607-1-(228)	OQ188825	OQ260800	–	–	–	–
4. GuangDong-Euc.	*C. kyotensis*	*C. aconidialis*	AA----	CSF23378	20210607-1-(229)	OQ188826	OQ260801	–	–	–	–
4. GuangDong-Euc.	*C. kyotensis*	*C. aconidialis*	AA----	CSF23380	20210607-1-(232)	OQ188827	OQ260802	–	–	–	–
4. GuangDong-Euc.	*C. kyotensis*	*C. aconidialis*	AA----	CSF23381	20210607-1-(233)	OQ188828	OQ260803	–	–	–	–
4. GuangDong-Euc.	*C. kyotensis*	*C. aconidialis*	AA----	CSF23383	20210607-1-(235)	OQ188829	OQ260804	–	–	–	–
4. GuangDong-Euc.	*C. kyotensis*	*C. aconidialis*	AA----	CSF23385	20210607-1-(237)	OQ188830	OQ260805	–	–	–	–
4. GuangDong-Euc.	*C. kyotensis*	*C. aconidialis*	AA----	CSF23386	20210607-1-(239)	OQ188831	OQ260806	–	–	–	–
4. GuangDong-Euc.	*C. kyotensis*	*C. aconidialis*	AA----	CSF23387	20210607-1-(240)	OQ188832	OQ260807	–	–	–	–
4. GuangDong-Euc.	*C. kyotensis*	*C. aconidialis*	AA----	CSF23388	20210607-1-(241)	OQ188833	OQ260808	–	–	–	–
4. GuangDong-Euc.	*C. kyotensis*	*C. aconidialis*	AA----	CSF23391	20210607-1-(245)	OQ188834	OQ260809	–	–	–	–
4. GuangDong-Euc.	*C. kyotensis*	*C. aconidialis*	AA----	CSF23392	20210607-1-(246)	OQ188835	OQ260810	–	–	–	–
4. GuangDong-Euc.	*C. kyotensis*	*C. aconidialis*	AA----	CSF23393	20210607-1-(247)	OQ188836	OQ260811	–	–	–	–
4. GuangDong-Euc.	*C. kyotensis*	*C. aconidialis*	AA----	CSF23395	20210607-1-(249)	OQ188837	OQ260812	–	–	–	–
4. GuangDong-Euc.	*C. kyotensis*	*C. aconidialis*	AA----	CSF23396	20210607-1-(250)	OQ188838	OQ260813	–	–	–	–
4. GuangDong-Euc.	*C. kyotensis*	*C. aconidialis*	ABAAAA	CSF23317	20210607-1-(154)	OQ188839	OQ260814	OQ261464	OQ302899	OQ303106	OQ303312
4. GuangDong-Euc.	*C. kyotensis*	*C. aconidialis*	AEAAAA	CSF23379	20210607-1-(231)	OQ188840	OQ260815	OQ261470	OQ302905	OQ303112	OQ303318
4. GuangDong-Euc.	*C. kyotensis*	*C. aconidialis*	AM----	CSF23231	20210607-1-(58)	OQ188841	OQ260816	–	–	–	–
4. GuangDong-Euc.	*C. kyotensis*	*C. aconidialis*	AM----	CSF23340	20210607-1-(180)	OQ188842	OQ260817	–	–	–	–
4. GuangDong-Euc.	*C. kyotensis*	*C. aconidialis*	AM----	CSF23353	20210607-1-(195)	OQ188843	OQ260818	–	–	–	–
4. GuangDong-Euc.	*C. kyotensis*	*C. aconidialis*	AOCAAA	CSF23284	20210607-1-(112)	OQ188844	OQ260819	OQ261486	OQ302921	OQ303128	OQ303334
4. GuangDong-Euc.	*C. kyotensis*	*C. aconidialis*	AR----	CSF23212	20210607-1-(37)	OQ188845	OQ260820	–	–	–	–
4. GuangDong-Euc.	*C. kyotensis*	*C. aconidialis*	AR----	CSF23341	20210607-1-(181)	OQ188846	OQ260821	–	–	–	–
4. GuangDong-Euc.	*C. kyotensis*	*C. aconidialis*	AR----	CSF23355	20210607-1-(197)	OQ188847	OQ260822	–	–	–	–
4. GuangDong-Euc.	*C. kyotensis*	*C. aconidialis*	ARAAAA	CSF23251	20210607-1-(79)	OQ188848	OQ260823	OQ261489	OQ302924	OQ303131	OQ303337
4. GuangDong-Euc.	*C. kyotensis*	*C. aconidialis*	ASACAA	CSF22524	20210607-1-(1)	OQ188849	OQ260824	OQ261492	OQ302927	OQ303134	OQ303340
4. GuangDong-Euc.	*C. kyotensis*	*C. aconidialis*	AT----	CSF22535	20210607-1-(17)	OQ188850	OQ260825	–	–	–	–
4. GuangDong-Euc.	*C. kyotensis*	*C. aconidialis*	AT----	CSF22539	20210607-1-(21)	OQ188851	OQ260826	–	–	–	–
4. GuangDong-Euc.	*C. kyotensis*	*C. aconidialis*	AT----	CSF23214	20210607-1-(39)	OQ188852	OQ260827	–	–	–	–
4. GuangDong-Euc.	*C. kyotensis*	*C. aconidialis*	AT----	CSF23227	20210607-1-(54)	OQ188853	OQ260828	–	–	–	–
4. GuangDong-Euc.	*C. kyotensis*	*C. aconidialis*	AT----	CSF23237	20210607-1-(64)	OQ188854	OQ260829	–	–	–	–
4. GuangDong-Euc.	*C. kyotensis*	*C. aconidialis*	AT----	CSF23240	20210607-1-(67)	OQ188855	OQ260830	–	–	–	–
4. GuangDong-Euc.	*C. kyotensis*	*C. aconidialis*	AT----	CSF23245	20210607-1-(72)	OQ188856	OQ260831	–	–	–	–
4. GuangDong-Euc.	*C. kyotensis*	*C. aconidialis*	AT----	CSF23247	20210607-1-(75)	OQ188857	OQ260832	–	–	–	–
4. GuangDong-Euc.	*C. kyotensis*	*C. aconidialis*	AT----	CSF23250	20210607-1-(78)	OQ188858	OQ260833	–	–	–	–
4. GuangDong-Euc.	*C. kyotensis*	*C. aconidialis*	AT----	CSF23262	20210607-1-(90)	OQ188859	OQ260834	–	–	–	–
4. GuangDong-Euc.	*C. kyotensis*	*C. aconidialis*	AT----	CSF23263	20210607-1-(91)	OQ188860	OQ260835	–	–	–	–
4. GuangDong-Euc.	*C. kyotensis*	*C. aconidialis*	AT----	CSF23269	20210607-1-(97)	OQ188861	OQ260836	–	–	–	–
4. GuangDong-Euc.	*C. kyotensis*	*C. aconidialis*	AT----	CSF23287	20210607-1-(115)	OQ188862	OQ260837	–	–	–	–
4. GuangDong-Euc.	*C. kyotensis*	*C. aconidialis*	AT----	CSF23301	20210607-1-(135)	OQ188863	OQ260838	–	–	–	–
4. GuangDong-Euc.	*C. kyotensis*	*C. aconidialis*	AT----	CSF23304	20210607-1-(139)	OQ188864	OQ260839	–	–	–	–
4. GuangDong-Euc.	*C. kyotensis*	*C. aconidialis*	AT----	CSF23309	20210607-1-(144)	OQ188865	OQ260840	–	–	–	–
4. GuangDong-Euc.	*C. kyotensis*	*C. aconidialis*	AT----	CSF23315	20210607-1-(152)	OQ188866	OQ260841	–	–	–	–
4. GuangDong-Euc.	*C. kyotensis*	*C. aconidialis*	AT----	CSF23321	20210607-1-(159)	OQ188867	OQ260842	–	–	–	–
4. GuangDong-Euc.	*C. kyotensis*	*C. aconidialis*	AT----	CSF23323	20210607-1-(162)	OQ188868	OQ260843	–	–	–	–
4. GuangDong-Euc.	*C. kyotensis*	*C. aconidialis*	AT----	CSF23324	20210607-1-(163)	OQ188869	OQ260844	–	–	–	–
4. GuangDong-Euc.	*C. kyotensis*	*C. aconidialis*	AT----	CSF23327	20210607-1-(166)	OQ188870	OQ260845	–	–	–	–
4. GuangDong-Euc.	*C. kyotensis*	*C. aconidialis*	AT----	CSF23329	20210607-1-(168)	OQ188871	OQ260846	–	–	–	–
4. GuangDong-Euc.	*C. kyotensis*	*C. aconidialis*	AT----	CSF23337	20210607-1-(177)	OQ188872	OQ260847	–	–	–	–
4. GuangDong-Euc.	*C. kyotensis*	*C. aconidialis*	AT----	CSF23339	20210607-1-(179)	OQ188873	OQ260848	–	–	–	–
4. GuangDong-Euc.	*C. kyotensis*	*C. aconidialis*	AT----	CSF23346	20210607-1-(187)	OQ188874	OQ260849	–	–	–	–
4. GuangDong-Euc.	*C. kyotensis*	*C. aconidialis*	AT----	CSF23347	20210607-1-(188)	OQ188875	OQ260850	–	–	–	–
4. GuangDong-Euc.	*C. kyotensis*	*C. aconidialis*	AT----	CSF23357	20210607-1-(199)	OQ188876	OQ260851	–	–	–	–
4. GuangDong-Euc.	*C. kyotensis*	*C. aconidialis*	AT----	CSF23358	20210607-1-(200)	OQ188877	OQ260852	–	–	–	–
4. GuangDong-Euc.	*C. kyotensis*	*C. aconidialis*	AT----	CSF23384	20210607-1-(236)	OQ188878	OQ260853	–	–	–	–
4. GuangDong-Euc.	*C. kyotensis*	*C. aconidialis*	AT----	CSF23390	20210607-1-(243)	OQ188879	OQ260854	–	–	–	–
4. GuangDong-Euc.	*C. kyotensis*	*C. aconidialis*	AT----	CSF23394	20210607-1-(248)	OQ188880	OQ260855	–	–	–	–
4. GuangDong-Euc.	*C. kyotensis*	*C. aconidialis*	GA----	CSF23294	20210607-1-(127)	OQ188881	OQ260856	–	–	–	–
4. GuangDong-Euc.	*C. kyotensis*	*C. aconidialis*	GA----	CSF23302	20210607-1-(137)	OQ188882	OQ260857	–	–	–	–
4. GuangDong-Euc.	*C. kyotensis*	*C. aconidialis*	GAAAAA	CSF23306	20210607-1-(141)	OQ188883	OQ260858	OQ261510	OQ302945	OQ303152	OQ303358
4. GuangDong-Euc.	*C. kyotensis*	*C. aconidialis*	GO----	CSF23325	20210607-1-(164)	OQ188884	OQ260859	–	–	–	–
4. GuangDong-Euc.	*C. kyotensis*	*C. aconidialis*	GOCAAA	CSF23221	20210607-1-(46)	OQ188885	OQ260860	OQ261512	OQ302947	OQ303154	OQ303360
4. GuangDong-Euc.	*C. kyotensis*	*C. hongkongensis*	AA----	CSF22530	20210607-1-(9)	OQ188886	OQ260861	–	–	–	–
4. GuangDong-Euc.	*C. kyotensis*	*C. hongkongensis*	AA----	CSF22536	20210607-1-(18)	OQ188887	OQ260862	–	–	–	–
4. GuangDong-Euc.	*C. kyotensis*	*C. hongkongensis*	AA----	CSF23210	20210607-1-(35)	OQ188888	OQ260863	–	–	–	–
4. GuangDong-Euc.	*C. kyotensis*	*C. hongkongensis*	AA----	CSF23285	20210607-1-(113)	OQ188889	OQ260864	–	–	–	–
4. GuangDong-Euc.	*C. kyotensis*	*C. hongkongensis*	AA----	CSF23330	20210607-1-(169)	OQ188890	OQ260865	–	–	–	–
4. GuangDong-Euc.	*C. kyotensis*	*C. hongkongensis*	AA----	CSF23332	20210607-1-(171)	OQ188891	OQ260866	–	–	–	–
4. GuangDong-Euc.	*C. kyotensis*	*C. hongkongensis*	AB----	CSF23244	20210607-1-(71)	OQ188892	OQ260867	–	–	–	–
4. GuangDong-Euc.	*C. kyotensis*	*C. hongkongensis*	AB----	CSF23289	20210607-1-(118)	OQ188893	OQ260868	–	–	–	–
4. GuangDong-Euc.	*C. kyotensis*	*C. hongkongensis*	AC----	CSF23230	20210607-1-(57)	OQ188894	OQ260869	–	–	–	–
4. GuangDong-Euc.	*C. kyotensis*	*C. hongkongensis*	ACAAAA	CSF23258	20210607-1-(86)	OQ188895	OQ260870	OQ261528	OQ302963	OQ303170	OQ303376
4. GuangDong-Euc.	*C. kyotensis*	*C. hongkongensis*	AD----	CSF23375	20210607-1-(225)	OQ188896	OQ260871	–	–	–	–
4. GuangDong-Euc.	*C. kyotensis*	*C. hongkongensis*	AFABAA	CSF23366	20210607-1-(214)	OQ188897	OQ260872	OQ261539	OQ302974	OQ303181	OQ303387
4. GuangDong-Euc.	*C. kyotensis*	*C. ilicicola*	BAAAAA	CSF23220	20210607-1-(45)	OQ188898	OQ260873	OQ261554	OQ302989	OQ303195	OQ303402
4. GuangDong-Euc.	*C. kyotensis*	*C. kyotensis*	AIDAAA	CSF23316	20210607-1-(153)	OQ188899	OQ260874	OQ261568	OQ303003	OQ303209	OQ303416
4. GuangDong-Euc.	*C. kyotensis*	*C. kyotensis*	DDDAAA	CSF23382	20210607-1-(234)	OQ188900	OQ260875	OQ261630	OQ303065	OQ303271	OQ303478
4. GuangDong-Euc.	*C. kyotensis*	*C. kyotensis*	DIEAAA	CSF23389	20210607-1-(242)	OQ188901	OQ260876	OQ261636	OQ303071	OQ303277	OQ303484
4. GuangDong-Euc.	*C. kyotensis*	*C. kyotensis*	DVACAA	CSF23370	20210607-1-(219)	OQ188902	OQ260877	OQ261654	OQ303089	OQ303295	OQ303502
5. GuangDong-Pin.	*C. kyotensis*	*C. aconidialis*	AA----	CSF22547	20210606-1-(1)	OQ188903	OQ260878	–	–	–	–
5. GuangDong-Pin.	*C. kyotensis*	*C. aconidialis*	AA----	CSF22548	20210606-1-(2)	OQ188904	OQ260879	–	–	–	–
5. GuangDong-Pin.	*C. kyotensis*	*C. aconidialis*	AA----	CSF22549	20210606-1-(3)	OQ188905	OQ260880	–	–	–	–
5. GuangDong-Pin.	*C. kyotensis*	*C. aconidialis*	AA----	CSF22551	20210606-1-(5)	OQ188906	OQ260881	–	–	–	–
5. GuangDong-Pin.	*C. kyotensis*	*C. aconidialis*	AA----	CSF22553	20210606-1-(7)	OQ188907	OQ260882	–	–	–	–
5. GuangDong-Pin.	*C. kyotensis*	*C. aconidialis*	AA----	CSF22554	20210606-1-(8)	OQ188908	OQ260883	–	–	–	–
5. GuangDong-Pin.	*C. kyotensis*	*C. aconidialis*	AA----	CSF22556	20210606-1-(10)	OQ188909	OQ260884	–	–	–	–
5. GuangDong-Pin.	*C. kyotensis*	*C. aconidialis*	AA----	CSF22557	20210606-1-(11)	OQ188910	OQ260885	–	–	–	–
5. GuangDong-Pin.	*C. kyotensis*	*C. aconidialis*	AA----	CSF22561	20210606-1-(16)	OQ188911	OQ260886	–	–	–	–
5. GuangDong-Pin.	*C. kyotensis*	*C. aconidialis*	AA----	CSF22563	20210606-1-(18)	OQ188912	OQ260887	–	–	–	–
5. GuangDong-Pin.	*C. kyotensis*	*C. aconidialis*	AA----	CSF22564	20210606-1-(19)	OQ188913	OQ260888	–	–	–	–
5. GuangDong-Pin.	*C. kyotensis*	*C. aconidialis*	AA----	CSF22568	20210606-1-(24)	OQ188914	OQ260889	–	–	–	–
5. GuangDong-Pin.	*C. kyotensis*	*C. aconidialis*	AA----	CSF23439	20210606-1-(30)	OQ188915	OQ260890	–	–	–	–
5. GuangDong-Pin.	*C. kyotensis*	*C. aconidialis*	AA----	CSF23440	20210606-1-(31)	OQ188916	OQ260891	–	–	–	–
5. GuangDong-Pin.	*C. kyotensis*	*C. aconidialis*	AA----	CSF23441	20210606-1-(32)	OQ188917	OQ260892	–	–	–	–
5. GuangDong-Pin.	*C. kyotensis*	*C. aconidialis*	AA----	CSF23442	20210606-1-(33)	OQ188918	OQ260893	–	–	–	–
5. GuangDong-Pin.	*C. kyotensis*	*C. aconidialis*	AA----	CSF23446	20210606-1-(37)	OQ188919	OQ260894	–	–	–	–
5. GuangDong-Pin.	*C. kyotensis*	*C. aconidialis*	AA----	CSF23448	20210606-1-(39)	OQ188920	OQ260895	–	–	–	–
5. GuangDong-Pin.	*C. kyotensis*	*C. aconidialis*	AA----	CSF23453	20210606-1-(45)	OQ188921	OQ260896	–	–	–	–
5. GuangDong-Pin.	*C. kyotensis*	*C. aconidialis*	AA----	CSF23454	20210606-1-(46)	OQ188922	OQ260897	–	–	–	–
5. GuangDong-Pin.	*C. kyotensis*	*C. aconidialis*	AA----	CSF23457	20210606-1-(49)	OQ188923	OQ260898	–	–	–	–
5. GuangDong-Pin.	*C. kyotensis*	*C. aconidialis*	AA----	CSF23459	20210606-1-(51)	OQ188924	OQ260899	–	–	–	–
5. GuangDong-Pin.	*C. kyotensis*	*C. aconidialis*	AA----	CSF23461	20210606-1-(53)	OQ188925	OQ260900	–	–	–	–
5. GuangDong-Pin.	*C. kyotensis*	*C. aconidialis*	AA----	CSF23462	20210606-1-(54)	OQ188926	OQ260901	–	–	–	–
5. GuangDong-Pin.	*C. kyotensis*	*C. aconidialis*	AA----	CSF23463	20210606-1-(55)	OQ188927	OQ260902	–	–	–	–
5. GuangDong-Pin.	*C. kyotensis*	*C. aconidialis*	AA----	CSF23466	20210606-1-(58)	OQ188928	OQ260903	–	–	–	–
5. GuangDong-Pin.	*C. kyotensis*	*C. aconidialis*	AA----	CSF23467	20210606-1-(59)	OQ188929	OQ260904	–	–	–	–
5. GuangDong-Pin.	*C. kyotensis*	*C. aconidialis*	AA----	CSF23479	20210606-1-(78)	OQ188930	OQ260905	–	–	–	–
5. GuangDong-Pin.	*C. kyotensis*	*C. aconidialis*	AA----	CSF23482	20210606-1-(81)	OQ188931	OQ260906	–	–	–	–
5. GuangDong-Pin.	*C. kyotensis*	*C. aconidialis*	AA----	CSF23484	20210606-1-(83)	OQ188932	OQ260907	–	–	–	–
5. GuangDong-Pin.	*C. kyotensis*	*C. aconidialis*	AA----	CSF23485	20210606-1-(84)	OQ188933	OQ260908	–	–	–	–
5. GuangDong-Pin.	*C. kyotensis*	*C. aconidialis*	AA----	CSF23487	20210606-1-(86)	OQ188934	OQ260909	–	–	–	–
5. GuangDong-Pin.	*C. kyotensis*	*C. aconidialis*	AA----	CSF23488	20210606-1-(87)	OQ188935	OQ260910	–	–	–	–
5. GuangDong-Pin.	*C. kyotensis*	*C. aconidialis*	AA----	CSF23491	20210606-1-(90)	OQ188936	OQ260911	–	–	–	–
5. GuangDong-Pin.	*C. kyotensis*	*C. aconidialis*	AA----	CSF23492	20210606-1-(91)	OQ188937	OQ260912	–	–	–	–
5. GuangDong-Pin.	*C. kyotensis*	*C. aconidialis*	AA----	CSF23496	20210606-1-(95)	OQ188938	OQ260913	–	–	–	–
5. GuangDong-Pin.	*C. kyotensis*	*C. aconidialis*	AA----	CSF23514	20210606-1-(118)	OQ188939	OQ260914	–	–	–	–
5. GuangDong-Pin.	*C. kyotensis*	*C. aconidialis*	AA----	CSF23515	20210606-1-(120)	OQ188940	OQ260915	–	–	–	–
5. GuangDong-Pin.	*C. kyotensis*	*C. aconidialis*	AA----	CSF23517	20210606-1-(124)	OQ188941	OQ260916	–	–	–	–
5. GuangDong-Pin.	*C. kyotensis*	*C. aconidialis*	AA----	CSF23518	20210606-1-(126)	OQ188942	OQ260917	–	–	–	–
5. GuangDong-Pin.	*C. kyotensis*	*C. aconidialis*	AA----	CSF23519	20210606-1-(127)	OQ188943	OQ260918	–	–	–	–
5. GuangDong-Pin.	*C. kyotensis*	*C. aconidialis*	AA----	CSF23520	20210606-1-(129)	OQ188944	OQ260919	–	–	–	–
5. GuangDong-Pin.	*C. kyotensis*	*C. aconidialis*	AA----	CSF23521	20210606-1-(130)	OQ188945	OQ260920	–	–	–	–
5. GuangDong-Pin.	*C. kyotensis*	*C. aconidialis*	AA----	CSF23524	20210606-1-(132)	OQ188946	OQ260921	–	–	–	–
5. GuangDong-Pin.	*C. kyotensis*	*C. aconidialis*	AA----	CSF23526	20210606-1-(135)	OQ188947	OQ260922	–	–	–	–
5. GuangDong-Pin.	*C. kyotensis*	*C. aconidialis*	AA----	CSF23527	20210606-1-(136)	OQ188948	OQ260923	–	–	–	–
5. GuangDong-Pin.	*C. kyotensis*	*C. aconidialis*	AA----	CSF23528	20210606-1-(137)	OQ188949	OQ260924	–	–	–	–
5. GuangDong-Pin.	*C. kyotensis*	*C. aconidialis*	AA----	CSF23529	20210606-1-(138)	OQ188950	OQ260925	–	–	–	–
5. GuangDong-Pin.	*C. kyotensis*	*C. aconidialis*	AA----	CSF23531	20210606-1-(140)	OQ188951	OQ260926	–	–	–	–
5. GuangDong-Pin.	*C. kyotensis*	*C. aconidialis*	AA----	CSF23532	20210606-1-(141)	OQ188952	OQ260927	–	–	–	–
5. GuangDong-Pin.	*C. kyotensis*	*C. aconidialis*	AA----	CSF23536	20210606-1-(144)	OQ188953	OQ260928	–	–	–	–
5. GuangDong-Pin.	*C. kyotensis*	*C. aconidialis*	AA----	CSF23537	20210606-1-(145)	OQ188954	OQ260929	–	–	–	–
5. GuangDong-Pin.	*C. kyotensis*	*C. aconidialis*	AA----	CSF23538	20210606-1-(146)	OQ188955	OQ260930	–	–	–	–
5. GuangDong-Pin.	*C. kyotensis*	*C. aconidialis*	AA----	CSF23540	20210606-1-(148)	OQ188956	OQ260931	–	–	–	–
5. GuangDong-Pin.	*C. kyotensis*	*C. aconidialis*	AA----	CSF23542	20210606-1-(150)	OQ188957	OQ260932	–	–	–	–
5. GuangDong-Pin.	*C. kyotensis*	*C. aconidialis*	AA----	CSF23544	20210606-1-(152)	OQ188958	OQ260933	–	–	–	–
5. GuangDong-Pin.	*C. kyotensis*	*C. aconidialis*	AA----	CSF23545	20210606-1-(154)	OQ188959	OQ260934	–	–	–	–
5. GuangDong-Pin.	*C. kyotensis*	*C. aconidialis*	AA----	CSF23546	20210606-1-(155)	OQ188960	OQ260935	–	–	–	–
5. GuangDong-Pin.	*C. kyotensis*	*C. aconidialis*	AA----	CSF23551	20210606-1-(160)	OQ188961	OQ260936	–	–	–	–
5. GuangDong-Pin.	*C. kyotensis*	*C. aconidialis*	AA----	CSF23552	20210606-1-(161)	OQ188962	OQ260937	–	–	–	–
5. GuangDong-Pin.	*C. kyotensis*	*C. aconidialis*	AA----	CSF23556	20210606-1-(165)	OQ188963	OQ260938	–	–	–	–
5. GuangDong-Pin.	*C. kyotensis*	*C. aconidialis*	AA----	CSF23559	20210606-1-(168)	OQ188964	OQ260939	–	–	–	–
5. GuangDong-Pin.	*C. kyotensis*	*C. aconidialis*	AA----	CSF23560	20210606-1-(169)	OQ188965	OQ260940	–	–	–	–
5. GuangDong-Pin.	*C. kyotensis*	*C. aconidialis*	AA----	CSF23564	20210606-1-(174)	OQ188966	OQ260941	–	–	–	–
5. GuangDong-Pin.	*C. kyotensis*	*C. aconidialis*	AA----	CSF23566	20210606-1-(176)	OQ188967	OQ260942	–	–	–	–
5. GuangDong-Pin.	*C. kyotensis*	*C. aconidialis*	AA----	CSF23568	20210606-1-(178)	OQ188968	OQ260943	–	–	–	–
5. GuangDong-Pin.	*C. kyotensis*	*C. aconidialis*	AA----	CSF23569	20210606-1-(179)	OQ188969	OQ260944	–	–	–	–
5. GuangDong-Pin.	*C. kyotensis*	*C. aconidialis*	AA----	CSF23570	20210606-1-(180)	OQ188970	OQ260945	–	–	–	–
5. GuangDong-Pin.	*C. kyotensis*	*C. aconidialis*	AA----	CSF23571	20210606-1-(181)	OQ188971	OQ260946	–	–	–	–
5. GuangDong-Pin.	*C. kyotensis*	*C. aconidialis*	AA----	CSF23573	20210606-1-(183)	OQ188972	OQ260947	–	–	–	–
5. GuangDong-Pin.	*C. kyotensis*	*C. aconidialis*	AA----	CSF23574	20210606-1-(184)	OQ188973	OQ260948	–	–	–	–
5. GuangDong-Pin.	*C. kyotensis*	*C. aconidialis*	AA----	CSF23575	20210606-1-(185)	OQ188974	OQ260949	–	–	–	–
5. GuangDong-Pin.	*C. kyotensis*	*C. aconidialis*	AA----	CSF23577	20210606-1-(187)	OQ188975	OQ260950	–	–	–	–
5. GuangDong-Pin.	*C. kyotensis*	*C. aconidialis*	AA----	CSF23578	20210606-1-(188)	OQ188976	OQ260951	–	–	–	–
5. GuangDong-Pin.	*C. kyotensis*	*C. aconidialis*	AA----	CSF23583	20210606-1-(195)	OQ188977	OQ260952	–	–	–	–
5. GuangDong-Pin.	*C. kyotensis*	*C. aconidialis*	AA----	CSF23586	20210606-1-(200)	OQ188978	OQ260953	–	–	–	–
5. GuangDong-Pin.	*C. kyotensis*	*C. aconidialis*	AA----	CSF23588	20210606-1-(202)	OQ188979	OQ260954	–	–	–	–
5. GuangDong-Pin.	*C. kyotensis*	*C. aconidialis*	AA----	CSF23589	20210606-1-(204)	OQ188980	OQ260955	–	–	–	–
5. GuangDong-Pin.	*C. kyotensis*	*C. aconidialis*	AA----	CSF23591	20210606-1-(206)	OQ188981	OQ260956	–	–	–	–
5. GuangDong-Pin.	*C. kyotensis*	*C. aconidialis*	AA----	CSF23592	20210606-1-(207)	OQ188982	OQ260957	–	–	–	–
5. GuangDong-Pin.	*C. kyotensis*	*C. aconidialis*	AA----	CSF23594	20210606-1-(209)	OQ188983	OQ260958	–	–	–	–
5. GuangDong-Pin.	*C. kyotensis*	*C. aconidialis*	AA----	CSF23595	20210606-1-(210)	OQ188984	OQ260959	–	–	–	–
5. GuangDong-Pin.	*C. kyotensis*	*C. aconidialis*	AA----	CSF23597	20210606-1-(212)	OQ188985	OQ260960	–	–	–	–
5. GuangDong-Pin.	*C. kyotensis*	*C. aconidialis*	AA----	CSF23598	20210606-1-(213)	OQ188986	OQ260961	–	–	–	–
5. GuangDong-Pin.	*C. kyotensis*	*C. aconidialis*	AA----	CSF23601	20210606-1-(216)	OQ188987	OQ260962	–	–	–	–
5. GuangDong-Pin.	*C. kyotensis*	*C. aconidialis*	AA----	CSF23604	20210606-1-(219)	OQ188988	OQ260963	–	–	–	–
5. GuangDong-Pin.	*C. kyotensis*	*C. aconidialis*	AA----	CSF23607	20210606-1-(222)	OQ188989	OQ260964	–	–	–	–
5. GuangDong-Pin.	*C. kyotensis*	*C. aconidialis*	AA----	CSF23609	20210606-1-(224)	OQ188990	OQ260965	–	–	–	–
5. GuangDong-Pin.	*C. kyotensis*	*C. aconidialis*	AA----	CSF23610	20210606-1-(225)	OQ188991	OQ260966	–	–	–	–
5. GuangDong-Pin.	*C. kyotensis*	*C. aconidialis*	AA----	CSF23612	20210606-1-(227)	OQ188992	OQ260967	–	–	–	–
5. GuangDong-Pin.	*C. kyotensis*	*C. aconidialis*	AA----	CSF23615	20210606-1-(230)	OQ188993	OQ260968	–	–	–	–
5. GuangDong-Pin.	*C. kyotensis*	*C. aconidialis*	AA----	CSF23617	20210606-1-(232)	OQ188994	OQ260969	–	–	–	–
5. GuangDong-Pin.	*C. kyotensis*	*C. aconidialis*	AA----	CSF23619	20210606-1-(234)	OQ188995	OQ260970	–	–	–	–
5. GuangDong-Pin.	*C. kyotensis*	*C. aconidialis*	AA----	CSF23620	20210606-1-(235)	OQ188996	OQ260971	–	–	–	–
5. GuangDong-Pin.	*C. kyotensis*	*C. aconidialis*	AA----	CSF23622	20210606-1-(237)	OQ188997	OQ260972	–	–	–	–
5. GuangDong-Pin.	*C. kyotensis*	*C. aconidialis*	AA----	CSF23624	20210606-1-(239)	OQ188998	OQ260973	–	–	–	–
5. GuangDong-Pin.	*C. kyotensis*	*C. aconidialis*	AA----	CSF23625	20210606-1-(240)	OQ188999	OQ260974	–	–	–	–
5. GuangDong-Pin.	*C. kyotensis*	*C. aconidialis*	AA----	CSF23626	20210606-1-(241)	OQ189000	OQ260975	–	–	–	–
5. GuangDong-Pin.	*C. kyotensis*	*C. aconidialis*	AA----	CSF23628	20210606-1-(244)	OQ189001	OQ260976	–	–	–	–
5. GuangDong-Pin.	*C. kyotensis*	*C. aconidialis*	AA----	CSF23629	20210606-1-(245)	OQ189002	OQ260977	–	–	–	–
5. GuangDong-Pin.	*C. kyotensis*	*C. aconidialis*	AA----	CSF23630	20210606-1-(246)	OQ189003	OQ260978	–	–	–	–
5. GuangDong-Pin.	*C. kyotensis*	*C. aconidialis*	AA----	CSF23632	20210606-1-(248)	OQ189004	OQ260979	–	–	–	–
5. GuangDong-Pin.	*C. kyotensis*	*C. aconidialis*	AA----	CSF23634	20210606-1-(250)	OQ189005	OQ260980	–	–	–	–
5. GuangDong-Pin.	*C. kyotensis*	*C. aconidialis*	AB----	CSF23445	20210606-1-(36)	OQ189006	OQ260981	–	–	–	–
5. GuangDong-Pin.	*C. kyotensis*	*C. aconidialis*	ABAAAA	CSF22562	20210606-1-(17)	OQ189007	OQ260982	OQ261465	OQ302900	OQ303107	OQ303313
5. GuangDong-Pin.	*C. kyotensis*	*C. aconidialis*	AF----	CSF23490	20210606-1-(89)	OQ189008	OQ260983	–	–	–	–
5. GuangDong-Pin.	*C. kyotensis*	*C. aconidialis*	AF----	CSF23495	20210606-1-(94)	OQ189009	OQ260984	–	–	–	–
5. GuangDong-Pin.	*C. kyotensis*	*C. aconidialis*	AF----	CSF23530	20210606-1-(139)	OQ189010	OQ260985	–	–	–	–
5. GuangDong-Pin.	*C. kyotensis*	*C. aconidialis*	AF----	CSF23603	20210606-1-(218)	OQ189011	OQ260986	–	–	–	–
5. GuangDong-Pin.	*C. kyotensis*	*C. aconidialis*	AF----	CSF23627	20210606-1-(242)	OQ189012	OQ260987	–	–	–	–
5. GuangDong-Pin.	*C. kyotensis*	*C. aconidialis*	AF----	CSF23631	20210606-1-(247)	OQ189013	OQ260988	–	–	–	–
5. GuangDong-Pin.	*C. kyotensis*	*C. aconidialis*	AFEAAA	CSF23623	20210606-1-(238)	OQ189014	OQ260989	OQ261472	OQ302907	OQ303114	OQ303320
5. GuangDong-Pin.	*C. kyotensis*	*C. aconidialis*	AHAAAA	CSF23621	20210606-1-(236)	OQ189015	OQ260990	OQ261474	OQ302909	OQ303116	OQ303322
5. GuangDong-Pin.	*C. kyotensis*	*C. aconidialis*	AJAAAA	CSF23585	20210606-1-(198)	OQ189016	OQ260991	OQ261478	OQ302913	OQ303120	OQ303326
5. GuangDong-Pin.	*C. kyotensis*	*C. aconidialis*	ARAAAA	CSF23444	20210606-1-(35)	OQ189017	OQ260992	OQ261490	OQ302925	OQ303132	OQ303338
5. GuangDong-Pin.	*C. kyotensis*	*C. aconidialis*	ASAAAA	CSF23497	20210606-1-(96)	OQ189018	OQ260993	OQ261491	OQ302926	OQ303133	OQ303339
5. GuangDong-Pin.	*C. kyotensis*	*C. aconidialis*	AT----	CSF23458	20210606-1-(50)	OQ189019	OQ260994	–	–	–	–
5. GuangDong-Pin.	*C. kyotensis*	*C. aconidialis*	AT----	CSF23464	20210606-1-(56)	OQ189020	OQ260995	–	–	–	–
5. GuangDong-Pin.	*C. kyotensis*	*C. aconidialis*	AT----	CSF23483	20210606-1-(82)	OQ189021	OQ260996	–	–	–	–
5. GuangDong-Pin.	*C. kyotensis*	*C. aconidialis*	AT----	CSF23493	20210606-1-(92)	OQ189022	OQ260997	–	–	–	–
5. GuangDong-Pin.	*C. kyotensis*	*C. aconidialis*	AT----	CSF23533	20210606-1-(142)	OQ189023	OQ260998	–	–	–	–
5. GuangDong-Pin.	*C. kyotensis*	*C. aconidialis*	AT----	CSF23539	20210606-1-(147)	OQ189024	OQ260999	–	–	–	–
5. GuangDong-Pin.	*C. kyotensis*	*C. aconidialis*	AT----	CSF23549	20210606-1-(158)	OQ189025	OQ261000	–	–	–	–
5. GuangDong-Pin.	*C. kyotensis*	*C. aconidialis*	AT----	CSF23553	20210606-1-(162)	OQ189026	OQ261001	–	–	–	–
5. GuangDong-Pin.	*C. kyotensis*	*C. aconidialis*	AT----	CSF23557	20210606-1-(166)	OQ189027	OQ261002	–	–	–	–
5. GuangDong-Pin.	*C. kyotensis*	*C. aconidialis*	AT----	CSF23561	20210606-1-(170)	OQ189028	OQ261003	–	–	–	–
5. GuangDong-Pin.	*C. kyotensis*	*C. aconidialis*	AT----	CSF23565	20210606-1-(175)	OQ189029	OQ261004	–	–	–	–
5. GuangDong-Pin.	*C. kyotensis*	*C. aconidialis*	AT----	CSF23567	20210606-1-(177)	OQ189030	OQ261005	–	–	–	–
5. GuangDong-Pin.	*C. kyotensis*	*C. aconidialis*	AT----	CSF23579	20210606-1-(189)	OQ189031	OQ261006	–	–	–	–
5. GuangDong-Pin.	*C. kyotensis*	*C. aconidialis*	AT----	CSF23613	20210606-1-(228)	OQ189032	OQ261007	–	–	–	–
5. GuangDong-Pin.	*C. kyotensis*	*C. aconidialis*	AT----	CSF23616	20210606-1-(231)	OQ189033	OQ261008	–	–	–	–
5. GuangDong-Pin.	*C. kyotensis*	*C. aconidialis*	GA----	CSF22558	20210606-1-(12)	OQ189034	OQ261009	–	–	–	–
5. GuangDong-Pin.	*C. kyotensis*	*C. aconidialis*	GA----	CSF23548	20210606-1-(157)	OQ189035	OQ261010	–	–	–	–
5. GuangDong-Pin.	*C. kyotensis*	*C. aconidialis*	GAAAAA	CSF23563	20210606-1-(173)	OQ189036	OQ261011	OQ261511	OQ302946	OQ303153	OQ303359
5. GuangDong-Pin.	*C. kyotensis*	*C. aconidialis*	GOCAAA	CSF23547	20210606-1-(156)	OQ189037	OQ261012	OQ261513	OQ302948	OQ303155	OQ303361
5. GuangDong-Pin.	*C. kyotensis*	*C. curvispora*	AA----	CSF22560	20210606-1-(15)	OQ189038	OQ261013	–	–	–	–
5. GuangDong-Pin.	*C. kyotensis*	*C. curvispora*	AA----	CSF22566	20210606-1-(21)	OQ189039	OQ261014	–	–	–	–
5. GuangDong-Pin.	*C. kyotensis*	*C. curvispora*	AAAAAA	CSF22555	20210606-1-(9)	OQ189040	OQ261015	OQ261522	OQ302957	OQ303164	OQ303370
5. GuangDong-Pin.	*C. kyotensis*	*C. curvispora*	AAAAAA	CSF23447	20210606-1-(38)	OQ189041	OQ261016	OQ261523	OQ302958	OQ303165	OQ303371
5. GuangDong-Pin.	*C. kyotensis*	*C. hongkongensis*	AA----	CSF22565	20210606-1-(20)	OQ189042	OQ261017	–	–	–	–
5. GuangDong-Pin.	*C. kyotensis*	*C. hongkongensis*	AA----	CSF22567	20210606-1-(22)	OQ189043	OQ261018	–	–	–	–
5. GuangDong-Pin.	*C. kyotensis*	*C. hongkongensis*	AA----	CSF22569	20210606-1-(25)	OQ189044	OQ261019	–	–	–	–
5. GuangDong-Pin.	*C. kyotensis*	*C. hongkongensis*	AA----	CSF23438	20210606-1-(28)	OQ189045	OQ261020	–	–	–	–
5. GuangDong-Pin.	*C. kyotensis*	*C. hongkongensis*	AA----	CSF23449	20210606-1-(40)	OQ189046	OQ261021	–	–	–	–
5. GuangDong-Pin.	*C. kyotensis*	*C. hongkongensis*	AA----	CSF23451	20210606-1-(42)	OQ189047	OQ261022	–	–	–	–
5. GuangDong-Pin.	*C. kyotensis*	*C. hongkongensis*	AA----	CSF23456	20210606-1-(48)	OQ189048	OQ261023	–	–	–	–
5. GuangDong-Pin.	*C. kyotensis*	*C. hongkongensis*	AA----	CSF23465	20210606-1-(57)	OQ189049	OQ261024	–	–	–	–
5. GuangDong-Pin.	*C. kyotensis*	*C. hongkongensis*	AA----	CSF23469	20210606-1-(61)	OQ189050	OQ261025	–	–	–	–
5. GuangDong-Pin.	*C. kyotensis*	*C. hongkongensis*	AA----	CSF23472	20210606-1-(68)	OQ189051	OQ261026	–	–	–	–
5. GuangDong-Pin.	*C. kyotensis*	*C. hongkongensis*	AA----	CSF23473	20210606-1-(69)	OQ189052	OQ261027	–	–	–	–
5. GuangDong-Pin.	*C. kyotensis*	*C. hongkongensis*	AA----	CSF23478	20210606-1-(75)	OQ189053	OQ261028	–	–	–	–
5. GuangDong-Pin.	*C. kyotensis*	*C. hongkongensis*	AA----	CSF23498	20210606-1-(97)	OQ189054	OQ261029	–	–	–	–
5. GuangDong-Pin.	*C. kyotensis*	*C. hongkongensis*	AA----	CSF23500	20210606-1-(99)	OQ189055	OQ261030	–	–	–	–
5. GuangDong-Pin.	*C. kyotensis*	*C. hongkongensis*	AA----	CSF23502	20210606-1-(101)	OQ189056	OQ261031	–	–	–	–
5. GuangDong-Pin.	*C. kyotensis*	*C. hongkongensis*	AA----	CSF23508	20210606-1-(110)	OQ189057	OQ261032	–	–	–	–
5. GuangDong-Pin.	*C. kyotensis*	*C. hongkongensis*	AA----	CSF23510	20210606-1-(114)	OQ189058	OQ261033	–	–	–	–
5. GuangDong-Pin.	*C. kyotensis*	*C. hongkongensis*	AA----	CSF23511	20210606-1-(115)	OQ189059	OQ261034	–	–	–	–
5. GuangDong-Pin.	*C. kyotensis*	*C. hongkongensis*	AA----	CSF23513	20210606-1-(117)	OQ189060	OQ261035	–	–	–	–
5. GuangDong-Pin.	*C. kyotensis*	*C. hongkongensis*	AA----	CSF23525	20210606-1-(134)	OQ189061	OQ261036	–	–	–	–
5. GuangDong-Pin.	*C. kyotensis*	*C. hongkongensis*	AA----	CSF23541	20210606-1-(149)	OQ189062	OQ261037	–	–	–	–
5. GuangDong-Pin.	*C. kyotensis*	*C. hongkongensis*	AA----	CSF23550	20210606-1-(159)	OQ189063	OQ261038	–	–	–	–
5. GuangDong-Pin.	*C. kyotensis*	*C. hongkongensis*	AA----	CSF23558	20210606-1-(167)	OQ189064	OQ261039	–	–	–	–
5. GuangDong-Pin.	*C. kyotensis*	*C. hongkongensis*	AA----	CSF23562	20210606-1-(172)	OQ189065	OQ261040	–	–	–	–
5. GuangDong-Pin.	*C. kyotensis*	*C. hongkongensis*	AA----	CSF23576	20210606-1-(186)	OQ189066	OQ261041	–	–	–	–
5. GuangDong-Pin.	*C. kyotensis*	*C. hongkongensis*	AA----	CSF23587	20210606-1-(201)	OQ189067	OQ261042	–	–	–	–
5. GuangDong-Pin.	*C. kyotensis*	*C. hongkongensis*	AA----	CSF23590	20210606-1-(205)	OQ189068	OQ261043	–	–	–	–
5. GuangDong-Pin.	*C. kyotensis*	*C. hongkongensis*	AA----	CSF23593	20210606-1-(208)	OQ189069	OQ261044	–	–	–	–
5. GuangDong-Pin.	*C. kyotensis*	*C. hongkongensis*	AA----	CSF23596	20210606-1-(211)	OQ189070	OQ261045	–	–	–	–
5. GuangDong-Pin.	*C. kyotensis*	*C. hongkongensis*	AA----	CSF23599	20210606-1-(214)	OQ189071	OQ261046	–	–	–	–
5. GuangDong-Pin.	*C. kyotensis*	*C. hongkongensis*	AA----	CSF23600	20210606-1-(215)	OQ189072	OQ261047	–	–	–	–
5. GuangDong-Pin.	*C. kyotensis*	*C. hongkongensis*	AA----	CSF23605	20210606-1-(220)	OQ189073	OQ261048	–	–	–	–
5. GuangDong-Pin.	*C. kyotensis*	*C. hongkongensis*	AA----	CSF23611	20210606-1-(226)	OQ189074	OQ261049	–	–	–	–
5. GuangDong-Pin.	*C. kyotensis*	*C. hongkongensis*	AA----	CSF23633	20210606-1-(249)	OQ189075	OQ261050	–	–	–	–
5. GuangDong-Pin.	*C. kyotensis*	*C. hongkongensis*	AAAAAA	CSF22552	20210606-1-(6)	OQ189076	OQ261051	OQ261525	OQ302960	OQ303167	OQ303373
5. GuangDong-Pin.	*C. kyotensis*	*C. hongkongensis*	AB----	CSF22550	20210606-1-(4)	OQ189077	OQ261052	–	–	–	–
5. GuangDong-Pin.	*C. kyotensis*	*C. hongkongensis*	AB----	CSF23522	20210606-1-(131)	OQ189078	OQ261053	–	–	–	–
5. GuangDong-Pin.	*C. kyotensis*	*C. hongkongensis*	ABBAAA	CSF23580	20210606-1-(191)	OQ189079	OQ261054	OQ261527	OQ302962	OQ303169	OQ303375
5. GuangDong-Pin.	*C. kyotensis*	*C. hongkongensis*	ACAAAA	CSF23476	20210606-1-(73)	OQ189080	OQ261055	OQ261529	OQ302964	OQ303171	OQ303377
5. GuangDong-Pin.	*C. kyotensis*	*C. hongkongensis*	AD----	CSF23460	20210606-1-(52)	OQ189081	OQ261056	–	–	–	–
5. GuangDong-Pin.	*C. kyotensis*	*C. hongkongensis*	AD----	CSF23516	20210606-1-(123)	OQ189082	OQ261057	–	–	–	–
5. GuangDong-Pin.	*C. kyotensis*	*C. hongkongensis*	ADBAAB	CSF23471	20210606-1-(64)	OQ189083	OQ261058	OQ261531	OQ302966	OQ303173	OQ303379
5. GuangDong-Pin.	*C. kyotensis*	*C. hongkongensis*	AE----	CSF23606	20210606-1-(221)	OQ189084	OQ261059	–	–	–	–
5. GuangDong-Pin.	*C. kyotensis*	*C. hongkongensis*	AEBAAA	CSF23443	20210606-1-(34)	OQ189085	OQ261060	OQ261533	OQ302968	OQ303175	OQ303381
5. GuangDong-Pin.	*C. kyotensis*	*C. hongkongensis*	AF----	CSF22559	20210606-1-(14)	OQ189086	OQ261061	–	–	–	–
5. GuangDong-Pin.	*C. kyotensis*	*C. hongkongensis*	AF----	CSF23486	20210606-1-(85)	OQ189087	OQ261062	–	–	–	–
5. GuangDong-Pin.	*C. kyotensis*	*C. hongkongensis*	AF----	CSF23503	20210606-1-(102)	OQ189088	OQ261063	–	–	–	–
5. GuangDong-Pin.	*C. kyotensis*	*C. hongkongensis*	AFAAAA	CSF23470	20210606-1-(63)	OQ189089	OQ261064	OQ261537	OQ302972	OQ303179	OQ303385
5. GuangDong-Pin.	*C. kyotensis*	*C. hongkongensis*	AFBAAA	CSF23602	20210606-1-(217)	OQ189090	OQ261065	OQ261542	OQ302977	OQ303184	OQ303390
5. GuangDong-Pin.	*C. kyotensis*	*C. hongkongensis*	AHAAAA	CSF23506	20210606-1-(108)	OQ189091	OQ261066	OQ261548	OQ302983	OQ303190	OQ303396
5. GuangDong-Pin.	*C. kyotensis*	*C. ilicicola*	BA----	CSF23501	20210606-1-(100)	OQ189092	OQ261067	–	–	–	–
5. GuangDong-Pin.	*C. kyotensis*	*C. ilicicola*	BAAAAA	CSF23489	20210606-1-(88)	OQ189093	OQ261068	OQ261555	OQ302990	OQ303196	OQ303403
5. GuangDong-Pin.	*C. kyotensis*	*C. kyotensis*	AA----	CSF23554	20210606-1-(163)	OQ189094	OQ261069	–	–	–	–
5. GuangDong-Pin.	*C. kyotensis*	*C. kyotensis*	ADAAAA	CSF23614	20210606-1-(229)	OQ189095	OQ261070	OQ261563	OQ302998	OQ303204	OQ303411
5. GuangDong-Pin.	*C. kyotensis*	*C. kyotensis*	AIDAAA	CSF23480	20210606-1-(79)	OQ189096	OQ261071	OQ261569	OQ303004	OQ303210	OQ303417
5. GuangDong-Pin.	*C. kyotensis*	*C. kyotensis*	AIDAAA	CSF23555	20210606-1-(164)	OQ189097	OQ261072	OQ261570	OQ303005	OQ303211	OQ303418
5. GuangDong-Pin.	*C. kyotensis*	*C. kyotensis*	AOAAAA	CSF23468	20210606-1-(60)	OQ189098	OQ261073	OQ261578	OQ303013	OQ303219	OQ303426
5. GuangDong-Pin.	*C. kyotensis*	*C. kyotensis*	AOAAAA	CSF23481	20210606-1-(80)	OQ189099	OQ261074	OQ261579	OQ303014	OQ303220	OQ303427
5. GuangDong-Pin.	*C. kyotensis*	*C. kyotensis*	AOAAAA	CSF23572	20210606-1-(182)	OQ189100	OQ261075	OQ261580	OQ303015	OQ303221	OQ303428
5. GuangDong-Pin.	*C. kyotensis*	*C. kyotensis*	AODAAA	CSF23455	20210606-1-(47)	OQ189101	OQ261076	OQ261581	OQ303016	OQ303222	OQ303429
5. GuangDong-Pin.	*C. kyotensis*	*C. kyotensis*	AODAAA	CSF23584	20210606-1-(196)	OQ189102	OQ261077	OQ261582	OQ303017	OQ303223	OQ303430
5. GuangDong-Pin.	*C. kyotensis*	*C. kyotensis*	APAAAA	CSF23505	20210606-1-(107)	OQ189103	OQ261078	OQ261583	OQ303018	OQ303224	OQ303431
5. GuangDong-Pin.	*C. kyotensis*	*C. kyotensis*	ARAAAA	CSF23437	20210606-1-(27)	OQ189104	OQ261079	OQ261585	OQ303020	OQ303226	OQ303433
5. GuangDong-Pin.	*C. kyotensis*	*C. kyotensis*	DA----	CSF23477	20210606-1-(74)	OQ189105	OQ261080	–	–	–	–
5. GuangDong-Pin.	*C. kyotensis*	*C. kyotensis*	DA----	CSF23504	20210606-1-(106)	OQ189106	OQ261081	–	–	–	–
5. GuangDong-Pin.	*C. kyotensis*	*C. kyotensis*	DCABAA	CSF23581	20210606-1-(193)	OQ189107	OQ261082	OQ261626	OQ303061	OQ303267	OQ303474
5. GuangDong-Pin.	*C. kyotensis*	*C. kyotensis*	DGDAAA	CSF23494	20210606-1-(93)	OQ189108	OQ261083	OQ261634	OQ303069	OQ303275	OQ303482
5. GuangDong-Pin.	*C. kyotensis*	*C. kyotensis*	DI----	CSF23509	20210606-1-(113)	OQ189109	OQ261084	–	–	–	–
5. GuangDong-Pin.	*C. kyotensis*	*C. kyotensis*	DLAAAA	CSF23507	20210606-1-(109)	OQ189110	OQ261085	OQ261643	OQ303078	OQ303284	OQ303491
5. GuangDong-Pin.	*C. kyotensis*	*C. kyotensis*	DO----	CSF23450	20210606-1-(41)	OQ189111	OQ261086	–	–	–	–
5. GuangDong-Pin.	*C. kyotensis*	*C. kyotensis*	DOAAAA	CSF23452	20210606-1-(43)	OQ189112	OQ261087	OQ261649	OQ303084	OQ303290	OQ303497
5. GuangDong-Pin.	*C. kyotensis*	*C. kyotensis*	DODBAA	CSF23582	20210606-1-(194)	OQ189113	OQ261088	OQ261650	OQ303085	OQ303291	OQ303498
5. GuangDong-Pin.	*C. kyotensis*	*C. kyotensis*	DRDAAA	CSF23475	20210606-1-(71)	OQ189114	OQ261089	OQ261652	OQ303087	OQ303293	OQ303500
5. GuangDong-Pin.	*C. kyotensis*	*C. kyotensis*	DRDAAA	CSF23534	20210606-1-(143)	OQ189115	OQ261090	OQ261653	OQ303088	OQ303294	OQ303501
5. GuangDong-Pin.	*C. kyotensis*	*C. kyotensis*	EADAAA	CSF23512	20210606-1-(116)	OQ189116	OQ261091	OQ261655	OQ303090	OQ303296	OQ303503
5. GuangDong-Pin.	*C. kyotensis*	*C. kyotensis*	ELAAAA	CSF23499	20210606-1-(98)	OQ189117	OQ261092	OQ261656	OQ303091	OQ303297	OQ303504
5. GuangDong-Pin.	*C. kyotensis*	*C. kyotensis*	EOAAAA	CSF23474	20210606-1-(70)	OQ189118	OQ261093	OQ261657	OQ303092	OQ303298	OQ303505
5. GuangDong-Pin.	*C. kyotensis*	*C. pacifica*	AAAAAA	CSF23543	20210606-1-(151)	OQ189119	OQ261094	OQ261658	OQ303093	OQ303299	OQ303506
5. GuangDong-Pin.	*C. kyotensis*	*C. pacifica*	BBBAAA	CSF23608	20210606-1-(223)	OQ189120	OQ261095	OQ261660	OQ303095	OQ303301	OQ303508
6. GuangDong-Cun.	*C. kyotensis*	*C. aconidialis*	AA----	CSF22543	20210609-1-(4)	OQ189121	OQ261096	–	–	–	–
6. GuangDong-Cun.	*C. kyotensis*	*C. aconidialis*	AA----	CSF22545	20210609-1-(13)	OQ189122	OQ261097	–	–	–	–
6. GuangDong-Cun.	*C. kyotensis*	*C. aconidialis*	AA----	CSF23397	20210609-1-(28)	OQ189123	OQ261098	–	–	–	–
6. GuangDong-Cun.	*C. kyotensis*	*C. aconidialis*	AA----	CSF23399	20210609-1-(35)	OQ189124	OQ261099	–	–	–	–
6. GuangDong-Cun.	*C. kyotensis*	*C. aconidialis*	AA----	CSF23400	20210609-1-(37)	OQ189125	OQ261100	–	–	–	–
6. GuangDong-Cun.	*C. kyotensis*	*C. aconidialis*	AA----	CSF23402	20210609-1-(58)	OQ189126	OQ261101	–	–	–	–
6. GuangDong-Cun.	*C. kyotensis*	*C. aconidialis*	AA----	CSF23403	20210609-1-(61)	OQ189127	OQ261102	–	–	–	–
6. GuangDong-Cun.	*C. kyotensis*	*C. aconidialis*	AA----	CSF23404	20210609-1-(67)	OQ189128	OQ261103	–	–	–	–
6. GuangDong-Cun.	*C. kyotensis*	*C. aconidialis*	AA----	CSF23405	20210609-1-(68)	OQ189129	OQ261104	–	–	–	–
6. GuangDong-Cun.	*C. kyotensis*	*C. aconidialis*	AA----	CSF23407	20210609-1-(74)	OQ189130	OQ261105	–	–	–	–
6. GuangDong-Cun.	*C. kyotensis*	*C. aconidialis*	AA----	CSF23410	20210609-1-(97)	OQ189131	OQ261106	–	–	–	–
6. GuangDong-Cun.	*C. kyotensis*	*C. aconidialis*	AA----	CSF23411	20210609-1-(103)	OQ189132	OQ261107	–	–	–	–
6. GuangDong-Cun.	*C. kyotensis*	*C. aconidialis*	AA----	CSF23412	20210609-1-(107)	OQ189133	OQ261108	–	–	–	–
6. GuangDong-Cun.	*C. kyotensis*	*C. aconidialis*	AA----	CSF23414	20210609-1-(115)	OQ189134	OQ261109	–	–	–	–
6. GuangDong-Cun.	*C. kyotensis*	*C. aconidialis*	AA----	CSF23415	20210609-1-(116)	OQ189135	OQ261110	–	–	–	–
6. GuangDong-Cun.	*C. kyotensis*	*C. aconidialis*	AA----	CSF23419	20210609-1-(150)	OQ189136	OQ261111	–	–	–	–
6. GuangDong-Cun.	*C. kyotensis*	*C. aconidialis*	AA----	CSF23420	20210609-1-(151)	OQ189137	OQ261112	–	–	–	–
6. GuangDong-Cun.	*C. kyotensis*	*C. aconidialis*	AA----	CSF23421	20210609-1-(155)	OQ189138	OQ261113	–	–	–	–
6. GuangDong-Cun.	*C. kyotensis*	*C. aconidialis*	AA----	CSF23422	20210609-1-(157)	OQ189139	OQ261114	–	–	–	–
6. GuangDong-Cun.	*C. kyotensis*	*C. aconidialis*	AA----	CSF23424	20210609-1-(166)	OQ189140	OQ261115	–	–	–	–
6. GuangDong-Cun.	*C. kyotensis*	*C. aconidialis*	AA----	CSF23425	20210609-1-(178)	OQ189141	OQ261116	–	–	–	–
6. GuangDong-Cun.	*C. kyotensis*	*C. aconidialis*	AA----	CSF23426	20210609-1-(181)	OQ189142	OQ261117	–	–	–	–
6. GuangDong-Cun.	*C. kyotensis*	*C. aconidialis*	AA----	CSF23427	20210609-1-(186)	OQ189143	OQ261118	–	–	–	–
6. GuangDong-Cun.	*C. kyotensis*	*C. aconidialis*	AA----	CSF23428	20210609-1-(212)	OQ189144	OQ261119	–	–	–	–
6. GuangDong-Cun.	*C. kyotensis*	*C. aconidialis*	AA----	CSF23430	20210609-1-(220)	OQ189145	OQ261120	–	–	–	–
6. GuangDong-Cun.	*C. kyotensis*	*C. aconidialis*	AA----	CSF23431	20210609-1-(231)	OQ189146	OQ261121	–	–	–	–
6. GuangDong-Cun.	*C. kyotensis*	*C. aconidialis*	AA----	CSF23432	20210609-1-(233)	OQ189147	OQ261122	–	–	–	–
6. GuangDong-Cun.	*C. kyotensis*	*C. aconidialis*	AA----	CSF23433	20210609-1-(235)	OQ189148	OQ261123	–	–	–	–
6. GuangDong-Cun.	*C. kyotensis*	*C. aconidialis*	AA----	CSF23435	20210609-1-(239)	OQ189149	OQ261124	–	–	–	–
6. GuangDong-Cun.	*C. kyotensis*	*C. aconidialis*	AA----	CSF23436	20210609-1-(240)	OQ189150	OQ261125	–	–	–	–
6. GuangDong-Cun.	*C. kyotensis*	*C. aconidialis*	AHAAAA	CSF23409	20210609-1-(92)	OQ189151	OQ261126	OQ261475	OQ302910	OQ303117	OQ303323
6. GuangDong-Cun.	*C. kyotensis*	*C. aconidialis*	AMCAAA	CSF22542	20210609-1-(3)	OQ189152	OQ261127	OQ261483	OQ302918	OQ303125	OQ303331
6. GuangDong-Cun.	*C. kyotensis*	*C. aconidialis*	AT----	CSF23398	20210609-1-(29)	OQ189153	OQ261128	–	–	–	–
6. GuangDong-Cun.	*C. kyotensis*	*C. aconidialis*	ATAAAA	CSF23429	20210609-1-(213)	OQ189154	OQ261129	OQ261493	OQ302928	OQ303135	OQ303341
6. GuangDong-Cun.	*C. kyotensis*	*C. aconidialis*	FAAAAA	CSF23401	20210609-1-(52)	OQ189155	OQ261130	OQ261509	OQ302944	OQ303151	OQ303357
6. GuangDong-Cun.	*C. kyotensis*	*C. ilicicola*	AA----	CSF23406	20210609-1-(71)	OQ189156	OQ261131	–	–	–	–
6. GuangDong-Cun.	*C. kyotensis*	*C. ilicicola*	AA----	CSF23416	20210609-1-(133)	OQ189157	OQ261132	–	–	–	–
6. GuangDong-Cun.	*C. kyotensis*	*C. ilicicola*	AA----	CSF23423	20210609-1-(164)	OQ189158	OQ261133	–	–	–	–
6. GuangDong-Cun.	*C. kyotensis*	*C. kyotensis*	CRAACA	CSF23408	20210609-1-(84)	OQ189159	OQ261134	OQ261615	OQ303050	OQ303256	OQ303463
6. GuangDong-Cun.	*C. kyotensis*	*C. kyotensis*	DA----	CSF23413	20210609-1-(108)	OQ189160	OQ261135	–	–	–	–
6. GuangDong-Cun.	*C. kyotensis*	*C. kyotensis*	DADAAA	CSF23418	20210609-1-(141)	OQ189161	OQ261136	OQ261625	OQ303060	OQ303266	OQ303473
6. GuangDong-Cun.	*C. kyotensis*	*C. kyotensis*	DDABAA	CSF22546	20210609-1-(17)	OQ189162	OQ261137	OQ261628	OQ303063	OQ303269	OQ303476
6. GuangDong-Cun.	*C. kyotensis*	*C. kyotensis*	DL----	CSF23434	20210609-1-(238)	OQ189163	OQ261138	–	–	–	–
6. GuangDong-Cun.	*C. kyotensis*	*C. pacifica*	ACAAAA	CSF22544	20210609-1-(11)	OQ189164	OQ261139	OQ261659	OQ303094	OQ303300	OQ303507
7. GuangXi-Euc.	*C. kyotensis*	*C. aconidialis*	AA----	CSF22570	20210621-1-(1)	OQ189165	OQ261140	–	–	–	–
7. GuangXi-Euc.	*C. kyotensis*	*C. aconidialis*	AA----	CSF22574	20210621-1-(3)	OQ189166	OQ261141	–	–	–	–
7. GuangXi-Euc.	*C. kyotensis*	*C. aconidialis*	AA----	CSF22582	20210621-1-(7)	OQ189167	OQ261142	–	–	–	–
7. GuangXi-Euc.	*C. kyotensis*	*C. aconidialis*	AA----	CSF22584	20210621-1-(8)	OQ189168	OQ261143	–	–	–	–
7. GuangXi-Euc.	*C. kyotensis*	*C. aconidialis*	AA----	CSF22590	20210621-1-(11)	OQ189169	OQ261144	–	–	–	–
7. GuangXi-Euc.	*C. kyotensis*	*C. aconidialis*	AA----	CSF22600	20210621-1-(16)	OQ189170	OQ261145	–	–	–	–
7. GuangXi-Euc.	*C. kyotensis*	*C. aconidialis*	AA----	CSF22602	20210621-1-(17)	OQ189171	OQ261146	–	–	–	–
7. GuangXi-Euc.	*C. kyotensis*	*C. aconidialis*	AA----	CSF22604	20210621-1-(18)	OQ189172	OQ261147	–	–	–	–
7. GuangXi-Euc.	*C. kyotensis*	*C. aconidialis*	AA----	CSF22608	20210621-1-(20)	OQ189173	OQ261148	–	–	–	–
7. GuangXi-Euc.	*C. kyotensis*	*C. aconidialis*	AA----	CSF22610	20210621-1-(21)	OQ189174	OQ261149	–	–	–	–
7. GuangXi-Euc.	*C. kyotensis*	*C. aconidialis*	AA----	CSF22612	20210621-1-(22)	OQ189175	OQ261150	–	–	–	–
7. GuangXi-Euc.	*C. kyotensis*	*C. aconidialis*	AA----	CSF22616	20210621-1-(25)	OQ189176	OQ261151	–	–	–	–
7. GuangXi-Euc.	*C. kyotensis*	*C. aconidialis*	AA----	CSF23635	20210621-1-(27)	OQ189177	OQ261152	–	–	–	–
7. GuangXi-Euc.	*C. kyotensis*	*C. aconidialis*	AA----	CSF23636	20210621-1-(29)	OQ189178	OQ261153	–	–	–	–
7. GuangXi-Euc.	*C. kyotensis*	*C. aconidialis*	AA----	CSF23637	20210621-1-(30)	OQ189179	OQ261154	–	–	–	–
7. GuangXi-Euc.	*C. kyotensis*	*C. aconidialis*	AA----	CSF23638	20210621-1-(31)	OQ189180	OQ261155	–	–	–	–
7. GuangXi-Euc.	*C. kyotensis*	*C. aconidialis*	AA----	CSF23639	20210621-1-(32)	OQ189181	OQ261156	–	–	–	–
7. GuangXi-Euc.	*C. kyotensis*	*C. aconidialis*	AA----	CSF23640	20210621-1-(33)	OQ189182	OQ261157	–	–	–	–
7. GuangXi-Euc.	*C. kyotensis*	*C. aconidialis*	AA----	CSF23641	20210621-1-(34)	OQ189183	OQ261158	–	–	–	–
7. GuangXi-Euc.	*C. kyotensis*	*C. aconidialis*	AA----	CSF23642	20210621-1-(35)	OQ189184	OQ261159	–	–	–	–
7. GuangXi-Euc.	*C. kyotensis*	*C. aconidialis*	AA----	CSF23647	20210621-1-(40)	OQ189185	OQ261160	–	–	–	–
7. GuangXi-Euc.	*C. kyotensis*	*C. aconidialis*	AA----	CSF23648	20210621-1-(42)	OQ189186	OQ261161	–	–	–	–
7. GuangXi-Euc.	*C. kyotensis*	*C. aconidialis*	AA----	CSF23649	20210621-1-(43)	OQ189187	OQ261162	–	–	–	–
7. GuangXi-Euc.	*C. kyotensis*	*C. aconidialis*	AA----	CSF23650	20210621-1-(44)	OQ189188	OQ261163	–	–	–	–
7. GuangXi-Euc.	*C. kyotensis*	*C. aconidialis*	AA----	CSF23652	20210621-1-(46)	OQ189189	OQ261164	–	–	–	–
7. GuangXi-Euc.	*C. kyotensis*	*C. aconidialis*	AA----	CSF23653	20210621-1-(48)	OQ189190	OQ261165	–	–	–	–
7. GuangXi-Euc.	*C. kyotensis*	*C. aconidialis*	AA----	CSF23654	20210621-1-(49)	OQ189191	OQ261166	–	–	–	–
7. GuangXi-Euc.	*C. kyotensis*	*C. aconidialis*	AA----	CSF23655	20210621-1-(50)	OQ189192	OQ261167	–	–	–	–
7. GuangXi-Euc.	*C. kyotensis*	*C. aconidialis*	AA----	CSF23656	20210621-1-(51)	OQ189193	OQ261168	–	–	–	–
7. GuangXi-Euc.	*C. kyotensis*	*C. aconidialis*	AA----	CSF23657	20210621-1-(52)	OQ189194	OQ261169	–	–	–	–
7. GuangXi-Euc.	*C. kyotensis*	*C. aconidialis*	AA----	CSF23658	20210621-1-(53)	OQ189195	OQ261170	–	–	–	–
7. GuangXi-Euc.	*C. kyotensis*	*C. aconidialis*	AA----	CSF23659	20210624-1-(2)	OQ189196	OQ261171	–	–	–	–
7. GuangXi-Euc.	*C. kyotensis*	*C. aconidialis*	AA----	CSF23661	20210624-1-(4)	OQ189197	OQ261172	–	–	–	–
7. GuangXi-Euc.	*C. kyotensis*	*C. aconidialis*	AA----	CSF23662	20210624-1-(5)	OQ189198	OQ261173	–	–	–	–
7. GuangXi-Euc.	*C. kyotensis*	*C. aconidialis*	AA----	CSF23663	20210624-1-(9)	OQ189199	OQ261174	–	–	–	–
7. GuangXi-Euc.	*C. kyotensis*	*C. aconidialis*	AA----	CSF23664	20210624-1-(11)	OQ189200	OQ261175	–	–	–	–
7. GuangXi-Euc.	*C. kyotensis*	*C. aconidialis*	AA----	CSF23665	20210624-1-(12)	OQ189201	OQ261176	–	–	–	–
7. GuangXi-Euc.	*C. kyotensis*	*C. aconidialis*	AA----	CSF23666	20210624-1-(16)	OQ189202	OQ261177	–	–	–	–
7. GuangXi-Euc.	*C. kyotensis*	*C. aconidialis*	AA----	CSF23667	20210624-1-(17)	OQ189203	OQ261178	–	–	–	–
7. GuangXi-Euc.	*C. kyotensis*	*C. aconidialis*	AA----	CSF23668	20210624-1-(18)	OQ189204	OQ261179	–	–	–	–
7. GuangXi-Euc.	*C. kyotensis*	*C. aconidialis*	AA----	CSF23670	20210624-1-(23)	OQ189205	OQ261180	–	–	–	–
7. GuangXi-Euc.	*C. kyotensis*	*C. aconidialis*	AA----	CSF23672	20210624-1-(26)	OQ189206	OQ261181	–	–	–	–
7. GuangXi-Euc.	*C. kyotensis*	*C. aconidialis*	AA----	CSF23673	20210624-1-(27)	OQ189207	OQ261182	–	–	–	–
7. GuangXi-Euc.	*C. kyotensis*	*C. aconidialis*	AA----	CSF23674	20210624-1-(29)	OQ189208	OQ261183	–	–	–	–
7. GuangXi-Euc.	*C. kyotensis*	*C. aconidialis*	AA----	CSF23676	20210624-1-(31)	OQ189209	OQ261184	–	–	–	–
7. GuangXi-Euc.	*C. kyotensis*	*C. aconidialis*	AA----	CSF23677	20210624-1-(32)	OQ189210	OQ261185	–	–	–	–
7. GuangXi-Euc.	*C. kyotensis*	*C. aconidialis*	AA----	CSF23678	20210624-1-(33)	OQ189211	OQ261186	–	–	–	–
7. GuangXi-Euc.	*C. kyotensis*	*C. aconidialis*	AA----	CSF23680	20210624-1-(35)	OQ189212	OQ261187	–	–	–	–
7. GuangXi-Euc.	*C. kyotensis*	*C. aconidialis*	AA----	CSF23681	20210624-1-(36)	OQ189213	OQ261188	–	–	–	–
7. GuangXi-Euc.	*C. kyotensis*	*C. aconidialis*	AA----	CSF23682	20210624-1-(37)	OQ189214	OQ261189	–	–	–	–
7. GuangXi-Euc.	*C. kyotensis*	*C. aconidialis*	AA----	CSF23683	20210624-1-(38)	OQ189215	OQ261190	–	–	–	–
7. GuangXi-Euc.	*C. kyotensis*	*C. aconidialis*	AA----	CSF23684	20210624-1-(39)	OQ189216	OQ261191	–	–	–	–
7. GuangXi-Euc.	*C. kyotensis*	*C. aconidialis*	AA----	CSF23685	20210624-1-(40)	OQ189217	OQ261192	–	–	–	–
7. GuangXi-Euc.	*C. kyotensis*	*C. aconidialis*	AA----	CSF23686	20210624-1-(42)	OQ189218	OQ261193	–	–	–	–
7. GuangXi-Euc.	*C. kyotensis*	*C. aconidialis*	AA----	CSF23687	20210624-1-(43)	OQ189219	OQ261194	–	–	–	–
7. GuangXi-Euc.	*C. kyotensis*	*C. aconidialis*	AA----	CSF23689	20210624-1-(45)	OQ189220	OQ261195	–	–	–	–
7. GuangXi-Euc.	*C. kyotensis*	*C. aconidialis*	AA----	CSF23690	20210624-1-(46)	OQ189221	OQ261196	–	–	–	–
7. GuangXi-Euc.	*C. kyotensis*	*C. aconidialis*	AA----	CSF23691	20210624-1-(47)	OQ189222	OQ261197	–	–	–	–
7. GuangXi-Euc.	*C. kyotensis*	*C. aconidialis*	AA----	CSF23692	20210624-1-(48)	OQ189223	OQ261198	–	–	–	–
7. GuangXi-Euc.	*C. kyotensis*	*C. aconidialis*	AA----	CSF23693	20210624-1-(49)	OQ189224	OQ261199	–	–	–	–
7. GuangXi-Euc.	*C. kyotensis*	*C. aconidialis*	AA----	CSF23694	20210624-1-(50)	OQ189225	OQ261200	–	–	–	–
7. GuangXi-Euc.	*C. kyotensis*	*C. aconidialis*	AA----	CSF23695	20210624-1-(51)	OQ189226	OQ261201	–	–	–	–
7. GuangXi-Euc.	*C. kyotensis*	*C. aconidialis*	AA----	CSF23698	20210624-1-(54)	OQ189227	OQ261202	–	–	–	–
7. GuangXi-Euc.	*C. kyotensis*	*C. aconidialis*	AA----	CSF23699	20210624-1-(55)	OQ189228	OQ261203	–	–	–	–
7. GuangXi-Euc.	*C. kyotensis*	*C. aconidialis*	AA----	CSF23701	20210624-1-(58)	OQ189229	OQ261204	–	–	–	–
7. GuangXi-Euc.	*C. kyotensis*	*C. aconidialis*	AA----	CSF23702	20210624-1-(60)	OQ189230	OQ261205	–	–	–	–
7. GuangXi-Euc.	*C. kyotensis*	*C. aconidialis*	AA----	CSF23704	20210624-1-(65)	OQ189231	OQ261206	–	–	–	–
7. GuangXi-Euc.	*C. kyotensis*	*C. aconidialis*	AA----	CSF23705	20210624-1-(66)	OQ189232	OQ261207	–	–	–	–
7. GuangXi-Euc.	*C. kyotensis*	*C. aconidialis*	AA----	CSF23706	20210624-1-(67)	OQ189233	OQ261208	–	–	–	–
7. GuangXi-Euc.	*C. kyotensis*	*C. aconidialis*	AA----	CSF23709	20210624-1-(74)	OQ189234	OQ261209	–	–	–	–
7. GuangXi-Euc.	*C. kyotensis*	*C. aconidialis*	AA----	CSF23710	20210624-1-(75)	OQ189235	OQ261210	–	–	–	–
7. GuangXi-Euc.	*C. kyotensis*	*C. aconidialis*	AA----	CSF23712	20210624-1-(77)	OQ189236	OQ261211	–	–	–	–
7. GuangXi-Euc.	*C. kyotensis*	*C. aconidialis*	AA----	CSF23713	20210624-1-(78)	OQ189237	OQ261212	–	–	–	–
7. GuangXi-Euc.	*C. kyotensis*	*C. aconidialis*	AA----	CSF23714	20210624-1-(79)	OQ189238	OQ261213	–	–	–	–
7. GuangXi-Euc.	*C. kyotensis*	*C. aconidialis*	AA----	CSF23717	20210624-1-(82)	OQ189239	OQ261214	–	–	–	–
7. GuangXi-Euc.	*C. kyotensis*	*C. aconidialis*	AA----	CSF23719	20210624-1-(84)	OQ189240	OQ261215	–	–	–	–
7. GuangXi-Euc.	*C. kyotensis*	*C. aconidialis*	AA----	CSF23720	20210624-1-(87)	OQ189241	OQ261216	–	–	–	–
7. GuangXi-Euc.	*C. kyotensis*	*C. aconidialis*	AA----	CSF23721	20210624-1-(88)	OQ189242	OQ261217	–	–	–	–
7. GuangXi-Euc.	*C. kyotensis*	*C. aconidialis*	AA----	CSF23723	20210624-1-(90)	OQ189243	OQ261218	–	–	–	–
7. GuangXi-Euc.	*C. kyotensis*	*C. aconidialis*	AA----	CSF23724	20210624-1-(92)	OQ189244	OQ261219	–	–	–	–
7. GuangXi-Euc.	*C. kyotensis*	*C. aconidialis*	AA----	CSF23725	20210624-1-(94)	OQ189245	OQ261220	–	–	–	–
7. GuangXi-Euc.	*C. kyotensis*	*C. aconidialis*	AA----	CSF23726	20210624-1-(96)	OQ189246	OQ261221	–	–	–	–
7. GuangXi-Euc.	*C. kyotensis*	*C. aconidialis*	AA----	CSF23727	20210624-1-(97)	OQ189247	OQ261222	–	–	–	–
7. GuangXi-Euc.	*C. kyotensis*	*C. aconidialis*	AA----	CSF23728	20210624-1-(100)	OQ189248	OQ261223	–	–	–	–
7. GuangXi-Euc.	*C. kyotensis*	*C. aconidialis*	AA----	CSF23729	20210624-1-(106)	OQ189249	OQ261224	–	–	–	–
7. GuangXi-Euc.	*C. kyotensis*	*C. aconidialis*	AA----	CSF23730	20210624-1-(107)	OQ189250	OQ261225	–	–	–	–
7. GuangXi-Euc.	*C. kyotensis*	*C. aconidialis*	AA----	CSF23731	20210624-1-(108)	OQ189251	OQ261226	–	–	–	–
7. GuangXi-Euc.	*C. kyotensis*	*C. aconidialis*	AA----	CSF23732	20210624-1-(113)	OQ189252	OQ261227	–	–	–	–
7. GuangXi-Euc.	*C. kyotensis*	*C. aconidialis*	AA----	CSF23733	20210624-1-(114)	OQ189253	OQ261228	–	–	–	–
7. GuangXi-Euc.	*C. kyotensis*	*C. aconidialis*	AA----	CSF23734	20210624-1-(115)	OQ189254	OQ261229	–	–	–	–
7. GuangXi-Euc.	*C. kyotensis*	*C. aconidialis*	AA----	CSF23735	20210624-1-(116)	OQ189255	OQ261230	–	–	–	–
7. GuangXi-Euc.	*C. kyotensis*	*C. aconidialis*	AA----	CSF23737	20210624-1-(118)	OQ189256	OQ261231	–	–	–	–
7. GuangXi-Euc.	*C. kyotensis*	*C. aconidialis*	AA----	CSF23742	20210624-1-(133)	OQ189257	OQ261232	–	–	–	–
7. GuangXi-Euc.	*C. kyotensis*	*C. aconidialis*	AA----	CSF23743	20210624-1-(138)	OQ189258	OQ261233	–	–	–	–
7. GuangXi-Euc.	*C. kyotensis*	*C. aconidialis*	AA----	CSF23745	20210624-1-(140)	OQ189259	OQ261234	–	–	–	–
7. GuangXi-Euc.	*C. kyotensis*	*C. aconidialis*	AA----	CSF23746	20210624-1-(142)	OQ189260	OQ261235	–	–	–	–
7. GuangXi-Euc.	*C. kyotensis*	*C. aconidialis*	AA----	CSF23749	20210624-1-(146)	OQ189261	OQ261236	–	–	–	–
7. GuangXi-Euc.	*C. kyotensis*	*C. aconidialis*	AA----	CSF23750	20210624-1-(148)	OQ189262	OQ261237	–	–	–	–
7. GuangXi-Euc.	*C. kyotensis*	*C. aconidialis*	AA----	CSF23751	20210624-1-(151)	OQ189263	OQ261238	–	–	–	–
7. GuangXi-Euc.	*C. kyotensis*	*C. aconidialis*	AA----	CSF23752	20210624-1-(152)	OQ189264	OQ261239	–	–	–	–
7. GuangXi-Euc.	*C. kyotensis*	*C. aconidialis*	AA----	CSF23753	20210624-1-(153)	OQ189265	OQ261240	–	–	–	–
7. GuangXi-Euc.	*C. kyotensis*	*C. aconidialis*	AA----	CSF23755	20210624-1-(155)	OQ189266	OQ261241	–	–	–	–
7. GuangXi-Euc.	*C. kyotensis*	*C. aconidialis*	AA----	CSF23756	20210624-1-(156)	OQ189267	OQ261242	–	–	–	–
7. GuangXi-Euc.	*C. kyotensis*	*C. aconidialis*	AA----	CSF23757	20210624-1-(157)	OQ189268	OQ261243	–	–	–	–
7. GuangXi-Euc.	*C. kyotensis*	*C. aconidialis*	AA----	CSF23759	20210624-1-(160)	OQ189269	OQ261244	–	–	–	–
7. GuangXi-Euc.	*C. kyotensis*	*C. aconidialis*	AA----	CSF23760	20210624-1-(161)	OQ189270	OQ261245	–	–	–	–
7. GuangXi-Euc.	*C. kyotensis*	*C. aconidialis*	AA----	CSF23762	20210624-1-(163)	OQ189271	OQ261246	–	–	–	–
7. GuangXi-Euc.	*C. kyotensis*	*C. aconidialis*	AA----	CSF23764	20210624-1-(165)	OQ189272	OQ261247	–	–	–	–
7. GuangXi-Euc.	*C. kyotensis*	*C. aconidialis*	AA----	CSF23766	20210624-1-(167)	OQ189273	OQ261248	–	–	–	–
7. GuangXi-Euc.	*C. kyotensis*	*C. aconidialis*	AA----	CSF23767	20210624-1-(168)	OQ189274	OQ261249	–	–	–	–
7. GuangXi-Euc.	*C. kyotensis*	*C. aconidialis*	AA----	CSF23768	20210624-1-(169)	OQ189275	OQ261250	–	–	–	–
7. GuangXi-Euc.	*C. kyotensis*	*C. aconidialis*	AA----	CSF23770	20210624-1-(171)	OQ189276	OQ261251	–	–	–	–
7. GuangXi-Euc.	*C. kyotensis*	*C. aconidialis*	AA----	CSF23772	20210624-2-(2)	OQ189277	OQ261252	–	–	–	–
7. GuangXi-Euc.	*C. kyotensis*	*C. aconidialis*	AA----	CSF23774	20210624-2-(4)	OQ189278	OQ261253	–	–	–	–
7. GuangXi-Euc.	*C. kyotensis*	*C. aconidialis*	AA----	CSF23775	20210624-2-(5)	OQ189279	OQ261254	–	–	–	–
7. GuangXi-Euc.	*C. kyotensis*	*C. aconidialis*	AA----	CSF23776	20210624-2-(6)	OQ189280	OQ261255	–	–	–	–
7. GuangXi-Euc.	*C. kyotensis*	*C. aconidialis*	AA----	CSF23777	20210624-2-(7)	OQ189281	OQ261256	–	–	–	–
7. GuangXi-Euc.	*C. kyotensis*	*C. aconidialis*	AA----	CSF23778	20210624-2-(8)	OQ189282	OQ261257	–	–	–	–
7. GuangXi-Euc.	*C. kyotensis*	*C. aconidialis*	AA----	CSF23780	20210624-2-(10)	OQ189283	OQ261258	–	–	–	–
7. GuangXi-Euc.	*C. kyotensis*	*C. aconidialis*	AA----	CSF23781	20210624-2-(12)	OQ189284	OQ261259	–	–	–	–
7. GuangXi-Euc.	*C. kyotensis*	*C. aconidialis*	AA----	CSF23785	20210624-2-(16)	OQ189285	OQ261260	–	–	–	–
7. GuangXi-Euc.	*C. kyotensis*	*C. aconidialis*	AA----	CSF23786	20210624-2-(17)	OQ189286	OQ261261	–	–	–	–
7. GuangXi-Euc.	*C. kyotensis*	*C. aconidialis*	AA----	CSF23787	20210624-2-(18)	OQ189287	OQ261262	–	–	–	–
7. GuangXi-Euc.	*C. kyotensis*	*C. aconidialis*	AA----	CSF23788	20210624-2-(19)	OQ189288	OQ261263	–	–	–	–
7. GuangXi-Euc.	*C. kyotensis*	*C. aconidialis*	AA----	CSF23789	20210624-2-(20)	OQ189289	OQ261264	–	–	–	–
7. GuangXi-Euc.	*C. kyotensis*	*C. aconidialis*	AA----	CSF23790	20210624-2-(21)	OQ189290	OQ261265	–	–	–	–
7. GuangXi-Euc.	*C. kyotensis*	*C. aconidialis*	AA----	CSF23791	20210624-2-(22)	OQ189291	OQ261266	–	–	–	–
7. GuangXi-Euc.	*C. kyotensis*	*C. aconidialis*	AA----	CSF23792	20210624-2-(23)	OQ189292	OQ261267	–	–	–	–
7. GuangXi-Euc.	*C. kyotensis*	*C. aconidialis*	AA----	CSF23793	20210624-2-(24)	OQ189293	OQ261268	–	–	–	–
7. GuangXi-Euc.	*C. kyotensis*	*C. aconidialis*	AA----	CSF23795	20210624-2-(26)	OQ189294	OQ261269	–	–	–	–
7. GuangXi-Euc.	*C. kyotensis*	*C. aconidialis*	AC----	CSF23748	20210624-1-(145)	OQ189295	OQ261270	–	–	–	–
7. GuangXi-Euc.	*C. kyotensis*	*C. aconidialis*	ACAAAA	CSF23671	20210624-1-(25)	OQ189296	OQ261271	OQ261466	OQ302901	OQ303108	OQ303314
7. GuangXi-Euc.	*C. kyotensis*	*C. aconidialis*	ACAAAA	CSF23740	20210624-1-(128)	OQ189297	OQ261272	OQ261467	OQ302902	OQ303109	OQ303315
7. GuangXi-Euc.	*C. kyotensis*	*C. aconidialis*	AL----	CSF22572	20210621-1-(2)	OQ189298	OQ261273	–	–	–	–
7. GuangXi-Euc.	*C. kyotensis*	*C. aconidialis*	AL----	CSF22576	20210621-1-(4)	OQ189299	OQ261274	–	–	–	–
7. GuangXi-Euc.	*C. kyotensis*	*C. aconidialis*	AL----	CSF22580	20210621-1-(6)	OQ189300	OQ261275	–	–	–	–
7. GuangXi-Euc.	*C. kyotensis*	*C. aconidialis*	AL----	CSF22589	20210621-1-(10)	OQ189301	OQ261276	–	–	–	–
7. GuangXi-Euc.	*C. kyotensis*	*C. aconidialis*	AL----	CSF22592	20210621-1-(12)	OQ189302	OQ261277	–	–	–	–
7. GuangXi-Euc.	*C. kyotensis*	*C. aconidialis*	AL----	CSF22614	20210621-1-(24)	OQ189303	OQ261278	–	–	–	–
7. GuangXi-Euc.	*C. kyotensis*	*C. aconidialis*	AL----	CSF23643	20210621-1-(36)	OQ189304	OQ261279	–	–	–	–
7. GuangXi-Euc.	*C. kyotensis*	*C. aconidialis*	AL----	CSF23646	20210621-1-(39)	OQ189305	OQ261280	–	–	–	–
7. GuangXi-Euc.	*C. kyotensis*	*C. aconidialis*	ALCAAA	CSF22578	20210621-1-(5)	OQ189306	OQ261281	OQ261481	OQ302916	OQ303123	OQ303329
7. GuangXi-Euc.	*C. kyotensis*	*C. aconidialis*	ALCAAA	CSF23747	20210624-1-(144)	OQ189307	OQ261282	OQ261482	OQ302917	OQ303124	OQ303330
7. GuangXi-Euc.	*C. kyotensis*	*C. aconidialis*	AMCDAA	CSF22599	20210621-1-(15)	OQ189308	OQ261283	OQ261484	OQ302919	OQ303126	OQ303332
7. GuangXi-Euc.	*C. kyotensis*	*C. aconidialis*	AT----	CSF23651	20210621-1-(45)	OQ189309	OQ261284	–	–	–	–
7. GuangXi-Euc.	*C. kyotensis*	*C. aconidialis*	AT----	CSF23736	20210624-1-(117)	OQ189310	OQ261285	–	–	–	–
7. GuangXi-Euc.	*C. kyotensis*	*C. aconidialis*	AT----	CSF23771	20210624-2-(1)	OQ189311	OQ261286	–	–	–	–
7. GuangXi-Euc.	*C. kyotensis*	*C. aconidialis*	AT----	CSF23773	20210624-2-(3)	OQ189312	OQ261287	–	–	–	–
7. GuangXi-Euc.	*C. kyotensis*	*C. aconidialis*	AT----	CSF23783	20210624-2-(14)	OQ189313	OQ261288	–	–	–	–
7. GuangXi-Euc.	*C. kyotensis*	*C. aconidialis*	ATAAAA	CSF22596	20210621-1-(14)	OQ189314	OQ261289	OQ261494	OQ302929	OQ303136	OQ303342
7. GuangXi-Euc.	*C. kyotensis*	*C. aconidialis*	BAAAAA	CSF23761	20210624-1-(162)	OQ189315	OQ261290	OQ261497	OQ302932	OQ303139	OQ303345
7. GuangXi-Euc.	*C. kyotensis*	*C. aconidialis*	EA----	CSF23794	20210624-2-(25)	OQ189316	OQ261291	–	–	–	–
7. GuangXi-Euc.	*C. kyotensis*	*C. aconidialis*	EAAAAA	CSF23741	20210624-1-(132)	OQ189317	OQ261292	OQ261507	OQ302942	OQ303149	OQ303355
7. GuangXi-Euc.	*C. kyotensis*	*C. aconidialis*	EAAAAA	CSF23779	20210624-2-(9)	OQ189318	OQ261293	OQ261508	OQ302943	OQ303150	OQ303356
7. GuangXi-Euc.	*C. kyotensis*	*C. hongkongensis*	AFAAAA	CSF23718	20210624-1-(83)	OQ189319	OQ261294	OQ261538	OQ302973	OQ303180	OQ303386
7. GuangXi-Euc.	*C. kyotensis*	*C. hongkongensis*	BFBAAA	CSF23782	20210624-2-(13)	OQ189320	OQ261295	OQ261550	OQ302985	OQ303192	OQ303398
7. GuangXi-Euc.	*C. kyotensis*	*C. ilicicola*	AA----	CSF22606	20210621-1-(19)	OQ189321	OQ261296	–	–	–	–
7. GuangXi-Euc.	*C. kyotensis*	*C. ilicicola*	AA----	CSF23700	20210624-1-(56)	OQ189322	OQ261297	–	–	–	–
7. GuangXi-Euc.	*C. kyotensis*	*C. ilicicola*	AA----	CSF23739	20210624-1-(122)	OQ189323	OQ261298	–	–	–	–
7. GuangXi-Euc.	*C. kyotensis*	*C. kyotensis*	CAABAA	CSF22586	20210621-1-(9)	OQ189324	OQ261299	OQ261587	OQ303022	OQ303228	OQ303435
7. GuangXi-Euc.	*C. kyotensis*	*C. kyotensis*	CADAAA	CSF23738	20210624-1-(121)	OQ189325	OQ261300	OQ261588	OQ303023	OQ303229	OQ303436
7. GuangXi-Euc.	*C. kyotensis*	*C. kyotensis*	CADAAA	CSF23784	20210624-2-(15)	OQ189326	OQ261301	OQ261589	OQ303024	OQ303230	OQ303437
7. GuangXi-Euc.	*C. kyotensis*	*C. kyotensis*	CADBAA	CSF23716	20210624-1-(81)	OQ189327	OQ261302	OQ261590	OQ303025	OQ303231	OQ303438
7. GuangXi-Euc.	*C. kyotensis*	*C. kyotensis*	CADDAA	CSF23644	20210621-1-(37)	OQ189328	OQ261303	OQ261591	OQ303026	OQ303232	OQ303439
7. GuangXi-Euc.	*C. kyotensis*	*C. kyotensis*	CEADAA	CSF23660	20210624-1-(3)	OQ189329	OQ261304	OQ261594	OQ303029	OQ303235	OQ303442
7. GuangXi-Euc.	*C. kyotensis*	*C. kyotensis*	CEDDAA	CSF23711	20210624-1-(76)	OQ189330	OQ261305	OQ261595	OQ303030	OQ303236	OQ303443
7. GuangXi-Euc.	*C. kyotensis*	*C. kyotensis*	CHDBAA	CSF23697	20210624-1-(53)	OQ189331	OQ261306	OQ261598	OQ303033	OQ303239	OQ303446
7. GuangXi-Euc.	*C. kyotensis*	*C. kyotensis*	CMDBAA	CSF23765	20210624-1-(166)	OQ189332	OQ261307	OQ261601	OQ303036	OQ303242	OQ303449
7. GuangXi-Euc.	*C. kyotensis*	*C. kyotensis*	CMDBAA	CSF23769	20210624-1-(170)	OQ189333	OQ261308	OQ261602	OQ303037	OQ303243	OQ303450
7. GuangXi-Euc.	*C. kyotensis*	*C. kyotensis*	CMDDAA	CSF23715	20210624-1-(80)	OQ189334	OQ261309	OQ261603	OQ303038	OQ303244	OQ303451
7. GuangXi-Euc.	*C. kyotensis*	*C. kyotensis*	CNDBAA	CSF22594	20210621-1-(13)	OQ189335	OQ261310	OQ261604	OQ303039	OQ303245	OQ303452
7. GuangXi-Euc.	*C. kyotensis*	*C. kyotensis*	COAAAA	CSF23708	20210624-1-(70)	OQ189336	OQ261311	OQ261606	OQ303041	OQ303247	OQ303454
7. GuangXi-Euc.	*C. kyotensis*	*C. kyotensis*	COABAA	CSF23675	20210624-1-(30)	OQ189337	OQ261312	OQ261607	OQ303042	OQ303248	OQ303455
7. GuangXi-Euc.	*C. kyotensis*	*C. kyotensis*	COABAA	CSF23754	20210624-1-(154)	OQ189338	OQ261313	OQ261608	OQ303043	OQ303249	OQ303456
7. GuangXi-Euc.	*C. kyotensis*	*C. kyotensis*	COABAA	CSF23758	20210624-1-(158)	OQ189339	OQ261314	OQ261609	OQ303044	OQ303250	OQ303457
7. GuangXi-Euc.	*C. kyotensis*	*C. kyotensis*	COABAA	CSF23763	20210624-1-(164)	OQ189340	OQ261315	OQ261610	OQ303045	OQ303251	OQ303458
7. GuangXi-Euc.	*C. kyotensis*	*C. kyotensis*	CODDAA	CSF23703	20210624-1-(64)	OQ189341	OQ261316	OQ261613	OQ303048	OQ303254	OQ303461
7. GuangXi-Euc.	*C. kyotensis*	*C. kyotensis*	CRABDA	CSF23696	20210624-1-(52)	OQ189342	OQ261317	OQ261616	OQ303051	OQ303257	OQ303464
7. GuangXi-Euc.	*C. kyotensis*	*C. kyotensis*	CRABDA	CSF23707	20210624-1-(69)	OQ189343	OQ261318	OQ261617	OQ303052	OQ303258	OQ303465
7. GuangXi-Euc.	*C. kyotensis*	*C. kyotensis*	CRABDA	CSF23722	20210624-1-(89)	OQ189344	OQ261319	OQ261618	OQ303053	OQ303259	OQ303466
7. GuangXi-Euc.	*C. kyotensis*	*C. kyotensis*	CUDAAA	CSF23744	20210624-1-(139)	OQ189345	OQ261320	OQ261622	OQ303057	OQ303263	OQ303470
7. GuangXi-Euc.	*C. kyotensis*	*C. kyotensis*	DEAAAA	CSF23645	20210621-1-(38)	OQ189346	OQ261321	OQ261631	OQ303066	OQ303272	OQ303479
8. GuangXi-Pin.	*C. kyotensis*	*C. aconidialis*	AA----	CSF22653	20210621-3-(46)	OQ189347	OQ261322	–	–	–	–
8. GuangXi-Pin.	*C. kyotensis*	*C. aconidialis*	AA----	CSF22654	20210622-1-(1)	OQ189348	OQ261323	–	–	–	–
8. GuangXi-Pin.	*C. kyotensis*	*C. aconidialis*	AA----	CSF22655	20210622-1-(8)	OQ189349	OQ261324	–	–	–	–
8. GuangXi-Pin.	*C. kyotensis*	*C. aconidialis*	AA----	CSF22656	20210622-1-(11)	OQ189350	OQ261325	–	–	–	–
8. GuangXi-Pin.	*C. kyotensis*	*C. aconidialis*	AA----	CSF22657	20210622-1-(12)	OQ189351	OQ261326	–	–	–	–
8. GuangXi-Pin.	*C. kyotensis*	*C. aconidialis*	AA----	CSF22658	20210622-1-(15)	OQ189352	OQ261327	–	–	–	–
8. GuangXi-Pin.	*C. kyotensis*	*C. aconidialis*	AA----	CSF22659	20210622-1-(16)	OQ189353	OQ261328	–	–	–	–
8. GuangXi-Pin.	*C. kyotensis*	*C. aconidialis*	AA----	CSF22660	20210622-1-(17)	OQ189354	OQ261329	–	–	–	–
8. GuangXi-Pin.	*C. kyotensis*	*C. aconidialis*	AA----	CSF22661	20210622-1-(20)	OQ189355	OQ261330	–	–	–	–
8. GuangXi-Pin.	*C. kyotensis*	*C. aconidialis*	AA----	CSF22664	20210622-1-(22)	OQ189356	OQ261331	–	–	–	–
8. GuangXi-Pin.	*C. kyotensis*	*C. aconidialis*	AA----	CSF22666	20210622-1-(23)	OQ189357	OQ261332	–	–	–	–
8. GuangXi-Pin.	*C. kyotensis*	*C. aconidialis*	AA----	CSF22668	20210622-1-(25)	OQ189358	OQ261333	–	–	–	–
8. GuangXi-Pin.	*C. kyotensis*	*C. aconidialis*	AA----	CSF22670	20210622-1-(35)	OQ189359	OQ261334	–	–	–	–
8. GuangXi-Pin.	*C. kyotensis*	*C. aconidialis*	AA----	CSF22671	20210622-1-(43)	OQ189360	OQ261335	–	–	–	–
8. GuangXi-Pin.	*C. kyotensis*	*C. aconidialis*	AA----	CSF22672	20210622-1-(44)	OQ189361	OQ261336	–	–	–	–
8. GuangXi-Pin.	*C. kyotensis*	*C. aconidialis*	AA----	CSF22673	20210622-1-(45)	OQ189362	OQ261337	–	–	–	–
8. GuangXi-Pin.	*C. kyotensis*	*C. aconidialis*	AA----	CSF22674	20210622-1-(47)	OQ189363	OQ261338	–	–	–	–
8. GuangXi-Pin.	*C. kyotensis*	*C. aconidialis*	AA----	CSF22675	20210622-1-(49)	OQ189364	OQ261339	–	–	–	–
8. GuangXi-Pin.	*C. kyotensis*	*C. aconidialis*	AA----	CSF22676	20210622-1-(50)	OQ189365	OQ261340	–	–	–	–
8. GuangXi-Pin.	*C. kyotensis*	*C. aconidialis*	AA----	CSF22677	20210622-1-(51)	OQ189366	OQ261341	–	–	–	–
8. GuangXi-Pin.	*C. kyotensis*	*C. aconidialis*	AA----	CSF22678	20210622-1-(52)	OQ189367	OQ261342	–	–	–	–
8. GuangXi-Pin.	*C. kyotensis*	*C. aconidialis*	AA----	CSF22682	20210622-1-(53)	OQ189368	OQ261343	–	–	–	–
8. GuangXi-Pin.	*C. kyotensis*	*C. aconidialis*	AA----	CSF22684	20210622-1-(57)	OQ189369	OQ261344	–	–	–	–
8. GuangXi-Pin.	*C. kyotensis*	*C. aconidialis*	AA----	CSF22685	20210622-1-(59)	OQ189370	OQ261345	–	–	–	–
8. GuangXi-Pin.	*C. kyotensis*	*C. aconidialis*	AA----	CSF22686	20210622-1-(61)	OQ189371	OQ261346	–	–	–	–
8. GuangXi-Pin.	*C. kyotensis*	*C. aconidialis*	AA----	CSF22687	20210622-1-(63)	OQ189372	OQ261347	–	–	–	–
8. GuangXi-Pin.	*C. kyotensis*	*C. aconidialis*	AA----	CSF22690	20210622-1-(65)	OQ189373	OQ261348	–	–	–	–
8. GuangXi-Pin.	*C. kyotensis*	*C. aconidialis*	AA----	CSF22692	20210622-1-(67)	OQ189374	OQ261349	–	–	–	–
8. GuangXi-Pin.	*C. kyotensis*	*C. aconidialis*	AA----	CSF22693	20210622-1-(69)	OQ189375	OQ261350	–	–	–	–
8. GuangXi-Pin.	*C. kyotensis*	*C. aconidialis*	AA----	CSF22694	20210622-1-(82)	OQ189376	OQ261351	–	–	–	–
8. GuangXi-Pin.	*C. kyotensis*	*C. aconidialis*	AA----	CSF22695	20210622-1-(83)	OQ189377	OQ261352	–	–	–	–
8. GuangXi-Pin.	*C. kyotensis*	*C. aconidialis*	AA----	CSF22697	20210622-1-(92)	OQ189378	OQ261353	–	–	–	–
8. GuangXi-Pin.	*C. kyotensis*	*C. aconidialis*	AT----	CSF22663	20210622-1-(98)	OQ189379	OQ261354	–	–	–	–
8. GuangXi-Pin.	*C. kyotensis*	*C. aconidialis*	AT----	CSF22689	20210622-1-(123)	OQ189380	OQ261355	–	–	–	–
8. GuangXi-Pin.	*C. kyotensis*	*C. hongkongensis*	AGBAAA	CSF22662	20210622-1-(21)	OQ189381	OQ261356	OQ261546	OQ302981	OQ303188	OQ303394
8. GuangXi-Pin.	*C. kyotensis*	*C. ilicicola*	AA----	CSF22679	20210622-1-(54)	OQ189382	OQ261357	–	–	–	–
8. GuangXi-Pin.	*C. kyotensis*	*C. ilicicola*	AA----	CSF22691	20210622-1-(71)	OQ189383	OQ261358	–	–	–	–
8. GuangXi-Pin.	*C. kyotensis*	*C. ilicicola*	AAAAAA	CSF22680	20210622-1-(55)	OQ189384	OQ261359	OQ261551	OQ302986	OQ303193	OQ303399
8. GuangXi-Pin.	*C. kyotensis*	*C. kyotensis*	CADDAA	CSF22683	20210622-1-(58)	OQ189385	OQ261360	OQ261592	OQ303027	OQ303233	OQ303440
8. GuangXi-Pin.	*C. kyotensis*	*C. kyotensis*	CNDBAA	CSF22646	20210621-3-(21)	OQ189386	OQ261361	OQ261605	OQ303040	OQ303246	OQ303453
8. GuangXi-Pin.	*C. kyotensis*	*C. kyotensis*	CODBAA	CSF22665	20210622-1-(24)	OQ189387	OQ261362	OQ261612	OQ303047	OQ303253	OQ303460
8. GuangXi-Pin.	*C. kyotensis*	*C. kyotensis*	CODDAA	CSF22698	20210622-1-(129)	OQ189388	OQ261363	OQ261614	OQ303049	OQ303255	OQ303462
8. GuangXi-Pin.	*C. kyotensis*	*C. kyotensis*	CSDBAA	CSF22688	20210622-1-(66)	OQ189389	OQ261364	OQ261619	OQ303054	OQ303260	OQ303467
8. GuangXi-Pin.	*C. kyotensis*	*C. kyotensis*	CSDBAA	CSF22696	20210622-1-(106)	OQ189390	OQ261365	OQ261620	OQ303055	OQ303261	OQ303468
8. GuangXi-Pin.	*C. kyotensis*	*C. kyotensis*	CTDBAA	CSF22669	20210622-1-(38)	OQ189391	OQ261366	OQ261621	OQ303056	OQ303262	OQ303469
8. GuangXi-Pin.	*C. kyotensis*	*C. kyotensis*	CUDAAA	CSF22667	20210622-1-(34)	OQ189392	OQ261367	OQ261623	OQ303058	OQ303264	OQ303471
9. GuangXi-Cun.	*C. kyotensis*	*C. aconidialis*	AA----	CSF22618	20210621-4-(38)	OQ189393	OQ261368	–	–	–	–
9. GuangXi-Cun.	*C. kyotensis*	*C. aconidialis*	AA----	CSF22619	20210623-1-(5)	OQ189394	OQ261369	–	–	–	–
9. GuangXi-Cun.	*C. kyotensis*	*C. aconidialis*	AA----	CSF22621	20210623-1-(31)	OQ189395	OQ261370	–	–	–	–
9. GuangXi-Cun.	*C. kyotensis*	*C. aconidialis*	AA----	CSF22622	20210623-1-(32)	OQ189396	OQ261371	–	–	–	–
9. GuangXi-Cun.	*C. kyotensis*	*C. aconidialis*	AA----	CSF22624	20210623-1-(46)	OQ189397	OQ261372	–	–	–	–
9. GuangXi-Cun.	*C. kyotensis*	*C. aconidialis*	AA----	CSF22625	20210623-1-(48)	OQ189398	OQ261373	–	–	–	–
9. GuangXi-Cun.	*C. kyotensis*	*C. aconidialis*	AA----	CSF22628	20210623-1-(79)	OQ189399	OQ261374	–	–	–	–
9. GuangXi-Cun.	*C. kyotensis*	*C. aconidialis*	AA----	CSF22629	20210623-1-(82)	OQ189400	OQ261375	–	–	–	–
9. GuangXi-Cun.	*C. kyotensis*	*C. aconidialis*	AA----	CSF22630	20210623-1-(88)	OQ189401	OQ261376	–	–	–	–
9. GuangXi-Cun.	*C. kyotensis*	*C. aconidialis*	AA----	CSF22631	20210623-1-(95)	OQ189402	OQ261377	–	–	–	–
9. GuangXi-Cun.	*C. kyotensis*	*C. aconidialis*	AA----	CSF22633	20210623-1-(100)	OQ189403	OQ261378	–	–	–	–
9. GuangXi-Cun.	*C. kyotensis*	*C. aconidialis*	AA----	CSF22635	20210623-1-(104)	OQ189404	OQ261379	–	–	–	–
9. GuangXi-Cun.	*C. kyotensis*	*C. aconidialis*	AA----	CSF22636	20210623-1-(105)	OQ189405	OQ261380	–	–	–	–
9. GuangXi-Cun.	*C. kyotensis*	*C. aconidialis*	AA----	CSF22637	20210623-1-(106)	OQ189406	OQ261381	–	–	–	–
9. GuangXi-Cun.	*C. kyotensis*	*C. aconidialis*	AA----	CSF22639	20210623-1-(126)	OQ189407	OQ261382	–	–	–	–
9. GuangXi-Cun.	*C. kyotensis*	*C. aconidialis*	AA----	CSF22640	20210623-1-(131)	OQ189408	OQ261383	–	–	–	–
9. GuangXi-Cun.	*C. kyotensis*	*C. aconidialis*	AA----	CSF22641	20210623-1-(141)	OQ189409	OQ261384	–	–	–	–
9. GuangXi-Cun.	*C. kyotensis*	*C. ilicicola*	AA----	CSF22620	20210623-1-(27)	OQ189410	OQ261385	–	–	–	–
9. GuangXi-Cun.	*C. kyotensis*	*C. ilicicola*	AA----	CSF22634	20210623-1-(101)	OQ189411	OQ261386	–	–	–	–
9. GuangXi-Cun.	*C. kyotensis*	*C. ilicicola*	AA----	CSF22638	20210623-1-(113)	OQ189412	OQ261387	–	–	–	–
9. GuangXi-Cun.	*C. kyotensis*	*C. ilicicola*	AAAA_A	CSF22632	20210623-1-(96)	OQ189413	OQ261388	OQ261552	OQ302987	–	OQ303400
10. YunNan-Euc.	*C. kyotensis*	*C. aconidialis*	AK----	CSF22699	20210708-1-(1)	OQ189414	OQ261389	–	–	–	–
10. YunNan-Euc.	*C. kyotensis*	*C. aconidialis*	AK----	CSF22701	20210708-1-(2)	OQ189415	OQ261390	–	–	–	–
10. YunNan-Euc.	*C. kyotensis*	*C. aconidialis*	AK----	CSF22703	20210708-1-(3)	OQ189416	OQ261391	–	–	–	–
10. YunNan-Euc.	*C. kyotensis*	*C. aconidialis*	AK----	CSF22715	20210708-1-(14)	OQ189417	OQ261392	–	–	–	–
10. YunNan-Euc.	*C. kyotensis*	*C. aconidialis*	AK----	CSF22719	20210708-1-(16)	OQ189418	OQ261393	–	–	–	–
10. YunNan-Euc.	*C. kyotensis*	*C. aconidialis*	AK----	CSF22721	20210708-1-(17)	OQ189419	OQ261394	–	–	–	–
10. YunNan-Euc.	*C. kyotensis*	*C. aconidialis*	AK----	CSF22726	20210708-1-(20)	OQ189420	OQ261395	–	–	–	–
10. YunNan-Euc.	*C. kyotensis*	*C. aconidialis*	AK----	CSF22728	20210708-1-(21)	OQ189421	OQ261396	–	–	–	–
10. YunNan-Euc.	*C. kyotensis*	*C. aconidialis*	AK----	CSF22732	20210708-1-(23)	OQ189422	OQ261397	–	–	–	–
10. YunNan-Euc.	*C. kyotensis*	*C. aconidialis*	AK----	CSF22736	20210708-1-(25)	OQ189423	OQ261398	–	–	–	–
10. YunNan-Euc.	*C. kyotensis*	*C. aconidialis*	AK----	CSF23813	20210708-1-(127)	OQ189424	OQ261399	–	–	–	–
10. YunNan-Euc.	*C. kyotensis*	*C. aconidialis*	AK----	CSF23818	20210708-1-(138)	OQ189425	OQ261400	–	–	–	–
10. YunNan-Euc.	*C. kyotensis*	*C. aconidialis*	AK----	CSF23819	20210708-1-(140)	OQ189426	OQ261401	–	–	–	–
10. YunNan-Euc.	*C. kyotensis*	*C. aconidialis*	AK----	CSF23820	20210708-1-(141)	OQ189427	OQ261402	–	–	–	–
10. YunNan-Euc.	*C. kyotensis*	*C. aconidialis*	AK----	CSF23822	20210708-1-(145)	OQ189428	OQ261403	–	–	–	–
10. YunNan-Euc.	*C. kyotensis*	*C. aconidialis*	AK----	CSF23824	20210708-1-(147)	OQ189429	OQ261404	–	–	–	–
10. YunNan-Euc.	*C. kyotensis*	*C. aconidialis*	AK----	CSF23834	20210708-1-(202)	OQ189430	OQ261405	–	–	–	–
10. YunNan-Euc.	*C. kyotensis*	*C. aconidialis*	AK----	CSF23836	20210708-1-(204)	OQ189431	OQ261406	–	–	–	–
10. YunNan-Euc.	*C. kyotensis*	*C. aconidialis*	AK----	CSF23840	20210708-1-(209)	OQ189432	OQ261407	–	–	–	–
10. YunNan-Euc.	*C. kyotensis*	*C. aconidialis*	AK----	CSF23842	20210708-1-(212)	OQ189433	OQ261408	–	–	–	–
10. YunNan-Euc.	*C. kyotensis*	*C. aconidialis*	AK----	CSF23845	20210708-1-(215)	OQ189434	OQ261409	–	–	–	–
10. YunNan-Euc.	*C. kyotensis*	*C. aconidialis*	AK----	CSF23849	20210708-1-(222)	OQ189435	OQ261410	–	–	–	–
10. YunNan-Euc.	*C. kyotensis*	*C. aconidialis*	AK----	CSF23851	20210708-1-(224)	OQ189436	OQ261411	–	–	–	–
10. YunNan-Euc.	*C. kyotensis*	*C. aconidialis*	AK----	CSF23852	20210708-1-(225)	OQ189437	OQ261412	–	–	–	–
10. YunNan-Euc.	*C. kyotensis*	*C. aconidialis*	AKAAAB	CSF22709	20210708-1-(11)	OQ189438	OQ261413	OQ261479	OQ302914	OQ303121	OQ303327
10. YunNan-Euc.	*C. kyotensis*	*C. aconidialis*	AKAAAB	CSF23815	20210708-1-(133)	OQ189439	OQ261414	OQ261480	OQ302915	OQ303122	OQ303328
10. YunNan-Euc.	*C. kyotensis*	*C. aconidialis*	ANAAAA	CSF23811	20210708-1-(103)	OQ189440	OQ261415	OQ261485	OQ302920	OQ303127	OQ303333
10. YunNan-Euc.	*C. kyotensis*	*C. asiatica*	AA----	CSF22705	20210708-1-(6)	OQ189441	OQ261416	–	–	–	–
10. YunNan-Euc.	*C. kyotensis*	*C. asiatica*	AA----	CSF22711	20210708-1-(12)	OQ189442	OQ261417	–	–	–	–
10. YunNan-Euc.	*C. kyotensis*	*C. asiatica*	AA----	CSF22713	20210708-1-(13)	OQ189443	OQ261418	–	–	–	–
10. YunNan-Euc.	*C. kyotensis*	*C. asiatica*	AA----	CSF22717	20210708-1-(15)	OQ189444	OQ261419	–	–	–	–
10. YunNan-Euc.	*C. kyotensis*	*C. asiatica*	AA----	CSF22725	20210708-1-(19)	OQ189445	OQ261420	–	–	–	–
10. YunNan-Euc.	*C. kyotensis*	*C. asiatica*	AA----	CSF22730	20210708-1-(22)	OQ189446	OQ261421	–	–	–	–
10. YunNan-Euc.	*C. kyotensis*	*C. asiatica*	AA----	CSF22734	20210708-1-(24)	OQ189447	OQ261422	–	–	–	–
10. YunNan-Euc.	*C. kyotensis*	*C. asiatica*	AA----	CSF23799	20210708-1-(34)	OQ189448	OQ261423	–	–	–	–
10. YunNan-Euc.	*C. kyotensis*	*C. asiatica*	AA----	CSF23816	20210708-1-(135)	OQ189449	OQ261424	–	–	–	–
10. YunNan-Euc.	*C. kyotensis*	*C. asiatica*	AA----	CSF23817	20210708-1-(137)	OQ189450	OQ261425	–	–	–	–
10. YunNan-Euc.	*C. kyotensis*	*C. asiatica*	AA----	CSF23838	20210708-1-(207)	OQ189451	OQ261426	–	–	–	–
10. YunNan-Euc.	*C. kyotensis*	*C. asiatica*	AA----	CSF23839	20210708-1-(208)	OQ189452	OQ261427	–	–	–	–
10. YunNan-Euc.	*C. kyotensis*	*C. asiatica*	AA----	CSF23841	20210708-1-(211)	OQ189453	OQ261428	–	–	–	–
10. YunNan-Euc.	*C. kyotensis*	*C. asiatica*	AA----	CSF23843	20210708-1-(213)	OQ189454	OQ261429	–	–	–	–
10. YunNan-Euc.	*C. kyotensis*	*C. asiatica*	AA----	CSF23844	20210708-1-(214)	OQ189455	OQ261430	–	–	–	–
10. YunNan-Euc.	*C. kyotensis*	*C. asiatica*	AA----	CSF23846	20210708-1-(217)	OQ189456	OQ261431	–	–	–	–
10. YunNan-Euc.	*C. kyotensis*	*C. asiatica*	AA----	CSF23847	20210708-1-(220)	OQ189457	OQ261432	–	–	–	–
10. YunNan-Euc.	*C. kyotensis*	*C. asiatica*	AA----	CSF23848	20210708-1-(221)	OQ189458	OQ261433	–	–	–	–
10. YunNan-Euc.	*C. kyotensis*	*C. asiatica*	AA----	CSF23850	20210708-1-(223)	OQ189459	OQ261434	–	–	–	–
10. YunNan-Euc.	*C. kyotensis*	*C. asiatica*	AAAAAA	CSF22708	20210708-1-(9)	OQ189460	OQ261435	OQ261514	OQ302949	OQ303156	OQ303362
10. YunNan-Euc.	*C. kyotensis*	*C. asiatica*	AAAAAA	CSF23833	20210708-1-(201)	OQ189461	OQ261436	OQ261515	OQ302950	OQ303157	OQ303363
10. YunNan-Euc.	*C. kyotensis*	*C. asiatica*	AB----	CSF22723	20210708-1-(18)	OQ189462	OQ261437	–	–	–	–
10. YunNan-Euc.	*C. kyotensis*	*C. asiatica*	AB----	CSF23798	20210708-1-(33)	OQ189463	OQ261438	–	–	–	–
10. YunNan-Euc.	*C. kyotensis*	*C. asiatica*	AB----	CSF23808	20210708-1-(75)	OQ189464	OQ261439	–	–	–	–
10. YunNan-Euc.	*C. kyotensis*	*C. asiatica*	AB----	CSF23823	20210708-1-(146)	OQ189465	OQ261440	–	–	–	–
10. YunNan-Euc.	*C. kyotensis*	*C. asiatica*	AB----	CSF23826	20210708-1-(150)	OQ189466	OQ261441	–	–	–	–
10. YunNan-Euc.	*C. kyotensis*	*C. asiatica*	AB----	CSF23835	20210708-1-(203)	OQ189467	OQ261442	–	–	–	–
10. YunNan-Euc.	*C. kyotensis*	*C. asiatica*	AB----	CSF23837	20210708-1-(205)	OQ189468	OQ261443	–	–	–	–
10. YunNan-Euc.	*C. kyotensis*	*C. asiatica*	ABAAAA	CSF23796	20210708-1-(28)	OQ189469	OQ261444	OQ261516	OQ302951	OQ303158	OQ303364
10. YunNan-Euc.	*C. kyotensis*	*C. asiatica*	ABAAAB	CSF23830	20210708-1-(180)	OQ189470	OQ261445	OQ261517	OQ302952	OQ303159	OQ303365
10. YunNan-Euc.	*C. kyotensis*	*C. ilicicola*	CA----	CSF23807	20210708-1-(65)	OQ189471	OQ261446	–	–	–	–
10. YunNan-Euc.	*C. kyotensis*	*C. ilicicola*	CA----	CSF23812	20210708-1-(126)	OQ189472	OQ261447	–	–	–	–
10. YunNan-Euc.	*C. kyotensis*	*C. ilicicola*	CA----	CSF23853	20210708-1-(232)	OQ189473	OQ261448	–	–	–	–
10. YunNan-Euc.	*C. kyotensis*	*C. ilicicola*	CAAABB	CSF23806	20210708-1-(59)	OQ189474	OQ261449	OQ261556	OQ302991	OQ303197	OQ303404
10. YunNan-Euc.	*C. kyotensis*	*C. ilicicola*	CAAABB	CSF23829	20210708-1-(178)	OQ189475	OQ261450	OQ261557	OQ302992	OQ303198	OQ303405
10. YunNan-Euc.	*C. kyotensis*	*C. yunnanensis*	AAAAAA	CSF23797	20210708-1-(31)	OQ189476	OQ261451	OQ261661	OQ303096	OQ303302	OQ303509
10. YunNan-Euc.	*C. kyotensis*	*C. yunnanensis*	ABAAAA	CSF23805	20210708-1-(47)	OQ189477	OQ261452	OQ261662	OQ303097	OQ303303	OQ303510
10. YunNan-Euc.	*C. colhounii*	*C. eucalypti*	AA----	CSF23825	20210708-1-(148)	OQ189478	OQ261453	–	–	–	–
10. YunNan-Euc.	*C. colhounii*	*C. eucalypti*	AA----	CSF23831	20210708-1-(194)	OQ189479	OQ261454	–	–	–	–
10. YunNan-Euc.	*C. colhounii*	*C. eucalypti*	AAAAAA	CSF23802	20210708-1-(41)	OQ189480	OQ261455	OQ261663	OQ303098	OQ303304	OQ303511
10. YunNan-Euc.	*C. colhounii*	*C. eucalypti*	AAAAAA	CSF23828	20210708-1-(162)	OQ189481	OQ261456	OQ261664	OQ303099	OQ303305	OQ303512
10. YunNan-Euc.	*C. colhounii*	*C. eucalypti*	BAAAAA	CSF23809	20210708-1-(88)	OQ189482	OQ261457	OQ261665	OQ303100	OQ303306	OQ303513
10. YunNan-Euc.	*C. colhounii*	*C. eucalypti*	BAAAAA	CSF23832	20210708-1-(197)	OQ189483	OQ261458	OQ261666	OQ303101	OQ303307	OQ303514
10. YunNan-Euc.	*C. colhounii*	*C. eucalypti*	CAAAAA	CSF23800	20210708-1-(37)	OQ189484	OQ261459	OQ261667	OQ303102	OQ303308	OQ303515
10. YunNan-Euc.	*C. colhounii*	*C. eucalypti*	DAAAAA	CSF23810	20210708-1-(99)	OQ189485	OQ261460	OQ261668	OQ303103	OQ303309	OQ303516
11. YunNan-Pin.	*C. colhounii*	*C. eucalypti*	DAAAAA	CSF23854	20210709-1-(224)	OQ189486	OQ261461	OQ261669	OQ303104	OQ303310	OQ303517
12. YunNan-Cun.	*C. kyotensis*	*C. canadiana*	AAAAAA	CSF22750	20210707-1-(141)	OQ189487	OQ261462	OQ261518	OQ302953	OQ303160	OQ303366

^a^ Code of 12 sampling sites connecting to “Site and Tree species code” in Table 1. ^b^ Genotype within each *Calonectria* species, determined by sequences of the *tef1*, *tub2*, *cmdA*, *his3*, *rpb2* and *act* regions; “-” means not available. ^c^ CSF: Culture Collection located at Research Institute of Fasting-growing Trees (RIFT), Chinese Academy of Forestry, ZhanJiang, GuangDong Province, China. ^d^ Information associated with sample point and isolate, for example, “20210527-1-(1)” indicates sample number “20210527-1-(1)” and isolate from this sample. ^e^ *tef1* = translation elongation factor 1-alpha; *tub2* = β-tubulin; *cmdA* = calmodulin; *his3* = histone H3; *rpb2* = the DNA-directed RNA polymerase II second largest subunit; *act* = actin. ^f^ “–” represents the relative locus was not amplified in this study.

## Appendix B. Isolate Numbers of Each Genotype in The Plantation of Each Tree Species in Each Province for Each *Calonectria* Species

**Table A2:** Isolate numbers of each genotype in the plantation of each tree species in each province for each *Calonectria* species.

*Calonectria* Species	Site Number	Tree Species and Province	Genotype Determined by *tef1* Gene Sequences	Number of Isolates Based on *tef1* Genotype	Genotype Determined by *tub2* Gene Sequences	Number of Isolates Based on *tub2* Genotype	Genotype Determined by *tef1* and *tub2* Gene Sequences	Number of Isolates Based on *tef1* and *tub2* Genotype	Number of Genotype Determined by *tef1* and *tub2* Gene Sequences of Each Species
*C. aconidialis*	1	*E. urophylla* × *E. grandis* in FuJian	11	132	11	109	11_11	92	7
			13	16	14	4	11_14	4	
			14	1	17	1	11_17	1	
					19	19	11_19	19	
					31	16	11_31	16	
							13_11	16	
							14_11	1	
	2	*P. massoniana* in FuJian	11	50	11	44	11_11	37	5
			13	7	19	5	11_19	5	
					26	1	11_26	1	
					31	7	11_31	7	
							13_11	7	
	3	*C. lanceolata* in FuJian	11	66	11	64	11_11	51	7
			13	13	14	1	11_14	1	
					16	1	11_16	1	
					19	5	11_19	5	
					27	1	11_27	1	
					31	7	11_31	7	
							13_11	13	
	4	*E. urophylla* × *E. grandis* in GuangDong	11	191	11	152	11_11	149	10
			17	5	12	1	11_12	1	
					15	1	11_15	1	
					23	3	11_23	3	
					25	3	11_25	1	
					28	4	11_28	4	
					29	1	11_29	1	
					30	31	11_30	31	
							17_11	3	
							17_25	2	
	5	*P. massoniana* in GuangDong	11	131	11	106	11_11	103	10
			17	4	12	2	11_12	2	
					16	7	11_16	7	
					18	1	11_18	1	
					20	1	11_20	1	
					25	1	11_28	1	
					28	1	11_29	1	
					29	1	11_30	15	
					30	15	17_11	3	
							17_25	1	
	6	*C. lanceolata* in GuangDong	11	34	11	31	11_11	30	5
			16	1	18	1	11_18	1	
					23	1	11_23	1	
					30	2	11_30	2	
							16_11	1	
	7	*E. urophylla* × *E. grandis* in GuangXi	11	150	11	134	11_11	130	7
			12	1	13	3	11_13	3	
			15	3	22	10	11_22	10	
					23	1	11_23	1	
					30	6	11_30	6	
							12_11	1	
							15_11	3	
	8	*P. massoniana* in GuangXi	11	34	11	32	11_11	32	2
					30	2	11_30	2	
	9	*C. lanceolata* in GuangXi	11	17	11	17	11_11	17	1
	10	*E. urophylla* × *E. grandis* in YunNan	11	27	21	26	11_21	26	2
					24	1	11_24	1	
	11	*P. massoniana* in YunNan	-	0	-	0	-	0	0
	12	*C. lanceolata* in YunNan	-	0	-	0	-	0	0
*C. kyotensis*	1	*E. urophylla* × *E. grandis* in FuJian	11	12	11	2	11_11	2	14
			12	1	14	1	11_14	1	
			13	3	16	13	11_16	5	
			14	21	19	6	11_19	3	
					21	7	11_28	1	
					22	1	12_28	1	
					23	4	13_16	2	
					27	1	13_23	1	
					28	2	14_16	6	
							14_19	3	
							14_21	7	
							14_22	1	
							14_23	3	
							14_27	1	
	2	*P. massoniana* in FuJian	11	22	11	7	11_11	2	20
			13	5	12	1	11_14	2	
			14	37	14	6	11_16	4	
					16	13	11_19	7	
					19	16	11_21	2	
					20	1	11_22	4	
					21	5	11_25	1	
					22	6	13_12	1	
					23	4	13_16	2	
					25	5	13_19	1	
							13_25	1	
							14_11	5	
							14_14	4	
							14_16	7	
							14_19	8	
							14_20	1	
							14_21	3	
							14_22	2	
							14_23	4	
							14_25	3	
	3	*C. lanceolata* in FuJian	-	0	-	0	-	0	0
	4	*E. urophylla* × *E. grandis* in GuangDong	11	1	14	1	11_19	1	4
			14	3	19	2	14_14	1	
					32	1	14_19	1	
							14_32	1	
	5	*P. massoniana* in GuangDong	11	11	11	4	11_11	1	16
			14	11	13	1	11_14	1	
			15	3	14	1	11_19	2	
					17	1	11_25	5	
					19	3	11_26	1	
					22	2	11_28	1	
					25	9	14_11	2	
					26	1	14_13	1	
					28	3	14_17	1	
							14_19	1	
							14_22	1	
							14_25	3	
							14_28	2	
							15_11	1	
							15_22	1	
							15_25	1	
	6	*C. lanceolata* in GuangDong	13	1	11	2	13_28	1	4
			14	4	14	1	14_11	2	
					22	1	14_14	1	
					28	1	14_22	1	
	7	*E. urophylla* × *E. grandis* in GuangXi	13	22	11	5	13_11	5	9
			14	1	15	3	13_15	2	
					18	1	13_18	1	
					23	3	13_23	3	
					24	1	13_24	1	
					25	6	13_25	6	
					28	3	13_28	3	
					31	1	13_31	1	
							14_15	1	
	8	*P. massoniana* in GuangXi	13	8	11	1	13_11	1	6
					24	1	13_24	1	
					25	2	13_25	2	
					29	2	13_29	2	
					30	1	13_30	1	
					31	1	13_31	1	
	9	*C. lanceolata* in GuangXi	-	0	-	0	-	0	0
	10	*E. urophylla* × *E. grandis* in YunNan	-	0	-	0	-	0	0
	11	*P. massoniana* in YunNan	-	0	-	0	-	0	0
	12	*C. lanceolata* in YunNan	-	0	-	0	-	0	0
*C. hongkongensis*	1	*E. urophylla* × *E. grandis* in FuJian	11	27	11	21	11_11	21	5
					12	1	11_12	1	
					16	3	11_16	3	
					18	1	11_18	1	
					19	1	11_19	1	
	2	*P. massoniana* in FuJian	11	39	11	26	11_11	26	6
					12	2	11_12	2	
					14	1	11_14	1	
					15	1	11_15	1	
					16	7	11_16	7	
					17	2	11_17	2	
	3	*C. lanceolata* in FuJian	11	6	11	4	11_11	4	3
					14	1	11_14	1	
					16	1	11_16	1	
	4	*E. urophylla* × *E. grandis* in GuangDong	11	12	11	6	11_11	6	5
					12	2	11_12	2	
					13	2	11_13	2	
					14	1	11_14	1	
					16	1	11_16	1	
	5	*P. massoniana* in GuangDong	11	50	11	35	11_11	35	7
					12	3	11_12	3	
					13	1	11_13	1	
					14	3	11_14	3	
					15	2	11_15	2	
					16	5	11_16	5	
					18	1	11_18	1	
	6	*C. lanceolata* in GuangDong	-	0	-	0	-	0	0
	7	*E. urophylla* × *E. grandis* in GuangXi	11	1	16	2	11_16	1	2
			12	1			12_16	1	
	8	*P. massoniana* in GuangXi	11	1	17	1	11_17	1	1
	9	*C. lanceolata* in GuangXi	-	0	-	0	-	0	0
	10	*E. urophylla* × *E. grandis* in YunNan	-	0	-	0	-	0	0
	11	*P. massoniana* in YunNan	-	0	-	0	-	0	0
	12	*C. lanceolata* in YunNan	-	0	-	0	-	0	0
*C. ilicicola*	1	*E. urophylla* × *E. grandis* in FuJian	11	3	11	3	11_11	3	1
	2	*P. massoniana* in FuJian	11	4	11	4	11_11	4	1
	3	*C. lanceolata* in FuJian	11	4	11	4	11_11	4	1
	4	*E. urophylla* × *E. grandis* in GuangDong	12	1	11	1	12_11	1	1
	5	*P. massoniana* in GuangDong	12	2	11	2	12_11	2	1
	6	*C. lanceolata* in GuangDong	11	3	11	3	11_11	3	1
	7	*E. urophylla* × *E. grandis* in GuangXi	11	3	11	3	11_11	3	1
	8	*P. massoniana* in GuangXi	11	3	11	3	11_11	3	1
	9	*C. lanceolata* in GuangXi	11	4	11	4	11_11	4	1
	10	*E. urophylla* × *E. grandis* in YunNan	13	5	11	5	13_11	5	1
	11	*P. massoniana* in YunNan	-	0	-	0	-	0	0
	12	*C. lanceolata* in YunNan	-	0	-	0	-	0	0
*C. asiatica*	1	*E. urophylla* × *E. grandis* in FuJian	-	0	-	0	-	0	0
	2	*P. massoniana* in FuJian	-	0	-	0	-	0	0
	3	*C. lanceolata* in FuJian	-	0	-	0	-	0	0
	4	*E. urophylla* × *E. grandis* in GuangDong	-	0	-	0	-	0	0
	5	*P. massoniana* in GuangDong	-	0	-	0	-	0	0
	6	*C. lanceolata* in GuangDong	-	0	-	0	-	0	0
	7	*E. urophylla* × *E. grandis* in GuangXi	-	0	-	0	-	0	0
	8	*P. massoniana* in GuangXi	-	0	-	0	-	0	0
	9	*C. lanceolata* in GuangXi	-	0	-	0	-	0	0
	10	*E. urophylla* × *E. grandis* in YunNan	11	30	11	21	11_11	21	2
					12	9	11_12	9	
	11	*P. massoniana* in YunNan	-	0	-	0	-	0	0
	12	*C. lanceolata* in YunNan	-	0	-	0	-	0	0
*C. eucalypti*	1	*E. urophylla* × *E. grandis* in FuJian	-	0	-	0	-	0	0
	2	*P. massoniana* in FuJian	-	0	-	0	-	0	0
	3	*C. lanceolata* in FuJian	-	0	-	0	-	0	0
	4	*E. urophylla* × *E. grandis* in GuangDong	-	0	-	0	-	0	0
	5	*P. massoniana* in GuangDong	-	0	-	0	-	0	0
	6	*C. lanceolata* in GuangDong	-	0	-	0	-	0	0
	7	*E. urophylla* × *E. grandis* in GuangXi	-	0	-	0	-	0	0
	8	*P. massoniana* in GuangXi	-	0	-	0	-	0	0
	9	*C. lanceolata* in GuangXi	-	0	-	0	-	0	0
	10	*E. urophylla* × *E. grandis* in YunNan	11	4	11	8	11_11	4	4
			12	2			12_11	2	
			13	1			13_11	1	
			14	1			14_11	1	
	11	*P. massoniana* in YunNan	14	1	11	1	14_11	1	1
	12	*C. lanceolata* in YunNan	-	0	-	0	-	0	0
*C. curvispora*	1	*E. urophylla* × *E. grandis* in FuJian	-	0	-	0	-	0	0
	2	*P. massoniana* in FuJian	-	0	-	0	-	0	0
	3	*C. lanceolata* in FuJian	-	0	-	0	-	0	0
	4	*E. urophylla* × *E. grandis* in GuangDong	-	0	-	0	-	0	0
	5	*P. massoniana* in GuangDong	11	4	11	4	11_11	4	1
	6	*C. lanceolata* in GuangDong	-	0	-	0	-	0	0
	7	*E. urophylla* × *E. grandis* in GuangXi	-	0	-	0	-	0	0
	8	*P. massoniana* in GuangXi	-	0	-	0	-	0	0
	9	*C. lanceolata* in GuangXi	-	0	-	0	-	0	0
	10	*E. urophylla* × *E. grandis* in YunNan	-	0	-	0	-	0	0
	11	*P. massoniana* in YunNan	-	0	-	0	-	0	0
	12	*C. lanceolata* in YunNan	-	0	-	0	-	0	0
*C. chinensis*	1	*E. urophylla* × *E. grandis* in FuJian	11	1	11	1	11_11	1	1
	2	*P. massoniana* in FuJian	-	0	-	0	-	0	0
	3	*C. lanceolata* in FuJian	11	1	11	2	11_11	1	2
	4		12	1			12_11	1	
	5	*E. urophylla* × *E. grandis* in GuangDong	-	0	-	0	-	0	0
	6	*P. massoniana* in GuangDong	-	0	-	0	-	0	0
	7	*C. lanceolata* in GuangDong	-	0	-	0	-	0	0
	8	*E. urophylla* × *E. grandis* in GuangXi	-	0	-	0	-	0	0
	9	*P. massoniana* in GuangXi	-	0	-	0	-	0	0
	10	*C. lanceolata* in GuangXi	-	0	-	0	-	0	0
	11	*E. urophylla* × *E. grandis* in YunNan	-	0	-	0	-	0	0
	11	*P. massoniana* in YunNan	-	0	-	0	-	0	0
	12	*C. lanceolata* in YunNan	-	0	-	0	-	0	0
*C. pacifica*	1	*E. urophylla* × *E. grandis* in FuJian	-	0	-	0	-	0	0
	2	*P. massoniana* in FuJian	-	0	-	0	-	0	0
	3	*C. lanceolata* in FuJian	-	0	-	0	-	0	0
	4	*E. urophylla* × *E. grandis* in GuangDong	-	0	-	0	-	0	0
	5	*P. massoniana* in GuangDong	11	1	11	1	11_11	1	2
	6		12	1	12	1	12_12	1	
	7	*C. lanceolata* in GuangDong	11	1	13	1	11_13	1	1
	8	*E. urophylla* × *E. grandis* in GuangXi	-	0	-	0	-	0	0
	9	*P. massoniana* in GuangXi	-	0	-	0	-	0	0
	10	*C. lanceolata* in GuangXi	-	0	-	0	-	0	0
		*E. urophylla × E. grandis* in YunNan	-	0	-	0	-	0	0
	11	*P. massoniana* in YunNan	-	0	-	0	-	0	0
	12	*C. lanceolata* in YunNan	-	0	-	0	-	0	0
*C. yunnanensis*	1	*E. urophylla × E. grandis* in FuJian	-	0	-	0	-	0	0
	2	*P. massoniana* in FuJian	-	0	-	0	-	0	0
	3	*C. lanceolata* in FuJian	-	0	-	0	-	0	0
	4	*E. urophylla × E. grandis* in GuangDong	-	0	-	0	-	0	0
	5	*P. massoniana* in GuangDong	-	0	-	0	-	0	0
	6	*C. lanceolata* in GuangDong	-	0	-	0	-	0	0
	7	*E. urophylla × E. grandis* in GuangXi	-	0	-	0	-	0	0
	8	*P. massoniana* in GuangXi	-	0	-	0	-	0	0
	9	*C. lanceolata* in GuangXi	-	0	-	0	-	0	0
	10	*E. urophylla × E. grandis* in YunNan	11	2	11	1	11_11	1	2
					12	1	11_12	1	
	11	*P. massoniana* in YunNan	-	0	-	0	-	0	0
	12	*C. lanceolata* in YunNan	-	0	-	0	-	0	0
*C. canadiana*	1	*E. urophylla × E. grandis* in FuJian	-	0	-	0	-	0	0
	2	*P. massoniana* in FuJian	-	0	-	0	-	0	0
	3	*C. lanceolata* in FuJian	-	0	-	0	-	0	0
	4	*E. urophylla × E. grandis* in GuangDong	-	0	-	0	-	0	0
	5	*P. massoniana* in GuangDong	-	0	-	0	-	0	0
	6	*C. lanceolata* in GuangDong	-	0	-	0	-	0	0
	7	*E. urophylla × E. grandis* in GuangXi	-	0	-	0	-	0	0
	8	*P. massoniana* in GuangXi	-	0	-	0	-	0	0
	9	*C. lanceolata* in GuangXi	-	0	-	0	-	0	0
	10	*E. urophylla × E. grandis* in YunNan	-	0	-	0	-	0	0
	11	*P. massoniana* in YunNan	-	0	-	0	-	0	0
	12	*C. lanceolata* in YunNan	11	1	11	1	11_11	1	1

## Appendix C. Number of Shared Genotypes of *Calonectria cconidialis* Determined by *tef1* and *tub2* Gene Sequences between Different Plantation Tree Species × Province

**Table A3:** Number of shared genotypes of *Calonectria aconidialis* determined by *tef1* and *tub2* gene sequences between different plantation tree species × province.

	*E. urophylla* × *E. grandis* in FuJian	*P. massoniana* in FuJian	*C. lanceolata* in FuJian	*E. urophylla* × *E. grandis* in GuangDong	*P. massoniana* in GuangDong	*C. lanceolata* in GuangDong	*E. urophylla* × *E. grandis* in GuangXi	*P. massoniana* in GuangXi	*C. lanceolata* in GuangXi	*E. urophylla* × *E. grandis* in YunNan	*P. massoniana* in YunNan	*C. lanceolata* in YunNan
*E. urophylla* × *E. grandis* in FuJian		4 ^1^	5	1 ^2^	1 ^3^	1	1	1	1	0	0	0
*P. massoniana* in FuJian			4	1	1	1	1	1	1	0	0	0
*C. lanceolata* in FuJian				1	2	1	1	1	1	0	0	0
*E. urophylla* × *E. grandis* in GuangDong					7	3	3	2	1	0	0	0
*P. massoniana* in GuangDong						3	2	2	1	0	0	0
*C. lanceolata* in GuangDong							3	2	1	0	0	0
*E. urophylla* × *E. grandis* in GuangXi								2	1	0	0	0
*P. massoniana* in GuangXi									1	0	0	0
*C. lanceolata* in GuangXi										0	0	0
*E. urophylla* × *E. grandis* in YunNan											0	0
*P. massoniana* in YunNan												0
*C. lanceolata* in YunNan												

^1^ Number highlighted in blue indicates number of shared genotypes between different tree species in the same sampled site (province). ^2^ Number highlighted in yellow indicates number of shared genotypes between different sampled sites of the same tree species. ^3^ Number highlighted in grey indicates number of shared genotypes between different sampled sites of different tree species.

## Appendix D. Number of Shared Genotypes of *Calonectria kyotensis* Determined by *tef1* and *tub2* Gene Sequences between Different Plantation Tree Species × Province

**Table A4:** Number of shared genotypes of *Calonectria kyotensis* determined by *tef1* and *tub2* gene sequences between different plantation tree species × province.

	*E. urophylla* × *E. grandis* in FuJian	*P. massoniana* in FuJian	*C. lanceolata* in FuJian	*E. urophylla* × *E. grandis* in GuangDong	*P. massoniana* in GuangDong	*C. lanceolata* in GuangDong	*E. urophylla* × *E. grandis* in GuangXi	*P. massoniana* in GuangXi	*C. lanceolata* in GuangXi	*E. urophylla* × *E. grandis* in YunNan	*P. massoniana* in YunNan	*C. lanceolata* in YunNan
*E. urophylla* × *E. grandis* in FuJian		10 ^1^	0	2 ^2^	6 ^3^	1	1	0	0	0	0	0
*P. massoniana* in FuJian			0	3	8	3	1	1	0	0	0	0
*C. lanceolata* in FuJian				0	0	0	0	0	0	0	0	0
*E. urophylla* × *E. grandis* in GuangDong					2	1	0	0	0	0	0	0
*P. massoniana* in GuangDong						2	0	0	0	0	0	0
*C. lanceolata* in GuangDong							1	0	0	0	0	0
*E. urophylla* × *E. grandis* in GuangXi								4	0	0	0	0
*P. massoniana* in GuangXi									0	0	0	0
*C. lanceolata* in GuangXi										0	0	0
*E. urophylla* × *E. grandis* in YunNan											0	0
*P. massoniana* in YunNan												0
*C. lanceolata* in YunNan												

^1^ Number highlighted in blue indicates number of shared genotypes between different tree species in the same sampled site (province). ^2^ Number highlighted in yellow indicates number of shared genotypes between different sampled sites of the same tree species. ^3^ Number highlighted in grey indicates number of shared genotypes between different sampled sites of different tree species.

## Appendix E. Number of Shared Genotypes of *Calonectria hongkongensis* Determined by *tef1* and *tub2* Gene Sequences between Different Plantation Tree Species × Province

**Table A5:** Number of shared genotypes of *Calonectria hongkongensis* determined by *tef1* and *tub2* gene sequences between different plantation tree species × province.

	*E. urophylla* × *E. grandis* in FuJian	*P. massoniana* in FuJian	*C. lanceolata* in FuJian	*E. urophylla* × *E. grandis* in GuangDong	*P. massoniana* in GuangDong	*C. lanceolata* in GuangDong	*E. urophylla* × *E. grandis* in GuangXi	*P. massoniana* in GuangXi	*C. lanceolata* in GuangXi	*E. urophylla* × *E. grandis* in YunNan	*P. massoniana* in YunNan	*C. lanceolata* in YunNan
*E. urophylla* × *E. grandis* in FuJian		3 ^1^	2	3 ^2^	4 ^3^	0	1	0	0	0	0	0
*P. massoniana* in FuJian			3	4	5	0	1	1	0	0	0	0
*C. lanceolata* in FuJian				3	3	0	1	0	0	0	0	0
*E. urophylla* × *E. grandis* in GuangDong					5	0	1	0	0	0	0	0
*P. massoniana* in GuangDong						0	1	0	0	0	0	0
*C. lanceolata* in GuangDong							0	0	0	0	0	0
*E. urophylla* × *E. grandis* in GuangXi								0	0	0	0	0
*P. massoniana* in GuangXi									0	0	0	0
*C. lanceolata* in GuangXi										0	0	0
*E. urophylla* × *E. grandis* in YunNan											0	0
*P. massoniana* in YunNan												0
*C. lanceolata* in YunNan												

^1^ Number highlighted in blue indicates number of shared genotypes between different tree species in the same sampled site (province). ^2^ Number highlighted in yellow indicates number of shared genotypes between different sampled sites of the same tree species. ^3^ Number highlighted in grey indicates number of shared genotypes between different sampled sites of different tree species.
